# First Description of a Yersinia pseudotuberculosis Clonal Outbreak in France, Confirmed Using a New Core Genome Multilocus Sequence Typing Method

**DOI:** 10.1128/spectrum.01145-22

**Published:** 2022-07-06

**Authors:** Cyril Savin, Anne-Sophie Le Guern, Fanny Chereau, Julien Guglielmini, Guillaume Heuzé, Christian Demeure, Javier Pizarro-Cerdá

**Affiliations:** a Institut Pasteur, Université de Paris Cité, Yersinia Research Unit, Paris, France; b Institut Pasteur, Université de Paris Cité, Yersinia National Reference Laboratory, Paris, France; c Institut Pasteur, Université de Paris Cité, WHO Collaborating Research & Reference Centre for Yersinia FRA-140, Paris, France; d French National Public Health Agency, Department of Infectious Diseases, Saint-Maurice, France; e Institut Pasteur, Université de Paris Cité, Hub de Bioinformatique et Biostatistique, Paris, France; f French National Public Health Agency, Ajaccio, France; University Paris-Saclay, Antoine Béclre, Service de Microbiologie, Institute for Integrative Biology of the Cell (I2BC), CEA, CNRS

**Keywords:** *Yersinia pseudotuberculosis*, outbreak, epidemiological investigation, cgMLST, enteric yersiniosis

## Abstract

Yersinia pseudotuberculosis is an enteric pathogen causing mild enteritis that can lead to mesenteric adenitis in children and septicemia in elderly patients. Most cases are sporadic, but outbreaks have already been described in different countries. We report for the first time a Y. pseudotuberculosis clonal outbreak in France, that occurred in 2020. An epidemiological investigation based on food queries pointed toward the consumption of tomatoes as the suspected source of infection. The *Yersinia* National Reference Laboratory (YNRL) developed a new cgMLST scheme with 1,921 genes specific to Y. pseudotuberculosis that identified the clustering of isolates associated with the outbreak and allowed to perform molecular typing in real time. In addition, this method allowed to retrospectively identify isolates belonging to this cluster from earlier in 2020. This method, which does not require specific bioinformatic skills, is now used systematically at the YNRL and proves to display an excellent discriminatory power and is available to the scientific community.

**IMPORTANCE** We describe in here a novel core-genome MLST method that allowed to identify in real time, and for the first time in France, a Y. pseudotuberculosis clonal outbreak that took place during the summer 2020 in Corsica. Our method allows to support epidemiological and microbiological investigations to establish a link between patients infected with closely associated Y. pseudotuberculosis isolates, and to identify the potential source of infection. In addition, we made this method available for the scientific community.

## INTRODUCTION

The *Yersinia* genus currently encompasses 26 different species, three of them being pathogenic for human: Y. pestis, the highly pathogenic agent of plague, and two enteropathogens, Yersinia enterocolitica and Yersinia pseudotuberculosis (1). Occurring predominantly in children, *Yptb* can cause mild enteritis characterized by fever, abdominal pain, and sometimes diarrhea that can lead to mesenteric adenitis (2). *Yptb* can cause an invasive infection, leading to septicemia in elderly patients or in individuals presenting underlying medical disorders (diabetes, cirrhosis, iron overload) (3). Most of the *Yptb* associated cases are sporadic, but some outbreaks have been reported in different parts of the world, including Japan (4), Canada (5), Europe (6), Russia (7), and more recently in New Zealand (8). The reservoir of *Yptb* is mostly wild mammals (particularly rodents, lagomorphs, wild boars) and birds. The pathogen can enter the food chain and outbreaks caused by consumption of contaminated iceberg lettuce (9), carrots (10), or raw milk (11) have been described.

In the case of an outbreak suspicion, epidemiological investigations are of key importance to establish a link between patients and to identify a common exposure. Molecular investigation methods allowing the establishment of a genetic link between bacterial isolates have been essential in many outbreak investigations to confirm a genetic link between clinical isolates or between clinical and environmental isolates. Pulsed-field gel electrophoresis (PFGE) used to be the gold standard technique (9). However, this method is time-consuming and labor-intensive. Its lack of reproducibility and resolution led to its replacement by multilocus variable-number tandem repeat analysis (MLVA) which has a better discriminatory power but is still time-consuming (12). Recent advances in sequencing methods have made whole-genome sequencing a rapid and affordable approach, available to surveillance laboratories. This has led to the development of core-genome Single Nucleotide Polymorphism (cgSNP) analyses to determine the genetic distance between bacterial isolates, with an excellent discriminatory power (8, 13). Nevertheless, cgSNP analyses require advanced bioinformatic skills and is not yet standardized between laboratories.

In France, the surveillance of enteric yersiniosis is conducted by the *Yersinia* National Reference Laboratory (YNRL) and Santé publique France (SpF), the national public health agency. The routine procedure at the YNRL includes whole-genome sequencing of all bacterial isolates, followed by a bioinformatic analysis using a 500-gene core genome multilocus sequence typing (cgMLST) dedicated to the *Yersinia* genus (14). This method allows identification of the species (including discrimination between *Yptb* and Y. pestis, which cannot be achieved with techniques commonly used in medical laboratories such as MALDI-TOF [15]) and eventually the lineage. Due to its relative low number of genes, this technique is not used to detect clusters. Therefore, the YNRL developed a new, discriminant and easy-to-use public tool for cluster identification. It is based on a cgMLST of 1,921 genes shared by most *Yptb* strains. Upon genome assembly, it does not require manipulation of raw data nor specific bioinformatic skills as it simply relies on a drag&drop upload of the genome through a web-based interface completed by an automatic scan of the genome and the generation of its allelic profile. We hereby describe this tool and highlight its usefulness in the investigation of the first described *Yptb* outbreak in France.

## RESULTS

### Historical diversity of Y. pseudotuberculosis isolates in France.

According to the French YNRL database, among the 324 *Yptb* isolates received between 1991 and 2019, 17 lineages currently circulate in France: isolates from lineages 15 and 10 are the most frequent (76 and 70 isolates, respectively), followed by lineages 17, 5, 7, 2 and 16 (Fig. S1). Even if the number of isolates has been quite stable over time (11.2 ± 5.2 per year since 1991), very few strains were reported in 1997 and 2002 (2 isolates each year) while a peak was observed in 2005 (28 isolates) (Fig. S1).

### Suspicion of a Y. pseudotuberculosis outbreak in France during 2020.

**FIG 1:**
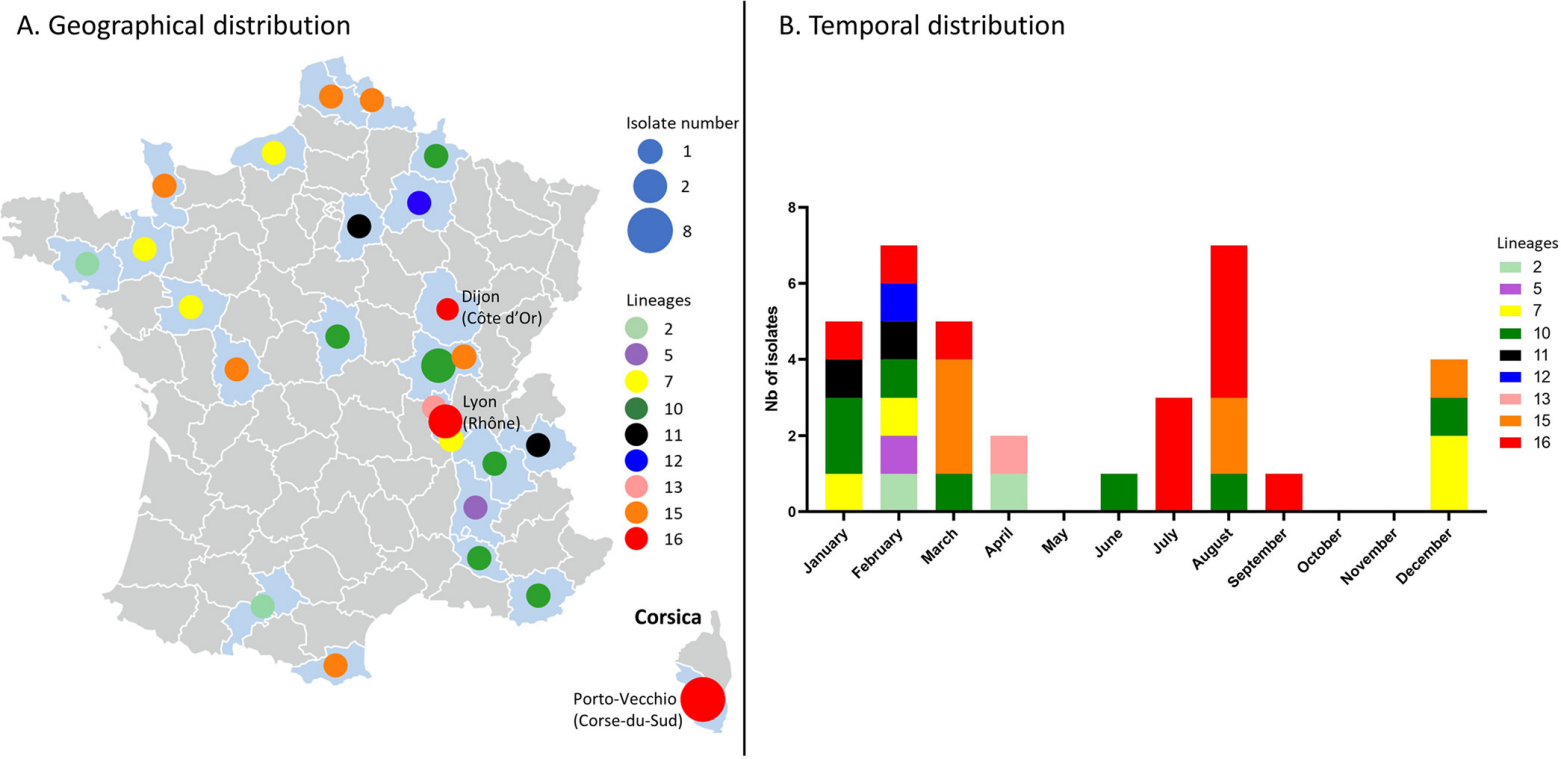
Geographical and temporal distribution of the 35 Y. pseudotuberculosis isolated in France in2020. (A) Map of France with the departments. Size of the circle depends on the number of isolates. Colors of the circles depends on the isolates’ lineages. (B) Number of strains per month. Colors of the squares depends on the isolates’ lineages.

In 2020, enteric yersiniosis surveillance by the YNRL led to the identification of 35 isolates of *Yptb*. Their geographical distribution indicates that, for almost all the lineages, isolates originated from different French departments (Fig. 1A). The 20 ones identified in the first semester belonged to 9 different lineages, lineage 10 being the most frequently isolated (5 isolates) whereas 3 isolates of each lineage 15 and 16 were found (Fig. 1B). At the end of July, 3 additional lineage 16 isolates were identified. The isolation of these specimens by a single medical laboratory in Porto-Vecchio (Corse-du-Sud) led the YNRL to alert SpF (see below) of a potential outbreak concerning *Yptb* lineage 16 (Fig. S2). In August 2020, 4 more lineage 16 isolates were identified, from the same laboratory, together with 3 other isolates (lineage 10 and 15). In September 2020, 2 lineage 16 isolates were identified but they originated from a laboratory located in Lyon (Rhône). Afterward, no more isolates from lineage 16 were reported in 2020. In total, 11 *Yptb* lineage 16 isolates were sent to the YNRL in 2020. Interestingly, in addition to the 8 lineage 16 isolates identified during the summer, 3 lineage 16 specimens were found, 2 by the same laboratory in Corse-du-Sud, and one in Dijon (Côte d’Or) at the beginning of the year. Whereas no *Yptb* were identified in October and November, 4 isolates belonging to 3 different lineages (7, 10 and 15) appeared in December (Fig. S2).

### Epidemiological, trace back and environmental investigations.

On August 12, 2020, the YNRL informed SpF of the identification of 3 patients infected by *Yptb* lineage 16 as determined by a cgMLST 500 genes, isolated in the same week (week 30) in a single medical laboratory in Corsica (Fig. S2). By comparison, 0 to 2 isolates belonging to lineage 16 had been isolated per year in France since 1991 (Fig. S1). Moreover, no *Yptb* had been isolated in the previous years by the medical laboratory using the same identification method. This unusual temporal and geographical group of cases, combined with the potential for invasive infections by *Yptb*, instigated an epidemiological investigation led by SpF and local public health authorities, to identify a potential common source of contamination and to implement control measures.

Cases were defined as any patient with identification of a *Yptb* lineage 16 isolate in the YNRL national database, from any type of specimen sampled from July 1^st^ 2020 in metropolitan France. In total, 8 cases were identified with sampling dates between July 23^rd^ and September 1^st^. One isolate per stool sample was recovered. The 8 *Yptb* isolates came from a laboratory in Porto-Vecchio (Corse-du-Sud department) for 6 patients and in a laboratory in Lyon (Rhône department) for 2 patients. The median age of the patients was 25 years old, with 4 patients between 5 to 15, 3 patients between 30 to 60 and 1 patient older than 90. The patients sex ratio was 1.7 (three women and five men). Interviews were performed for the 8 cases. The onset of the symptoms covered a period from July 10^th^ to August 26^th^. Cases presented with diarrhea (*n* = 7), fever (*n* = 6), abdominal pain (*n* = 5), vomiting (*n* = 2). Most cases have managed their symptoms at home, one 10-year-old child was admitted to the hospital during one night for observation. Two patients were residents of Corsica and 6 were in holidays in Corsica during the incubation period. Moreover, 7 of them were located (residency or holidays) in a 10 km radius area in Northeast of Corsica (Haute-Corse department).

The descriptive analysis of food queries showed that the food item most frequently consumed was tomatoes, consumed by 6 cases (Table 1). Moreover, all 6 cases purchased tomatoes from the same greengrocer's shop X in Northeast of Corsica (6 cases). No common leisure activity or visited place (such as a restaurant) was identified. Seven of the cases resided in an area supplied by the same water distribution network. No contamination episodes of the water distribution network covering the area were identified in the historical records (15 campaigns in 2019).

**TABLE 1:** Food items consumed by 3 or more cases in the week before onset of symptoms, Infections of *Y. pseudotuberculosis*, France, 2020

Food item	Reported consumption^*a*^ (*n* = 8 cases)
Tomatoes	6
Tomatoes from greengrocer X	6
Corsican sheep cheese	4
Pâté	3
Salami	3
Figatellu	3
Nectarines	3
Cucumbers	3

*^a^*For two cases, a family member was interviewed, and the information may not have covered all foods eaten by the case patient.

Food investigation established that the suspected tomatoes originated from a local production unit, based in the same geographical area. On-site inspection did not identify any nonconformity potentially leading to contamination of the tomatoes during production, harvest, storage, packaging, or transport, nor any problem with traceability. Three other companies commercialized tomatoes from the suspected batch, but no trace back could be performed as no sales records were kept. The tomatoes were not rinsed before distribution and the water used for irrigation came from the public distribution network for agricultural use. No verification of the quality of this water system was conducted.

### A new Y. pseudotuberculosis cgMLST confirmed the cluster of isolates.

**FIG 2:**
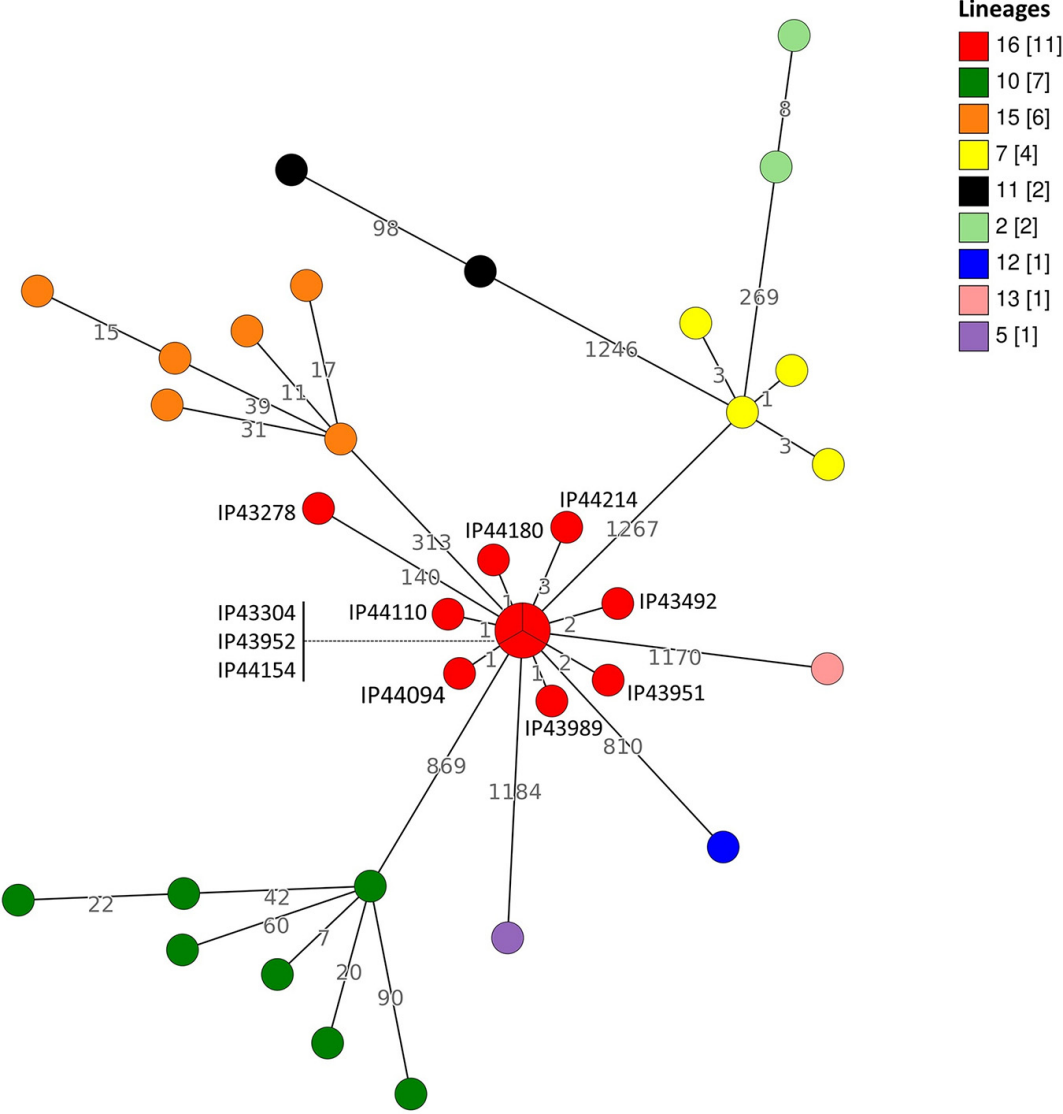
Minimum spanning tree obtained using the allelic profiles of the cgMLST (1,921 genes) on the 35 Y. pseudotuberculosis isolates in France in 2020. The branch lengths are based on a logarithmic scale. Numbers close to the branches reveals the alleles differences. Colors of the circles depends on the isolates’ lineages. Pie charts identifies several isolates with the same allelic profile.

As our newly developed 1,921 genes cgMLST scheme specific to the species *Yptb* is used in routine at the YNRL, identification of the cluster of isolates associated with this pseudotuberculosis outbreak was possible. This new typing method was also applied to the *Yptb* strains isolated in France in 2020 and their genetic relatedness was determined (Table S2 and Fig. 2).

The observed distance between isolates within each lineage (0 to 140 alleles) is much lower than distances observed between isolates from different lineages (269 to 1,267 alleles), confirming that the lineage s are well demarcated from each other using this novel cgMLST (Fig. 2).

Within each lineage 7 and 16, isolates are more closely related to each other compared to isolates within lineages 10 and 15, suggesting more clonality. Lineage 7 isolates (4 specimens) have 1 to 3 allele differences and may be considered belonging to the same cluster. However, no interviews of these patients were conducted to identify a potential common exposure and their distant isolation dates (Fig. 1 and 2) weaken the hypothesis of a common source of infection.

Interestingly, among the 11 lineage 16 isolates, 10 of them showed close genetic relatedness (between 0 and 3 differences) and may be considered belonging to the same cluster. Whereas isolates IP43304 and IP43492 were recovered at the beginning of 2020, the 8 other specimens were isolated within 38 days (from 28^th^ of July to 3^rd^ of September 2020) and correspond to the cases investigated during the summer (see above). The close genetic relatedness of the 8 isolates, together with their close geographical and temporal isolation, confirmed a cluster of cases due to a *Yptb* lineage 16 infection. Interestingly, the 2 isolates IP43304 and IP43492 were also recovered from the laboratory in Porto-Vecchio in February and March 2020. No interviews of these two patients from early 2020 on the exposures were conducted, given the distance to onset of symptoms. The high allele difference number between isolate IP43278 and the other isolates (≥140) from lineage 16 (Fig. 2) excludes IP43278 from the cluster.

### Performance comparison between the novel Y. pseudotuberculosis cgMLST and classical cgSNP analysis.

**FIG 3:**
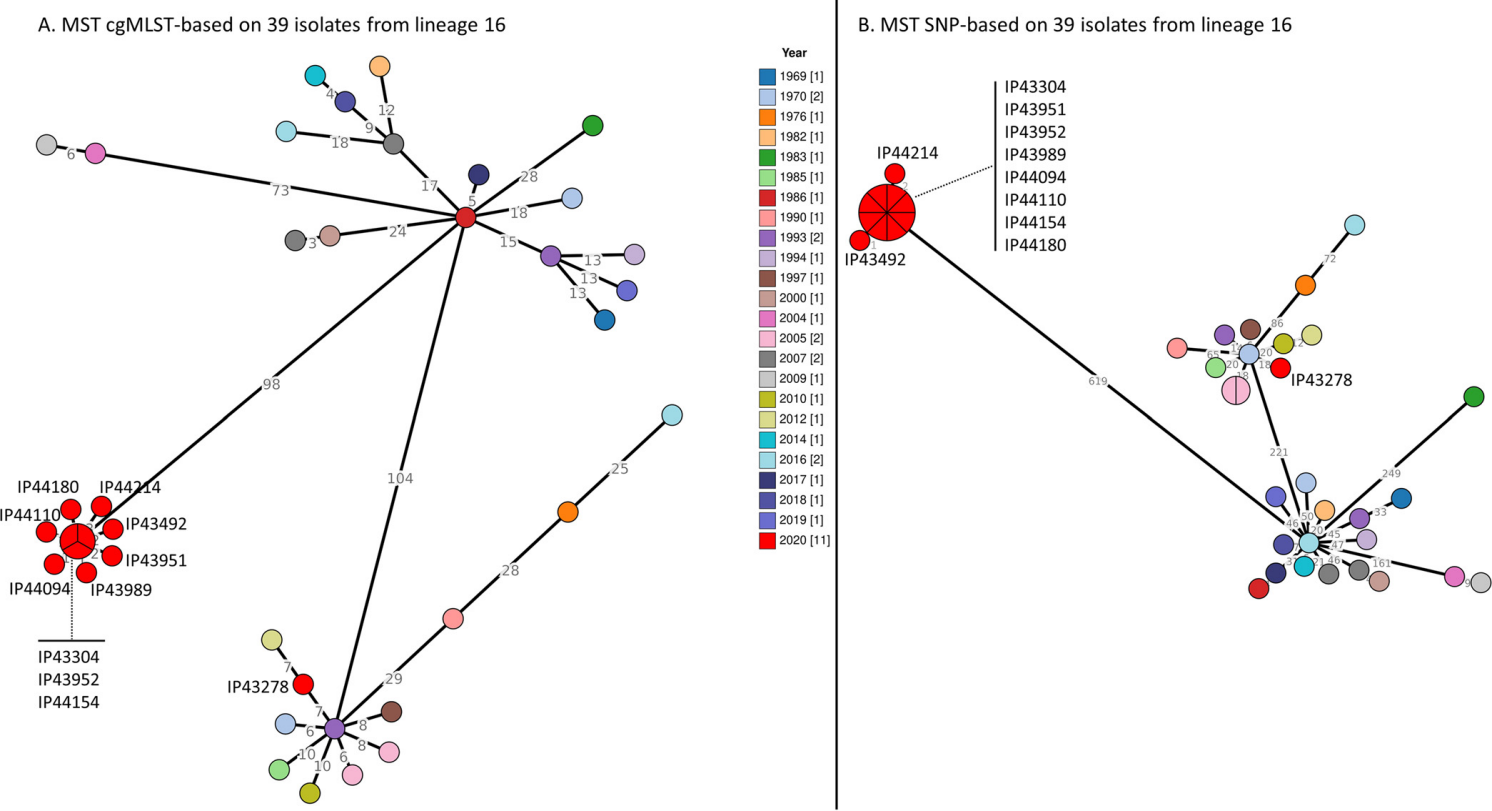
Minimum spanning tree reconstructed on the 39 Y. pseudotuberculosis belonging to the lineage 16 isolated in France,1969 to 2020. (A) MST cgMLST-based (B) MST SNP-based. The branch lengths are based on a linear scale. Numbers close to the branches reveals the alleles differences (A) or SNP differences (B). Colors of the circles depends on the isolates’ lineages. Pie charts identifies several isolates with the same allelic profile (3.A.) or same SNP profile (3.B.).

Taking advantage of our novel, easy-to-use *Yptb* cgMLST scheme, we compared the performance of both methods on the 11 lineage 16 isolates from 2020 together with 28 additional lineage 16 clinical isolates from our French database (1969–2019). Minimum spanning trees (MST) were reconstructed with both cgMLST data and cgSNP analyses on these 39 isolates (Fig. 3).

cgMLST-based MST allows to determine the genetic distance in terms of allelic distance. Among the 39 studied isolates, 37 cgMLST profiles were identified. Only 3 isolates belonged to the same cgMLST profile, and they corresponded to specimens from the 2020 Corsica outbreak (Fig. 3A). Pairwise allelic distances between the 39 isolates (table S3) confirm that 3 isolates have the same cgMLST profile (IP43304, IP43952 and IP44154) whereas other isolates have allelic distances between 1 and 157 (table S3).

The cgSNP-based MST (Fig. 3B) allows determination of the genetic distance in terms of point mutations (SNPs). On this tree, we can observe 31 different SNP profiles: 2 isolates from 2005 have the same SNP profile and 8 isolates (IP43304, IP43951, IP43952, IP43989, IP44094, IP44110, IP44154 and IP44180) from the 2020 outbreak have the same profile. Pairwise SNP comparisons (Table S3) confirm the null distance between those isolates, whereas other isolates have between 1 and 802 SNP distances.

This lower number of profiles obtained with the cgSNP-based MST indicates that some of the isolates with different cgMLST profiles have merged into a single cgSNP profile. Simpson’s index of diversity estimation for cgMLST is 0.996 (SD = 0.004), whereas for cgSNP analysis is 0.96 (SD = 0.023). Using unpaired t test with Welch’s correction, comparison of Simpson’s indexes of diversity led to a significant *P*-value < 0.0001. Our cgMLST proved to have a better discriminatory power than cgSNP.

## DISCUSSION

We report here for the first time a *Yptb* clonal outbreak in France, with 8 cases identified during the summer 2020. All cases had been exposed in Corsica, seven of them were exposed in a 10 km radius area in Northeast Corsica. Consumption of local tomatoes was the suspected source of infection. A new cgMLST confirmed that the 8 cases belonged to the same cluster. Moreover, two earlier cases (February and March 2020, both detected in Corsica) were also identified as belonging to the same cluster (although they could not be interviewed on their exposures and no common exposure with the summer cases could be identified).

The number of cases reported during the outbreak is low. However, incidence of pseudotuberculosis is also low in France, with an average number of 11 isolates per year (Fig. S1). This incidence is probably underestimated: all symptomatic patients do not visit their doctor and they rarely prescribe stool examinations in case of diarrhea with no complications. In addition, notification of *Yptb* or transmission of the isolates to the YNRL are not mandatory. Furthermore, detection and isolation of *Yptb* in medical laboratories is difficult: a lower growth rate compared to other enterobacteria, and the presence of competitive microbiota renders *Yersinia* spp. Isolation complex (16, 17). Growth of some *Yptb* strains is impaired on selective CIN agar (18) and precisely this medium is used in all the French medical laboratories to recover *Yersinia* isolates from stool samples. The recent implementation of panel-based testing systems (i.e., multiplex PCR) targeting enterobacteria could alleviate this issue, leading to stool culture only when a PCR positive signal is obtained (19, 20). However, some PCR kits target only Y. enterocolitica specific chromosomal genes, reinforcing the low identification rate of *Yptb*.

Epidemiological investigation pointed tomatoes as the suspected source of infection source for the summer cases. However, as no sampling of tomatoes was performed, this suspected source could not be confirmed. Contaminated vegetables were also suspected in previously reported *Yptb* outbreaks, with iceberg lettuce in 1998 (9) and carrots in 2006 (10) in Finland were confirmed as sources of contamination. As wildlife is considered the *Yptb* reservoir, feces of carrier animals may contaminate environmental water, soil, and grass (21). Contamination of vegetables in the fields can be direct (feces) or indirect (irrigation with contaminated water). Wild boars and pigs are recognized as reservoirs of *Yptb* (22): as Corsica hosts a large population of wild boars and allows the wandering of pigs, it is possible to hypothesize a contamination of vegetables in the fields from this reservoir. Wild rodents may also contaminate vegetables in the fields or during storage. Carrier animals may contaminate their environment as long as they host the pathogen, possibly leading to several episodes or sources of contamination (23).

An increase in *Yptb* cases had been observed and investigated in 2005 in France (Fig. S1 and 1). However, the epidemiological investigation identified a high genetic diversity in the isolates as well as the absence of a geographically defined cluster. This increase in clinical cases has been linked to an increase in prevalence in rodent reservoirs (3).

Identification of outbreak-related isolates and trace-back investigations to identify a potential source of contamination were difficult when techniques such as PFGE (9) or MLVA (12) were used. Depending on the pathogen, the discriminatory power of PFGE may differ and not be optimal (24–26) and its lack of reproducibility between laboratories restricts its use to retrospective epidemiology (27, 28). PFGE has often been replaced by MLVA, which has proven to display a better discriminatory power (26, 29) but is still labor-intensive and time-consuming. Development of whole-genome-based typing methods alleviates these issues and allows rapid detection of clusters as well as near-real-time alerts of public health authorities. The confirmation of genetic relatedness of clinical and food samples remains a strong lever for recalling food products from the market. Rapid trace-back investigation strengthens the possibility to identify a common source of contamination and to remove it from the food chain (30).

Different cgMLST schemes have been developed for foodborne pathogen identification. They have proven to be useful in public health surveillance and have provided tools allowing international collaboration (13, 31, 32). Discriminatory power comparisons between cgMLST and cgSNP analysis have shown a very high discriminatory power for both methods, thus arguing for the use of whole-genome-based methods for epidemiological investigation (Fig. 3) (13). The comparison confirmed the better performance and resolution power of our novel cgMLST specific to *Yptb*. cgSNP analysis has already been used in the investigation of an outbreak due to *Yptb* infection. This tool allowed the identification of a point-source contamination in the food chain (8). However, it requires advanced bioinformatic skills not widely available in National Reference laboratories worldwide. In this framework, we developed a new cgMLST for *Yptb* that proved to be more discriminant than cgSNP analysis (Fig. 3 and Table S2). Here, allelic distance identification was very useful as it confirms that the 8 summer isolates belong to the same cluster with 0 to 3 alleles difference and suggests a persistent or recurrent contamination of the food chain as 2 isolates were identified in February and March 2020. Interestingly, lineage 16 specimens are absent in previous years samplings (Fig. 3A) suggesting that this clone emerged recently.

Our new cgMLST does not require additional laboratory manipulation and is usable in real-time after the identification of the bacterial species, as it only requires the genome assembly of the isolate. Furthermore, it relies on the simple comparison of allelic profiles and should help future international collaboration to determine whether a clone is circulating in several countries. It is available to the scientific community (https://bigsdb.pasteur.fr/yersinia/) and is intended mainly for molecular typing purposes in public health laboratories (as National Reference laboratories).

We report, for the first time in France, an outbreak of *Yptb* infections due to the same clone. Epidemiological and microbiological investigations established a link between the patients and identified the consumption of tomatoes from a unique greengrocery store as the suspected source of infection. Our recently developed cgMLST exhibits an excellent discriminatory power and allows epidemiological investigation in real-time.

## MATERIALS AND METHODS

### Y. pseudotuberculosis isolates and taxonomic assignment.

*Yersinia* isolates, together with some clinical and demographic data, are regularly sent to the French YNRL for enteric yersiniosis by medical laboratories for complete characterization. Isolation and taxonomic assignments are performed as described by Savin et al. (14) based on a 500-gene cgMLST scheme designed to identify all the species of the *Yersinia* genus, as well as the lineage. A total of 359 French isolates of *Yptb* received at the YNRL between 1991 and 2020 for complete characterization were genotypically assigned. In addition, genomic data of 9 isolates of *Yptb* lineage 16 with a clinical origin (1969 to 1990) were extracted from our database for comparison of their genetic relatedness.

### A novel cgMLST as a tool for identification of Y. pseudotuberculosis clusters.

From our 1,346 *Yersinia* reference genomes data set, constituted for the phylogenetic analysis of the *Yersinia* genus (14), 485 genomes (including 385 French isolates received by the YNRL, 66 isolates from abroad in our collection as well as 34 isolates with publicly available genomes) were selected based on the following criteria: (i) we selected genomes sequences generated in our laboratory with a coverage higher than 50×, and (ii) we selected genomes displaying a size between 4 and 5.5 Mb, less than 500 contigs and *N*_50_ value above 10,000 nucleotides. Then, a selection of 100 genomes representative of the whole data set diversity was performed as previously described (14). Core genes definition and selection of the genes was also performed as described in (14) for the whole *Yersinia* genus. In brief, a total of 2,351 genes present in more than 95% of these representative genomes were selected as core genes of the *Ypstb* species. For each of these core genes, a file containing all alleles was generated. Each file was parsed, and genes were removed if (i) a character other than A, T, G or C was present in any of the sequences, (ii) the gene had a paralog, or (iii) there was a gap of more than 6 nucleotides in the multiple sequence alignment. We also removed genes that belong to the 500 genes of the *Yersinia* spp cgMLST scheme (14). These filtering criteria led to the selection of 1,421 core genes specific to *Yptb*, to which the 500 genes of the *Yersinia* spp cgMLST scheme were added, resulting in 1,921 core genes deemed suitable for cgMLST analysis and molecular investigation (Table S1).

A database was created for *Yptb* in the Institut Pasteur’s MLST and whole-genome MLST resource (https://bigsdb.pasteur.fr), which uses the BIGSdb software tool (33, 34). All *de novo* assembled genomes were uploaded into the isolates database, and the reference alleles of the 1,921 cgMLST loci were defined in the linked database of reference sequences and profiles (“seqdef”). Within BIGSdb, a scan of the genome sequence was performed for each isolate using parameters (min 80 % identity, min 80 % alignment, blastn word size of 20 nt) to check for the presence of each core gene and to determine its allele number. The BIGSdb–*Yptb* database of cgMLST profiles is accessible at https://bigsdb.pasteur.fr/yersinia/. A comparison of the allelic profiles can be performed either with the ‘Genome comparator’ plugin or by the construction of a minimum spanning tree (MST) with GrapeTree (35) using the corresponding BIGSdb plugin.

Since 2018 at the YNRL, after the identification of a *Yptb* isolate using the 500-gene *Yersinia* spp cgMLST scheme (14), each isolate is also submitted to this new 1921-gene *Ypstb* cgMLST scheme to evaluate its genetic distance with other isolates from the database. When a cluster of isolates (≤5 allelic difference) is identified, the YNRL alerts SpF who determines whether an epidemiological investigation is required.

### Core-genome SNP analysis.

Genome sequences of all the isolates and paired-end quality-filtered FASTQ files were obtained as described by Savin et al. (14). Variant calling was performed using the IP32953 reference strain (accession number: NC_006155) with Snippy version 4.6.0 and core-SNPs were extracted using snp-sites (https://github.com/tseemann/snippy). A comparison of the isolates using the core-SNPs was performed by the construction of an MST with GrapeTree (35).

### Discriminatory power determination.

The discriminatory power of the molecular typing method was determined using the Simpson’s index of diversity (ID). It calculates the probability of a technique to attribute the same profile to epidemiologically unrelated isolates. The higher the index is, the better the discriminatory power is (36). The standard deviation was estimated as proposed by Grundman et al. (37). The statistical comparison of Simpson’s indexes of diversity was performed using GraphPad Prism 9.3.1 (GraphPad Software, USA).

### Epidemiological, trace-back, and environmental investigations.

The YNRL alerts SpF of any unusual signal of *Yptb* from the microbiological surveillance, including clusters, to determine whether an epidemiological investigation is required. For this outbreak, all patients corresponding to the outbreak case definition were contacted by SpF and queried about their previous exposure to animals, visits to natural areas (sea, lake, forest, river, farms), drinking water supply and food consumption (dairy products, meats, fresh vegetables), using a standard trawling questionnaire. The questionnaire covered the 10 days before the onset of the symptoms. Places of travel (holiday period) were also recorded. Historical records of tap water quality were verified by the regional Health Agency (ARS de Corse) and trace-back investigation of suspected foods were performed by the French Directorate General for Food (DGAl) and the General Directorate for Competition Policy, Consumer Affairs and Fraud Control (DGCCRF).

### Data availability.

The genomes and the allelic profiles of all the investigated isolates are publicly available at the following URL: https://bigsdb.pasteur.fr/yersinia/.
