# Supplementary material for: First Description of a Yersinia pseudotuberculosis Clonal Outbreak in France, Confirmed Using a New Core Genome Multilocus Sequence Typing Method
Source: Microbiol Spectr. 2022 Jul 6;10(4):e01145-22. doi: 10.1128/spectrum.01145-22 (PMC9431522; doi:10.1128/spectrum.01145-22)

Supplemental Material

First description of a *Yersinia pseudotuberculosis* clonal outbreak in France, confirmed using a new core genome multilocus sequence typing method

Table S1: List of the 1,921 genes used for this cgMLST.

Table S2: Allelic profiles of the 35 isolates from 2020 in France.

Table S3: Pairwise distance matrix cgMLST-based and SNP-based obtained comparing the 39 *Y. pseudotuberculosis* isolates belonging to the lineage 16.

Figure S1: Repartition of the different *Y. pseudotuberculosis* lineages in France according to the year of isolation.

Figure S2: Timeline of the lineage 16 isolates during summer 2020. Number between brackets correspond to the isolation month.

Table S1: List of the 1,921 genes used for this cgMLST.

BIGSdb locus	gene	synonym	locus tag	old locus tag	alternative locus tag	product	protein id	Comment
yeps_YPTB0001	mioC		YPTB_RS00075	YPTB0001		FMN-binding protein MioC	WP_002212258.1	
yeps_YPTB0004	viaA		YPTB_RS00090	YPTB0004		ATPase RavA stimulator ViaA	WP_002212255.1	
yeps_YPTB0006	kup	trkD	YPTB_RS00100	YPTB0006		low affinity potassium transporter Kup	WP_011191440.1	
yeps_YPTB0007	rbsD		YPTB_RS00105	YPTB0007		D-ribose pyranase	WP_002212252.1	
yeps_YPTB0008	rbsK		YPTB_RS00110	YPTB0008		ribokinase	WP_011191441.1	
yeps_YPTB0009			YPTB_RS00115	YPTB0009		hypothetical protein	WP_002215906.1	
yeps_YPTB0020	yihI		YPTB_RS00195	YPTB0020		Der GTPase-activating protein YihI	WP_002213158.1	
yeps_YPTB0021	hemN		YPTB_RS00200	YPTB0021		oxygen-independent coproporphyrinogen III oxidase	WP_002213155.1	
yeps_YPTB0023	glnL		YPTB_RS00210	YPTB0023		nitrogen regulation protein NR(II)	WP_002213151.1	
yeps_YPTB0025	typA		YPTB_RS00225	YPTB0025		ribosome-dependent GTPase TypA	WP_002217380.1	
yeps_YPTB0026	yihX		YPTB_RS00230	YPTB0026		glucose-1-phosphatase	WP_002209011.1	
yeps_YPTB0027			YPTB_RS00235	YPTB0027		virulence factor BrkB family protein	WP_011191445.1	
yeps_YPTB0029	fabY		YPTB_RS00245	YPTB0029		fatty acid biosynthesis protein FabY	WP_002209008.1	
yeps_YPTB0030			YPTB_RS00250	YPTB0030		hypothetical protein	WP_002209007.1	
yeps_YPTB0031			YPTB_RS00255	YPTB0031		uracil-xanthine permease family protein	WP_011191446.1	
yeps_YPTB0034	trmH	spoU	YPTB_RS00270	YPTB0034		tRNA (guanosine(18)-2'-O)-methyltransferase TrmH	WP_002209003.1	
yeps_YPTB0035	spoT		YPTB_RS00275	YPTB0035		bifunctional GTP diphosphokinase/guanosine-3',5'-bis pyrophosphate 3'-pyrophosphohydrolase	WP_002209002.1	
yeps_YPTB0036	rpoZ		YPTB_RS00280	YPTB0036		DNA-directed RNA polymerase subunit omega	WP_004392061.1	
yeps_YPTB0038	ligB		YPTB_RS00290	YPTB0038		NAD-dependent DNA ligase LigB	WP_011191447.1	
yeps_YPTB0041	rph		YPTB_RS00305	YPTB0041		ribonuclease PH	WP_002208997.1	
yeps_YPTB0042	pyrE		YPTB_RS00310	YPTB0042		orotate phosphoribosyltransferase	WP_002208996.1	
yeps_YPTB0045	coaBC		YPTB_RS00325	YPTB0045		bifunctional phosphopantothenoylcysteine decarboxylase/phosphopantothenate--cysteine ligase CoaBC	WP_002208993.1	
yeps_YPTB0046	radC		YPTB_RS00330	YPTB0046		DNA repair protein RadC	WP_002208992.1	
yeps_YPTB0050	coaD		YPTB_RS00350	YPTB0050		pantetheine-phosphate adenylyltransferase	WP_011191449.1	
yeps_YPTB0051			YPTB_RS00355	YPTB0051		glycosyltransferase family 2 protein	WP_002208987.1	
yeps_YPTB0054	rfaF	waaF	YPTB_RS00370	YPTB0054		ADP-heptose--LPS heptosyltransferase RfaF	WP_011191450.1	
yeps_YPTB0056			YPTB_RS00380	YPTB0056		glycine C-acetyltransferase	WP_002208982.1	
yeps_YPTB0057	tdh		YPTB_RS00385	YPTB0057		L-threonine 3-dehydrogenase	WP_011191451.1	
yeps_YPTB0058			YPTB_RS00390	YPTB0058		divergent polysaccharide deacetylase family protein	WP_011191452.1	
yeps_YPTB0059	envC		YPTB_RS00395	YPTB0059		murein hydrolase activator EnvC	WP_002208980.1	
yeps_YPTB0060	gpmM		YPTB_RS00400	YPTB0060		2,3-bisphosphoglycerate-independent phosphoglycerate mutase	WP_011191453.1	
yeps_YPTB0061			YPTB_RS00405	YPTB0061		rhodanese-like domain-containing protein	WP_002208978.1	
yeps_YPTB0062	grxC		YPTB_RS00410	YPTB0062		glutaredoxin 3	WP_002208977.1	
yeps_YPTB0063	secB	prlG	YPTB_RS00415	YPTB0063		protein-export chaperone SecB	WP_002208976.1	
yeps_YPTB0066	cysE		YPTB_RS00430	YPTB0066		serine O-acetyltransferase	WP_002208974.1	
yeps_YPTB0067	trmL		YPTB_RS00435	YPTB0067		tRNA (uridine(34)/cytosine(34)/5-carboxymethylaminomethyluridine(34)-2'-O)-methyltransferase TrmL	WP_011191455.1	
yeps_YPTB0068	ada		YPTB_RS00440	YPTB0068		bifunctional DNA-binding transcriptional regulator/O6-methylguanine-DNA methyltransferase Ada	WP_011191456.1	
yeps_YPTB0069	cpxA		YPTB_RS00445	YPTB0069		envelope stress sensor histidine kinase CpxA	WP_002208971.1	
yeps_YPTB0071	cpxP		YPTB_RS00455	YPTB0071		cell-envelope stress modulator CpxP	WP_011191457.1	
yeps_YPTB0073	fieF		YPTB_RS00465	YPTB0073		CDF family cation-efflux transporter FieF	WP_002208967.1	
yeps_YPTB0075			YPTB_RS00475	YPTB0075		sulfate ABC transporter substrate-binding protein	WP_011191459.1	
yeps_YPTB0076			YPTB_RS00480	YPTB0076		molybdate ABC transporter substrate-binding protein	WP_011191460.1	
yeps_YPTB0078			YPTB_RS00490	YPTB0078		4-carboxy-4-hydroxy-2-oxoadipate aldolase/oxaloacetate decarboxylase	WP_002208962.1	
yeps_YPTB0079			YPTB_RS00495	YPTB0079		PIG-L family deacetylase	WP_002208961.1	
yeps_YPTB0081	tpiA	tpi	YPTB_RS00505	YPTB0081		triose-phosphate isomerase	WP_002208959.1	
yeps_YPTB0083			YPTB_RS00515	YPTB0083		DUF805 domain-containing protein	WP_002208957.1	
yeps_YPTB0084	fpr	mvrA	YPTB_RS00520	YPTB0084		ferredoxin--NADP(+) reductase	WP_002208956.1	
yeps_YPTB0085	glpX		YPTB_RS00525	YPTB0085		class II fructose-bisphosphatase	WP_002223912.1	
yeps_YPTB0087			YPTB_RS00535	YPTB0087		aquaporin family protein	WP_002208954.1	
yeps_YPTB0088				YPTB0088		hypothetical protein	CAH19328.1	
yeps_YPTB0090			YPTB_RS00550	YPTB0090		peptidase domain-containing ABC transporter	WP_011191463.1	
yeps_YPTB0091			YPTB_RS00555	YPTB0091		HlyD family secretion protein	WP_002208949.1	
yeps_YPTB0095	rraA		YPTB_RS00575	YPTB0095		ribonuclease E activity regulator RraA	WP_002208945.1	
yeps_YPTB0096			YPTB_RS00580	YPTB0096		1,4-dihydroxy-2-naphthoate polyprenyltransferase	WP_011191464.1	
yeps_YPTB0098	hslV		YPTB_RS00590	YPTB0098		ATP-dependent protease subunit HslV	WP_002208942.1	
yeps_YPTB0100	cytR		YPTB_RS00600	YPTB0100		DNA-binding transcriptional regulator CytR	WP_002216730.1	
yeps_YPTB0102	rpmE		YPTB_RS00615	YPTB0102		50S ribosomal protein L31	WP_002216737.1	
yeps_YPTB0106			YPTB_RS00640	YPTB0106		bifunctional aspartate kinase/homoserine dehydrogenase II	WP_002208934.1	
yeps_YPTB0115			YPTB_RS00695	YPTB0115		type I secretion system permease/ATPase	WP_011191472.1	
yeps_YPTB0119			YPTB_RS00715	YPTB0119		glutathione peroxidase	WP_002209479.1	
yeps_YPTB0120	oxyR		YPTB_RS00720	YPTB0120		DNA-binding transcriptional regulator OxyR	WP_011191476.1	
yeps_YPTB0122	fabR		YPTB_RS00730	YPTB0122		HTH-type transcriptional repressor FabR	WP_002209476.1	
yeps_YPTB0123			YPTB_RS00735	YPTB0123		YijD family membrane protein	WP_002209475.1	
yeps_YPTB0124	trmA		YPTB_RS00740	YPTB0124		tRNA (uridine(54)-C5)-methyltransferase TrmA	WP_002209474.1	
yeps_YPTB0128			YPTB_RS00800	YPTB0128		sugar ABC transporter ATP-binding protein	WP_011191480.1	
yeps_YPTB0129			YPTB_RS00805	YPTB0129		ABC transporter permease	WP_002220992.1	
yeps_YPTB0130	yjfF		YPTB_RS00810	YPTB0130		sugar ABC transporter permease YjfF	WP_002212021.1	
yeps_YPTB0131	hdfR		YPTB_RS00815	YPTB0131		HTH-type transcriptional regulator HdfR	WP_002212020.1	
yeps_YPTB0135	ilvM		YPTB_RS00835	YPTB0135		acetolactate synthase 2 small subunit	WP_002212016.1	
yeps_YPTB0136			YPTB_RS00840	YPTB0136		branched-chain amino acid transaminase	WP_002212015.1	
yeps_YPTB0144	ilvY		YPTB_RS00880	YPTB0144		HTH-type transcriptional activator IlvY	WP_002212008.1	
yeps_YPTB0163	rep		YPTB_RS00995	YPTB0163		DNA helicase Rep	WP_002211993.1	
yeps_YPTB0165	rhlB	mmrA	YPTB_RS01005	YPTB0165		ATP-dependent RNA helicase RhlB	WP_002228177.1	
yeps_YPTB0168	wecA		YPTB_RS01020	YPTB0168		UDP-N-acetylglucosamine--undecaprenyl-phosphate N-acetylglucosaminephosphotransferase	WP_002211988.1	
yeps_YPTB0169	wzzE	wzz	YPTB_RS01025	YPTB0169		ECA polysaccharide chain length modulation protein	WP_012304740.1	
yeps_YPTB0172	rffG		YPTB_RS01040	YPTB0172		dTDP-glucose 4,6-dehydratase	WP_172601537.1	
yeps_YPTB0174	rffC	wecD	YPTB_RS01050	YPTB0174		dTDP-4-amino-4,6-dideoxy-D-galactose acyltransferase	WP_012304738.1	
yeps_YPTB0175	rffA	wecE	YPTB_RS01055	YPTB0175		dTDP-4-amino-4,6-dideoxygalactose transaminase	WP_002211981.1	
yeps_YPTB0176	wzxE	wzx	YPTB_RS01060	YPTB0176		lipid III flippase WzxE	WP_011191502.1	
yeps_YPTB0178	wzyE		YPTB_RS01070	YPTB0178		ECA oligosaccharide polymerase	WP_002211978.1	
yeps_YPTB0183	hemD		YPTB_RS01115	YPTB0183		uroporphyrinogen-III synthase	WP_011191507.1	
yeps_YPTB0191			YPTB_RS01160	YPTB0191		DUF484 domain-containing protein	WP_002211472.1	
yeps_YPTB0192	xerC		YPTB_RS01165	YPTB0192		tyrosine recombinase XerC	WP_011191512.1	
yeps_YPTB0194	uvrD	mutU,pdeB,rad,recL	YPTB_RS01175	YPTB0194		DNA helicase II	WP_002211475.1	
yeps_YPTB0198	corA		YPTB_RS01195	YPTB0198		magnesium/cobalt transporter CorA	WP_002211480.1	
yeps_YPTB0200			YPTB_RS01205	YPTB0200		thioesterase family protein	WP_002211482.1	
yeps_YPTB0201	pldA		YPTB_RS01210	YPTB0201		phospholipase A	WP_002211483.1	
yeps_YPTB0202	recQ		YPTB_RS01215	YPTB0202		ATP-dependent DNA helicase RecQ	WP_002211484.1	
yeps_YPTB0203	rhtC		YPTB_RS01220	YPTB0203		threonine export protein RhtC	WP_002211485.1	
yeps_YPTB0205	pldB		YPTB_RS01230	YPTB0205		lysophospholipase L2	WP_002211487.1	
yeps_YPTB0208	glpQ		YPTB_RS01245	YPTB0208		glycerophosphodiester phosphodiesterase	WP_002211490.1	
yeps_YPTB0209	glpA		YPTB_RS01255	YPTB0209		anaerobic glycerol-3-phosphate dehydrogenase subunit A	WP_002211491.1	
yeps_YPTB0210	glpB		YPTB_RS01260	YPTB0210		glycerol-3-phosphate dehydrogenase subunit GlpB	WP_011191516.1	
yeps_YPTB0212			YPTB_RS01270	YPTB0212		DcrB family lipoprotein	WP_002211494.1	
yeps_YPTB0213			YPTB_RS01275	YPTB0213		7-cyano-7-deazaguanine/7-aminomethyl-7-deazaguanine transporter	WP_011191518.1	
yeps_YPTB0214	tusA		YPTB_RS01280	YPTB0214		sulfurtransferase TusA	WP_002215973.1	
yeps_YPTB0217			YPTB_RS01295	YPTB0217		DUF1820 family protein	WP_002211499.1	
yeps_YPTB0218			YPTB_RS01300	YPTB0218		DUF2500 domain-containing protein	WP_002211500.1	
yeps_YPTB0220	rsmD		YPTB_RS01310	YPTB0220		16S rRNA (guanine(966)-N(2))-methyltransferase	WP_011191520.1	
yeps_YPTB0223	ftsX	ftsS	YPTB_RS01330	YPTB0223		permease-like cell division protein FtsX	WP_002211505.1	
yeps_YPTB0225	panM		YPTB_RS01340	YPTB0225		aspartate 1-decarboxylase autocleavage activator PanM	WP_011191522.1	
yeps_YPTB0226			YPTB_RS01345	YPTB0226		branched-chain amino acid ABC transporter substrate-binding protein	WP_011191523.1	
yeps_YPTB0230	livF		YPTB_RS01365	YPTB0230		high-affinity branched-chain amino acid ABC transporter ATP-binding protein LivF	WP_002211512.1	
yeps_YPTB0235			YPTB_RS01390	YPTB0235		fimbria/pilus periplasmic chaperone	WP_011191529.1	
yeps_YPTB0242	ugpQ		YPTB_RS01425	YPTB0242		glycerophosphodiester phosphodiesterase	WP_011191534.1	
yeps_YPTB0243			YPTB_RS01430	YPTB0243		AEC family transporter	WP_002224020.1	
yeps_YPTB0246			YPTB_RS01445	YPTB0246		carboxylate/amino acid/amine transporter	WP_002215597.1	
yeps_YPTB0248	metE		YPTB_RS01455	YPTB0248		5-methyltetrahydropteroyltriglutamate--homocysteine S-methyltransferase	WP_011191538.1	
yeps_YPTB0249			YPTB_RS01460	YPTB0249		dienelactone hydrolase family protein	WP_012413425.1	
yeps_YPTB0254	rmuC		YPTB_RS01485	YPTB0254		DNA recombination protein RmuC	WP_002211532.1	
yeps_YPTB0255	ubiE		YPTB_RS01490	YPTB0255		bifunctional demethylmenaquinone methyltransferase/2-methoxy-6-polyprenyl-1,4-benzoquinol methylase UbiE	WP_002224024.1	
yeps_YPTB0256			YPTB_RS01495	YPTB0256		SCP2 domain-containing protein	WP_002215928.1	
yeps_YPTB0257	ubiB		YPTB_RS01500	YPTB0257		ubiquinone biosynthesis regulatory protein kinase UbiB	WP_002211535.1	
yeps_YPTB0258			YPTB_RS01505	YPTB0258		Sec-independent protein translocase subunit TatA	WP_002211536.1	
yeps_YPTB0259	tatB	mtta2	YPTB_RS01510	YPTB0259		Sec-independent protein translocase protein TatB	WP_011191542.1	
yeps_YPTB0260	tatC	mttB	YPTB_RS01515	YPTB0260		Sec-independent protein translocase subunit TatC	WP_011191543.1	
yeps_YPTB0268	pepQ		YPTB_RS01555	YPTB0268		Xaa-Pro dipeptidase	WP_011191548.1	
yeps_YPTB0269			YPTB_RS01560	YPTB0269		IMPACT family protein	WP_002231137.1	
yeps_YPTB0273	birA	bioR,dhbB	YPTB_RS01610	YPTB0273		bifunctional biotin--[acetyl-CoA-carboxylase] ligase/biotin operon repressor BirA	WP_011191550.1	
yeps_YPTB0274	coaA	panK,rts	YPTB_RS01615	YPTB0274		type I pantothenate kinase	WP_002212290.1	
yeps_YPTB0276	tuf		YPTB_RS01645	YPTB0276		elongation factor Tu	WP_002210669.1	
yeps_YPTB0281	rplJ		YPTB_RS01670	YPTB0281		50S ribosomal protein L10	WP_002210674.1	
yeps_YPTB0283	rpoB		YPTB_RS01680	YPTB0283		DNA-directed RNA polymerase subunit beta	WP_002210676.1	
yeps_YPTB0286			YPTB_RS01700	YPTB0286		thiazole synthase	WP_002228257.1	
yeps_YPTB0287	thiS		YPTB_RS01705	YPTB0287		sulfur carrier protein ThiS	WP_002217275.1	
yeps_YPTB0288			YPTB_RS01710	YPTB0288		HesA/MoeB/ThiF family protein	WP_002210680.1	
yeps_YPTB0289	thiE		YPTB_RS01715	YPTB0289		thiamine phosphate synthase	WP_012303440.1	
yeps_YPTB0291	rsd		YPTB_RS01730	YPTB0291		sigma D regulator	WP_011191553.1	
yeps_YPTB0292	nudC		YPTB_RS01735	YPTB0292		NAD(+) diphosphatase	WP_011191554.1	
yeps_YPTB0294	hemE		YPTB_RS01745	YPTB0294		uroporphyrinogen decarboxylase	WP_011191555.1	
yeps_YPTB0297	hupA		YPTB_RS01760	YPTB0297		DNA-binding protein HU-alpha	WP_002210689.1	
yeps_YPTB0299	purD		YPTB_RS01770	YPTB0299		phosphoribosylamine--glycine ligase	WP_011191558.1	
yeps_YPTB0310			YPTB_RS01850	YPTB0310		two component system response regulator	WP_011191565.1	
yeps_YPTB0311			YPTB_RS01855	YPTB0311		two component system sensor kinase	WP_011191566.1	
yeps_YPTB0315			YPTB_RS01875	YPTB0315		AraC family transcriptional regulator	WP_011171760.1	
yeps_YPTB0316	sctF		YPTB_RS01880	YPTB0316		type III secretion system needle filament subunit SctF	WP_011191571.1	
yeps_YPTB0317			YPTB_RS01885	YPTB0317		EscG/YscG/SsaH family type III secretion system needle protein co-chaperone	WP_002209040.1	
yeps_YPTB0318	sctI		YPTB_RS01890	YPTB0318		type III secretion system inner rod subunit SctI	WP_002209041.1	
yeps_YPTB0319	ssaJ		YPTB_RS01895	YPTB0319		EscJ/YscJ/HrcJ family type III secretion inner membrane ring protein SsaJ	WP_011191572.1	
yeps_YPTB0321	sctL		YPTB_RS01905	YPTB0321		type III secretion system stator protein SctL	WP_011191573.1	
yeps_YPTB0325				YPTB0325		conserved hypothetical protein	CAH19565.1	
yeps_YPTB0326			YPTB_RS01930	YPTB0326		YscQ/HrcQ family type III secretion apparatus protein	WP_012413433.1	
yeps_YPTB0328			YPTB_RS01940	YPTB0328		EscS/YscS/HrcS family type III secretion system export apparatus protein	WP_002209051.1	
yeps_YPTB0335	metC		YPTB_RS01975	YPTB0335		cystathionine beta-lyase	WP_002215044.1	
yeps_YPTB0336			YPTB_RS01980	YPTB0336		heme ABC transporter ATP-binding protein	WP_002209058.1	
yeps_YPTB0338			YPTB_RS01990	YPTB0338		hemin ABC transporter substrate-binding protein	WP_011191579.1	
yeps_YPTB0339			YPTB_RS01995	YPTB0339		hemin-degrading factor	WP_002209061.1	
yeps_YPTB0340			YPTB_RS02000	YPTB0340		TonB-dependent hemoglobin/transferrin/lactoferrin family receptor	WP_011191580.1	

yeps_YPTB0341			YPTB_RS23515	YPTB0341		hemin uptake protein HemP	WP_011191581.1	
yeps_YPTB0342			YPTB_RS02010	YPTB0342		SDR family oxidoreductase	WP_011191582.1	
yeps_YPTB0347			YPTB_RS02035	YPTB0347		citrate lyase subunit beta	WP_002209069.1	
yeps_YPTB0348			YPTB_RS02040	YPTB0348		cysteine protease StiP family protein	WP_002209070.1	
yeps_YPTB0349			YPTB_RS02045	YPTB0349		hypothetical protein	WP_002209071.1	
yeps_YPTB0350			YPTB_RS02050	YPTB0350		phosphoribosyltransferase domain-containing protein	WP_002209072.1	
yeps_YPTB0352			YPTB_RS02060	YPTB0352		tellurium resistance TerZ family protein	WP_011191586.1	
yeps_YPTB0354			YPTB_RS02070	YPTB0354		tellurite resistance TerB family protein	WP_002209076.1	
yeps_YPTB0355			YPTB_RS02075	YPTB0355		TerC/Alx family metal homeostasis membrane protein	WP_011191588.1	
yeps_YPTB0356			YPTB_RS02080	YPTB0356		TerD family protein	WP_002209078.1	
yeps_YPTB0367	ubiA	cyr	YPTB_RS02135	YPTB0367		4-hydroxybenzoate octaprenyltransferase	WP_002209088.1	
yeps_YPTB0369			YPTB_RS02145	YPTB0369		diacylglycerol kinase	WP_002209089.1	
yeps_YPTB0371	zur		YPTB_RS02155	YPTB0371		zinc uptake transcriptional repressor Zur	WP_002214647.1	
yeps_YPTB0378			YPTB_RS02190	YPTB0378		MmcQ/YjbR family DNA-binding protein	WP_002209098.1	
yeps_YPTB0379	uvrA	dinE	YPTB_RS02195	YPTB0379		excinuclease ABC subunit UvrA	WP_011191598.1	
yeps_YPTB0381	rhaM		YPTB_RS02205	YPTB0381		L-rhamnose mutarotase	WP_002209101.1	
yeps_YPTB0382	fucO		YPTB_RS02210	YPTB0382		lactaldehyde reductase	WP_011191599.1	
yeps_YPTB0383	rhaD		YPTB_RS02215	YPTB0383		rhamnulose-1-phosphate aldolase	WP_011191600.1	
yeps_YPTB0384			YPTB_RS02220	YPTB0384		L-rhamnose isomerase	WP_011191601.1	
yeps_YPTB0385	rhaB		YPTB_RS02225	YPTB0385		rhamnulokinase	WP_002209105.1	
yeps_YPTB0386	rhaS	rhaC2	YPTB_RS02240	YPTB0386		HTH-type transcriptional activator RhaS	WP_011191602.1	
yeps_YPTB0387	rhaR	rhaC1	YPTB_RS02245	YPTB0387		HTH-type transcriptional activator RhaR	WP_011191603.1	
yeps_YPTB0391			YPTB_RS02260	YPTB0391		subtilase family AB5 toxin binding subunit	WP_002209112.1	
yeps_YPTB0396	aegA		YPTB_RS02285	YPTB0396		formate-dependent uric acid utilization protein AegA	WP_011191607.1	
yeps_YPTB0397			YPTB_RS02290	YPTB0397		4Fe-4S binding protein	WP_002209119.1	
yeps_YPTB0400	cutA		YPTB_RS02305	YPTB0400		divalent cation tolerance protein CutA	WP_002209122.1	
yeps_YPTB0401			YPTB_RS02310	YPTB0401		anaerobic C4-dicarboxylate transporter	WP_011191610.1	
yeps_YPTB0409	sugE		YPTB_RS02350	YPTB0409		quaternary ammonium compound efflux SMR transporter SugE	WP_002228124.1	
yeps_YPTB0410	frdD		YPTB_RS02355	YPTB0410		fumarate reductase subunit FrdD	WP_002209134.1	
yeps_YPTB0414	epmA		YPTB_RS02380	YPTB0414		elongation factor P--(R)-beta-lysine ligase	WP_002209139.1	
yeps_YPTB0415	mscM		YPTB_RS02385	YPTB0415		miniconductance mechanosensitive channel MscM	WP_032466065.1	
yeps_YPTB0416	psd		YPTB_RS02390	YPTB0416		archaetidylserine decarboxylase	WP_002209141.1	
yeps_YPTB0417	rsgA		YPTB_RS02395	YPTB0417		small ribosomal subunit biogenesis GTPase RsgA	WP_002209142.1	
yeps_YPTB0420	nnr		YPTB_RS02435	YPTB0420		bifunctional ADP-dependent NAD(P)H-hydrate dehydratase/NAD(P)H-hydrate epimerase	WP_011191613.1	
yeps_YPTB0421	tsaE		YPTB_RS02440	YPTB0421		tRNA (adenosine(37)-N6)-threonylcarbamoyltransferase complex ATPase subunit type 1 TsaE	WP_011191614.1	
yeps_YPTB0426	hflX		YPTB_RS02465	YPTB0426		GTPase HflX	WP_002209152.1	
yeps_YPTB0428	hflC	hflA	YPTB_RS02475	YPTB0428		protease modulator HflC	WP_002209155.1	
yeps_YPTB0430			YPTB_RS02485	YPTB0430		adenylosuccinate synthase	WP_002209157.1	
yeps_YPTB0431	nsrR		YPTB_RS02490	YPTB0431		nitric oxide-sensing transcriptional repressor NsrR	WP_002217229.1	
yeps_YPTB0435	bsmA		YPTB_RS02510	YPTB0435		biofilm peroxide resistance protein BsmA	WP_002215294.1	
yeps_YPTB0436	yjfP		YPTB_RS02515	YPTB0436		esterase	WP_011191618.1	
yeps_YPTB0437				YPTB0437		hypothetical protein	CAH19677.1	
yeps_YPTB0442			YPTB_RS02545	YPTB0442		DUF3757 domain-containing protein	WP_002210157.1	
yeps_YPTB0443			YPTB_RS02550	YPTB0443		OapA family protein	WP_002210158.1	
yeps_YPTB0445	ytfE		YPTB_RS02560	YPTB0445		iron-sulfur cluster repair protein YtfE	WP_011191621.1	
yeps_YPTB0446			YPTB_RS02565	YPTB0446		bifunctional 2',3'-cyclic-nucleotide 2'-phosphodiesterase/3'-nucleotidase	WP_011191622.1	
yeps_YPTB0447	cysQ	amtA	YPTB_RS02570	YPTB0447		3'(2'),5'-bisphosphate nucleotidase CysQ	WP_032466061.1	
yeps_YPTB0448			YPTB_RS02575	YPTB0448		YtfJ family protein	WP_002210162.1	
yeps_YPTB0449			YPTB_RS02580	YPTB0449		DUF1107 domain-containing protein	WP_002210163.1	
yeps_YPTB0451	msrA	pms	YPTB_RS02590	YPTB0451		peptide-methionine (S)-S-oxide reductase MsrA	WP_002210165.1	
yeps_YPTB0452			YPTB_RS02595	YPTB0452		autotransporter assembly complex protein TamA	WP_011191624.1	
yeps_YPTB0454			YPTB_RS02605	YPTB0454		gamma-glutamylcyclotransferase	WP_002210168.1	
yeps_YPTB0455	ppa		YPTB_RS02610	YPTB0455		inorganic diphosphatase	WP_002210169.1	
yeps_YPTB0456	fbp	fdp	YPTB_RS02615	YPTB0456		class 1 fructose-bisphosphatase	WP_011191626.1	
yeps_YPTB0467	cgtA		YPTB_RS02675	YPTB0467		Obg family GTPase CgtA	WP_002210181.1	
yeps_YPTB0468	pmrB		YPTB_RS02680	YPTB0468		two-component system sensor histidine kinase PmrB	WP_011191629.1	
yeps_YPTB0469	pmrA		YPTB_RS02685	YPTB0469		two-component system response regulator PmrA	WP_002210183.1	
yeps_YPTB0474	ftsH	hflB,mrsC,tolZ	YPTB_RS02710	YPTB0474		ATP-dependent zinc metalloprotease FtsH	WP_002228195.1	
yeps_YPTB0477	secG		YPTB_RS02725	YPTB0477		preprotein translocase subunit SecG	WP_002210190.1	
yeps_YPTB0479	nusA		YPTB_RS02745	YPTB0479		transcription termination factor NusA	WP_002209253.1	
yeps_YPTB0482	truB	p35	YPTB_RS02760	YPTB0482		tRNA pseudouridine(55) synthase TruB	WP_002209256.1	
yeps_YPTB0491			YPTB_RS02805	YPTB0491		efflux RND transporter periplasmic adaptor subunit	WP_002209266.1	
yeps_YPTB0493			YPTB_RS02815	YPTB0493		efflux transporter outer membrane subunit	WP_011191634.1	
yeps_YPTB0495			YPTB_RS02825	YPTB0495		U32 family peptidase	WP_011191635.1	
yeps_YPTB0496			YPTB_RS02835	YPTB0496		SCP2 domain-containing protein	WP_002209272.1	
yeps_YPTB0497			YPTB_RS02840	YPTB0497		N-acetyltransferase	WP_011191636.1	
yeps_YPTB0498			YPTB_RS02850	YPTB0498		GIY-YIG nuclease family protein	WP_011191637.1	
yeps_YPTB0499			YPTB_RS02855	YPTB0499		heparinase II/III family protein	WP_011191638.1	
yeps_YPTB0502			YPTB_RS02870	YPTB0502		sugar ABC transporter permease	WP_011191641.1	
yeps_YPTB0503			YPTB_RS02875	YPTB0503		carbohydrate ABC transporter permease	WP_002209279.1	
yeps_YPTB0504	ugpC		YPTB_RS02880	YPTB0504		sn-glycerol-3-phosphate ABC transporter ATP-binding protein UgpC	WP_002209280.1	
yeps_YPTB0505			YPTB_RS02885	YPTB0505		alginate lyase family protein	WP_002209281.1	
yeps_YPTB0507			YPTB_RS02895	YPTB0507		hypothetical protein	WP_002209283.1	
yeps_YPTB0508	phnP		YPTB_RS02900	YPTB0508		phosphonate metabolism protein PhnP	WP_002209284.1	
yeps_YPTB0509	phnN		YPTB_RS02905	YPTB0509		ribose 1,5-bisphosphokinase	WP_002209285.1	
yeps_YPTB0510	phnM		YPTB_RS02910	YPTB0510		alpha-D-ribose 1-methylphosphonate 5-triphosphate diphosphatase	WP_011191643.1	
yeps_YPTB0511	phnL		YPTB_RS02915	YPTB0511		phosphonate C-P lyase system protein PhnL	WP_011191644.1	
yeps_YPTB0512	phnK		YPTB_RS02920	YPTB0512		phosphonate C-P lyase system protein PhnK	WP_011191645.1	
yeps_YPTB0513			YPTB_RS02925	YPTB0513		alpha-D-ribose 1-methylphosphonate 5-phosphate C-P-lyase PhnJ	WP_011191646.1	
yeps_YPTB0516	phnG		YPTB_RS24180	YPTB0516		phosphonate C-P lyase system protein PhnG	WP_002209292.1	
yeps_YPTB0517	phnF		YPTB_RS24185	YPTB0517		phosphonate metabolism transcriptional regulator PhnF	WP_011191649.1	
yeps_YPTB0518	nrdG	yjgE	YPTB_RS02950	YPTB0518		anaerobic ribonucleoside-triphosphate reductase-activating protein	WP_025470743.1	
yeps_YPTB0519	nrdD		YPTB_RS02955	YPTB0519		anaerobic ribonucleoside-triphosphate reductase	WP_002209296.1	
yeps_YPTB0520			YPTB_RS02960	YPTB0520		ABC transporter ATP-binding protein	WP_002209297.1	
yeps_YPTB0521			YPTB_RS02965	YPTB0521		ABC transporter ATP-binding protein	WP_002209298.1	
yeps_YPTB0522			YPTB_RS02970	YPTB0522		ABC transporter permease	WP_002209299.1	
yeps_YPTB0526	argF		YPTB_RS02990	YPTB0526		ornithine carbamoyltransferase	WP_011191651.1	
yeps_YPTB0529			YPTB_RS03005	YPTB0529		valine--tRNA ligase	WP_011191653.1	
yeps_YPTB0533	lptG		YPTB_RS03025	YPTB0533		LPS export ABC transporter permease LptG	WP_002223173.1	
yeps_YPTB0541			YPTB_RS03070	YPTB0541		AraC family transcriptional regulator	WP_011191658.1	
yeps_YPTB0542			YPTB_RS03075	YPTB0542		PTS fructose-like transporter subunit IIB	WP_002209180.1	
yeps_YPTB0546			YPTB_RS03095	YPTB0546		DUF2501 domain-containing protein	WP_002209184.1	
yeps_YPTB0547	lsrG		YPTB_RS03100	YPTB0547		(4S)-4-hydroxy-5-phosphonooxypentane-2,3-dione isomerase	WP_002209186.1	
yeps_YPTB0549	lsrB		YPTB_RS03110	YPTB0549		autoinducer 2 ABC transporter substrate-binding protein LsrB	WP_011191662.1	
yeps_YPTB0550	lsrD		YPTB_RS03115	YPTB0550		autoinducer 2 ABC transporter permease LsrD	WP_011191663.1	
yeps_YPTB0551	lsrC		YPTB_RS03120	YPTB0551		autoinducer 2 ABC transporter permease LsrC	WP_011191664.1	
yeps_YPTB0552	lsrA		YPTB_RS03125	YPTB0552		autoinducer 2 ABC transporter ATP-binding protein LsrA	WP_011191665.1	
yeps_YPTB0554	lsrK		YPTB_RS03140	YPTB0554		autoinducer-2 kinase	WP_002230543.1	
yeps_YPTB0564			YPTB_RS03185	YPTB0564		LapA family protein	WP_002209198.1	
yeps_YPTB0565			YPTB_RS03190	YPTB0565		efflux RND transporter periplasmic adaptor subunit	WP_002209199.1	
yeps_YPTB0575	prfC	miaD,tos	YPTB_RS03260	YPTB0575		peptide chain release factor 3	WP_011191681.1	
yeps_YPTB0581	deoC	dra	YPTB_RS03290	YPTB0581		deoxyribose-phosphate aldolase	WP_002216078.1	
yeps_YPTB0582	deoA	tpp,ttg	YPTB_RS03295	YPTB0582		thymidine phosphorylase	WP_011191687.1	
yeps_YPTB0585			YPTB_RS03310	YPTB0585		YtjB family periplasmic protein	WP_002209218.1	
yeps_YPTB0586	serB		YPTB_RS03315	YPTB0586		phosphoserine phosphatase	WP_002209219.1	
yeps_YPTB0587	radA	sms	YPTB_RS03320	YPTB0587		DNA repair protein RadA	WP_002209220.1	
yeps_YPTB0588	nadR	nadI,pnuA	YPTB_RS03325	YPTB0588		multifunctional transcriptional regulator/nicotinamide-nucleotide adenylyltransferase/ribosylnicotinamide kinase NadR	WP_002209221.1	
yeps_YPTB0590	ettA		YPTB_RS03335	YPTB0590		energy-dependent translational throttle protein EttA	WP_011191691.1	
yeps_YPTB0591			YPTB_RS03340	YPTB0591		OmpA family protein	WP_011191692.1	
yeps_YPTB0593			YPTB_RS03350	YPTB0593		YfiR family protein	WP_002209225.1	
yeps_YPTB0595	sltY		YPTB_RS03360	YPTB0595		murein transglycosylase	WP_011191694.1	
yeps_YPTB0597	yjjX		YPTB_RS03370	YPTB0597		inosine/xanthosine triphosphatase	WP_011191695.1	
yeps_YPTB0600	creA	yjjD	YPTB_RS03385	YPTB0600		protein CreA	WP_002209232.1	
yeps_YPTB0611	dnaK		YPTB_RS03440	YPTB0611		molecular chaperone DnaK	WP_002209248.1	
yeps_YPTB0612	dnaJ	groP,grpC	YPTB_RS03445	YPTB0612		molecular chaperone DnaJ	WP_002209249.1	
yeps_YPTB0613	nhaA	ant,antA	YPTB_RS03450	YPTB0613		Na+/H+ antiporter NhaA	WP_011191700.1	
yeps_YPTB0615	rpsT	sup(s20)	YPTB_RS03460	YPTB0615		30S ribosomal protein S20	WP_002220715.1	
yeps_YPTB0617	ileS	ilvS	YPTB_RS03475	YPTB0617		isoleucine--tRNA ligase	WP_002210509.1	
yeps_YPTB0618	lspA		YPTB_RS03480	YPTB0618		signal peptidase II	WP_002210508.1	
yeps_YPTB0619	fkpB	slpA,yaaD	YPTB_RS03485	YPTB0619		FKBP-type peptidyl-prolyl cis-trans isomerase	WP_002228112.1	
yeps_YPTB0623	carA	arg,cap,pyrA	YPTB_RS03505	YPTB0623		glutamine-hydrolyzing carbamoyl-phosphate synthase small subunit	WP_002224759.1	
yeps_YPTB0624	carB	arg,cap,pyrA	YPTB_RS03510	YPTB0624		carbamoyl-phosphate synthase large subunit	WP_011191702.1	
yeps_YPTB0625			YPTB_RS03515	YPTB0625		LysE family translocator	WP_011191703.1	
yeps_YPTB0626			YPTB_RS03520	YPTB0626		threonine/serine exporter ThrE family protein	WP_011191704.1	
yeps_YPTB0627			YPTB_RS03525	YPTB0627		threonine/serine exporter	WP_032466075.1	
yeps_YPTB0631	apaH	cfcB	YPTB_RS03545	YPTB0631		bis(5'-nucleosyl)-tetraphosphatase (symmetrical) ApaH	WP_011191708.1	
yeps_YPTB0634	pdxA		YPTB_RS03560	YPTB0634		4-hydroxythreonine-4-phosphate dehydrogenase PdxA	WP_011191711.1	
yeps_YPTB0635	surA		YPTB_RS03565	YPTB0635		peptidylprolyl isomerase SurA	WP_002210488.1	
yeps_YPTB0637	djlA	yabH	YPTB_RS03575	YPTB0637		co-chaperone DjlA	WP_011191713.1	
yeps_YPTB0641	tssB		YPTB_RS03595	YPTB0641		type VI secretion system contractile sheath small subunit	WP_002210481.1	
yeps_YPTB0643			YPTB_RS03605	YPTB0643		Hcp family type VI secretion system effector	WP_002210479.1	
yeps_YPTB0644			YPTB_RS03610	YPTB0644		type VI secretion system baseplate subunit TssE	WP_002210478.1	
yeps_YPTB0645	tssF		YPTB_RS03615	YPTB0645		type VI secretion system baseplate subunit TssF	WP_011191714.1	
yeps_YPTB0647	tssH		YPTB_RS03625	YPTB0647		type VI secretion system ATPase TssH	WP_002210475.1	
yeps_YPTB0649			YPTB_RS03635	YPTB0649		pentapeptide repeat-containing protein	WP_011191716.1	
yeps_YPTB0651			YPTB_RS03645	YPTB0651		DUF3540 domain-containing protein	WP_002210473.1	
yeps_YPTB0652			YPTB_RS03650	YPTB0652		DUF4150 domain-containing protein	WP_002214737.1	
yeps_YPTB0653	tssJ		YPTB_RS03655	YPTB0653		type VI secretion system lipoprotein TssJ	WP_011191717.1	
yeps_YPTB0654	tssK		YPTB_RS03660	YPTB0654		type VI secretion system baseplate subunit TssK	WP_002210470.1	
yeps_YPTB0660			YPTB_RS03690	YPTB0660		DedA family protein	WP_002210465.1	
yeps_YPTB0661	thiQ	sfuC,yabJ	YPTB_RS03695	YPTB0661		thiamine ABC transporter ATP-binding protein ThiQ	WP_011191721.1	
yeps_YPTB0662	thiP	sfuB,yabK	YPTB_RS03700	YPTB0662		thiamine/thiamine pyrophosphate ABC transporter permease ThiP	WP_011191722.1	
yeps_YPTB0668			YPTB_RS03735	YPTB0668		sugar efflux transporter	WP_011191726.1	
yeps_YPTB0669	leuD		YPTB_RS03740	YPTB0669		3-isopropylmalate dehydratase small subunit	WP_002210456.1	
yeps_YPTB0670	leuC		YPTB_RS03745	YPTB0670		3-isopropylmalate dehydratase large subunit	WP_011191727.1	
yeps_YPTB0680	rsmH		YPTB_RS03815	YPTB0680		16S rRNA (cytosine(1402)-N(4))-methyltransferase RsmH	WP_002210442.1	
yeps_YPTB0681	ftsL	mraR,yabD	YPTB_RS03820	YPTB0681		cell division protein FtsL	WP_002210441.1	
yeps_YPTB0682			YPTB_RS03825	YPTB0682		peptidoglycan glycosyltransferase FtsI	WP_011191731.1	
yeps_YPTB0683	murE		YPTB_RS03830	YPTB0683		UDP-N-acetylmuramoyl-L-alanyl-D-glutamate--2,6-diaminopimelate ligase	WP_012105673.1	
yeps_YPTB0684	murF	mra	YPTB_RS03835	YPTB0684		UDP-N-acetylmuramoyl-tripeptide--D-alanyl-D-alanine ligase	WP_011191733.1	
yeps_YPTB0686	murD		YPTB_RS03845	YPTB0686		UDP-N-acetylmuramoyl-L-alanine--D-glutamate ligase	WP_011191734.1	

yeps_YPTB0687	ftsW		YPTB_RS03850	YPTB0687		cell division protein FtsW	WP_002210435.1	
yeps_YPTB0688	murG		YPTB_RS03855	YPTB0688		undecaprenyldiphospho-muramoylpentapeptide beta-N-acetylglucosaminyltransferase	WP_011191735.1	
yeps_YPTB0689	murC		YPTB_RS03860	YPTB0689		UDP-N-acetylmuramate--L-alanine ligase	WP_002216457.1	
yeps_YPTB0694	lpxC	asmB,envA	YPTB_RS03885	YPTB0694		UDP-3-O-acyl-N-acetylglucosamine deacetylase	WP_002228285.1	
yeps_YPTB0695			YPTB_RS03890	YPTB0695		DUF721 domain-containing protein	WP_002210428.1	
yeps_YPTB0696	secM		YPTB_RS03895	YPTB0696		secA translation cis-regulator SecM	WP_011191738.1	
yeps_YPTB0706	gspE	pulE	YPTB_RS03955	YPTB0706		type II secretion system protein GspE	WP_011191740.1	
yeps_YPTB0708	nadC		YPTB_RS03965	YPTB0708		carboxylating nicotinate-nucleotide diphosphorylase	WP_002209324.1	
yeps_YPTB0710	ampE		YPTB_RS03975	YPTB0710		beta-lactamase regulator AmpE	WP_002209326.1	
yeps_YPTB0717			YPTB_RS04010	YPTB0717		protein YacL	WP_002209334.1	
yeps_YPTB0718	yddG		YPTB_RS04015	YPTB0718		aromatic amino acid DMT transporter YddG	WP_011191744.1	
yeps_YPTB0720	speE		YPTB_RS04025	YPTB0720		polyamine aminopropyltransferase	WP_011191746.1	
yeps_YPTB0721			YPTB_RS04030	YPTB0721		YacC family pilotin-like protein	WP_002228215.1	
yeps_YPTB0722	cueO		YPTB_RS04035	YPTB0722		multicopper oxidase CueO	WP_011191747.1	
yeps_YPTB0724	can		YPTB_RS04045	YPTB0724		carbonate dehydratase	WP_002209342.1	
yeps_YPTB0725			YPTB_RS04050	YPTB0725		ABC transporter ATP-binding protein	WP_011191749.1	
yeps_YPTB0727			YPTB_RS04060	YPTB0727		polysaccharide deacetylase family protein	WP_012413481.1	
yeps_YPTB0728	panD		YPTB_RS04065	YPTB0728		aspartate 1-decarboxylase	WP_011191751.1	
yeps_YPTB0729	panC		YPTB_RS04070	YPTB0729		pantoate--beta-alanine ligase	WP_011191752.1	
yeps_YPTB0730	panB		YPTB_RS04075	YPTB0730		3-methyl-2-oxobutanoate hydroxymethyltransferase	WP_012304571.1	
yeps_YPTB0738	mrcB	pbpF,ponB	YPTB_RS04115	YPTB0738		bifunctional glycosyl transferase/transpeptidase	WP_011191759.1	
yeps_YPTB0739	fhuC		YPTB_RS04120	YPTB0739		Fe3+-hydroxamate ABC transporter ATP-binding protein FhuC	WP_002209359.1	
yeps_YPTB0745			YPTB_RS04150	YPTB0745		TRIC cation channel family protein	WP_002209366.1	
yeps_YPTB0746	btuF		YPTB_RS04155	YPTB0746		vitamin B12 ABC transporter substrate-binding protein BtuF	WP_011191763.1	
yeps_YPTB0748	dgt	optA	YPTB_RS04165	YPTB0748		dGTPase	WP_011191765.1	
yeps_YPTB0749	degP		YPTB_RS04170	YPTB0749		serine endoprotease DegP	WP_011191766.1	
yeps_YPTB0753	mazG		YPTB_RS04190	YPTB0753		nucleoside triphosphate pyrophosphohydrolase	WP_011191769.1	
yeps_YPTB0754	pyrG		YPTB_RS04195	YPTB0754		CTP synthase (glutamine hydrolyzing)	WP_002209376.1	
yeps_YPTB0755	eno		YPTB_RS04200	YPTB0755		phosphopyruvate hydratase	WP_011191770.1	
yeps_YPTB0757	queE		YPTB_RS04210	YPTB0757		7-carboxy-7-deazaguanine synthase QueE	WP_002209379.1	
yeps_YPTB0758	queD		YPTB_RS04215	YPTB0758		6-carboxytetrahydropterin synthase QueD	WP_002209380.1	
yeps_YPTB0760	cysI		YPTB_RS04225	YPTB0760		assimilatory sulfite reductase (NADPH) hemoprotein subunit	WP_011191773.1	
yeps_YPTB0763			YPTB_RS04240	YPTB0763		type II toxin-antitoxin system HicA family toxin	WP_002209384.1	
yeps_YPTB0766	cysN		YPTB_RS04260	YPTB0766		sulfate adenylyltransferase subunit CysN	WP_002209387.1	
yeps_YPTB0767	cysC		YPTB_RS04265	YPTB0767		adenylyl-sulfate kinase	WP_002209388.1	
yeps_YPTB0769	ftsB		YPTB_RS04275	YPTB0769		cell division protein FtsB	WP_002209390.1	
yeps_YPTB0771	ispF	mecS,ygbB	YPTB_RS04285	YPTB0771		2-C-methyl-D-erythritol 2,4-cyclodiphosphate synthase	WP_002209392.1	
yeps_YPTB0772	truD		YPTB_RS04290	YPTB0772		tRNA pseudouridine(13) synthase TruD	WP_011191777.1	
yeps_YPTB0773	surE	ygbC	YPTB_RS04295	YPTB0773		5'/3'-nucleotidase SurE	WP_011191778.1	
yeps_YPTB0775	nlpD		YPTB_RS04305	YPTB0775		murein hydrolase activator NlpD	WP_012304557.1	
yeps_YPTB0779	rpiB	yjcA	YPTB_RS04330	YPTB0779		ribose 5-phosphate isomerase B	WP_002209402.1	
yeps_YPTB0780			YPTB_RS04335	YPTB0780		erythritol/L-threitol dehydrogenase	WP_002209403.1	
yeps_YPTB0781			YPTB_RS04340	YPTB0781		D-threitol dehydrogenase	WP_002209404.1	
yeps_YPTB0782			YPTB_RS04345	YPTB0782		dihydroxyacetone kinase subunit DhaK	WP_002228229.1	
yeps_YPTB0783	dhaL		YPTB_RS04350	YPTB0783		dihydroxyacetone kinase subunit L	WP_011191782.1	
yeps_YPTB0792			YPTB_RS04395	YPTB0792		TonB-dependent receptor	WP_002223222.1	
yeps_YPTB0794	map	pepM(S.t.)	YPTB_RS04410	YPTB0794		type I methionyl aminopeptidase	WP_002209416.1	
yeps_YPTB0795			YPTB_RS04415	YPTB0795		ParD-like family protein	WP_002209417.1	
yeps_YPTB0798			YPTB_RS04430	YPTB0798		L-ribulose-5-phosphate 3-epimerase	WP_011191789.1	
yeps_YPTB0800			YPTB_RS04440	YPTB0800		ABC transporter permease	WP_002209421.1	
yeps_YPTB0802			YPTB_RS04450	YPTB0802		substrate-binding domain-containing protein	WP_041175489.1	
yeps_YPTB0803			YPTB_RS04455	YPTB0803		DeoR/GlpR family DNA-binding transcription regulator	WP_011191793.1	
yeps_YPTB0806	dmsB		YPTB_RS04470	YPTB0806		dimethylsulfoxide reductase subunit B	WP_011191795.1	
yeps_YPTB0807			YPTB_RS04475	YPTB0807		dimethyl sulfoxide reductase anchor subunit	WP_011191796.1	
yeps_YPTB0811	katG		YPTB_RS04495	YPTB0811		catalase/peroxidase HPI	WP_002209433.1	
yeps_YPTB0812			YPTB_RS04500	YPTB0812		D-ribose ABC transporter substrate-binding protein	WP_011191800.1	
yeps_YPTB0813			YPTB_RS04505	YPTB0813		DUF2291 family protein	WP_002209435.1	
yeps_YPTB0814			YPTB_RS04510	YPTB0814		sugar ABC transporter ATP-binding protein	WP_011191801.1	
yeps_YPTB0816			YPTB_RS04520	YPTB0816		transketolase	WP_002209438.1	
yeps_YPTB0819			YPTB_RS04535	YPTB0819		L-fucose/L-arabinose isomerase family protein	WP_011191804.1	
yeps_YPTB0820			YPTB_RS04540	YPTB0820		sugar-binding transcriptional regulator	WP_002209443.1	
yeps_YPTB0821			YPTB_RS04545	YPTB0821		MFS transporter	WP_011191805.1	
yeps_YPTB0822	pncC		YPTB_RS04550	YPTB0822		nicotinamide-nucleotide amidase	WP_002209445.1	
yeps_YPTB0825	alaS	act,ala-act,lovB	YPTB_RS04565	YPTB0825		alanine--tRNA ligase	WP_011191808.1	
yeps_YPTB0828			YPTB_RS04600	YPTB0828		DedA family protein	WP_002209451.1	
yeps_YPTB0829	gshA	gsh-I	YPTB_RS04605	YPTB0829		glutamate--cysteine ligase	WP_172957065.1	
yeps_YPTB0832			YPTB_RS04620	YPTB0832		inner membrane protein YpjD	WP_002209455.1	
yeps_YPTB0836	trmD		YPTB_RS04640	YPTB0836		tRNA (guanosine(37)-N1)-methyltransferase TrmD	WP_002222284.1	
yeps_YPTB0850	pssA	pss	YPTB_RS04745	YPTB0850		CDP-diacylglycerol--serine O-phosphatidyltransferase	WP_002208751.1	
yeps_YPTB0853	trxC	yfiG	YPTB_RS04760	YPTB0853		thioredoxin TrxC	WP_011191817.1	
yeps_YPTB0856	emrA		YPTB_RS04775	YPTB0856		multidrug efflux MFS transporter periplasmic adaptor subunit EmrA	WP_002208745.1	
yeps_YPTB0860			YPTB_RS04795	YPTB0860		MFS transporter	WP_011191821.1	
yeps_YPTB0862			YPTB_RS04805	YPTB0862		AtzE family amidohydrolase	WP_011191823.1	
yeps_YPTB0863	hpxX		YPTB_RS04810	YPTB0863		oxalurate catabolism protein HpxX	WP_002208738.1	
yeps_YPTB0866			YPTB_RS04825	YPTB0866		transporter substrate-binding domain-containing protein	WP_011191825.1	
yeps_YPTB0867			YPTB_RS04830	YPTB0867		amino acid ABC transporter permease	WP_002208734.1	
yeps_YPTB0868			YPTB_RS04835	YPTB0868		amino acid ABC transporter permease	WP_011191826.1	
yeps_YPTB0869			YPTB_RS04840	YPTB0869		amino acid ABC transporter ATP-binding protein	WP_002208732.1	
yeps_YPTB0881			YPTB_RS04900	YPTB0881		hypothetical protein	WP_011191838.1	
yeps_YPTB0883	fadE		YPTB_RS04910	YPTB0883		acyl-CoA dehydrogenase FadE	WP_011191839.1	
yeps_YPTB0884	lpcA	gmhA,tfrA,yafI	YPTB_RS04915	YPTB0884		D-sedoheptulose 7-phosphate isomerase	WP_002208720.1	
yeps_YPTB0885			YPTB_RS04920	YPTB0885		class II glutamine amidotransferase	WP_002208719.1	
yeps_YPTB0886			YPTB_RS04925	YPTB0886		murein L,D-transpeptidase	WP_011191840.1	
yeps_YPTB0888			YPTB_RS04935	YPTB0888		NADH:ubiquinone reductase (Na(+)-transporting) subunit B	WP_002208716.1	
yeps_YPTB0891	nqrE	nqr5	YPTB_RS04950	YPTB0891		NADH:ubiquinone reductase (Na(+)-transporting) subunit E	WP_002208713.1	
yeps_YPTB0893			YPTB_RS04960	YPTB0893		FAD:protein FMN transferase	WP_002227821.1	
yeps_YPTB0912	ppnP		YPTB_RS05065	YPTB0912		pyrimidine/purine nucleoside phosphorylase	WP_002208692.1	
yeps_YPTB0914	mak		YPTB_RS05075	YPTB0914		fructokinase	WP_011191852.1	
yeps_YPTB0916	sbcD	yajA	YPTB_RS05085	YPTB0916		exonuclease subunit SbcD	WP_011191854.1	
yeps_YPTB0917	phoB	phoRc	YPTB_RS05095	YPTB0917		phosphate response regulator transcription factor PhoB	WP_002208685.1	
yeps_YPTB0918	phoR		YPTB_RS05100	YPTB0918		phosphate regulon sensor histidine kinase PhoR	WP_002208684.1	
yeps_YPTB0923			YPTB_RS05125	YPTB0923		SDR family oxidoreductase	WP_002208677.1	
yeps_YPTB0927	queA		YPTB_RS05150	YPTB0927		tRNA preQ1(34) S-adenosylmethionine ribosyltransferase-isomerase QueA	WP_011191859.1	
yeps_YPTB0929	yajC		YPTB_RS05160	YPTB0929		preprotein translocase subunit YajC	WP_002208671.1	
yeps_YPTB0935	ribE	ribC	YPTB_RS05195	YPTB0935		6,7-dimethyl-8-ribityllumazine synthase	WP_002208666.1	
yeps_YPTB0937	thiL		YPTB_RS05205	YPTB0937		thiamine-phosphate kinase	WP_011191861.1	
yeps_YPTB0938	pgpA	yajN	YPTB_RS05210	YPTB0938		phosphatidylglycerophosphatase A	WP_002208663.1	
yeps_YPTB0939	dxs		YPTB_RS05215	YPTB0939		1-deoxy-D-xylulose-5-phosphate synthase	WP_011191862.1	
yeps_YPTB0940	ispA		YPTB_RS05220	YPTB0940		(2E,6E)-farnesyl diphosphate synthase	WP_011191863.1	
yeps_YPTB0941	xseB	yajE	YPTB_RS05225	YPTB0941		exodeoxyribonuclease VII small subunit	WP_002208660.1	
yeps_YPTB0942				YPTB0942		hypothetical protein	CAH20182.1	
yeps_YPTB0943	thiI	nuvA,yajJ,yajK	YPTB_RS05235	YPTB0943		tRNA 4-thiouridine(8) synthase ThiI	WP_011191864.1	
yeps_YPTB0944	yajL		YPTB_RS05240	YPTB0944		protein deglycase YajL	WP_002208657.1	
yeps_YPTB0945	panE	abpA	YPTB_RS05245	YPTB0945		2-dehydropantoate 2-reductase	WP_002208656.1	
yeps_YPTB0946			YPTB_RS05250	YPTB0946		YajQ family cyclic di-GMP-binding protein	WP_002208655.1	
yeps_YPTB0949			YPTB_RS05265	YPTB0949		cytochrome o ubiquinol oxidase subunit IV	WP_002208652.1	
yeps_YPTB0954	ampG		YPTB_RS05290	YPTB0954		muropeptide MFS transporter AmpG	WP_002223277.1	
yeps_YPTB0956	bolA		YPTB_RS05300	YPTB0956		transcriptional regulator BolA	WP_002208644.1	
yeps_YPTB0957				YPTB0957		hypothetical protein	CAH20197.1	
yeps_YPTB0965			YPTB_RS05345	YPTB0965		acyl-CoA thioesterase	WP_002208636.1	
yeps_YPTB0967			YPTB_RS05355	YPTB0967		SgrR family transcriptional regulator	WP_011191867.1	
yeps_YPTB0968	cof		YPTB_RS05360	YPTB0968		HMP-PP phosphatase	WP_011191868.1	
yeps_YPTB0973	glnK	ybaI	YPTB_RS05385	YPTB0973		P-II family nitrogen regulator	WP_002208627.1	
yeps_YPTB0974	amtB	ybaG	YPTB_RS05390	YPTB0974		ammonium transporter AmtB	WP_002228344.1	
yeps_YPTB0975	tesB		YPTB_RS05395	YPTB0975		acyl-CoA thioesterase II	WP_002208625.1	
yeps_YPTB0976			YPTB_RS05400	YPTB0976		YbaY family lipoprotein	WP_011191872.1	
yeps_YPTB0986			YPTB_RS05450	YPTB0986		DsrE/DsrF/TusD sulfur relay family protein	WP_002208613.1	
yeps_YPTB0992	dnaX	dnaZ,dnaZX	YPTB_RS05480	YPTB0992		DNA polymerase III subunit gamma/tau	WP_002208605.1	
yeps_YPTB1024			YPTB_RS05650	YPTB1024		NfeD family protein	WP_002208577.1	
yeps_YPTB1026			YPTB_RS05660	YPTB1026		co-chaperone YbbN	WP_011191894.1	
yeps_YPTB1028	tesA	apeA,pldC	YPTB_RS05670	YPTB1028		multifunctional acyl-CoA thioesterase I/protease I/lysophospholipase L1	WP_002208573.1	
yeps_YPTB1030			YPTB_RS05680	YPTB1030		ABC transporter permease	WP_011191896.1	
yeps_YPTB1031	purK		YPTB_RS05685	YPTB1031		5-(carboxyamino)imidazole ribonucleotide synthase	WP_002208570.1	
yeps_YPTB1032	purE		YPTB_RS05690	YPTB1032		5-(carboxyamino)imidazole ribonucleotide mutase	WP_002208569.1	
yeps_YPTB1035	cysS		YPTB_RS05705	YPTB1035		cysteine--tRNA ligase	WP_011191897.1	
yeps_YPTB1036	ybcJ		YPTB_RS05710	YPTB1036		ribosome-associated protein YbcJ	WP_002209775.1	
yeps_YPTB1037	folD	ads	YPTB_RS05715	YPTB1037		bifunctional methylenetetrahydrofolate dehydrogenase/methenyltetrahydrofolate cyclohydrolase FolD	WP_002209774.1	
yeps_YPTB1038			YPTB_RS05725	YPTB1038		AlpA family phage regulatory protein	WP_002215270.1	
yeps_YPTB1040			YPTB_RS05735	YPTB1040		hypothetical protein	WP_011191899.1	
yeps_YPTB1041			YPTB_RS05740	YPTB1041		hypothetical protein	WP_002215266.1	
yeps_YPTB1043			YPTB_RS05745	YPTB1043		hypothetical protein	WP_041175443.1	
yeps_YPTB1044			YPTB_RS05755	YPTB1044		CDP-alcohol phosphatidyltransferase family protein	WP_002209770.1	
yeps_YPTB1045			YPTB_RS05760	YPTB1045		phosphatidate cytidylyltransferase	WP_011191901.1	
yeps_YPTB1047			YPTB_RS05770	YPTB1047		bifunctional alpha/beta hydrolase/class I SAM-dependent methyltransferase	WP_011191902.1	
yeps_YPTB1048			YPTB_RS05775	YPTB1048		phosphatase PAP2/dual specificity phosphatase family protein	WP_011191903.1	
yeps_YPTB1050			YPTB_RS05785	YPTB1050		hypothetical protein	WP_002215253.1	
yeps_YPTB1051			YPTB_RS05790	YPTB1051		LysR family transcriptional regulator	WP_011191905.1	
yeps_YPTB1053			YPTB_RS05800	YPTB1053		aldo/keto reductase	WP_011191906.1	
yeps_YPTB1054			YPTB_RS05815	YPTB1054		hypothetical protein	WP_011191907.1	
yeps_YPTB1056			YPTB_RS05825	YPTB1056		GDYXXLXY domain-containing protein	WP_011191909.1	
yeps_YPTB1057			YPTB_RS05830	YPTB1057		DUF4401 domain-containing protein	WP_011191910.1	
yeps_YPTB1068	pbpC		YPTB_RS05890	YPTB1068		penicillin-binding protein 1C	WP_002223314.1	
yeps_YPTB1071			YPTB_RS05905	YPTB1071		CoA-acylating methylmalonate-semialdehyde dehydrogenase	WP_011191918.1	
yeps_YPTB1073	iolG		YPTB_RS05915	YPTB1073		inositol 2-dehydrogenase	WP_011191920.1	
yeps_YPTB1074			YPTB_RS05920	YPTB1074		substrate-binding domain-containing protein	WP_002210300.1	
yeps_YPTB1075			YPTB_RS05925	YPTB1075		sugar ABC transporter ATP-binding protein	WP_011191921.1	
yeps_YPTB1076			YPTB_RS05930	YPTB1076		ABC transporter permease	WP_002210302.1	
yeps_YPTB1082			YPTB_RS05960	YPTB1082		ABC transporter ATP-binding protein/permease	WP_002214723.1	
yeps_YPTB1083			YPTB_RS05965	YPTB1083		antibiotic biosynthesis monooxygenase	WP_002210309.1	
yeps_YPTB1086			YPTB_RS05985	YPTB1086		LuxR family transcriptional regulator	WP_002210313.1	
yeps_YPTB1087			YPTB_RS05990	YPTB1087		cold shock domain-containing protein	WP_012304395.1	

yeps_YPTB1089	crcB		YPTB_RS06000	YPTB1089		fluoride efflux transporter CrcB	WP_011191930.1	
yeps_YPTB1090	tatA	mttaI	YPTB_RS06010	YPTB1090		Sec-independent protein translocase subunit TatA	WP_002210319.1	
yeps_YPTB1092	lipB		YPTB_RS06020	YPTB1092		lipoyl(octanoyl) transferase LipB	WP_002218201.1	
yeps_YPTB1093			YPTB_RS06025	YPTB1093		DUF493 family protein YbeD	WP_002210322.1	
yeps_YPTB1094	dacA		YPTB_RS06030	YPTB1094		D-alanyl-D-alanine carboxypeptidase DacA	WP_011191931.1	
yeps_YPTB1095	rlpA		YPTB_RS06035	YPTB1095		endolytic peptidoglycan transglycosylase RlpA	WP_002210324.1	
yeps_YPTB1099	rsfS		YPTB_RS06055	YPTB1099		ribosome silencing factor	WP_002210329.1	
yeps_YPTB1100	nadD		YPTB_RS06060	YPTB1100		nicotinate-nucleotide adenylyltransferase	WP_002210330.1	
yeps_YPTB1101	holA		YPTB_RS06065	YPTB1101		DNA polymerase III subunit delta	WP_011191933.1	
yeps_YPTB1102	lptE		YPTB_RS06070	YPTB1102		LPS assembly lipoprotein LptE	WP_002210332.1	
yeps_YPTB1107			YPTB_RS06095	YPTB1107		amino acid ABC transporter permease	WP_002223337.1	
yeps_YPTB1109	lnt		YPTB_RS06105	YPTB1109		apolipoprotein N-acyltransferase	WP_002210341.1	
yeps_YPTB1111	ybeY		YPTB_RS06115	YPTB1111		rRNA maturation RNase YbeY	WP_011191934.1	
yeps_YPTB1117			YPTB_RS06185	YPTB1117		ROK family transcriptional regulator	WP_002224863.1	
yeps_YPTB1118	nagA		YPTB_RS06190	YPTB1118		N-acetylglucosamine-6-phosphate deacetylase	WP_002210351.1	
yeps_YPTB1123			YPTB_RS06215	YPTB1123		beta-N-acetylhexosaminidase	WP_011191940.1	
yeps_YPTB1124	fur		YPTB_RS06220	YPTB1124		ferric iron uptake transcriptional regulator	WP_011191941.1	
yeps_YPTB1126				YPTB1126		hypothetical	CAH20366.1	
yeps_YPTB1136			YPTB_RS06285	YPTB1136		type II toxin-antitoxin system RatA family toxin	WP_002210715.1	
yeps_YPTB1137			YPTB_RS06290	YPTB1137		RnfH family protein	WP_002210716.1	
yeps_YPTB1138	bamE		YPTB_RS06295	YPTB1138		outer membrane protein assembly factor BamE	WP_002210717.1	
yeps_YPTB1140	nadK		YPTB_RS06305	YPTB1140		NAD(+) kinase	WP_011191944.1	
yeps_YPTB1142			YPTB_RS06315	YPTB1142		citrate synthase	WP_002210721.1	
yeps_YPTB1143	sdhC	cybA	YPTB_RS06320	YPTB1143		succinate dehydrogenase cytochrome b556 subunit	WP_002210722.1	
yeps_YPTB1152	cydB		YPTB_RS06365	YPTB1152		cytochrome d ubiquinol oxidase subunit II	WP_002210731.1	
yeps_YPTB1153	cydX		YPTB_RS06370	YPTB1153		cytochrome bd-I oxidase subunit CydX	WP_002210732.1	
yeps_YPTB1154	ybgE		YPTB_RS06375	YPTB1154		cyd operon protein YbgE	WP_011191945.1	
yeps_YPTB1156	tolQ		YPTB_RS06385	YPTB1156		Tol-Pal system protein TolQ	WP_002210735.1	
yeps_YPTB1160	pal	excC	YPTB_RS06405	YPTB1160		peptidoglycan-associated lipoprotein Pal	WP_011191946.1	
yeps_YPTB1167			YPTB_RS06475	YPTB1167		PsiF family protein	WP_011191948.1	
yeps_YPTB1168	galM		YPTB_RS06480	YPTB1168		galactose-1-epimerase	WP_011191949.1	
yeps_YPTB1170	galT	galB	YPTB_RS06490	YPTB1170		galactose-1-phosphate uridylyltransferase	WP_002210749.1	
yeps_YPTB1171	galE	galD	YPTB_RS06495	YPTB1171		UDP-glucose 4-epimerase GalE	WP_011191951.1	
yeps_YPTB1173	modF	phrA	YPTB_RS06510	YPTB1173		molybdate ABC transporter ATP-binding protein ModF	WP_011191952.1	
yeps_YPTB1174	modE	modR	YPTB_RS06515	YPTB1174		molybdenum-dependent transcriptional regulator	WP_002210754.1	
yeps_YPTB1175			YPTB_RS06520	YPTB1175		AcrZ family multidrug efflux pump-associated protein	WP_002217291.1	
yeps_YPTB1176	modA		YPTB_RS06525	YPTB1176		molybdate ABC transporter substrate-binding protein	WP_002210756.1	
yeps_YPTB1178	modC	chlD,narD	YPTB_RS06535	YPTB1178		molybdenum ABC transporter ATP-binding protein ModC	WP_011191953.1	
yeps_YPTB1179			YPTB_RS06540	YPTB1179		pyridoxal phosphatase	WP_011191954.1	
yeps_YPTB1180	pgl		YPTB_RS06545	YPTB1180		6-phosphogluconolactonase	WP_011191955.1	
yeps_YPTB1182	bioB		YPTB_RS06555	YPTB1182		biotin synthase BioB	WP_002210762.1	
yeps_YPTB1183	bioF		YPTB_RS06560	YPTB1183		8-amino-7-oxononanoate synthase	WP_011191957.1	
yeps_YPTB1184	bioC		YPTB_RS06565	YPTB1184		malonyl-ACP O-methyltransferase BioC	WP_011191958.1	
yeps_YPTB1185	bioD		YPTB_RS06570	YPTB1185		dethiobiotin synthase	WP_002216554.1	
yeps_YPTB1186			YPTB_RS06575	YPTB1186		ABC transporter ATP-binding protein	WP_002210766.1	
yeps_YPTB1187	uvrB		YPTB_RS06580	YPTB1187		excinuclease ABC subunit B	WP_011191959.1	
yeps_YPTB1189	yvcK		YPTB_RS06590	YPTB1189		uridine diphosphate-N-acetylglucosamine-binding protein YvcK	WP_002210769.1	
yeps_YPTB1193	moaE	chlA5	YPTB_RS06615	YPTB1193		molybdopterin synthase catalytic subunit MoaE	WP_002210774.1	
yeps_YPTB1197	betI		YPTB_RS06635	YPTB1197		transcriptional regulator BetI	WP_002218278.1	
yeps_YPTB1199			YPTB_RS06645	YPTB1199		LysR family transcriptional regulator	WP_002214199.1	
yeps_YPTB1201	xapA	pndA	YPTB_RS06655	YPTB1201		xanthosine phosphorylase	WP_011191966.1	
yeps_YPTB1202			YPTB_RS06660	YPTB1202		nucleoside permease	WP_011191967.1	
yeps_YPTB1203			YPTB_RS06665	YPTB1203		zinc resistance sensor/chaperone ZraP	WP_011191968.1	
yeps_YPTB1204			YPTB_RS06670	YPTB1204		PAS domain S-box protein	WP_011191969.1	
yeps_YPTB1205			YPTB_RS06675	YPTB1205		sigma 54-interacting transcriptional regulator	WP_011191970.1	
yeps_YPTB1207			YPTB_RS06685	YPTB1207		LysR family transcriptional regulator	WP_002220168.1	
yeps_YPTB1208			YPTB_RS06695	YPTB1208		EthD family reductase	WP_002220163.1	
yeps_YPTB1211			YPTB_RS06710	YPTB1211		ATP-binding cassette domain-containing protein	WP_011191972.1	
yeps_YPTB1220	yajD		YPTB_RS06760	YPTB1220		HNH nuclease YajD	WP_002214185.1	
yeps_YPTB1221			YPTB_RS06765	YPTB1221		suppressor of fused domain protein	WP_011191976.1	
yeps_YPTB1222			YPTB_RS06770	YPTB1222		GlsB/YeaQ/YmgE family stress response membrane protein	WP_002210786.1	
yeps_YPTB1229	urtB		YPTB_RS06805	YPTB1229		urea ABC transporter permease subunit UrtB	WP_002210793.1	
yeps_YPTB1231	urtD		YPTB_RS06815	YPTB1231		urea ABC transporter ATP-binding protein UrtD	WP_011191979.1	
yeps_YPTB1250			YPTB_RS06905	YPTB1250		nicotinamide mononucleotide deamidase-related protein YfaY	WP_011191993.1	
yeps_YPTB1251	eco	eti	YPTB_RS06910	YPTB1251		serine protease inhibitor ecotin	WP_002210815.1	
yeps_YPTB1252			YPTB_RS06915	YPTB1252		2Fe-2S ferredoxin-like protein	WP_002210816.1	
yeps_YPTB1255			YPTB_RS06930	YPTB1255		bifunctional 3-demethylubiquinone 3-O-methyltransferase/2-octaprenyl-6-hydroxy phenol methylase	WP_002210820.1	
yeps_YPTB1257	rcsC	hmwC	YPTB_RS06945	YPTB1257		two-component system sensor histidine kinase RcsC	WP_011171779.1	
yeps_YPTB1258	rcsB		YPTB_RS06950	YPTB1258		transcriptional regulator RcsB	WP_002210824.1	
yeps_YPTB1259	rcsD		YPTB_RS06955	YPTB1259		phosphotransferase RcsD	WP_011191996.1	
yeps_YPTB1263			YPTB_RS06995	YPTB1263		MFS transporter	WP_011191998.1	
yeps_YPTB1268			YPTB_RS07020	YPTB1268		LexA family transcriptional regulator	WP_002208864.1	
yeps_YPTB1270			YPTB_RS07030	YPTB1270		four-carbon acid sugar kinase family protein	WP_012304340.1	
yeps_YPTB1272			YPTB_RS07040	YPTB1272		DeoR/GlpR family DNA-binding transcription regulator	WP_002208861.1	
yeps_YPTB1273			YPTB_RS07045	YPTB1273		hydroxypyruvate isomerase family protein	WP_002208860.1	
yeps_YPTB1274			YPTB_RS07050	YPTB1274		hypothetical protein	WP_011192001.1	
yeps_YPTB1276			YPTB_RS07060	YPTB1276		DUF2635 domain-containing protein	WP_002208857.1	
yeps_YPTB1279			YPTB_RS07075	YPTB1279		phage tail assembly protein	WP_002208854.1	
yeps_YPTB1283			YPTB_RS07100	YPTB1283		phage baseplate assembly protein	WP_002215460.1	
yeps_YPTB1285			YPTB_RS07110	YPTB1285		baseplate J/gp47 family protein	WP_011192005.1	
yeps_YPTB1286			YPTB_RS22575	YPTB1286		YmfQ family protein	WP_011192006.1	
yeps_YPTB1289			YPTB_RS07135	YPTB1289		MurR/RpiR family transcriptional regulator	WP_002208843.1	
yeps_YPTB1290			YPTB_RS07140	YPTB1290		6-phospho-beta-glucosidase	WP_002208842.1	
yeps_YPTB1300			YPTB_RS07205	YPTB1300		DEAD/DEAH box helicase	WP_011192012.1	
yeps_YPTB1301	rsuA		YPTB_RS07210	YPTB1301		16S rRNA pseudouridine(516) synthase RsuA	WP_011192013.1	
yeps_YPTB1304	yejF		YPTB_RS07225	YPTB1304		microcin C ABC transporter ATP-binding protein YejF	WP_011192015.1	
yeps_YPTB1305			YPTB_RS07230	YPTB1305		ABC transporter permease subunit	WP_002353954.1	
yeps_YPTB1306			YPTB_RS07235	YPTB1306		microcin C ABC transporter permease YejB	WP_002208826.1	
yeps_YPTB1307			YPTB_RS07240	YPTB1307		extracellular solute-binding protein	WP_011192016.1	
yeps_YPTB1310			YPTB_RS07255	YPTB1310		phosphatase PAP2 family protein	WP_011192018.1	
yeps_YPTB1311			YPTB_RS07260	YPTB1311		GTP-binding protein	WP_011192019.1	
yeps_YPTB1312				YPTB1312		putative membrane protein	CAH20552.1	
yeps_YPTB1313			YPTB_RS07270	YPTB1313		GntR family transcriptional regulator	WP_002208816.1	
yeps_YPTB1314			YPTB_RS07275	YPTB1314		mannitol dehydrogenase family protein	WP_002224659.1	
yeps_YPTB1315	uxuA		YPTB_RS07280	YPTB1315		mannonate dehydratase	WP_011192020.1	
yeps_YPTB1317	mtr		YPTB_RS07290	YPTB1317		tryptophan permease	WP_002208812.1	
yeps_YPTB1318			YPTB_RS07295	YPTB1318		YkgJ family cysteine cluster protein	WP_011192021.1	
yeps_YPTB1329	fruB	fpr,fruF	YPTB_RS07350	YPTB1329		fused PTS fructose transporter subunit IIA/HPr protein	WP_002208799.1	
yeps_YPTB1342			YPTB_RS07415	YPTB1342		iron ABC transporter permease	WP_002215451.1	
yeps_YPTB1343			YPTB_RS07420	YPTB1343		ABC transporter ATP-binding protein	WP_011192030.1	
yeps_YPTB1347			YPTB_RS07445	YPTB1347		isopenicillin N synthase family oxygenase	WP_011192033.1	
yeps_YPTB1348			YPTB_RS07450	YPTB1348		MetQ/NlpA family ABC transporter substrate-binding protein	WP_011192034.1	
yeps_YPTB1355	ybjG		YPTB_RS07490	YPTB1355		undecaprenyl-diphosphate phosphatase	WP_002208768.1	
yeps_YPTB1356			YPTB_RS07495	YPTB1356		phosphatase PAP2 family protein	WP_024063536.1	
yeps_YPTB1359			YPTB_RS07510	YPTB1359		YbjC family protein	WP_002216036.1	
yeps_YPTB1360			YPTB_RS07520	YPTB1360		YbjN domain-containing protein	WP_002208763.1	
yeps_YPTB1365			YPTB_RS07545	YPTB1365		YbjO family protein	WP_011192038.1	
yeps_YPTB1366	rlmC		YPTB_RS07550	YPTB1366		23S rRNA (uracil(747)-C(5))-methyltransferase RlmC	WP_011192039.1	
yeps_YPTB1369			YPTB_RS07570	YPTB1369		ABC transporter substrate-binding protein	WP_012413601.1	
yeps_YPTB1370			YPTB_RS07575	YPTB1370		iron ABC transporter permease	WP_197684172.1	
yeps_YPTB1371			YPTB_RS07580	YPTB1371		ABC transporter ATP-binding protein	WP_002211375.1	
yeps_YPTB1372			YPTB_RS07585	YPTB1372		nicotianamine synthase	WP_012304311.1	
yeps_YPTB1374			YPTB_RS07595	YPTB1374		DMT family transporter	WP_002223483.1	
yeps_YPTB1375	artM		YPTB_RS07605	YPTB1375		arginine ABC transporter permease ArtM	WP_002211370.1	
yeps_YPTB1378	artP		YPTB_RS07620	YPTB1378		arginine ABC transporter ATP-binding protein ArtP	WP_011192044.1	
yeps_YPTB1379			YPTB_RS07625	YPTB1379		chorismate mutase	WP_002211366.1	
yeps_YPTB1380			YPTB_RS07630	YPTB1380		lipoprotein	WP_002211365.1	
yeps_YPTB1382			YPTB_RS07640	YPTB1382		DUF2867 domain-containing protein	WP_011192045.1	
yeps_YPTB1385	hcr		YPTB_RS07655	YPTB1385		NADH oxidoreductase	WP_011192047.1	
yeps_YPTB1388			YPTB_RS07670	YPTB1388		ATP-dependent endonuclease	WP_002211356.1	
yeps_YPTB1389			YPTB_RS07675	YPTB1389		VirK/YbjX family protein	WP_002211355.1	
yeps_YPTB1392	cspD	cspH	YPTB_RS07690	YPTB1392		cold shock-like protein CspD	WP_002211350.1	
yeps_YPTB1393	clpS		YPTB_RS07695	YPTB1393		ATP-dependent Clp protease adapter ClpS	WP_002211349.1	
yeps_YPTB1396	aat		YPTB_RS07710	YPTB1396		leucyl/phenylalanyl-tRNA--protein transferase	WP_002211346.1	
yeps_YPTB1402	lolA	lplA	YPTB_RS07745	YPTB1402		outer membrane lipoprotein chaperone LolA	WP_002211338.1	
yeps_YPTB1404	serS		YPTB_RS07755	YPTB1404		serine--tRNA ligase	WP_002211336.1	
yeps_YPTB1415	aroA		YPTB_RS07810	YPTB1415		3-phosphoshikimate 1-carboxyvinyltransferase	WP_011192056.1	
yeps_YPTB1419			YPTB_RS07830	YPTB1419		ComEC family protein	WP_011192058.1	
yeps_YPTB1420	msbA		YPTB_RS07835	YPTB1420		lipid A ABC transporter ATP-binding protein/permease MsbA	WP_002211320.1	
yeps_YPTB1421	lpxK		YPTB_RS07840	YPTB1421		tetraacyldisaccharide 4'-kinase	WP_002211319.1	
yeps_YPTB1423			YPTB_RS07855	YPTB1423		cold-shock protein	WP_002211317.1	
yeps_YPTB1424			YPTB_RS07860	YPTB1424		hypothetical protein	WP_002211315.1	
yeps_YPTB1425	kdsB		YPTB_RS07865	YPTB1425		3-deoxy-manno-octulosonate cytidylyltransferase	WP_002211314.1	
yeps_YPTB1426			YPTB_RS07870	YPTB1426		YcbJ family phosphotransferase	WP_011192060.1	
yeps_YPTB1427	cmoM		YPTB_RS07880	YPTB1427		tRNA uridine 5-oxyacetic acid(34) methyltransferase CmoM	WP_002211311.1	
yeps_YPTB1429	mukE	kicA	YPTB_RS07890	YPTB1429		chromosome partition protein MukE	WP_002211309.1	
yeps_YPTB1437	pncB		YPTB_RS07930	YPTB1437		nicotinate phosphoribosyltransferase	WP_002228013.1	
yeps_YPTB1438	pepN		YPTB_RS07935	YPTB1438		aminopeptidase N	WP_011192063.1	
yeps_YPTB1440			YPTB_RS07945	YPTB1440		cell division protein ZapC	WP_011192064.1	
yeps_YPTB1443			YPTB_RS07960	YPTB1443		ABC transporter ATP-binding protein	WP_002211292.1	
yeps_YPTB1450	fabA		YPTB_RS08000	YPTB1450		bifunctional 3-hydroxydecanoyl-ACP dehydratase/trans-2-decenoyl-ACP isomerase	WP_002220006.1	
yeps_YPTB1451			YPTB_RS08005	YPTB1451		Lon protease family protein	WP_002224683.1	
yeps_YPTB1456	yccS		YPTB_RS08030	YPTB1456		TIGR01666 family membrane protein	WP_011192070.1	
yeps_YPTB1457			YPTB_RS08035	YPTB1457		YccF domain-containing protein	WP_011192071.1	
yeps_YPTB1459			YPTB_RS08045	YPTB1459		methylglyoxal synthase	WP_002213060.1	
yeps_YPTB1462	hspQ		YPTB_RS08060	YPTB1462		heat shock protein HspQ	WP_002213054.1	
yeps_YPTB1464	yccX		YPTB_RS08070	YPTB1464		acylphosphatase	WP_002213049.1	
yeps_YPTB1465	tusE		YPTB_RS08075	YPTB1465		sulfurtransferase TusE	WP_002213046.1	
yeps_YPTB1478			YPTB_RS08150	YPTB1478		SDR family oxidoreductase	WP_002213024.1	
yeps_YPTB1479			YPTB_RS08155	YPTB1479		lipocalin-like domain-containing protein	WP_002228570.1	
yeps_YPTB1480			YPTB_RS08160	YPTB1480		phosphopantetheine-binding protein	WP_002213017.1	

yeps_YPTB1481			YPTB_RS08165	YPTB1481		ACP S-malonyltransferase	WP_002213016.1	
yeps_YPTB1517			YPTB_RS08355	YPTB1517		S-(hydroxymethyl)glutathione dehydrogenase/class III alcohol dehydrogenase	WP_002224699.1	
yeps_YPTB1518			YPTB_RS08360	YPTB1518		LysR family transcriptional regulator	WP_002211958.1	
yeps_YPTB1520	folE		YPTB_RS08370	YPTB1520		GTP cyclohydrolase I FolE	WP_002211960.1	
yeps_YPTB1521			YPTB_RS08375	YPTB1521		DUF418 family protein	WP_012413631.1	
yeps_YPTB1522	mglB		YPTB_RS08380	YPTB1522		galactose/glucose ABC transporter substrate-binding protein MglB	WP_002211963.1	
yeps_YPTB1526			YPTB_RS08400	YPTB1526		NAD-dependent malic enzyme	WP_002211968.1	
yeps_YPTB1527	cdd		YPTB_RS08405	YPTB1527		cytidine deaminase	WP_011192106.1	
yeps_YPTB1528			YPTB_RS08410	YPTB1528		CidB/LrgB family autolysis modulator	WP_011192107.1	
yeps_YPTB1536	udk		YPTB_RS08450	YPTB1536		uridine kinase	WP_002211872.1	
yeps_YPTB1538	asmA		YPTB_RS08460	YPTB1538		outer membrane assembly protein AsmA	WP_002211874.1	
yeps_YPTB1542			YPTB_RS08480	YPTB1542		lysine N(6)-hydroxylase/L-ornithine N(5)-oxygenase family protein	WP_002211878.1	
yeps_YPTB1543			YPTB_RS08485	YPTB1543		acetyltransferase	WP_002214999.1	
yeps_YPTB1548			YPTB_RS08510	YPTB1548		iron-siderophore ABC transporter substrate-binding protein	WP_011192116.1	
yeps_YPTB1552	galU		YPTB_RS08530	YPTB1552		UTP--glucose-1-phosphate uridylyltransferase GalU	WP_002211887.1	
yeps_YPTB1556	hisF		YPTB_RS08555	YPTB1556		imidazole glycerol phosphate synthase subunit HisF	WP_011192119.1	
yeps_YPTB1560	hisC		YPTB_RS08575	YPTB1560		histidinol-phosphate transaminase	WP_011192121.1	
yeps_YPTB1561	hisD		YPTB_RS08580	YPTB1561		histidinol dehydrogenase	WP_011192122.1	
yeps_YPTB1562	hisG		YPTB_RS08585	YPTB1562		ATP phosphoribosyltransferase	WP_002211896.1	
yeps_YPTB1563			YPTB_RS08590	YPTB1563		SDR family oxidoreductase	WP_002211898.1	
yeps_YPTB1574			YPTB_RS08660	YPTB1574		thiol-disulfide oxidoreductase DCC family protein	WP_012413636.1	
yeps_YPTB1575			YPTB_RS08665	YPTB1575		SDR family oxidoreductase	WP_002216393.1	
yeps_YPTB1577			YPTB_RS08675	YPTB1577		L-fuconate dehydratase	WP_002211917.1	
yeps_YPTB1579	argG		YPTB_RS08685	YPTB1579		argininosuccinate synthase	WP_002211920.1	
yeps_YPTB1580			YPTB_RS08690	YPTB1580		EmmdR/YeeO family multidrug/toxin efflux MATE transporter	WP_002224723.1	
yeps_YPTB1604			YPTB_RS08830	YPTB1604		TRAP transporter large permease	WP_002213124.1	
yeps_YPTB1605			YPTB_RS08835	YPTB1605		TRAP transporter small permease	WP_002213122.1	
yeps_YPTB1609			YPTB_RS08860	YPTB1609		MFS transporter	WP_002230721.1	
yeps_YPTB1611	mtfA		YPTB_RS08875	YPTB1611		DgsA anti-repressor MtfA	WP_002211042.1	
yeps_YPTB1612			YPTB_RS08885	YPTB1612		YciI family protein	WP_002211043.1	
yeps_YPTB1613			YPTB_RS08890	YPTB1613		serine protein kinase RIO	WP_002211044.1	
yeps_YPTB1633			YPTB_RS09005	YPTB1633		PTS mannose/fructose/sorbose transporter subunit IIC	WP_002211068.1	
yeps_YPTB1634	manX	gptB,ptsL	YPTB_RS09010	YPTB1634		PTS mannose transporter subunit IIAB	WP_002211069.1	
yeps_YPTB1635			YPTB_RS09015	YPTB1635		TerC family protein	WP_002231099.1	
yeps_YPTB1641			YPTB_RS09045	YPTB1641		5-carboxymethyl-2-hydroxymuconate Delta-isomerase	WP_002211073.1	
yeps_YPTB1642	hpaH	hpcG	YPTB_RS09050	YPTB1642		2-oxo-hepta-3-ene-1,7-dioic acid hydratase	WP_011192151.1	
yeps_YPTB1645	hpaB		YPTB_RS09065	YPTB1645		4-hydroxyphenylacetate 3-monooxygenase, oxygenase component	WP_002211077.1	
yeps_YPTB1646	hpaC		YPTB_RS09070	YPTB1646		4-hydroxyphenylacetate 3-monooxygenase, reductase component	WP_002211078.1	
yeps_YPTB1648			YPTB_RS09080	YPTB1648		CoA pyrophosphatase	WP_002211080.1	
yeps_YPTB1649	pabB		YPTB_RS09085	YPTB1649		aminodeoxychorismate synthase component 1	WP_011192153.1	
yeps_YPTB1650			YPTB_RS09090	YPTB1650		YoaH family protein	WP_011192154.1	
yeps_YPTB1652	dbpA		YPTB_RS09100	YPTB1652		ATP-dependent RNA helicase DbpA	WP_011192156.1	
yeps_YPTB1653			YPTB_RS09105	YPTB1653		YebG family protein	WP_002211085.1	
yeps_YPTB1654			YPTB_RS09110	YPTB1654		N(4)-acetylcytidine aminohydrolase	WP_002211086.1	
yeps_YPTB1655			YPTB_RS09115	YPTB1655		protein YebF	WP_012304226.1	
yeps_YPTB1669			YPTB_RS09195	YPTB1669		flagellar synthesis protein FlgN	WP_002211107.1	
yeps_YPTB1670	flgM		YPTB_RS09200	YPTB1670		anti-sigma-28 factor FlgM	WP_002211108.1	
yeps_YPTB1674	flgD	lfgD	YPTB_RS09220	YPTB1674		flagellar hook assembly protein FlgD	WP_011192164.1	
yeps_YPTB1676			YPTB_RS09230	YPTB1676		flagellar basal body rod protein FlgF	WP_011192165.1	
yeps_YPTB1677	flgG		YPTB_RS09235	YPTB1677		flagellar basal-body rod protein FlgG	WP_011192166.1	
yeps_YPTB1678	flgH	flaFVIII,flaY	YPTB_RS09240	YPTB1678		flagellar basal body L-ring protein FlgH	WP_002211116.1	
yeps_YPTB1679			YPTB_RS09245	YPTB1679		flagellar basal body P-ring protein FlgI	WP_002211117.1	
yeps_YPTB1682	flgL	flaT,flaU	YPTB_RS09260	YPTB1682		flagellar hook-associated protein FlgL	WP_002211120.1	
yeps_YPTB1683			YPTB_RS09265	YPTB1683		sugar-binding transcriptional regulator	WP_041175448.1	
yeps_YPTB1684			YPTB_RS09270	YPTB1684		bifunctional aldolase/short-chain dehydrogenase	WP_011192168.1	
yeps_YPTB1686			YPTB_RS09280	YPTB1686		substrate-binding domain-containing protein	WP_002211125.1	
yeps_YPTB1687			YPTB_RS09285	YPTB1687		sugar ABC transporter ATP-binding protein	WP_011192170.1	
yeps_YPTB1688			YPTB_RS09290	YPTB1688		ABC transporter permease	WP_011192171.1	
yeps_YPTB1691	fliR	mopE	YPTB_RS09305	YPTB1691		flagellar type III secretion system protein FliR	WP_011192174.1	
yeps_YPTB1693	fliP	mopC	YPTB_RS09315	YPTB1693		flagellar type III secretion system pore protein FliP	WP_002220494.1	
yeps_YPTB1696	fliM	CheC2,flaAII,flaQII	YPTB_RS09330	YPTB1696		flagellar motor switch protein FliM	WP_011192177.1	
yeps_YPTB1697	fliL	cheC1,flaAI,flaQI	YPTB_RS09335	YPTB1697		flagellar basal body-associated protein FliL	WP_002227961.1	
yeps_YPTB1699	fliJ	flaO,flaS	YPTB_RS09345	YPTB1699		flagella biosynthesis chaperone FliJ	WP_002211139.1	
yeps_YPTB1701	fliH	flaAII.3,flaBIII	YPTB_RS09355	YPTB1701		flagellar assembly protein FliH	WP_011192180.1	
yeps_YPTB1703	fliF	flaAII.1,flaBI	YPTB_RS09365	YPTB1703		flagellar M-ring protein FliF	WP_011192181.1	
yeps_YPTB1704	fliE	flaAI,flaN	YPTB_RS09370	YPTB1704		flagellar hook-basal body complex protein FliE	WP_011192182.1	
yeps_YPTB1705			YPTB_RS09375	YPTB1705		hypothetical protein	WP_002215990.1	
yeps_YPTB1708			YPTB_RS09390	YPTB1708		DNA-3-methyladenine glycosylase	WP_002211145.1	
yeps_YPTB1709			YPTB_RS09395	YPTB1709		metal-dependent phosphohydrolase	WP_011192184.1	
yeps_YPTB1710			YPTB_RS09400	YPTB1710		AraC family transcriptional regulator	WP_002211147.1	
yeps_YPTB1711	fliT		YPTB_RS09410	YPTB1711		flagella biosynthesis regulatory protein FliT	WP_011192185.1	
yeps_YPTB1712	fliS	lafC	YPTB_RS09415	YPTB1712		flagellar export chaperone FliS	WP_011192186.1	
yeps_YPTB1713	fliD	flaV,flbC	YPTB_RS09420	YPTB1713		flagellar filament capping protein FliD	WP_011192187.1	
yeps_YPTB1714			YPTB_RS09425	YPTB1714		FliC/FljB family flagellin	WP_002211152.1	
yeps_YPTB1716	fliZ		YPTB_RS09435	YPTB1716		flagella biosynthesis regulatory protein FliZ	WP_002211154.1	
yeps_YPTB1718	tcyJ		YPTB_RS09445	YPTB1718		cystine ABC transporter substrate-binding protein	WP_002211157.1	
yeps_YPTB1719	tcyL		YPTB_RS09450	YPTB1719		cystine ABC transporter permease	WP_011192190.1	
yeps_YPTB1722			YPTB_RS09465	YPTB1722		DUF6516 family protein	WP_012413668.1	
yeps_YPTB1725			YPTB_RS09485	YPTB1725		FTR1 family protein	WP_002403646.1	
yeps_YPTB1728	wrbA		YPTB_RS09500	YPTB1728		NAD(P)H:quinone oxidoreductase	WP_002211168.1	
yeps_YPTB1729			YPTB_RS09505	YPTB1729		N-acetylneuraminate epimerase	WP_002211169.1	
yeps_YPTB1912			YPTB_RS10495	YPTB1912		Gfo/Idh/MocA family oxidoreductase	WP_011192351.1	
yeps_YPTB1915			YPTB_RS10510	YPTB1915		carbohydrate ABC transporter permease	WP_002213186.1	
yeps_YPTB1916	ugpC		YPTB_RS10515	YPTB1916		sn-glycerol-3-phosphate ABC transporter ATP-binding protein UgpC	WP_011192353.1	
yeps_YPTB1917			YPTB_RS10530	YPTB1917		molecular chaperone	WP_002227954.1	
yeps_YPTB1919			YPTB_RS10540	YPTB1919		fimbrial biogenesis outer membrane usher protein	WP_032466356.1	
yeps_YPTB1920			YPTB_RS10545	YPTB1920		molecular chaperone	WP_011192355.1	
yeps_YPTB1926	ripC		YPTB_RS10575	YPTB1926		itaconate degradation C-C-lyase RipC	WP_002212068.1	
yeps_YPTB1927	ripR		YPTB_RS10580	YPTB1927		itaconate degradation transcriptional regulator RipR	WP_012105184.1	
yeps_YPTB1928			YPTB_RS10585	YPTB1928		carboxypeptidase-like regulatory domain-containing protein	WP_011192359.1	
yeps_YPTB1929			YPTB_RS10590	YPTB1929		hypothetical protein	WP_011192360.1	
yeps_YPTB1931			YPTB_RS10600	YPTB1931		amidohydrolase	WP_011192361.1	
yeps_YPTB1933	nqrE	nqr5	YPTB_RS10610	YPTB1933		NADH:ubiquinone reductase (Na(+)-transporting) subunit E	WP_002212061.1	
yeps_YPTB1944			YPTB_RS10670	YPTB1944		ABC transporter ATP-binding protein	WP_011192364.1	
yeps_YPTB1946			YPTB_RS10680	YPTB1946		cytochrome c	WP_002227951.1	
yeps_YPTB1950	pgaB		YPTB_RS10700	YPTB1950		poly-beta-1,6-N-acetyl-D-glucosamine N-deacetylase PgaB	WP_002212043.1	
yeps_YPTB1951			YPTB_RS10705	YPTB1951		poly-beta-1,6 N-acetyl-D-glucosamine synthase	WP_002224456.1	
yeps_YPTB1952	pgaD		YPTB_RS10710	YPTB1952		poly-beta-1,6-N-acetyl-D-glucosamine biosynthesis protein PgaD	WP_011192367.1	
yeps_YPTB1954			YPTB_RS10720	YPTB1954		hypothetical protein	WP_002212038.1	
yeps_YPTB1957	narX	narR	YPTB_RS10735	YPTB1957		nitrate/nitrite two-component system sensor histidine kinase NarX	WP_011192369.1	
yeps_YPTB1965	hutG		YPTB_RS10780	YPTB1965		N-formylglutamate deformylase	WP_011192373.1	
yeps_YPTB1968			YPTB_RS10795	YPTB1968		formimidoylglutamate deiminase	WP_011192376.1	
yeps_YPTB1969			YPTB_RS10800	YPTB1969		HutD family protein	WP_011192377.1	
yeps_YPTB1972			YPTB_RS10815	YPTB1972		NAD(P)-dependent oxidoreductase	WP_011192380.1	
yeps_YPTB1974			YPTB_RS10825	YPTB1974		coenzyme F390 synthetase	WP_011192382.1	
yeps_YPTB1975			YPTB_RS10830	YPTB1975		NAD(P)-dependent oxidoreductase	WP_012413710.1	
yeps_YPTB1977			YPTB_RS10840	YPTB1977		hypothetical protein	WP_002211270.1	
yeps_YPTB1978			YPTB_RS10845	YPTB1978		glycosyltransferase	WP_011192385.1	
yeps_YPTB1983			YPTB_RS10875	YPTB1983		RpiB/LacA/LacB family sugar-phosphate isomerase	WP_002211263.1	
yeps_YPTB1984			YPTB_RS10880	YPTB1984		class I SAM-dependent methyltransferase	WP_002211262.1	
yeps_YPTB1985			YPTB_RS10885	YPTB1985		SDR family oxidoreductase	WP_011192390.1	
yeps_YPTB1986			YPTB_RS10890	YPTB1986		hypothetical protein	WP_002211260.1	
yeps_YPTB1987			YPTB_RS10895	YPTB1987		hypothetical protein	WP_002211259.1	
yeps_YPTB1990			YPTB_RS10910	YPTB1990		cupin domain-containing protein	WP_002211256.1	
yeps_YPTB1991			YPTB_RS10915	YPTB1991		carboxymuconolactone decarboxylase family protein	WP_011192392.1	
yeps_YPTB1992			YPTB_RS10920	YPTB1992		heavy metal sensor histidine kinase	WP_002211254.1	
yeps_YPTB1993			YPTB_RS10925	YPTB1993		heavy metal response regulator transcription factor	WP_011192393.1	
yeps_YPTB1998	ychF		YPTB_RS10950	YPTB1998		redox-regulated ATPase YchF	WP_002211245.1	
yeps_YPTB2000	ychH		YPTB_RS10960	YPTB2000		stress-induced protein YchH	WP_002211242.1	
yeps_YPTB2003	lolB		YPTB_RS10985	YPTB2003		lipoprotein insertase outer membrane protein LolB	WP_011192397.1	
yeps_YPTB2004	hemA		YPTB_RS10990	YPTB2004		glutamyl-tRNA reductase	WP_002211237.1	
yeps_YPTB2006	prmC		YPTB_RS11000	YPTB2006		peptide chain release factor N(5)-glutamine methyltransferase	WP_011192399.1	
yeps_YPTB2014			YPTB_RS11050	YPTB2014		ABC transporter permease	WP_011192400.1	
yeps_YPTB2015			YPTB_RS11055	YPTB2015		ABC transporter permease	WP_002211226.1	
yeps_YPTB2020			YPTB_RS11085	YPTB2020		molecular chaperone	WP_002211220.1	
yeps_YPTB2021			YPTB_RS11090	YPTB2021		lipoprotein	WP_002211218.1	
yeps_YPTB2029	argS		YPTB_RS11130	YPTB2029		arginine--tRNA ligase	WP_011192408.1	
yeps_YPTB2033	cmoA		YPTB_RS11150	YPTB2033		carboxy-S-adenosyl-L-methionine synthase CmoA	WP_002211207.1	
yeps_YPTB2034			YPTB_RS11155	YPTB2034		MAPEG family protein	WP_002211206.1	
yeps_YPTB2037	nudB	ntpA	YPTB_RS11170	YPTB2037		dihydroneopterin triphosphate diphosphatase	WP_002211203.1	
yeps_YPTB2039	ruvC		YPTB_RS11180	YPTB2039		crossover junction endodeoxyribonuclease RuvC	WP_002211201.1	
yeps_YPTB2041	ruvB		YPTB_RS11190	YPTB2041		Holliday junction branch migration DNA helicase RuvB	WP_002211198.1	
yeps_YPTB2043	znuC		YPTB_RS11200	YPTB2043		zinc ABC transporter ATP-binding protein ZnuC	WP_011192413.1	
yeps_YPTB2044	znuA		YPTB_RS11205	YPTB2044		zinc ABC transporter substrate-binding protein ZnuA	WP_011192414.1	
yeps_YPTB2048			YPTB_RS11225	YPTB2048		MurR/RpiR family transcriptional regulator	WP_002211192.1	
yeps_YPTB2049	zwf		YPTB_RS11230	YPTB2049		glucose-6-phosphate dehydrogenase	WP_011906215.1	
yeps_YPTB2054			YPTB_RS11260	YPTB2054		ATP-dependent DNA helicase	WP_011192419.1	
yeps_YPTB2055	tsaB		YPTB_RS11265	YPTB2055		tRNA (adenosine(37)-N6)-threonylcarbamoyltransferase complex dimerization subunit type 1 TsaB	WP_011192420.1	
yeps_YPTB2062			YPTB_RS11300	YPTB2062		YcgL domain-containing protein	WP_002211743.1	
yeps_YPTB2063			YPTB_RS11305	YPTB2063		lytic murein transglycosylase	WP_011192421.1	
yeps_YPTB2065			YPTB_RS11315	YPTB2065		YcgN family cysteine cluster protein	WP_002211739.1	
yeps_YPTB2076			YPTB_RS11370	YPTB2076		pirin family protein	WP_011192427.1	
yeps_YPTB2085			YPTB_RS11415	YPTB2085		YeaC family protein	WP_002217933.1	
yeps_YPTB2087	ansA		YPTB_RS11425	YPTB2087		asparaginase	WP_011192430.1	
yeps_YPTB2088	sppA		YPTB_RS11430	YPTB2088		signal peptide peptidase SppA	WP_011192431.1	
yeps_YPTB2091			YPTB_RS11445	YPTB2091		DNA topoisomerase III	WP_041175458.1	
yeps_YPTB2119			YPTB_RS11590	YPTB2119		septation protein A	WP_002210640.1	
yeps_YPTB2124			YPTB_RS11615	YPTB2124		BON domain-containing protein	WP_002210636.1	
yeps_YPTB2127	trpCF		YPTB_RS11630	YPTB2127		bifunctional indole-3-glycerol-phosphate synthase TrpC/phosphoribosylanthranilate isomerase TrpF	WP_011192443.1	
yeps_YPTB2130			YPTB_RS11645	YPTB2130		anthranilate synthase component 1	WP_002228454.1	
yeps_YPTB2136	cobO		YPTB_RS11675	YPTB2136		cob(I)yrinic acid a,c-diamide adenosyltransferase	WP_002210626.1	

yeps_YPTB2138	sohB		YPTB_RS11685	YPTB2138		protease SohB	WP_002210624.1	
yeps_YPTB2153			YPTB_RS11765	YPTB2153		DUF2164 domain-containing protein	WP_002210609.1	
yeps_YPTB2154	fsa		YPTB_RS11770	YPTB2154		fructose-6-phosphate aldolase	WP_002215192.1	
yeps_YPTB2155			YPTB_RS11775	YPTB2155		YbdD/YjiX family protein	WP_002210607.1	
yeps_YPTB2157			YPTB_RS11785	YPTB2157		exoribonuclease II	WP_011192452.1	
yeps_YPTB2158			YPTB_RS11790	YPTB2158		MFS transporter	WP_011192453.1	
yeps_YPTB2159			YPTB_RS11795	YPTB2159		SDR family oxidoreductase	WP_011192454.1	
yeps_YPTB2161			YPTB_RS11805	YPTB2161		electron transport complex subunit E	WP_011192455.1	
yeps_YPTB2162	rsxG		YPTB_RS11810	YPTB2162		electron transport complex subunit RsxG	WP_002210600.1	
yeps_YPTB2168			YPTB_RS11840	YPTB2168		DUF2569 domain-containing protein	WP_002210594.1	
yeps_YPTB2170			YPTB_RS11850	YPTB2170		HlyD family type I secretion periplasmic adaptor subunit	WP_011192461.1	
yeps_YPTB2173			YPTB_RS11865	YPTB2173		ribulokinase	WP_011192464.1	
yeps_YPTB2175	araG		YPTB_RS11875	YPTB2175		L-arabinose ABC transporter ATP-binding protein AraG	WP_002210588.1	
yeps_YPTB2176	araH		YPTB_RS11880	YPTB2176		L-arabinose ABC transporter permease AraH	WP_164491550.1	
yeps_YPTB2177	araC		YPTB_RS11885	YPTB2177		arabinose operon transcriptional regulator AraC	WP_011192467.1	
yeps_YPTB2178			YPTB_RS11890	YPTB2178		oxidoreductase	WP_002210585.1	
yeps_YPTB2179			YPTB_RS11895	YPTB2179		bile acid:sodium symporter	WP_011192468.1	
yeps_YPTB2184			YPTB_RS11925	YPTB2184		YdgA family protein	WP_011192473.1	
yeps_YPTB2200			YPTB_RS12010	YPTB2200		pyridoxal phosphate-dependent aminotransferase	WP_011192488.1	
yeps_YPTB2206			YPTB_RS12040	YPTB2206		sugar ABC transporter ATP-binding protein	WP_011192492.1	
yeps_YPTB2209			YPTB_RS12055	YPTB2209		ABC transporter permease	WP_002211034.1	
yeps_YPTB2210			YPTB_RS12060	YPTB2210		ABC transporter ATP-binding protein	WP_011192495.1	
yeps_YPTB2211			YPTB_RS12065	YPTB2211		AMP nucleosidase	WP_002223601.1	
yeps_YPTB2217	ilvN		YPTB_RS12095	YPTB2217		acetolactate synthase small subunit	WP_002230908.1	
yeps_YPTB2218	yjjG		YPTB_RS12100	YPTB2218		pyrimidine 5'-nucleotidase	WP_002211025.1	
yeps_YPTB2221	ogt		YPTB_RS12120	YPTB2221		methylated-DNA--[protein]-cysteine S-methyltransferase	WP_002211023.1	
yeps_YPTB2222			YPTB_RS12125	YPTB2222		FNR family transcription factor	WP_011192502.1	
yeps_YPTB2225	pntA		YPTB_RS12140	YPTB2225		Re/Si-specific NAD(P)(+) transhydrogenase subunit alpha	WP_011192503.1	
yeps_YPTB2226				YPTB2226		hypothetical protein	CAH21464.1	
yeps_YPTB2227			YPTB_RS12150	YPTB2227		DUF1471 family protein YdgH	WP_002211018.1	
yeps_YPTB2228			YPTB_RS12155	YPTB2228		amino acid permease	WP_002211017.1	
yeps_YPTB2229			YPTB_RS12160	YPTB2229		hypothetical protein	WP_002211016.1	
yeps_YPTB2230	rstA	urpT	YPTB_RS12165	YPTB2230		two-component system response regulator RstA	WP_002211015.1	
yeps_YPTB2231	rstB	uspT	YPTB_RS12170	YPTB2231		two-component system sensor histidine kinase RstB	WP_011192505.1	
yeps_YPTB2232			YPTB_RS12175	YPTB2232		carboxypeptidase M32	WP_011192506.1	
yeps_YPTB2239			YPTB_RS12215	YPTB2239		helix-turn-helix domain-containing protein	WP_002211007.1	
yeps_YPTB2241	hrpA		YPTB_RS12225	YPTB2241		ATP-dependent RNA helicase HrpA	WP_012413748.1	
yeps_YPTB2242	azoR		YPTB_RS12230	YPTB2242		FMN-dependent NADH-azoreductase	WP_002211004.1	
yeps_YPTB2246			YPTB_RS12250	YPTB2246		YnbE family lipoprotein	WP_002211000.1	
yeps_YPTB2248			YPTB_RS12260	YPTB2248		2-hydroxyacid dehydrogenase	WP_002210998.1	
yeps_YPTB2251			YPTB_RS12275	YPTB2251		MgtC/SapB family protein	WP_011192514.1	
yeps_YPTB2252			YPTB_RS12280	YPTB2252		multidrug efflux SMR transporter	WP_002210994.1	
yeps_YPTB2254	ttcA		YPTB_RS12290	YPTB2254		tRNA 2-thiocytidine(32) synthetase TtcA	WP_011192516.1	
yeps_YPTB2258			YPTB_RS12310	YPTB2258		ABC transporter substrate-binding protein	WP_002210987.1	
yeps_YPTB2262	tyrR		YPTB_RS12330	YPTB2262		transcriptional regulator TyrR	WP_011192519.1	
yeps_YPTB2264			YPTB_RS12340	YPTB2264		RES family NAD+ phosphorylase	WP_011192520.1	
yeps_YPTB2265			YPTB_RS12345	YPTB2265		YcjF family protein	WP_002210980.1	
yeps_YPTB2267	pspD		YPTB_RS12355	YPTB2267		phage shock protein PspD	WP_002216375.1	
yeps_YPTB2268	pspC		YPTB_RS12360	YPTB2268		envelope stress response membrane protein PspC	WP_002210977.1	
yeps_YPTB2269	pspB		YPTB_RS12365	YPTB2269		envelope stress response membrane protein PspB	WP_002210976.1	
yeps_YPTB2270	pspA		YPTB_RS12370	YPTB2270		phage shock protein PspA	WP_011192521.1	
yeps_YPTB2276	sapF		YPTB_RS12405	YPTB2276		peptide ABC transporter ATP-binding protein SapF	WP_002210969.1	
yeps_YPTB2279			YPTB_RS12420	YPTB2279		YIP1 family protein	WP_002210965.1	
yeps_YPTB2280			YPTB_RS12425	YPTB2280		DUF3811 domain-containing protein	WP_002210964.1	
yeps_YPTB2281	gstA		YPTB_RS12430	YPTB2281		glutathione transferase GstA	WP_002210962.1	
yeps_YPTB2282	pdxY		YPTB_RS12435	YPTB2282		pyridoxal kinase PdxY	WP_011192523.1	
yeps_YPTB2284	pdxH		YPTB_RS12445	YPTB2284		pyridoxamine 5'-phosphate oxidase	WP_002210959.1	
yeps_YPTB2285			YPTB_RS12450	YPTB2285		lipoprotein	WP_002218323.1	
yeps_YPTB2292			YPTB_RS12485	YPTB2292		TetR/AcrR family transcriptional regulator	WP_002210951.1	
yeps_YPTB2293			YPTB_RS12490	YPTB2293		alkene reductase	WP_011192525.1	
yeps_YPTB2295	gloA		YPTB_RS12500	YPTB2295		lactoylglutathione lyase	WP_011192527.1	
yeps_YPTB2298			YPTB_RS12520	YPTB2298		C40 family peptidase	WP_012105035.1	
yeps_YPTB2299	sodB		YPTB_RS12525	YPTB2299		superoxide dismutase [Fe]	WP_011192530.1	
yeps_YPTB2300	purR		YPTB_RS12530	YPTB2300		HTH-type transcriptional repressor PurR	WP_002210943.1	
yeps_YPTB2303	cfa	cdfA	YPTB_RS12545	YPTB2303		cyclopropane fatty acyl phospholipid synthase	WP_002210940.1	
yeps_YPTB2304			YPTB_RS12550	YPTB2304		riboflavin synthase	WP_011192532.1	
yeps_YPTB2309	sufE		YPTB_RS12590	YPTB2309		cysteine desulfuration protein SufE	WP_002211804.1	
yeps_YPTB2310	sufS		YPTB_RS12595	YPTB2310		cysteine desulfurase SufS	WP_011192533.1	
yeps_YPTB2313	sufB		YPTB_RS12610	YPTB2313		Fe-S cluster assembly protein SufB	WP_011192536.1	
yeps_YPTB2314	sufA		YPTB_RS12615	YPTB2314		Fe-S cluster assembly scaffold SufA	WP_002211809.1	
yeps_YPTB2382			YPTB_RS12960	YPTB2382		spore coat U domain-containing protein	WP_002216613.1	
yeps_YPTB2387			YPTB_RS12985	YPTB2387		YebV family protein	WP_002210859.1	
yeps_YPTB2388			YPTB_RS12990	YPTB2388		ASCH domain-containing protein	WP_011192562.1	
yeps_YPTB2393			YPTB_RS13020	YPTB2393		hypothetical protein	WP_002210869.1	
yeps_YPTB2395			YPTB_RS13030	YPTB2395		N-acetylmuramoyl-L-alanine amidase	WP_011192565.1	
yeps_YPTB2398			YPTB_RS13050	YPTB2398		chemotaxis response regulator protein-glutamate methylesterase	WP_011192566.1	
yeps_YPTB2399	cheR	cheX	YPTB_RS13055	YPTB2399		protein-glutamate O-methyltransferase CheR	WP_002214065.1	
yeps_YPTB2401			YPTB_RS13065	YPTB2401		methyl-accepting chemotaxis protein	WP_011192568.1	
yeps_YPTB2413	dsrB		YPTB_RS24100	YPTB2413		protein DsrB	WP_002210892.1	
yeps_YPTB2418			YPTB_RS13155	YPTB2418		Lrp/AsnC family transcriptional regulator	WP_002210900.1	
yeps_YPTB2419			YPTB_RS13160	YPTB2419		Kdo hydroxylase family protein	WP_012413770.1	
yeps_YPTB2420	ydfZ		YPTB_RS13165	YPTB2420		putative selenium delivery protein YdfZ	WP_002210902.1	
yeps_YPTB2421			YPTB_RS13170	YPTB2421		AppA family phytase/histidine-type acid phosphatase	WP_011192581.1	
yeps_YPTB2425			YPTB_RS13195	YPTB2425		hypothetical protein	WP_002210908.1	
yeps_YPTB2430	mnmA		YPTB_RS13220	YPTB2430		tRNA 2-thiouridine(34) synthase MnmA	WP_002210913.1	
yeps_YPTB2435	phoQ		YPTB_RS13245	YPTB2435		two-component system sensor histidine kinase PhoQ	WP_002210918.1	
yeps_YPTB2438	cobB		YPTB_RS13260	YPTB2438		NAD-dependent protein deacylase	WP_002210921.1	
yeps_YPTB2439	nagK		YPTB_RS13265	YPTB2439		N-acetylglucosamine kinase	WP_011192586.1	
yeps_YPTB2440	lolE		YPTB_RS13270	YPTB2440		lipoprotein-releasing ABC transporter permease subunit LolE	WP_011192587.1	
yeps_YPTB2441	lolD		YPTB_RS13275	YPTB2441		lipoprotein-releasing ABC transporter ATP-binding protein LolD	WP_011192588.1	
yeps_YPTB2449	nagZ		YPTB_RS13315	YPTB2449		beta-N-acetylhexosaminidase	WP_032466495.1	
yeps_YPTB2451	lpoB		YPTB_RS13325	YPTB2451		penicillin-binding protein activator LpoB	WP_011192593.1	
yeps_YPTB2452			YPTB_RS13330	YPTB2452		YcfL family protein	WP_002213088.1	
yeps_YPTB2453	hinT		YPTB_RS13335	YPTB2453		purine nucleoside phosphoramidase	WP_002213087.1	
yeps_YPTB2463	ptsG	glcA,umG	YPTB_RS13390	YPTB2463		PTS glucose transporter subunit IIBC	WP_011192602.1	
yeps_YPTB2464			YPTB_RS13395	YPTB2464		metal-dependent hydrolase	WP_002213084.1	
yeps_YPTB2465	holB		YPTB_RS13400	YPTB2465		DNA polymerase III subunit delta'	WP_011192603.1	
yeps_YPTB2466			YPTB_RS13405	YPTB2466		dTMP kinase	WP_011192604.1	
yeps_YPTB2469	fabF	fabJ	YPTB_RS13420	YPTB2469		beta-ketoacyl-ACP synthase II	WP_002213079.1	
yeps_YPTB2472	fabD	tfpA	YPTB_RS13435	YPTB2472		ACP S-malonyltransferase	WP_002210934.1	
yeps_YPTB2473			YPTB_RS13440	YPTB2473		ketoacyl-ACP synthase III	WP_002210933.1	
yeps_YPTB2476	yceD		YPTB_RS13455	YPTB2476		23S rRNA accumulation protein YceD	WP_002210930.1	
yeps_YPTB2480			YPTB_RS22940	YPTB2480		IS3 family transposase	CAH21718.1	
yeps_YPTB2481			YPTB_RS13475	YPTB2481		antibiotic biosynthesis monooxygenase	WP_002213107.1	
yeps_YPTB2482	pyrC		YPTB_RS13480	YPTB2482		dihydroorotase	WP_011192610.1	
yeps_YPTB2483	dinI		YPTB_RS13485	YPTB2483		DNA damage-inducible protein I	WP_002213110.1	
yeps_YPTB2484	bssS		YPTB_RS13495	YPTB2484		biofilm formation regulator BssS	WP_011192611.1	
yeps_YPTB2488			YPTB_RS13515	YPTB2488		rhodanese-related sulfurtransferase	WP_002211854.1	
yeps_YPTB2489				YPTB2489		putative membrane protein	CAH21727.1	
yeps_YPTB2503	fae		YPTB_RS13600	YPTB2503		formaldehyde-activating enzyme	WP_011192622.1	
yeps_YPTB2504			YPTB_RS13605	YPTB2504		aldo/keto reductase	WP_011192623.1	
yeps_YPTB2505	cas6f		YPTB_RS13610	YPTB2505		type I-F CRISPR-associated endoribonuclease Cas6/Csy4	WP_002211866.1	
yeps_YPTB2506	csy3		YPTB_RS13615	YPTB2506		type I-F CRISPR-associated protein Csy3	WP_011192624.1	
yeps_YPTB2507	csy2		YPTB_RS13620	YPTB2507		type I-F CRISPR-associated protein Csy2	WP_011192625.1	
yeps_YPTB2508	csy1		YPTB_RS13625	YPTB2508		type I-F CRISPR-associated protein Csy1	WP_011192626.1	
yeps_YPTB2513			YPTB_RS13650	YPTB2513		hypothetical protein	WP_002230953.1	
yeps_YPTB2514			YPTB_RS13655	YPTB2514		DUF1861 family protein	WP_011192631.1	
yeps_YPTB2516			YPTB_RS13665	YPTB2516		carbohydrate ABC transporter permease	WP_002210197.1	
yeps_YPTB2517			YPTB_RS13670	YPTB2517		sugar ABC transporter permease	WP_011192633.1	
yeps_YPTB2518			YPTB_RS13675	YPTB2518		ABC transporter substrate-binding protein	WP_011192634.1	
yeps_YPTB2519			YPTB_RS13680	YPTB2519		LacI family DNA-binding transcriptional regulator	WP_002210200.1	
yeps_YPTB2520			YPTB_RS13685	YPTB2520		phosphomannomutase/phosphoglucomutase	WP_002210201.1	
yeps_YPTB2535			YPTB_RS13765	YPTB2535		sugar ABC transporter permease	WP_002210221.1	
yeps_YPTB2536			YPTB_RS13770	YPTB2536		sugar ABC transporter ATP-binding protein	WP_011192642.1	
yeps_YPTB2537			YPTB_RS13775	YPTB2537		substrate-binding domain-containing protein	WP_011192643.1	
yeps_YPTB2541			YPTB_RS13795	YPTB2541		cation diffusion facilitator family transporter	WP_011192647.1	
yeps_YPTB2542	ompX	omp4	YPTB_RS13800	YPTB2542		outer membrane protein OmpX	WP_002210229.1	
yeps_YPTB2543	rhtA		YPTB_RS13805	YPTB2543		threonine/homoserine exporter RhtA	WP_002213809.1	
yeps_YPTB2544			YPTB_RS13810	YPTB2544		GNAT family N-acetyltransferase	WP_002210231.1	
yeps_YPTB2546	dps		YPTB_RS13820	YPTB2546		DNA starvation/stationary phase protection protein Dps	WP_002210233.1	
yeps_YPTB2547			YPTB_RS13825	YPTB2547		YdcF family protein	WP_002210234.1	
yeps_YPTB2548	glnH		YPTB_RS13835	YPTB2548		glutamine ABC transporter substrate-binding protein GlnH	WP_011192648.1	
yeps_YPTB2550	glnQ		YPTB_RS13845	YPTB2550		glutamine ABC transporter ATP-binding protein GlnQ	WP_011192649.1	
yeps_YPTB2556	menE		YPTB_RS13870	YPTB2556		o-succinylbenzoate--CoA ligase	WP_011192653.1	
yeps_YPTB2559	menH		YPTB_RS13885	YPTB2559		2-succinyl-6-hydroxy-2,4-cyclohexadiene-1-carboxylate synthase	WP_011192655.1	
yeps_YPTB2560	menD		YPTB_RS13890	YPTB2560		2-succinyl-5-enolpyruvyl-6-hydroxy-3-cyclohexene-1-carboxylic-acid synthase	WP_011192656.1	
yeps_YPTB2561	menF		YPTB_RS13895	YPTB2561		isochorismate synthase MenF	WP_011192657.1	
yeps_YPTB2572			YPTB_RS13960	YPTB2572		gluconokinase	WP_002210265.1	
yeps_YPTB2575	nuoN		YPTB_RS13975	YPTB2575		NADH-quinone oxidoreductase subunit NuoN	WP_002210268.1	
yeps_YPTB2576	nuoM		YPTB_RS13980	YPTB2576		NADH-quinone oxidoreductase subunit M	WP_011192666.1	
yeps_YPTB2579	nuoJ		YPTB_RS13995	YPTB2579		NADH-quinone oxidoreductase subunit J	WP_002210272.1	
yeps_YPTB2582	nuoG		YPTB_RS14010	YPTB2582		NADH-quinone oxidoreductase subunit NuoG	WP_011192668.1	
yeps_YPTB2583	nuoF		YPTB_RS14015	YPTB2583		NADH-quinone oxidoreductase subunit NuoF	WP_011192669.1	
yeps_YPTB2584	nuoE		YPTB_RS14020	YPTB2584		NADH-quinone oxidoreductase subunit NuoE	WP_172601542.1	
yeps_YPTB2593			YPTB_RS14070	YPTB2593		sugar phosphatase	WP_011192675.1	
yeps_YPTB2596				YPTB2596		hypothetical protein	CAH21834.1	
yeps_YPTB2603	yfcD		YPTB_RS14120	YPTB2603		NUDIX hydrolase YfcD	WP_002209742.1	
yeps_YPTB2605			YPTB_RS14130	YPTB2605		EAL domain-containing protein	WP_012303928.1	
yeps_YPTB2606			YPTB_RS14135	YPTB2606		TIGR01777 family oxidoreductase	WP_002209739.1	
yeps_YPTB2611			YPTB_RS14160	YPTB2611		UbiX family flavin prenyltransferase	WP_002209734.1	
yeps_YPTB2616	accD	dedB,usg	YPTB_RS14185	YPTB2616		acetyl-CoA carboxylase, carboxyltransferase subunit beta	WP_002209729.1	

yeps_YPTB2621			YPTB_RS14210	YPTB2621		helix-turn-helix transcriptional regulator	WP_011192684.1	
yeps_YPTB2628			YPTB_RS14250	YPTB2628		YfcL family protein	WP_002209715.1	
yeps_YPTB2631	mepA		YPTB_RS14265	YPTB2631		penicillin-insensitive murein endopeptidase	WP_002225365.1	
yeps_YPTB2633	prmB		YPTB_RS14275	YPTB2633		50S ribosomal protein L3 N(5)-glutamine methyltransferase	WP_002209710.1	
yeps_YPTB2634	smrB		YPTB_RS14280	YPTB2634		endonuclease SmrB	WP_002227846.1	
yeps_YPTB2635	sixA		YPTB_RS14285	YPTB2635		phosphohistidine phosphatase SixA	WP_002209707.1	
yeps_YPTB2638			YPTB_RS14300	YPTB2638		YfcZ/YiiS family protein	WP_002227845.1	
yeps_YPTB2641	ccmI		YPTB_RS14315	YPTB2641		c-type cytochrome biogenesis protein CcmI	WP_011192696.1	
yeps_YPTB2642			YPTB_RS14320	YPTB2642		cytochrome c-type biogenesis protein CcmH	WP_002214855.1	
yeps_YPTB2643			YPTB_RS14325	YPTB2643		DsbE family thiol:disulfide interchange protein	WP_002209699.1	
yeps_YPTB2644			YPTB_RS14330	YPTB2644		heme lyase CcmF/NrfE family subunit	WP_011192697.1	
yeps_YPTB2645	ccmE		YPTB_RS14335	YPTB2645		cytochrome c maturation protein CcmE	WP_002209697.1	
yeps_YPTB2647			YPTB_RS14345	YPTB2647		heme ABC transporter permease	WP_011192699.1	
yeps_YPTB2648	ccmB		YPTB_RS14350	YPTB2648		heme exporter protein CcmB	WP_002209694.1	
yeps_YPTB2649	ccmA		YPTB_RS14355	YPTB2649		cytochrome c biogenesis heme-transporting ATPase CcmA	WP_011192700.1	
yeps_YPTB2650			YPTB_RS14360	YPTB2650		hypothetical protein	WP_011192701.1	
yeps_YPTB2651			YPTB_RS14370	YPTB2651		LemA family protein	WP_002209691.1	
yeps_YPTB2654			YPTB_RS14385	YPTB2654		hypothetical protein	WP_002209687.1	
yeps_YPTB2655			YPTB_RS14390	YPTB2655		type VI secretion system baseplate subunit TssE	WP_002209686.1	
yeps_YPTB2661	tssL		YPTB_RS14420	YPTB2661		type VI secretion system protein TssL, long form	WP_011192706.1	
yeps_YPTB2662	tssK		YPTB_RS14425	YPTB2662		type VI secretion system baseplate subunit TssK	WP_002211571.1	
yeps_YPTB2673	tssG		YPTB_RS14485	YPTB2673		type VI secretion system baseplate subunit TssG	WP_011192712.1	
yeps_YPTB2674			YPTB_RS14490	YPTB2674		ImpA family type VI secretion system protein	WP_011192713.1	
yeps_YPTB2675			YPTB_RS14495	YPTB2675		fimbrial protein	WP_011192714.1	
yeps_YPTB2676			YPTB_RS14500	YPTB2676		glycosyl transferase	WP_011192715.1	
yeps_YPTB2678			YPTB_RS14510	YPTB2678		OmpA family protein	WP_011192717.1	
yeps_YPTB2679			YPTB_RS14515	YPTB2679		DcrB-related protein	WP_002215321.1	
yeps_YPTB2689	dmsB		YPTB_RS14565	YPTB2689		dimethylsulfoxide reductase subunit B	WP_002211603.1	
yeps_YPTB2693			YPTB_RS14585	YPTB2693		DUF799 domain-containing protein	WP_002211607.1	
yeps_YPTB2697			YPTB_RS14605	YPTB2697		cytochrome b	WP_002211611.1	
yeps_YPTB2698	alaC		YPTB_RS14610	YPTB2698		alanine transaminase	WP_002231034.1	
yeps_YPTB2700	glk		YPTB_RS14620	YPTB2700		glucokinase	WP_002211615.1	
yeps_YPTB2701			YPTB_RS14625	YPTB2701		multidrug/biocide efflux PACE transporter	WP_011192730.1	
yeps_YPTB2703			YPTB_RS14635	YPTB2703		aldo/keto reductase	WP_011192731.1	
yeps_YPTB2704			YPTB_RS14640	YPTB2704		DUF2502 domain-containing protein	WP_002216909.1	
yeps_YPTB2711	ligA	dnaL,lig,lop,pdeC	YPTB_RS14705	YPTB2711		NAD-dependent DNA ligase LigA	WP_011192735.1	
yeps_YPTB2713	cysZ		YPTB_RS14715	YPTB2713		sulfate transporter CysZ	WP_002231042.1	
yeps_YPTB2717	crr	gsr,iex,tgs,treD	YPTB_RS14735	YPTB2717		PTS glucose transporter subunit IIA	WP_002208491.1	
yeps_YPTB2718			YPTB_RS14740	YPTB2718		HAMP domain-containing protein	WP_011192737.1	
yeps_YPTB2719			YPTB_RS14745	YPTB2719		response regulator transcription factor	WP_002208494.1	
yeps_YPTB2721			YPTB_RS14755	YPTB2721		MacB family efflux pump subunit	WP_011192738.1	
yeps_YPTB2737			YPTB_RS14835	YPTB2737		MurR/RpiR family transcriptional regulator	WP_011192747.1	
yeps_YPTB2739			YPTB_RS14850	YPTB2739		N-acetylmannosamine kinase	WP_002208516.1	
yeps_YPTB2741			YPTB_RS14860	YPTB2741		N-acetylmannosamine-6-phosphate 2-epimerase	WP_071819112.1	
yeps_YPTB2742			YPTB_RS14865	YPTB2742		dihydrodipicolinate synthase family protein	WP_002208518.1	
yeps_YPTB2743			YPTB_RS14870	YPTB2743		Dyp-type peroxidase	WP_011192751.1	
yeps_YPTB2744			YPTB_RS14875	YPTB2744		RpoE-regulated lipoprotein	WP_002208521.1	
yeps_YPTB2755			YPTB_RS14935	YPTB2755		YaiI/YqxD family protein	WP_002208527.1	
yeps_YPTB2756	maeB		YPTB_RS14940	YPTB2756		NADP-dependent oxaloacetate-decarboxylating malate dehydrogenase	WP_011192757.1	
yeps_YPTB2757	nudK		YPTB_RS14945	YPTB2757		GDP-mannose pyrophosphatase NudK	WP_002208529.1	
yeps_YPTB2758	napC		YPTB_RS14950	YPTB2758		cytochrome c-type protein NapC	WP_002208531.1	
yeps_YPTB2759	napB		YPTB_RS14955	YPTB2759		nitrate reductase cytochrome c-type subunit	WP_002214527.1	
yeps_YPTB2764			YPTB_RS14980	YPTB2764		helix-turn-helix domain-containing protein	WP_002208538.1	
yeps_YPTB2765	acrD		YPTB_RS14985	YPTB2765		multidrug efflux RND transporter permease AcrD	WP_011192758.1	
yeps_YPTB2766			YPTB_RS14990	YPTB2766		hypothetical protein	WP_002208540.1	
yeps_YPTB2773			YPTB_RS15025	YPTB2773		tetratricopeptide repeat protein	WP_002208547.1	
yeps_YPTB2774			YPTB_RS15030	YPTB2774		ArsC family reductase	WP_002208548.1	
yeps_YPTB2776			YPTB_RS15040	YPTB2776		M15 family metallopeptidase	WP_002208550.1	
yeps_YPTB2777			YPTB_RS15045	YPTB2777		YpfN family protein	WP_002208551.1	
yeps_YPTB2782	bamC		YPTB_RS15070	YPTB2782		outer membrane protein assembly factor BamC	WP_011192764.1	
yeps_YPTB2783	dapA		YPTB_RS15075	YPTB2783		4-hydroxy-tetrahydrodipicolinate synthase	WP_032466692.1	
yeps_YPTB2784			YPTB_RS15080	YPTB2784		glycine cleavage system transcriptional repressor	WP_002227068.1	
yeps_YPTB2786				YPTB2786		hypothetical protein	CAH22024.1	
yeps_YPTB2791	arsC		YPTB_RS15115	YPTB2791		arsenate reductase (glutaredoxin)	WP_002208565.1	
yeps_YPTB2792	hda		YPTB_RS15120	YPTB2792		DnaA inactivator Hda	WP_002228401.1	
yeps_YPTB2793	uraA		YPTB_RS15125	YPTB2793		uracil permease	WP_011192769.1	
yeps_YPTB2795	purM		YPTB_RS15135	YPTB2795		phosphoribosylformylglycinamidine cyclo-ligase	WP_011192770.1	
yeps_YPTB2796	purN		YPTB_RS15140	YPTB2796		phosphoribosylglycinamide formyltransferase	WP_011192771.1	
yeps_YPTB2800	pstA	phoT	YPTB_RS15160	YPTB2800		phosphate ABC transporter permease PstA	WP_011192773.1	
yeps_YPTB2801			YPTB_RS15165	YPTB2801		ABC transporter permease subunit	WP_011192774.1	
yeps_YPTB2802	ppk1		YPTB_RS15170	YPTB2802		polyphosphate kinase 1	WP_002209782.1	
yeps_YPTB2803	ppx		YPTB_RS15175	YPTB2803		exopolyphosphatase	WP_002209783.1	
yeps_YPTB2804			YPTB_RS15180	YPTB2804		DUF2633 family protein	WP_012303868.1	
yeps_YPTB2805	mgtE		YPTB_RS15185	YPTB2805		magnesium transporter	WP_002209784.1	
yeps_YPTB2808			YPTB_RS15200	YPTB2808		ABC transporter permease	WP_011192776.1	
yeps_YPTB2812			YPTB_RS15220	YPTB2812		ABC transporter ATP-binding protein	WP_011192780.1	
yeps_YPTB2814			YPTB_RS15230	YPTB2814		MdtB/MuxB family multidrug efflux RND transporter permease subunit	WP_011192782.1	
yeps_YPTB2815	mdtC		YPTB_RS15235	YPTB2815		multidrug efflux RND transporter permease subunit MdtC	WP_011192783.1	
yeps_YPTB2816			YPTB_RS15240	YPTB2816		MFS transporter	WP_011192784.1	
yeps_YPTB2818	baeR		YPTB_RS15250	YPTB2818		two-component system response regulator BaeR	WP_011192786.1	
yeps_YPTB2821	yegS		YPTB_RS15265	YPTB2821		lipid kinase YegS	WP_011192787.1	
yeps_YPTB2822			YPTB_RS15270	YPTB2822		inhibitor of vertebrate lysozyme family protein	WP_002209800.1	
yeps_YPTB2824	thiD		YPTB_RS15280	YPTB2824		bifunctional hydroxymethylpyrimidine kinase/phosphomethylpyrimidine kinase	WP_011192789.1	
yeps_YPTB2833	guaB	guaR	YPTB_RS15335	YPTB2833		IMP dehydrogenase	WP_002227862.1	
yeps_YPTB2835			YPTB_RS15345	YPTB2835		zinc ribbon domain-containing protein	WP_011192798.1	
yeps_YPTB2839			YPTB_RS15365	YPTB2839		YfgM family protein	WP_002209815.1	
yeps_YPTB2840	hisS		YPTB_RS15370	YPTB2840		histidine--tRNA ligase	WP_002209816.1	
yeps_YPTB2841	ispG		YPTB_RS15375	YPTB2841		flavodoxin-dependent (E)-4-hydroxy-3-methylbut-2-enyl-diphosphate synthase	WP_002209817.1	
yeps_YPTB2845	ndk		YPTB_RS15395	YPTB2845		nucleoside-diphosphate kinase	WP_011192804.1	
yeps_YPTB2852	pepB		YPTB_RS15430	YPTB2852		aminopeptidase PepB	WP_011192810.1	
yeps_YPTB2856	hscB		YPTB_RS15450	YPTB2856		co-chaperone HscB	WP_011192812.1	
yeps_YPTB2858	iscU		YPTB_RS15460	YPTB2858		Fe-S cluster assembly scaffold IscU	WP_011192813.1	
yeps_YPTB2860	iscR		YPTB_RS15470	YPTB2860		Fe-S cluster assembly transcriptional regulator IscR	WP_002222202.1	
yeps_YPTB2862	suhB		YPTB_RS15480	YPTB2862		inositol-1-monophosphatase	WP_002213305.1	
yeps_YPTB2863			YPTB_RS15485	YPTB2863		nickel/cobalt transporter	WP_002213308.1	
yeps_YPTB2864			YPTB_RS15490	YPTB2864		DUF1007 family protein	WP_011192815.1	
yeps_YPTB2870	hmpA		YPTB_RS15520	YPTB2870		NO-inducible flavohemoprotein	WP_002211553.1	
yeps_YPTB2874	glrR		YPTB_RS15540	YPTB2874		two-component system response regulator GlrR	WP_002211556.1	
yeps_YPTB2880	mltF		YPTB_RS15570	YPTB2880		membrane-bound lytic murein transglycosylase MltF	WP_024062994.1	
yeps_YPTB2882	yfhb		YPTB_RS15580	YPTB2882		phosphatidylglycerophosphatase C	WP_002211564.1	
yeps_YPTB2885	fdx			YPTB2885		putative [4Fe-4S] ferredoxin	CAH22123.1	
yeps_YPTB2888	recO		YPTB_RS15610	YPTB2888		DNA repair protein RecO	WP_002209680.1	
yeps_YPTB2891	lepB		YPTB_RS15625	YPTB2891		signal peptidase I	WP_011192827.1	
yeps_YPTB2892	lepA		YPTB_RS15630	YPTB2892		translation elongation factor 4	WP_002209677.1	
yeps_YPTB2896	rseA	mclA	YPTB_RS15650	YPTB2896		anti-sigma-E factor RseA	WP_002209673.1	
yeps_YPTB2901			YPTB_RS15675	YPTB2901		ankyrin repeat domain-containing protein	WP_002209667.1	
yeps_YPTB2903	grcA		YPTB_RS15690	YPTB2903		autonomous glycyl radical cofactor GrcA	WP_002209664.1	
yeps_YPTB2904	ung		YPTB_RS15695	YPTB2904		uracil-DNA glycosylase	WP_002209663.1	
yeps_YPTB2906			YPTB_RS15705	YPTB2906		DUF979 domain-containing protein	WP_011192829.1	
yeps_YPTB2908	pxpA		YPTB_RS15715	YPTB2908		5-oxoprolinase subunit PxpA	WP_002209659.1	
yeps_YPTB2910	pxpB		YPTB_RS15725	YPTB2910		5-oxoprolinase subunit PxpB	WP_011192830.1	
yeps_YPTB2911			YPTB_RS15730	YPTB2911		type 2 GTP cyclohydrolase I	WP_025471060.1	
yeps_YPTB2912			YPTB_RS15735	YPTB2912		3',5'-cyclic-nucleotide phosphodiesterase	WP_002209656.1	
yeps_YPTB2913	phrB	phr	YPTB_RS15740	YPTB2913		deoxyribodipyrimidine photo-lyase	WP_011192831.1	
yeps_YPTB2917	kdpB		YPTB_RS15770	YPTB2917		potassium-transporting ATPase subunit KdpB	WP_011192833.1	
yeps_YPTB2918	kdpC		YPTB_RS15775	YPTB2918		potassium-transporting ATPase subunit KdpC	WP_072083175.1	
yeps_YPTB2922			YPTB_RS15795	YPTB2922		hypothetical protein	WP_002209646.1	
yeps_YPTB2923	pgm		YPTB_RS15805	YPTB2923		phosphoglucomutase (alpha-D-glucose-1,6-bisphosphate-dependent)	WP_041175466.1	
yeps_YPTB2924	seqA		YPTB_RS15810	YPTB2924		replication initiation negative regulator SeqA	WP_011192836.1	
yeps_YPTB2925			YPTB_RS15820	YPTB2925		hypothetical protein	WP_002212246.1	
yeps_YPTB2926				YPTB2926		hypothetical protein	CAH22164.1	
yeps_YPTB2927	chbG		YPTB_RS15830	YPTB2927		chitin disaccharide deacetylase	WP_002212244.1	
yeps_YPTB2928	chbR		YPTB_RS15835	YPTB2928		transcriptional regulator ChbR	WP_002212243.1	
yeps_YPTB2936			YPTB_RS15885	YPTB2936		HoxN/HupN/NixA family nickel/cobalt transporter	WP_011192840.1	
yeps_YPTB2937	yut		YPTB_RS15890	YPTB2937		urea transporter	WP_011192841.1	
yeps_YPTB2938			YPTB_RS15895	YPTB2938		urease accessory protein UreD	WP_011192842.1	
yeps_YPTB2943			YPTB_RS15920	YPTB2943		urease subunit beta	WP_002222230.1	
yeps_YPTB2964	dnaQ	mutD	YPTB_RS16030	YPTB2964		DNA polymerase III subunit epsilon	WP_002210700.1	
yeps_YPTB2969			YPTB_RS16055	YPTB2969		endonuclease/exonuclease/phosphatase family protein	WP_002220059.1	
yeps_YPTB2976	rcsF		YPTB_RS16120	YPTB2976		Rcs stress response system protein RcsF	WP_002212158.1	
yeps_YPTB2979	nlpE		YPTB_RS16135	YPTB2979		envelope stress response activation lipoprotein NlpE	WP_002212155.1	
yeps_YPTB2980	arfB		YPTB_RS16140	YPTB2980		aminoacyl-tRNA hydrolase	WP_002212154.1	
yeps_YPTB2981			YPTB_RS16145	YPTB2981		YaeQ family protein	WP_002212153.1	
yeps_YPTB2983	rof		YPTB_RS16155	YPTB2983		Rho-binding antiterminator	WP_002218374.1	
yeps_YPTB2984			YPTB_RS16160	YPTB2984		cytochrome c	WP_002212150.1	
yeps_YPTB2985	tilS		YPTB_RS16165	YPTB2985		tRNA lysidine(34) synthetase TilS	WP_011192857.1	
yeps_YPTB2986			YPTB_RS16170	YPTB2986		VOC family protein	WP_002212148.1	
yeps_YPTB2988	dnaE	polC	YPTB_RS16180	YPTB2988		DNA polymerase III subunit alpha	WP_012104742.1	
yeps_YPTB2989	rnhB		YPTB_RS16185	YPTB2989		ribonuclease HII	WP_002212145.1	
yeps_YPTB2991	lpxA		YPTB_RS16195	YPTB2991		acyl-ACP--UDP-N-acetylglucosamine O-acyltransferase	WP_002212143.1	
yeps_YPTB2995	bamA		YPTB_RS16215	YPTB2995		outer membrane protein assembly factor BamA	WP_002212139.1	
yeps_YPTB2996	rseP		YPTB_RS16220	YPTB2996		sigma E protease regulator RseP	WP_002212138.1	
yeps_YPTB2999	ispC		YPTB_RS16235	YPTB2999		1-deoxy-D-xylulose-5-phosphate reductoisomerase	WP_011192861.1	
yeps_YPTB3000	frr	rrf	YPTB_RS16240	YPTB3000		ribosome recycling factor	WP_002212134.1	
yeps_YPTB3001	pyrH	smbA	YPTB_RS16245	YPTB3001		UMP kinase	WP_002212133.1	
yeps_YPTB3002	tsf		YPTB_RS16250	YPTB3002		translation elongation factor Ts	WP_002212132.1	
yeps_YPTB3007			YPTB_RS16275	YPTB3007		DUF3461 family protein	WP_002212127.1	
yeps_YPTB3008			YPTB_RS16280	YPTB3008		flavodoxin	WP_011192865.1	
yeps_YPTB3009	truC		YPTB_RS16285	YPTB3009		tRNA pseudouridine(65) synthase TruC	WP_002212125.1	
yeps_YPTB3016			YPTB_RS16325	YPTB3016		DUF423 domain-containing protein	WP_002212118.1	
yeps_YPTB3019	csdE		YPTB_RS16340	YPTB3019		cysteine desulfurase sulfur acceptor subunit CsdE	WP_002212115.1	

yeps_YPTB3027	recC		YPTB_RS16395	YPTB3027		exodeoxyribonuclease V subunit gamma	WP_011192871.1	
yeps_YPTB3028			YPTB_RS16400	YPTB3028		peptidase	WP_012104731.1	
yeps_YPTB3029			YPTB_RS16405	YPTB3029		YgdB family protein	WP_002211629.1	
yeps_YPTB3030			YPTB_RS16410	YPTB3030		prepilin peptidase-dependent protein	WP_002211630.1	
yeps_YPTB3031			YPTB_RS16415	YPTB3031		prepilin peptidase-dependent protein	WP_002228638.1	
yeps_YPTB3044	galR		YPTB_RS16490	YPTB3044		HTH-type transcriptional regulator GalR	WP_002209845.1	
yeps_YPTB3045	lysA		YPTB_RS16495	YPTB3045		diaminopimelate decarboxylase	WP_011192878.1	
yeps_YPTB3046			YPTB_RS16500	YPTB3046		LysR family transcriptional regulator	WP_011192879.1	
yeps_YPTB3048			YPTB_RS16510	YPTB3048		LysR family transcriptional regulator	WP_011192880.1	
yeps_YPTB3049			YPTB_RS16515	YPTB3049		MBL fold metallo-hydrolase	WP_002209850.1	
yeps_YPTB3076	kbaZ		YPTB_RS16650	YPTB3076		tagatose-bisphosphate aldolase subunit KbaZ	WP_011192897.1	
yeps_YPTB3077			YPTB_RS16655	YPTB3077		SIS domain-containing protein	WP_011192898.1	
yeps_YPTB3078			YPTB_RS16660	YPTB3078		PTS system mannose/fructose/N-acetylgalactosamine-transporter subunit IIB	WP_002209878.1	
yeps_YPTB3079			YPTB_RS16665	YPTB3079		PTS mannose/fructose/sorbose/N-acetylgalactosamine transporter subunit IIC	WP_002209879.1	
yeps_YPTB3080			YPTB_RS16670	YPTB3080		PTS system mannose/fructose/sorbose family transporter subunit IID	WP_002209880.1	
yeps_YPTB3081			YPTB_RS16675	YPTB3081		PTS sugar transporter subunit IIA	WP_002209881.1	
yeps_YPTB3082	nagA		YPTB_RS16680	YPTB3082		N-acetylglucosamine-6-phosphate deacetylase	WP_002209882.1	
yeps_YPTB3083	kduD		YPTB_RS16685	YPTB3083		2-dehydro-3-deoxy-D-gluconate 5-dehydrogenase KduD	WP_002230627.1	
yeps_YPTB3087			YPTB_RS16705	YPTB3087		DUF2264 domain-containing protein	WP_011192901.1	
yeps_YPTB3088			YPTB_RS16710	YPTB3088		tagatose bisphosphate family class II aldolase	WP_011192902.1	
yeps_YPTB3089			YPTB_RS16715	YPTB3089		DJ-1/PfpI family protein	WP_011192903.1	
yeps_YPTB3097			YPTB_RS16755	YPTB3097		beta-galactosidase	WP_002209897.1	
yeps_YPTB3098			YPTB_RS16760	YPTB3098		arabinogalactan endo-beta-1,4-galactanase	WP_002209898.1	
yeps_YPTB3099			YPTB_RS16765	YPTB3099		sugar ABC transporter permease	WP_002209899.1	
yeps_YPTB3100			YPTB_RS16770	YPTB3100		sugar ABC transporter permease	WP_002209900.1	
yeps_YPTB3101			YPTB_RS16775	YPTB3101		extracellular solute-binding protein	WP_002209901.1	
yeps_YPTB3103			YPTB_RS16785	YPTB3103		sugar ABC transporter ATP-binding protein	WP_011192908.1	
yeps_YPTB3117			YPTB_RS16865	YPTB3117		Fic family protein	WP_002209918.1	
yeps_YPTB3118			YPTB_RS16870	YPTB3118		hypothetical protein	WP_011192915.1	
yeps_YPTB3166	dsbC		YPTB_RS17155	YPTB3166		bifunctional protein-disulfide isomerase/oxidoreductase DsbC	WP_002209932.1	
yeps_YPTB3168	fldB		YPTB_RS17165	YPTB3168		flavodoxin FldB	WP_011192956.1	
yeps_YPTB3171	creB	blrA	YPTB_RS17180	YPTB3171		two-component system response regulator CreB	WP_002209937.1	
yeps_YPTB3172			YPTB_RS17185	YPTB3172		protein YgfX	WP_002215144.1	
yeps_YPTB3173	sdhE		YPTB_RS17190	YPTB3173		FAD assembly factor SdhE	WP_002209939.1	
yeps_YPTB3174	ygfZ		YPTB_RS17195	YPTB3174		tRNA-modifying protein YgfZ	WP_011192958.1	
yeps_YPTB3175			YPTB_RS17200	YPTB3175		DUF2165 family protein	WP_002209941.1	
yeps_YPTB3176			YPTB_RS17205	YPTB3176		hemolysin III family protein	WP_011192959.1	
yeps_YPTB3184	ubiH		YPTB_RS17250	YPTB3184		2-octaprenyl-6-methoxyphenyl hydroxylase	WP_011192963.1	
yeps_YPTB3185	pepP		YPTB_RS17255	YPTB3185		Xaa-Pro aminopeptidase	WP_011192964.1	
yeps_YPTB3186			YPTB_RS17260	YPTB3186		YecA family protein	WP_002209953.1	
yeps_YPTB3187	zapA		YPTB_RS17265	YPTB3187		cell division protein ZapA	WP_002209954.1	
yeps_YPTB3190	rpiA		YPTB_RS17280	YPTB3190		ribose-5-phosphate isomerase RpiA	WP_002209957.1	
yeps_YPTB3191			YPTB_RS17285	YPTB3191		LysR family transcriptional regulator ArgP	WP_002209958.1	
yeps_YPTB3192			YPTB_RS17290	YPTB3192		oxidative stress defense protein	WP_011192966.1	
yeps_YPTB3203	metK		YPTB_RS17350	YPTB3203		methionine adenosyltransferase	WP_002209971.1	
yeps_YPTB3206	rsmE		YPTB_RS17370	YPTB3206		16S rRNA (uracil(1498)-N(3))-methyltransferase	WP_002209975.1	
yeps_YPTB3207	gshB	gsh-II	YPTB_RS17375	YPTB3207		glutathione synthase	WP_002209976.1	
yeps_YPTB3208			YPTB_RS17380	YPTB3208		YqgE/AlgH family protein	WP_011192971.1	
yeps_YPTB3209	ruvX		YPTB_RS17385	YPTB3209		Holliday junction resolvase RuvX	WP_011192972.1	
yeps_YPTB3211	aguA		YPTB_RS17395	YPTB3211		agmatine deiminase	WP_002209980.1	
yeps_YPTB3212			YPTB_RS17400	YPTB3212		type IV pilus twitching motility protein PilT	WP_002209981.1	
yeps_YPTB3213			YPTB_RS17405	YPTB3213		YggS family pyridoxal phosphate-dependent enzyme	WP_011192973.1	
yeps_YPTB3214			YPTB_RS17410	YPTB3214		pyrroline-5-carboxylate reductase	WP_011192974.1	
yeps_YPTB3216			YPTB_RS17420	YPTB3216		DUF167 family protein YggU	WP_011192976.1	
yeps_YPTB3221	glsB		YPTB_RS17445	YPTB3221		glutaminase B	WP_011192980.1	
yeps_YPTB3223	trmB		YPTB_RS17455	YPTB3223		tRNA (guanosine(46)-N7)-methyltransferase TrmB	WP_011192981.1	
yeps_YPTB3231			YPTB_RS17500	YPTB3231		substrate-binding domain-containing protein	WP_011192985.1	
yeps_YPTB3258			YPTB_RS17640	YPTB3258		acyl-homoserine-lactone synthase	WP_002215903.1	
yeps_YPTB3261			YPTB_RS17655	YPTB3261		intradiol ring-cleavage dioxygenase	WP_011192997.1	
yeps_YPTB3262			YPTB_RS17660	YPTB3262		MFS transporter	WP_011192998.1	
yeps_YPTB3263			YPTB_RS17665	YPTB3263		aerobactin synthase IucA	WP_011192999.1	
yeps_YPTB3264			YPTB_RS17670	YPTB3264		acetyltransferase	WP_011193000.1	
yeps_YPTB3265	iucC		YPTB_RS17675	YPTB3265		IucA/IucC family siderophore biosynthesis protein	WP_011193001.1	
yeps_YPTB3266			YPTB_RS17680	YPTB3266		lysine N(6)-hydroxylase/L-ornithine N(5)-oxygenase family protein	WP_011193002.1	
yeps_YPTB3267			YPTB_RS17685	YPTB3267		TonB-dependent siderophore receptor	WP_011193003.1	
yeps_YPTB3269			YPTB_RS17695	YPTB3269		cellulase family glycosylhydrolase	WP_012303626.1	
yeps_YPTB3270			YPTB_RS17700	YPTB3270		LacI family transcriptional regulator	WP_032465892.1	
yeps_YPTB3290			YPTB_RS17800	YPTB3290		ABC transporter ATP-binding protein/permease	WP_011193020.1	
yeps_YPTB3291			YPTB_RS17805	YPTB3291		ABC transporter ATP-binding protein/permease	WP_011193021.1	
yeps_YPTB3294			YPTB_RS17820	YPTB3294		Gfo/Idh/MocA family oxidoreductase	WP_011193024.1	
yeps_YPTB3295			YPTB_RS17825	YPTB3295		NAD-dependent epimerase/dehydratase family protein	WP_011193025.1	
yeps_YPTB3355			YPTB_RS18135	YPTB3355		flagellar biosynthetic protein FliR	WP_002213766.1	
yeps_YPTB3382	exbD		YPTB_RS18275	YPTB3382		TonB system transport protein ExbD	WP_012414002.1	
yeps_YPTB3384	metC		YPTB_RS18285	YPTB3384		cystathionine beta-lyase	WP_002212172.1	
yeps_YPTB3385			YPTB_RS18290	YPTB3385		DedA family protein	WP_002212173.1	
yeps_YPTB3386			YPTB_RS18295	YPTB3386		AraC family transcriptional regulator	WP_002213786.1	
yeps_YPTB3387	yqhD		YPTB_RS18300	YPTB3387		alcohol dehydrogenase	WP_002212174.1	
yeps_YPTB3388	dkgA		YPTB_RS18310	YPTB3388		2,5-didehydrogluconate reductase DkgA	WP_011193092.1	
yeps_YPTB3390	ftsP		YPTB_RS18320	YPTB3390		cell division protein FtsP	WP_002212176.1	
yeps_YPTB3391			YPTB_RS18325	YPTB3391		1-acylglycerol-3-phosphate O-acyltransferase	WP_002212177.1	
yeps_YPTB3392	parC		YPTB_RS18330	YPTB3392		DNA topoisomerase IV subunit A	WP_002212178.1	
yeps_YPTB3393			YPTB_RS18340	YPTB3393		NAD(P)H-dependent oxidoreductase	WP_002212179.1	
yeps_YPTB3394			YPTB_RS18345	YPTB3394		LysR family transcriptional regulator	WP_011193094.1	
yeps_YPTB3399	nudF		YPTB_RS18370	YPTB3399		ADP-ribose diphosphatase	WP_002212184.1	
yeps_YPTB3402			YPTB_RS18390	YPTB3402		glutathionylspermidine synthase family protein	WP_002212188.1	
yeps_YPTB3403	ygiD		YPTB_RS18395	YPTB3403		4,5-DOPA dioxygenase extradiol	WP_002212189.1	
yeps_YPTB3404	ribB	htrP	YPTB_RS18400	YPTB3404		3,4-dihydroxy-2-butanone-4-phosphate synthase	WP_002212190.1	
yeps_YPTB3408	glnE		YPTB_RS18425	YPTB3408		bifunctional [glutamate--ammonia ligase]-adenylyl-L-tyrosine phosphorylase/[glutamate--ammonia-ligase] adenylyltransferase	WP_002212194.1	
yeps_YPTB3409			YPTB_RS18430	YPTB3409		inorganic triphosphatase	WP_002212195.1	
yeps_YPTB3410			YPTB_RS18435	YPTB3410		SH3 domain-containing protein	WP_011193100.1	
yeps_YPTB3411			YPTB_RS18440	YPTB3411		multifunctional CCA addition/repair protein	WP_011193101.1	
yeps_YPTB3412	bacA		YPTB_RS18445	YPTB3412		undecaprenyl-diphosphate phosphatase	WP_002212198.1	
yeps_YPTB3414	plsY		YPTB_RS18455	YPTB3414		glycerol-3-phosphate 1-O-acyltransferase PlsY	WP_002217581.1	
yeps_YPTB3415	tsaD		YPTB_RS18460	YPTB3415		tRNA (adenosine(37)-N6)-threonylcarbamoyltransferase complex transferase subunit TsaD	WP_002212201.1	
yeps_YPTB3417	dnaG	dnaP,parB	YPTB_RS18470	YPTB3417		DNA primase	WP_002210358.1	
yeps_YPTB3434			YPTB_RS18585	YPTB3434		hypothetical protein	WP_002210374.1	
yeps_YPTB3439			YPTB_RS18610	YPTB3439		glycoside hydrolase family 3 C-terminal domain-containing protein	WP_011193110.1	
yeps_YPTB3440			YPTB_RS18615	YPTB3440		hypothetical protein	WP_011193111.1	
yeps_YPTB3441			YPTB_RS18620	YPTB3441		carbohydrate ABC transporter permease	WP_011193112.1	
yeps_YPTB3442			YPTB_RS18625	YPTB3442		sugar ABC transporter permease	WP_011193113.1	
yeps_YPTB3443			YPTB_RS18630	YPTB3443		extracellular solute-binding protein	WP_011193114.1	
yeps_YPTB3444			YPTB_RS18635	YPTB3444		LacI family transcriptional regulator	WP_011193115.1	
yeps_YPTB3445			YPTB_RS18640	YPTB3445		hypothetical protein	WP_011193116.1	
yeps_YPTB3446			YPTB_RS18645	YPTB3446		ABC transporter ATP-binding protein	WP_002353714.1	
yeps_YPTB3447			YPTB_RS18650	YPTB3447		membrane protein	WP_011193117.1	
yeps_YPTB3448			YPTB_RS18655	YPTB3448		transporter	WP_011193118.1	
yeps_YPTB3468			YPTB_RS18775	YPTB3468		HdeD family acid-resistance protein	WP_002210400.1	
yeps_YPTB3469			YPTB_RS18780	YPTB3469		NADPH-dependent 2,4-dienoyl-CoA reductase	WP_011193129.1	
yeps_YPTB3472			YPTB_RS18795	YPTB3472		M48 family metallopeptidase	WP_002210404.1	
yeps_YPTB3473			YPTB_RS18800	YPTB3473		Gfo/Idh/MocA family oxidoreductase	WP_002210405.1	
yeps_YPTB3475			YPTB_RS18810	YPTB3475		YgjV family protein	WP_002210407.1	
yeps_YPTB3477			YPTB_RS18820	YPTB3477		tagaturonate reductase	WP_011193132.1	
yeps_YPTB3480	exuR		YPTB_RS18840	YPTB3480		transcriptional regulator ExuR	WP_002210412.1	
yeps_YPTB3481			YPTB_RS18845	YPTB3481		hypothetical protein	WP_012303498.1	
yeps_YPTB3482			YPTB_RS18850	YPTB3482		DedA family protein	WP_002210414.1	
yeps_YPTB3483	mzrA		YPTB_RS18855	YPTB3483		EnvZ/OmpR regulon moderator MzrA	WP_002210415.1	
yeps_YPTB3484			YPTB_RS18860	YPTB3484		DUF1090 domain-containing protein	WP_002210416.1	
yeps_YPTB3486			YPTB_RS18875	YPTB3486		phage holin family protein	WP_002210419.1	
yeps_YPTB3487			YPTB_RS18880	YPTB3487		YqjK-like family protein	WP_011193134.1	
yeps_YPTB3489			YPTB_RS18890	YPTB3489		glutathione S-transferase family protein	WP_011193135.1	
yeps_YPTB3490			YPTB_RS18895	YPTB3490		LysR family transcriptional regulator	WP_011193136.1	
yeps_YPTB3494			YPTB_RS18915	YPTB3494		YraN family protein	WP_002210147.1	
yeps_YPTB3495	diaA		YPTB_RS18920	YPTB3495		DnaA initiator-associating protein DiaA	WP_011193139.1	
yeps_YPTB3496	yraP		YPTB_RS18925	YPTB3496		divisome-associated lipoprotein YraP	WP_011193140.1	
yeps_YPTB3497	mtgA		YPTB_RS18930	YPTB3497		monofunctional biosynthetic peptidoglycan transglycosylase	WP_011193141.1	
yeps_YPTB3499				YPTB3499		hypothetical protein	CAH22737.1	
yeps_YPTB3500	arcB		YPTB_RS18945	YPTB3500		aerobic respiration two-component sensor histidine kinase ArcB	WP_002210142.1	
yeps_YPTB3501			YPTB_RS18955	YPTB3501		TIGR01212 family radical SAM protein	WP_002210140.1	
yeps_YPTB3505	sspB		YPTB_RS18975	YPTB3505		ClpXP protease specificity-enhancing factor	WP_002216203.1	
yeps_YPTB3509			YPTB_RS19000	YPTB3509		cell division protein ZapE	WP_002210131.1	
yeps_YPTB3511	degQ	hhoA	YPTB_RS19010	YPTB3511		serine endoprotease DegQ	WP_002218066.1	
yeps_YPTB3512	degS	hhoB,htrH	YPTB_RS19015	YPTB3512		outer membrane-stress sensor serine endopeptidase DegS	WP_002210128.1	
yeps_YPTB3513	murA	murZ	YPTB_RS19020	YPTB3513		UDP-N-acetylglucosamine 1-carboxyvinyltransferase	WP_002210127.1	
yeps_YPTB3514	ibaG		YPTB_RS19025	YPTB3514		BolA family iron metabolism protein IbaG	WP_012104414.1	
yeps_YPTB3516	mlaC		YPTB_RS19035	YPTB3516		phospholipid-binding protein MlaC	WP_002210124.1	
yeps_YPTB3517	mlaD		YPTB_RS19040	YPTB3517		outer membrane lipid asymmetry maintenance protein MlaD	WP_011193146.1	
yeps_YPTB3519	mlaF		YPTB_RS19050	YPTB3519		phospholipid ABC transporter ATP-binding protein MlaF	WP_002210121.1	
yeps_YPTB3520			YPTB_RS19055	YPTB3520		calcium/sodium antiporter	WP_002210120.1	
yeps_YPTB3521	kdsD		YPTB_RS19060	YPTB3521		arabinose-5-phosphate isomerase KdsD	WP_002210119.1	
yeps_YPTB3522	kdsC		YPTB_RS19065	YPTB3522		3-deoxy-manno-octulosonate-8-phosphatase KdsC	WP_002228203.1	
yeps_YPTB3529	rapZ		YPTB_RS19100	YPTB3529		RNase adapter RapZ	WP_002210113.1	
yeps_YPTB3530	npr		YPTB_RS19105	YPTB3530		PTS phosphocarrier protein NPr	WP_002210112.1	
yeps_YPTB3532			YPTB_RS19115	YPTB3532		aspartate carbamoyltransferase regulatory subunit	WP_012104412.1	
yeps_YPTB3541	pmbA	tldE	YPTB_RS19160	YPTB3541		metalloprotease PmbA	WP_011193152.1	
yeps_YPTB3542			YPTB_RS19165	YPTB3542		ribosome-associated protein	WP_011193153.1	
yeps_YPTB3543			YPTB_RS19170	YPTB3543		ribonuclease inhibitor	WP_002210098.1	
yeps_YPTB3544			YPTB_RS19175	YPTB3544		ribonuclease Ba	WP_002214288.1	
yeps_YPTB3546	aaeB		YPTB_RS19185	YPTB3546		p-hydroxybenzoic acid efflux pump subunit AaeB	WP_002210095.1	
yeps_YPTB3566	csrD		YPTB_RS19285	YPTB3566		RNase E specificity factor CsrD	WP_011193164.1	
yeps_YPTB3567			YPTB_RS19290	YPTB3567		oxidoreductase	WP_002210074.1	
yeps_YPTB3571	accB	fabE	YPTB_RS19310	YPTB3571		acetyl-CoA carboxylase biotin carboxyl carrier protein	WP_002230506.1	

yeps_YPTB3579			YPTB_RS19350	YPTB3579		GntR family transcriptional regulator	WP_011193167.1	
yeps_YPTB3580			YPTB_RS19355	YPTB3580		MFS transporter	WP_011193168.1	
yeps_YPTB3581			YPTB_RS19360	YPTB3581		carboxymuconolactone decarboxylase family protein	WP_011193169.1	
yeps_YPTB3582			YPTB_RS19365	YPTB3582		NAD(P)-dependent oxidoreductase	WP_011193170.1	
yeps_YPTB3589			YPTB_RS19415	YPTB3589		esterase-like activity of phytase family protein	WP_011193175.1	
yeps_YPTB3590	yghU		YPTB_RS19420	YPTB3590		glutathione-dependent disulfide-bond oxidoreductase	WP_032466981.1	
yeps_YPTB3591			YPTB_RS19425	YPTB3591		SIS domain-containing protein	WP_011193177.1	
yeps_YPTB3592			YPTB_RS19430	YPTB3592		FGGY-family carbohydrate kinase	WP_002210047.1	
yeps_YPTB3593			YPTB_RS19435	YPTB3593		ABC transporter permease	WP_011193178.1	
yeps_YPTB3595			YPTB_RS19445	YPTB3595		sugar ABC transporter ATP-binding protein	WP_002210044.1	
yeps_YPTB3596			YPTB_RS19450	YPTB3596		autoinducer 2 ABC transporter substrate-binding protein	WP_002210043.1	
yeps_YPTB3597	lpxP		YPTB_RS19455	YPTB3597		kdo(2)-lipid IV(A) palmitoleoyltransferase	WP_002210042.1	
yeps_YPTB3598			YPTB_RS19460	YPTB3598		hypothetical protein	WP_011193180.1	
yeps_YPTB3599			YPTB_RS19465	YPTB3599		Na+/H+ antiporter	WP_002215816.1	
yeps_YPTB3603	ssuC		YPTB_RS19485	YPTB3603		aliphatic sulfonate ABC transporter permease SsuC	WP_002213853.1	
yeps_YPTB3604	ssuD		YPTB_RS19490	YPTB3604		FMNH2-dependent alkanesulfonate monooxygenase	WP_011193183.1	
yeps_YPTB3607	deoC	dra	YPTB_RS19505	YPTB3607		deoxyribose-phosphate aldolase	WP_011193185.1	
yeps_YPTB3608			YPTB_RS19510	YPTB3608		ribokinase	WP_011193186.1	
yeps_YPTB3609	rbsD		YPTB_RS19515	YPTB3609		D-ribose pyranase	WP_011193187.1	
yeps_YPTB3610			YPTB_RS19520	YPTB3610		helix-turn-helix domain-containing protein	WP_012303467.1	
yeps_YPTB3611			YPTB_RS19525	YPTB3611		Gfo/Idh/MocA family oxidoreductase	WP_002210025.1	
yeps_YPTB3626	tssA		YPTB_RS19610	YPTB3626		type VI secretion system protein TssA	WP_002212110.1	
yeps_YPTB3628			YPTB_RS19620	YPTB3628		Fis family transcriptional regulator	WP_002212108.1	
yeps_YPTB3629	tssH		YPTB_RS19625	YPTB3629		type VI secretion system ATPase TssH	WP_002212107.1	
yeps_YPTB3631	tssK		YPTB_RS19635	YPTB3631		type VI secretion system baseplate subunit TssK	WP_002212105.1	
yeps_YPTB3632	tssJ		YPTB_RS19640	YPTB3632		type VI secretion system lipoprotein TssJ	WP_002212104.1	
yeps_YPTB3633	tagH		YPTB_RS19645	YPTB3633		type VI secretion system-associated FHA domain protein TagH	WP_011193200.1	
yeps_YPTB3635	tssF		YPTB_RS19655	YPTB3635		type VI secretion system baseplate subunit TssF	WP_011193201.1	
yeps_YPTB3636	tssE		YPTB_RS19660	YPTB3636		type VI secretion system baseplate subunit TssE	WP_002212100.1	
yeps_YPTB3637	tssC		YPTB_RS19665	YPTB3637		type VI secretion system contractile sheath large subunit	WP_002230616.1	
yeps_YPTB3638	tssB		YPTB_RS19670	YPTB3638		type VI secretion system contractile sheath small subunit	WP_011193202.1	
yeps_YPTB3639			YPTB_RS19675	YPTB3639		Hcp family type VI secretion system effector	WP_011193203.1	
yeps_YPTB3642			YPTB_RS19690	YPTB3642		maltoporin	WP_002212092.1	
yeps_YPTB3643	malK		YPTB_RS19695	YPTB3643		maltose/maltodextrin ABC transporter ATP-binding protein MalK	WP_002212091.1	
yeps_YPTB3645	malE		YPTB_RS19705	YPTB3645		maltose/maltodextrin ABC transporter substrate-binding protein MalE	WP_002212089.1	
yeps_YPTB3646	malF		YPTB_RS19710	YPTB3646		maltose ABC transporter permease MalF	WP_011193206.1	
yeps_YPTB3647	malG		YPTB_RS19715	YPTB3647		maltose ABC transporter permease MalG	WP_002212087.1	
yeps_YPTB3648	psiE		YPTB_RS19720	YPTB3648		phosphate-starvation-inducible protein PsiE	WP_002212086.1	
yeps_YPTB3650	lysC	apk	YPTB_RS19730	YPTB3650		lysine-sensitive aspartokinase 3	WP_011193207.1	
yeps_YPTB3653	metH		YPTB_RS19750	YPTB3653		methionine synthase	WP_038400771.1	
yeps_YPTB3654	iclR		YPTB_RS19755	YPTB3654		glyoxylate bypass operon transcriptional repressor IclR	WP_002230619.1	
yeps_YPTB3661	tsaC		YPTB_RS19830	YPTB3661		L-threonylcarbamoyladenylate synthase type 1 TsaC	WP_002209025.1	
yeps_YPTB3662			YPTB_RS19835	YPTB3662		topoisomerase DNA-binding C4 zinc finger domain-containing protein	WP_012105865.1	
yeps_YPTB3666	fmt		YPTB_RS19855	YPTB3666		methionyl-tRNA formyltransferase	WP_002209020.1	
yeps_YPTB3669	mscL		YPTB_RS19870	YPTB3669		large-conductance mechanosensitive channel protein MscL	WP_002209017.1	
yeps_YPTB3670			YPTB_RS19875	YPTB3670		alternative ribosome-rescue factor A	WP_002209016.1	
yeps_YPTB3671	zntR		YPTB_RS19880	YPTB3671		Zn(2+)-responsive transcriptional regulator	WP_002215702.1	
yeps_YPTB3672	rplQ		YPTB_RS19885	YPTB3672		50S ribosomal protein L17	WP_002209014.1	
yeps_YPTB3673	rpoA		YPTB_RS19890	YPTB3673		DNA-directed RNA polymerase subunit alpha	WP_002209013.1	
yeps_YPTB3703	fusA	far,fus	YPTB_RS20040	YPTB3703		elongation factor G	WP_002212325.1	
yeps_YPTB3707	tusC		YPTB_RS20060	YPTB3707		sulfurtransferase complex subunit TusC	WP_002212321.1	
yeps_YPTB3708	tusD		YPTB_RS20065	YPTB3708		sulfurtransferase complex subunit TusD	WP_002212320.1	
yeps_YPTB3713			YPTB_RS20090	YPTB3713		YheV family putative metal-binding protein	WP_002212315.1	
yeps_YPTB3715	kefG		YPTB_RS20100	YPTB3715		glutathione-regulated potassium-efflux system ancillary protein KefG	WP_002215966.1	
yeps_YPTB3716			YPTB_RS20105	YPTB3716		ABC transporter ATP-binding protein	WP_002230378.1	
yeps_YPTB3719	tauD	ssiD	YPTB_RS20120	YPTB3719		taurine dioxygenase	WP_002212310.1	
yeps_YPTB3720	tauC	ssiC	YPTB_RS20125	YPTB3720		taurine ABC transporter permease TauC	WP_002212309.1	
yeps_YPTB3721	tauB	ssiB	YPTB_RS20130	YPTB3721		taurine ABC transporter ATP-binding subunit	WP_011193220.1	
yeps_YPTB3723			YPTB_RS20140	YPTB3723		LysE family translocator	WP_011193221.1	
yeps_YPTB3724			YPTB_RS20145	YPTB3724		hydrolase	WP_002212302.1	
yeps_YPTB3726			YPTB_RS20155	YPTB3726		phosphoribulokinase	WP_002212300.1	
yeps_YPTB3727			YPTB_RS20160	YPTB3727		DUF1240 domain-containing protein	WP_002228139.1	
yeps_YPTB3728			YPTB_RS20165	YPTB3728		OsmC family protein	WP_002212298.1	
yeps_YPTB3735			YPTB_RS20200	YPTB3735		6-phospho-beta-glucosidase	WP_011193227.1	
yeps_YPTB3736			YPTB_RS20205	YPTB3736		LacI family transcriptional regulator	WP_002208881.1	
yeps_YPTB3737			YPTB_RS20210	YPTB3737		TonB-dependent copper receptor	WP_011193228.1	
yeps_YPTB3739			YPTB_RS20220	YPTB3739		cytosine deaminase	WP_011193230.1	
yeps_YPTB3740			YPTB_RS20225	YPTB3740		nitrite reductase large subunit NirB	WP_011193231.1	
yeps_YPTB3741	nirD		YPTB_RS20230	YPTB3741		nitrite reductase small subunit NirD	WP_002208887.1	
yeps_YPTB3742	nirC		YPTB_RS20235	YPTB3742		nitrite transporter NirC	WP_002208888.1	
yeps_YPTB3743	cysG			YPTB3743		uroporphyrin-III C-methyltransferase / precorrin-2 oxidase /...	CAH22981.1	
yeps_YPTB3744	trpS		YPTB_RS20245	YPTB3744		tryptophan--tRNA ligase	WP_002208891.1	
yeps_YPTB3745			YPTB_RS20250	YPTB3745		phosphoglycolate phosphatase	WP_011193233.1	
yeps_YPTB3747	dam		YPTB_RS20260	YPTB3747		adenine-specific DNA-methyltransferase	WP_002208895.1	
yeps_YPTB3748			YPTB_RS20265	YPTB3748		SPOR domain-containing protein	WP_011193234.1	
yeps_YPTB3754			YPTB_RS20295	YPTB3754		PilN domain-containing protein	WP_011193236.1	
yeps_YPTB3755	pilM		YPTB_RS20300	YPTB3755		pilus assembly protein PilM	WP_011193237.1	
yeps_YPTB3757	nudE		YPTB_RS20310	YPTB3757		ADP compounds hydrolase NudE	WP_002208907.1	
yeps_YPTB3758			YPTB_RS20320	YPTB3758		intracellular growth attenuator family protein	WP_011193239.1	
yeps_YPTB3759	yrfG		YPTB_RS20325	YPTB3759		GMP/IMP nucleotidase	WP_002208909.1	
yeps_YPTB3761	hslO		YPTB_RS20335	YPTB3761		Hsp33 family molecular chaperone HslO	WP_002208911.1	
yeps_YPTB3765	greB		YPTB_RS20355	YPTB3765		transcription elongation factor GreB	WP_002208915.1	
yeps_YPTB3769			YPTB_RS20380	YPTB3769		ferrous iron transporter C	WP_002208920.1	
yeps_YPTB3770			YPTB_RS20385	YPTB3770		DUF1471 domain-containing protein	WP_002208921.1	
yeps_YPTB3771	bioH		YPTB_RS20390	YPTB3771		pimeloyl-ACP methyl ester esterase BioH	WP_011193243.1	
yeps_YPTB3772	gntX		YPTB_RS20395	YPTB3772		DNA utilization protein GntX	WP_002208923.1	
yeps_YPTB3773	nfuA		YPTB_RS20400	YPTB3773		Fe-S biogenesis protein NfuA	WP_002208924.1	
yeps_YPTB3774	malQ		YPTB_RS23610	YPTB3774		4-alpha-glucanotransferase	WP_002208925.1	
yeps_YPTB3775	malP		YPTB_RS23615	YPTB3775		maltodextrin phosphorylase	WP_011193244.1	
yeps_YPTB3776	malT		YPTB_RS20415	YPTB3776		HTH-type transcriptional regulator MalT	WP_002208926.1	
yeps_YPTB3777	glpE		YPTB_RS20420	YPTB3777		thiosulfate sulfurtransferase GlpE	WP_002218928.1	
yeps_YPTB3778	glpG		YPTB_RS20425	YPTB3778		rhomboid family intramembrane serine protease GlpG	WP_011193245.1	
yeps_YPTB3783	glgP	glgY	YPTB_RS20460	YPTB3783		glycogen phosphorylase	WP_011193248.1	
yeps_YPTB3784	glgA		YPTB_RS20465	YPTB3784		glycogen synthase GlgA	WP_002209498.1	
yeps_YPTB3785	glgC		YPTB_RS20470	YPTB3785		glucose-1-phosphate adenylyltransferase	WP_011193249.1	
yeps_YPTB3788			YPTB_RS20485	YPTB3788		sensor histidine kinase	WP_011193252.1	
yeps_YPTB3796			YPTB_RS20535	YPTB3796		gluconokinase	WP_002209512.1	
yeps_YPTB3797	gntT	gntM,usgA	YPTB_RS20540	YPTB3797		gluconate transporter	WP_011193259.1	
yeps_YPTB3798	gntR		YPTB_RS20545	YPTB3798		gluconate operon transcriptional repressor GntR	WP_011193260.1	
yeps_YPTB3799			YPTB_RS20550	YPTB3799		pirin family protein	WP_002209515.1	
yeps_YPTB3802			YPTB_RS20565	YPTB3802		adenosine kinase	WP_011193262.1	
yeps_YPTB3803			YPTB_RS20570	YPTB3803		ketose 1,6-bisphosphate aldolase	WP_002209519.1	
yeps_YPTB3804			YPTB_RS20575	YPTB3804		D-lyxose/D-mannose family sugar isomerase	WP_011193263.1	
yeps_YPTB3805			YPTB_RS20580	YPTB3805		ABC transporter substrate-binding protein	WP_002209521.1	
yeps_YPTB3806			YPTB_RS20585	YPTB3806		ribose ABC transporter permease	WP_002209522.1	
yeps_YPTB3812	uspA		YPTB_RS20615	YPTB3812		universal stress protein UspA	WP_002209528.1	
yeps_YPTB3813	gdhA		YPTB_RS20620	YPTB3813		NADP-specific glutamate dehydrogenase	WP_002209529.1	
yeps_YPTB3814			YPTB_RS20625	YPTB3814		M10 family metallopeptidase C-terminal domain-containing protein	WP_012414110.1	
yeps_YPTB3815	rsmJ		YPTB_RS20630	YPTB3815		16S rRNA (guanine(1516)-N(2))-methyltransferase RsmJ	WP_002215483.1	
yeps_YPTB3818	gorA		YPTB_RS20645	YPTB3818		glutathione-disulfide reductase	WP_032466815.1	
yeps_YPTB3820			YPTB_RS20655	YPTB3820		GntP family permease	WP_011193273.1	
yeps_YPTB3821			YPTB_RS20660	YPTB3821		glycerate kinase	WP_011193274.1	
yeps_YPTB3829				YPTB3829		possible Tautomerase enzyme	CAH23067.1	
yeps_YPTB3830			YPTB_RS20705	YPTB3830		sugar kinase	WP_011193283.1	
yeps_YPTB3832			YPTB_RS20715	YPTB3832		dicarboxylate/amino acid:cation symporter	WP_002209553.1	
yeps_YPTB3834			YPTB_RS20725	YPTB3834		pectate lyase	WP_002209555.1	
yeps_YPTB3838			YPTB_RS20745	YPTB3838		ABC transporter ATP-binding protein	WP_161597821.1	
yeps_YPTB3839	dppD		YPTB_RS20750	YPTB3839		dipeptide ABC transporter ATP-binding protein	WP_002209562.1	
yeps_YPTB3841	dppB		YPTB_RS20760	YPTB3841		dipeptide ABC transporter permease DppB	WP_002228714.1	
yeps_YPTB3842			YPTB_RS20765	YPTB3842		ABC transporter substrate-binding protein	WP_011193287.1	
yeps_YPTB3846	uhpB		YPTB_RS20795	YPTB3846		signal transduction histidine-protein kinase/phosphatase UhpB	WP_002230402.1	
yeps_YPTB3848	eptB		YPTB_RS20805	YPTB3848		kdo(2)-lipid A phosphoethanolamine 7''-transferase	WP_011193291.1	
yeps_YPTB3850			YPTB_RS20815	YPTB3850		amino acid permease	WP_011193292.1	
yeps_YPTB3856			YPTB_RS20845	YPTB3856		ATP-grasp domain-containing protein	WP_011193294.1	
yeps_YPTB3857			YPTB_RS20850	YPTB3857		siderophore ABC transporter substrate-binding protein	WP_002209581.1	
yeps_YPTB3858			YPTB_RS20855	YPTB3858		ABC transporter permease	WP_011193295.1	
yeps_YPTB3859			YPTB_RS20860	YPTB3859		iron chelate uptake ABC transporter family permease subunit	WP_011193296.1	
yeps_YPTB3888			YPTB_RS21005	YPTB3888		sugar ABC transporter permease	WP_002209589.1	
yeps_YPTB3904	ibpA	hslT,htpN	YPTB_RS21085	YPTB3904		heat shock chaperone IbpA	WP_002209636.1	
yeps_YPTB3905	ibpB	hslS,htpE	YPTB_RS21090	YPTB3905		heat shock chaperone IbpB	WP_002209635.1	
yeps_YPTB3906			YPTB_RS21095	YPTB3906		putative transporter	WP_011193331.1	
yeps_YPTB3910	ghrB		YPTB_RS21115	YPTB3910		glyoxylate/hydroxypyruvate reductase GhrB	WP_002209630.1	
yeps_YPTB3911			YPTB_RS21120	YPTB3911		transcriptional regulator	WP_011193334.1	
yeps_YPTB3912			YPTB_RS21125	YPTB3912		OmpA family lipoprotein	WP_002209628.1	
yeps_YPTB3913			YPTB_RS21130	YPTB3913		N-acetyltransferase	WP_011193335.1	
yeps_YPTB3919			YPTB_RS21160	YPTB3919		mannitol-1-phosphate 5-dehydrogenase	WP_011193338.1	
yeps_YPTB3922			YPTB_RS21175	YPTB3922		DM13 domain-containing protein	WP_002209617.1	
yeps_YPTB3925	sodA		YPTB_RS21190	YPTB3925		superoxide dismutase [Mn]	WP_002209614.1	
yeps_YPTB3926	fdhD		YPTB_RS21195	YPTB3926		formate dehydrogenase accessory sulfurtransferase FdhD	WP_011193341.1	
yeps_YPTB3929	fdoI		YPTB_RS21215	YPTB3929		formate dehydrogenase cytochrome b556 subunit	WP_011193343.1	
yeps_YPTB3930	fdhE		YPTB_RS21220	YPTB3930		formate dehydrogenase accessory protein FdhE	WP_002209609.1	
yeps_YPTB3931			YPTB_RS21225	YPTB3931		L-seryl-tRNA(Sec) selenium transferase	WP_011193344.1	
yeps_YPTB3941	recF	uvrF	YPTB_RS21285	YPTB3941		DNA replication/repair protein RecF	WP_002209643.1	
yeps_YPTB3943	dnaA		YPTB_RS21300	YPTB3943		chromosomal replication initiator protein DnaA	WP_002220732.1	
yeps_YPTB3944				YPTB3944		hypothetical protein	CAH23182.1	
yeps_YPTB3948	yidC		YPTB_RS21315	YPTB3948		membrane protein insertase YidC	WP_002220739.1	
yeps_YPTB3949	mnmE		YPTB_RS21320	YPTB3949		tRNA uridine-5-carboxymethylaminomethyl(34) synthesis GTPase MnmE	WP_011193350.1	
yeps_YPTB3967	atpD	papB,uncD	YPTB_RS21410	YPTB3967		F0F1 ATP synthase subunit beta	WP_002220753.1	
yeps_YPTB3968	atpG	papC,uncG	YPTB_RS21415	YPTB3968		F0F1 ATP synthase subunit gamma	WP_002220756.1	
yeps_YPTB3972	atpE	papH,uncE	YPTB_RS21435	YPTB3972		F0F1 ATP synthase subunit C	WP_000429386.1	
x_1	glnA		YPTB_RS00215	YPTB0024		glutamate--ammonia ligase	WP_002213150.1	

x_2					BZ17_946	hypothetical protein	AJJ56340.1	
x_3					BZ17_887	putative membrane protein	AJJ57145.1	
x_4					BZ17_159	putative membrane protein	AJJ56946.1	
x_5					BZ17_111	hypothetical protein	AJJ56799.1	
x_6	fdnG		YPTB_RS21205	YPTB3927		formate dehydrogenase-N subunit alpha	WP_011193342.1	fragment, translation exception
yeps_x_1	typA	bipA	YPTB_RS00225	YPTB0025		ribosome-dependent GTPase TypA	WP_002217380.1	fragment on the opposite strand
yeps_x_10	emrB		YPTB_RS04770	YPTB0855		multidrug efflux MFS transporter permease subunit EmrB	WP_002208746.1	fragment on the opposite strand
yeps_x_11			YPTB_RS04895	YPTB0880		filamentous hemagglutinin N-terminal domain-containing protein	WP_011191837.1	fragment on the same strand but different frame
yeps_x_12			YPTB_RS04895	YPTB0880		filamentous hemagglutinin N-terminal domain-containing protein	WP_011191837.1	fragment on the opposite strand
yeps_x_13			YPTB_RS04895	YPTB0880		filamentous hemagglutinin N-terminal domain-containing protein	WP_011191837.1	fragment on the opposite strand
yeps_x_14	aroL		YPTB_RS05055	YPTB0911		shikimate kinase AroL	WP_011191851.1	
yeps_x_15					BZ17_1504	rhs element Vgr domain protein	AJJ53360.1	
yeps_x_16			YPTB_RS22525	YPTB1081		hypothetical protein	WP_011191926.1	fragment on the opposite strand
yeps_x_17					BZ17_1459	glycogen synthesis family protein	AJJ54910.1	
yeps_x_18					BZ17_1356	hypothetical protein	AJJ56909.1	
yeps_x_19					BZ17_1338	hypothetical protein	AJJ57070.1	
yeps_x_2	typA	bipA	YPTB_RS00225	YPTB0025		ribosome-dependent GTPase TypA	WP_002217380.1	fragment on the opposite strand
yeps_x_20					BZ17_1234	hypothetical protein	AJJ56635.1	
yeps_x_21					BZ17_1181	hypothetical protein	AJJ53380.1	
yeps_x_22			YPTB_RS07475	YPTB1352		HAAAP family serine/threonine permease	WP_011192035.1	fragment on the opposite strand
yeps_x_23					BZ17_1069	putative membrane protein	AJJ54317.1	
yeps_x_24			YPTB_RS08855	YPTB1608		alpha-galactosidase	WP_011192140.1	fragment on the opposite strand
yeps_x_25	mtfA		YPTB_RS08875	YPTB1611		DgsA anti-repressor MtfA	WP_002211042.1	fragment on the opposite strand
yeps_x_26			YPTB_RS09000	YPTB1632		PTS mannose transporter subunit IID	WP_002211067.1	fragment on the opposite strand
yeps_x_27				YPTB1706	BZ17_795	hypothetical protein	CAH20945.1	fragment
yeps_x_28					BZ17_562	hypothetical protein	AJJ54038.1	
yeps_x_29					BZ17_511	hypothetical protein	AJJ56644.1	
yeps_x_3					BZ17_2521	hypothetical protein	AJJ56380.1	
yeps_x_30					BZ17_482	hypothetical protein	AJJ56760.1	
yeps_x_31								
yeps_x_32					BZ17_324	hypothetical protein	AJJ55948.1	
yeps_x_33			YPTB_RS12180	YPTB2233		RHS repeat protein	CAH21471.1	pseudogene ; fragment on the opposite strand
yeps_x_34					BZ17_162	hypothetical protein	AJJ54783.1	
yeps_x_35					BZ17_66	hypothetical protein	AJJ55378.1	
yeps_x_36					BZ17_60	putative membrane protein	AJJ55430.1	
yeps_x_37					BZ17_29	putative membrane protein	AJJ53437.1	
yeps_x_38								
yeps_x_39			YPTB_RS13510	YPTB2487		AI-2E family transporter	WP_002211853.1	fragment on the opposite strand
yeps_x_4					BZ17_2291	hypothetical protein	AJJ55702.1	
yeps_x_40				YPTB2564		hypothetical protein	CAH21802.1	fragment on the opposite strand
yeps_x_41	nuoM		YPTB_RS13980	YPTB2576		NADH-quinone oxidoreductase subunit M	WP_011192666.1	fragment on the opposite strand
yeps_x_42					BZ17_3718	hypothetical protein	AJJ54599.1	
yeps_x_43	kdpD		YPTB_RS15780	YPTB2919		two-component system sensor histidine kinase KdpD	WP_002209649.1	fragment on the opposite strand
yeps_x_44				YPTB3040				pseudogene
yeps_x_45			YPTB_RS16755	YPTB3097		beta-galactosidase	WP_002209897.1	fragment on the opposite strand
yeps_x_46			YPTB_RS17130	YPTB3162		tyrosine-type recombinase/integrase	WP_011192954.1	fragment on the opposite strand
yeps_x_47	prfB		YPTB_RS17145	YPTB3164		peptide chain release factor 2	WP_002228062.1	fragment
yeps_x_48	gcvP		YPTB_RS17230	YPTB3180		aminomethyl-transferring glycine dehydrogenase	WP_002209947.1	fragment on the opposite strand
yeps_x_49					BZ17_3408	hypothetical protein	AJJ55544.1	
yeps_x_5					BZ17_1959	hypothetical protein	AJJ56833.1	
yeps_x_50			YPTB_RS17680	YPTB3266		lysine N(6)-hydroxylase/L-ornithine N(5)-oxygenase family protein	WP_011193002.1	fragment on the opposite strand ; overlaps next cds
yeps_x_51					BZ17_3208	hypothetical protein	AJJ55852.1	
yeps_x_52			YPTB_RS18590	YPTB3435		membrane protein	WP_002214224.1	
yeps_x_53	uxaC		YPTB_RS18825	YPTB3478		glucuronate isomerase	WP_002210410.1	fragment on the opposite strand
yeps_x_54					BZ17_3092	hypothetical protein	AJJ56691.1	
yeps_x_55	murA		YPTB_RS19020	YPTB3513		UDP-N-acetylglucosamine 1-carboxyvinyltransferase	WP_002210127.1	fragment on the opposite strand
yeps_x_56	yhdP		YPTB_RS19255	YPTB3560		AsmA2 domain-containing protein YhdP	WP_011193163.1	fragment on the opposite strand
yeps_x_57	metH		YPTB_RS19750	YPTB3653		methionine synthase	WP_038400771.1	fragment on the opposite strand
yeps_x_58	mrcA		YPTB_RS20305	YPTB3756		peptidoglycan glycosyltransferase/peptidoglycan DD-transpeptidase MrcA	WP_011193238.1	fragment on the opposite strand
yeps_x_59	malT		YPTB_RS20415	YPTB3776		HTH-type transcriptional regulator MalT	WP_002208926.1	fragment on the opposite strand
yeps_x_6			YPTB_RS03685	YPTB0659		DNA polymerase II	WP_011191720.1	fragment on the opposite strand
yeps_x_60					BZ17_2753	hypothetical protein	AJJ56110.1	
yeps_x_61					BZ17_2741	hypothetical protein	AJJ54297.1	
yeps_x_62			YPTB_RS21095	YPTB3906		putative transporter	WP_011193331.1	fragment on the opposite strand
yeps_x_63					BZ17_2660	hypothetical protein	AJJ53741.1	
yeps_x_64	fdnG		YPTB_RS21205	YPTB3927	BZ17_2652	formate dehydrogenase-N subunit alpha	WP_011193342.1	fragment
yeps_x_65	atpC		YPTB_RS21405	YPTB3966		F0F1 ATP synthase subunit epsilon	WP_002215546.1	fragment on the opposite strand
yeps_x_66	atpD		YPTB_RS21410	YPTB3967		F0F1 ATP synthase subunit beta	WP_002220753.1	fragment on the opposite strand
yeps_x_7	pyrG		YPTB_RS04195	YPTB0754		CTP synthase (glutamine hydrolyzing)	WP_002209376.1	fragment on the opposite strand
yeps_x_8								
yeps_x_9	emrB		YPTB_RS04770	YPTB0855		multidrug efflux MFS transporter permease subunit EmrB	WP_002208746.1	fragment on the opposite strand
YPTB0002	asnC		YPTB_RS00080	YPTB0002		transcriptional regulator AsnC	WP_002212257.1	
YPTB0003			YPTB_RS00085	YPTB0003		aspartate--ammonia ligase	WP_002212256.1	
YPTB0028	dtd		YPTB_RS00240	YPTB0028		D-tyrosyl-tRNA(Tyr) deacylase	WP_002209009.1	
YPTB0033	recG		YPTB_RS00265	YPTB0033		ATP-dependent DNA helicase RecG	WP_002209004.1	
YPTB0037	gmk	spoR	YPTB_RS00285	YPTB0037		guanylate kinase	WP_002209000.1	
YPTB0044	dut	dnaS,sof	YPTB_RS00320	YPTB0044		dUTP diphosphatase	WP_011566231.1	
YPTB0047	rpmB		YPTB_RS00335	YPTB0047		50S ribosomal protein L28	WP_002208991.1	
YPTB0055	rfaD	htrM	YPTB_RS00375	YPTB0055		ADP-glyceromanno-heptose 6-epimerase	WP_002208983.1	
YPTB0070	cpxR		YPTB_RS00450	YPTB0070		envelope stress response regulator transcription factor CpxR	WP_002208970.1	
YPTB0086	glpK		YPTB_RS00530	YPTB0086		glycerol kinase GlpK	WP_002218876.1	
YPTB0089	zapB		YPTB_RS00545	YPTB0089		septal ring assembly protein ZapB	WP_002208953.1	
YPTB0097	hslU		YPTB_RS00585	YPTB0097		HslU--HslV peptidase ATPase subunit	WP_002208943.1	
YPTB0101	priA		YPTB_RS00610	YPTB0101		primosomal protein N'	WP_011191465.1	
YPTB0104	metJ		YPTB_RS00630	YPTB0104		met regulon transcriptional regulator MetJ	WP_004392248.1	
YPTB0121	sthA	sth,udhA	YPTB_RS00725	YPTB0121		Si-specific NAD(P)(+) transhydrogenase	WP_002209477.1	
YPTB0132			YPTB_RS00820	YPTB0132		DUF413 domain-containing protein	WP_002212019.1	
YPTB0134			YPTB_RS00830	YPTB0134		acetolactate synthase 2 catalytic subunit	WP_011191482.1	
YPTB0137	ilvD		YPTB_RS00845	YPTB0137		dihydroxy-acid dehydratase	WP_011191483.1	
YPTB0166	trxA	fipA,tsnC	YPTB_RS01010	YPTB0166		thioredoxin TrxA	WP_002211990.1	
YPTB0167	rho		YPTB_RS01015	YPTB0167		transcription termination factor Rho	WP_002211989.1	
YPTB0171	wecC		YPTB_RS01035	YPTB0171		UDP-N-acetyl-D-mannosamine dehydrogenase	WP_011191499.1	
YPTB0177			YPTB_RS01065	YPTB0177		TDP-N-acetylfucosamine:lipid II N-acetylfucosaminyltransferase	WP_011191503.1	
YPTB0182	hemX		YPTB_RS01110	YPTB0182		uroporphyrinogen-III C-methyltransferase	WP_011191506.1	
YPTB0190	dapF		YPTB_RS01155	YPTB0190		diaminopimelate epimerase	WP_002211471.1	
YPTB0193	yigB		YPTB_RS01170	YPTB0193		5-amino-6-(5-phospho-D-ribitylamino)uracil phosphatase YigB	WP_011191513.1	
YPTB0219			YPTB_RS01305	YPTB0219		DUF1145 family protein	WP_002211501.1	
YPTB0222	ftsE		YPTB_RS01325	YPTB0222		cell division ATP-binding protein FtsE	WP_004391337.1	
YPTB0224	rpoH	fam,hin,htpR	YPTB_RS01335	YPTB0224		RNA polymerase sigma factor RpoH	WP_002211506.1	
YPTB0227	livH		YPTB_RS01350	YPTB0227		high-affinity branched-chain amino acid ABC transporter permease LivH	WP_002211509.1	
YPTB0228			YPTB_RS01355	YPTB0228		high-affinity branched-chain amino acid ABC transporter permease LivM	WP_002211510.1	
YPTB0229	livG		YPTB_RS01360	YPTB0229		high-affinity branched-chain amino acid ABC transporter ATP-binding protein LivG	WP_011191524.1	
YPTB0240	ugpE		YPTB_RS01415	YPTB0240		sn-glycerol-3-phosphate ABC transporter permease UgpE	WP_002211522.1	
YPTB0247	metR		YPTB_RS01450	YPTB0247		HTH-type transcriptional regulator MetR	WP_011191537.1	
YPTB0250	udp		YPTB_RS01465	YPTB0250		uridine phosphorylase	WP_002211528.1	
YPTB0261	tatD		YPTB_RS01520	YPTB0261		3'-5' ssDNA/RNA exonuclease TatD	WP_011191544.1	
YPTB0262	hemB		YPTB_RS01525	YPTB0262		porphobilinogen synthase	WP_002211541.1	
YPTB0264	ubiD		YPTB_RS01535	YPTB0264		4-hydroxy-3-polyprenylbenzoate decarboxylase	WP_011191546.1	
YPTB0265	fre		YPTB_RS01540	YPTB0265		NAD(P)H-flavin reductase	WP_002215918.1	
YPTB0266	fadA	oldA	YPTB_RS01545	YPTB0266		acetyl-CoA C-acyltransferase FadA	WP_002211545.1	
YPTB0267	fadB	oldB	YPTB_RS01550	YPTB0267		fatty acid oxidation complex subunit alpha FadB	WP_011191547.1	
YPTB0270	trkH		YPTB_RS01565	YPTB0270		Trk system potassium transporter TrkH	WP_002211550.1	
YPTB0277	secE		YPTB_RS01650	YPTB0277		preprotein translocase subunit SecE	WP_002210670.1	
YPTB0278	nusG		YPTB_RS01655	YPTB0278		transcription termination/antitermination protein NusG	WP_002210671.1	
YPTB0279	rplK	relC	YPTB_RS01660	YPTB0279		50S ribosomal protein L11	WP_002210672.1	
YPTB0280	rplA		YPTB_RS01665	YPTB0280		50S ribosomal protein L1	WP_002210673.1	
YPTB0282	rplL		YPTB_RS01675	YPTB0282		50S ribosomal protein L7/L12	WP_002210675.1	
YPTB0296			YPTB_RS01755	YPTB0296		YjaG family protein	WP_002230622.1	
YPTB0337			YPTB_RS01985	YPTB0337		iron ABC transporter permease	WP_002209059.1	
YPTB0370	lexA	exrA,spr,tsl,umuA	YPTB_RS02150	YPTB0370		transcriptional repressor LexA	WP_002209090.1	
YPTB0373	pspG		YPTB_RS02165	YPTB0373		envelope stress response protein PspG	WP_002209093.1	
YPTB0375	dnaB	groP,grpA	YPTB_RS02175	YPTB0375		replicative DNA helicase	WP_002209095.1	
YPTB0376	alr		YPTB_RS02180	YPTB0376		alanine racemase	WP_011191596.1	
YPTB0377			YPTB_RS02185	YPTB0377		aspartate/tyrosine/aromatic aminotransferase	WP_011191597.1	
YPTB0402	aspA		YPTB_RS02315	YPTB0402		aspartate ammonia-lyase	WP_002230464.1	
YPTB0404			YPTB_RS02325	YPTB0404		co-chaperone GroES	WP_002209127.1	
YPTB0408	efp		YPTB_RS02345	YPTB0408		elongation factor P	WP_002209131.1	
YPTB0411	frdC		YPTB_RS02360	YPTB0411		fumarate reductase subunit FrdC	WP_002209135.1	
YPTB0412			YPTB_RS02365	YPTB0412		succinate dehydrogenase/fumarate reductase iron-sulfur subunit	WP_002209136.1	
YPTB0424	miaA	trpX	YPTB_RS02455	YPTB0424		tRNA (adenosine(37)-N6)-dimethylallyltransferase MiaA	WP_002209149.1	
YPTB0425	hfq		YPTB_RS02460	YPTB0425		RNA chaperone Hfq	WP_002209151.1	
YPTB0438	rpsF		YPTB_RS02525	YPTB0438		30S ribosomal protein S6	WP_002210153.1	
YPTB0439	priB		YPTB_RS02530	YPTB0439		primosomal replication protein N	WP_002210154.1	
YPTB0440	rpsR		YPTB_RS02535	YPTB0440		30S ribosomal protein S18	WP_002210155.1	
YPTB0441	rplI		YPTB_RS02540	YPTB0441		50S ribosomal protein L9	WP_002210156.1	
YPTB0444			YPTB_RS02555	YPTB0444		peptidylprolyl isomerase	WP_011191620.1	
YPTB0450			YPTB_RS02585	YPTB0450		hemolysin family protein	WP_002210164.1	
YPTB0463	ispB	cel	YPTB_RS02655	YPTB0463		octaprenyl diphosphate synthase	WP_011191628.1	
YPTB0464	rplU		YPTB_RS02660	YPTB0464		50S ribosomal protein L21	WP_002210178.1	
YPTB0465	rpmA		YPTB_RS02665	YPTB0465		50S ribosomal protein L27	WP_002210179.1	
YPTB0470	dacB		YPTB_RS02690	YPTB0470		serine-type D-Ala-D-Ala carboxypeptidase	WP_002217314.1	
YPTB0471	greA		YPTB_RS02695	YPTB0471		transcription elongation factor GreA	WP_002210184.1	
YPTB0472	yhbY		YPTB_RS02700	YPTB0472		ribosome assembly RNA-binding protein YhbY	WP_002210185.1	
YPTB0473	rlmE		YPTB_RS02705	YPTB0473		23S rRNA (uridine(2552)-2'-O)-methyltransferase RlmE	WP_002228196.1	
YPTB0475	folP	dhpS	YPTB_RS02715	YPTB0475		dihydropteroate synthase	WP_011191630.1	
YPTB0476	glmM		YPTB_RS02720	YPTB0476		phosphoglucosamine mutase	WP_002210189.1	
YPTB0478	rimP		YPTB_RS02740	YPTB0478		ribosome maturation factor RimP	WP_002222054.1	
YPTB0481	rbfA	p15B	YPTB_RS02755	YPTB0481		30S ribosome-binding factor RbfA	WP_002209255.1	
YPTB0483	rpsO		YPTB_RS02765	YPTB0483		30S ribosomal protein S15	WP_002209257.1	
YPTB0485	nlpI		YPTB_RS02775	YPTB0485		lipoprotein NlpI	WP_002209260.1	
YPTB0494			YPTB_RS02820	YPTB0494		U32 family peptidase	WP_002209269.1	

YPTB0523			YPTB_RS02975	YPTB0523		ABC transporter permease	WP_002209300.1	
YPTB0530			YPTB_RS03010	YPTB0530		DNA polymerase III subunit chi	WP_002209309.1	
YPTB0531	pepA	ampA,carP,xerB	YPTB_RS03015	YPTB0531		leucyl aminopeptidase	WP_002209310.1	
YPTB0532	lptF		YPTB_RS03020	YPTB0532		LPS export ABC transporter permease LptF	WP_002209311.1	
YPTB0548	lsrF		YPTB_RS03105	YPTB0548		3-hydroxy-5-phosphonooxypentane-2,4-dione thiolase	WP_011191661.1	
YPTB0573			YPTB_RS03250	YPTB0573		DNA polymerase III subunit psi	WP_011191680.1	
YPTB0574	rimI		YPTB_RS03255	YPTB0574		ribosomal protein S18-alanine N-acetyltransferase	WP_002216075.1	
YPTB0576	osmY		YPTB_RS03265	YPTB0576		molecular chaperone OsmY	WP_011191682.1	
YPTB0577			YPTB_RS03270	YPTB0577		DUF1328 domain-containing protein	WP_011191683.1	
YPTB0583	deoB	drm,thyR,tlr	YPTB_RS03300	YPTB0583		phosphopentomutase	WP_011191688.1	
YPTB0598	gpmB		YPTB_RS03375	YPTB0598		2,3-diphosphoglycerate-dependent phosphoglycerate mutase GpmB	WP_002209230.1	
YPTB0599	robA		YPTB_RS03380	YPTB0599		MDR efflux pump AcrAB transcriptional activator RobA	WP_002209231.1	
YPTB0603	thrB		YPTB_RS03400	YPTB0603		homoserine kinase	WP_002209238.1	
YPTB0604	thrC		YPTB_RS03405	YPTB0604		threonine synthase	WP_002209239.1	
YPTB0616	ribF		YPTB_RS03470	YPTB0616		bifunctional riboflavin kinase/FAD synthetase	WP_002210510.1	
YPTB0620	ispH		YPTB_RS03490	YPTB0620		4-hydroxy-3-methylbut-2-enyl diphosphate reductase	WP_011191701.1	
YPTB0622	dapB		YPTB_RS03500	YPTB0622		4-hydroxy-tetrahydrodipicolinate reductase	WP_002210504.1	
YPTB0632	apaG	corD	YPTB_RS03550	YPTB0632		Co2+/Mg2+ efflux protein ApaG	WP_011191709.1	
YPTB0633	rsmA		YPTB_RS03555	YPTB0633		16S rRNA (adenine(1518)-N(6)/adenine(1519)-N(6))-dimethyltransferase RsmA	WP_011191710.1	
YPTB0671	leuB		YPTB_RS03750	YPTB0671		3-isopropylmalate dehydrogenase	WP_011191728.1	
YPTB0685	mraY	murX	YPTB_RS03840	YPTB0685		phospho-N-acetylmuramoyl-pentapeptide-transferase	WP_002210437.1	
YPTB0690			YPTB_RS03865	YPTB0690		D-alanine--D-alanine ligase	WP_002210432.1	
YPTB0692	ftsA	divA	YPTB_RS03875	YPTB0692		cell division protein FtsA	WP_002210431.1	
YPTB0693	ftsZ	sfiB,sulB	YPTB_RS03880	YPTB0693		cell division protein FtsZ	WP_002210430.1	
YPTB0701	zapD		YPTB_RS03925	YPTB0701		cell division protein ZapD	WP_002209318.1	
YPTB0703			YPTB_RS03940	YPTB0703		GMP reductase	WP_002209320.1	
YPTB0712	pdhR	aceC,genA,yacB	YPTB_RS03985	YPTB0712		pyruvate dehydrogenase complex transcriptional repressor PdhR	WP_002216080.1	
YPTB0713	aceE		YPTB_RS03990	YPTB0713		pyruvate dehydrogenase (acetyl-transferring), homodimeric type	WP_011191742.1	
YPTB0719	speD		YPTB_RS04020	YPTB0719		adenosylmethionine decarboxylase	WP_011191745.1	
YPTB0723	hpt		YPTB_RS04040	YPTB0723		hypoxanthine phosphoribosyltransferase	WP_011191748.1	
YPTB0726			YPTB_RS04055	YPTB0726		ABC transporter permease	WP_002209344.1	
YPTB0731	folK	hppK	YPTB_RS04080	YPTB0731		2-amino-4-hydroxy-6-hydroxymethyldihydropteridine diphosphokinase	WP_002209351.1	
YPTB0734	dksA	msmA	YPTB_RS04095	YPTB0734		RNA polymerase-binding protein DksA	WP_002209354.1	
YPTB0744	erpA		YPTB_RS04145	YPTB0744		iron-sulfur cluster insertion protein ErpA	WP_002209365.1	
YPTB0747	mtnN		YPTB_RS04160	YPTB0747		5'-methylthioadenosine/S-adenosylhomocysteine nucleosidase	WP_011191764.1	
YPTB0751	relA		YPTB_RS04180	YPTB0751		GTP diphosphokinase	WP_002209373.1	
YPTB0765	cysD		YPTB_RS04255	YPTB0765		sulfate adenylyltransferase subunit CysD	WP_002209386.1	
YPTB0768			YPTB_RS04270	YPTB0768		DUF3561 family protein	WP_002209389.1	
YPTB0774			YPTB_RS04300	YPTB0774		protein-L-isoaspartate(D-aspartate) O-methyltransferase	WP_002209395.1	
YPTB0833	ffh		YPTB_RS04625	YPTB0833		signal recognition particle protein	WP_011191811.1	
YPTB0834	rpsP		YPTB_RS04630	YPTB0834		30S ribosomal protein S16	WP_002209458.1	
YPTB0835	rimM	yfjA	YPTB_RS04635	YPTB0835		ribosome maturation factor RimM	WP_011191812.1	
YPTB0842	tyrA		YPTB_RS04670	YPTB0842		bifunctional chorismate mutase/prephenate dehydrogenase	WP_002209465.1	
YPTB0851			YPTB_RS04750	YPTB0851		bifunctional acetate--CoA ligase family protein/GNAT family N-acetyltransferase	WP_011191815.1	
YPTB0857	mprA		YPTB_RS04780	YPTB0857		transcriptional repressor MprA	WP_002208744.1	
YPTB0858	ygaH		YPTB_RS04785	YPTB0858		L-valine transporter subunit YgaH	WP_011191819.1	
YPTB0873			YPTB_RS04860	YPTB0873		pyridoxal phosphate-dependent aminotransferase	WP_011191830.1	
YPTB0876			YPTB_RS04875	YPTB0876		acireductone dioxygenase	WP_011191833.1	
YPTB0887			YPTB_RS04930	YPTB0887		Na(+)-translocating NADH-quinone reductase subunit A	WP_002208717.1	
YPTB0889			YPTB_RS04940	YPTB0889		Na(+)-translocating NADH-quinone reductase subunit C	WP_011191841.1	
YPTB0890			YPTB_RS04945	YPTB0890		NADH:ubiquinone reductase (Na(+)-transporting) subunit D	WP_002208714.1	
YPTB0892	nqrF	nqr6	YPTB_RS04955	YPTB0892		NADH:ubiquinone reductase (Na(+)-transporting) subunit F	WP_011191842.1	
YPTB0894	nqrM		YPTB_RS04965	YPTB0894		(Na+)-NQR maturation NqrM	WP_002214684.1	
YPTB0903	crl		YPTB_RS05015	YPTB0903		sigma factor-binding protein Crl	WP_002208702.1	
YPTB0920	brnQ	hrbA	YPTB_RS05110	YPTB0920		branched-chain amino acid transport system II carrier protein	WP_002208680.1	
YPTB0925			YPTB_RS05140	YPTB0925		peroxiredoxin C	WP_002208675.1	
YPTB0930	secD		YPTB_RS05165	YPTB0930		protein translocase subunit SecD	WP_002223272.1	
YPTB0933	nrdR		YPTB_RS05185	YPTB0933		transcriptional regulator NrdR	WP_002208668.1	
YPTB0934	ribD	ribG,ybaE	YPTB_RS05190	YPTB0934		bifunctional diaminohydroxyphosphoribosylaminopyrimidine deaminase/5-amino-6-(5-phosphoribosylamino)uracil reductase RibD	WP_002208667.1	
YPTB0936	nusB	groNB,ssaD,ssyB	YPTB_RS05200	YPTB0936		transcription antitermination factor NusB	WP_002208665.1	
YPTB0950			YPTB_RS05270	YPTB0950		cytochrome o ubiquinol oxidase subunit III	WP_002208651.1	
YPTB0951	cyoB		YPTB_RS05275	YPTB0951		cytochrome o ubiquinol oxidase subunit I	WP_002208650.1	
YPTB0955			YPTB_RS05295	YPTB0955		lipoprotein	WP_002208646.1	
YPTB0958	tig		YPTB_RS05310	YPTB0958		trigger factor	WP_002208643.1	
YPTB0959	clpP	lopP	YPTB_RS05315	YPTB0959		ATP-dependent Clp endopeptidase proteolytic subunit ClpP	WP_002208642.1	
YPTB0960	clpX	lopC	YPTB_RS05320	YPTB0960		ATP-dependent protease ATP-binding subunit ClpX	WP_011191865.1	
YPTB0962	hupB	dpeA,hopD	YPTB_RS05330	YPTB0962		DNA-binding protein HU-beta	WP_002208639.1	
YPTB0963	ppiD	ybaU	YPTB_RS05335	YPTB0963		peptidylprolyl isomerase	WP_011191866.1	
YPTB0966	queC		YPTB_RS05350	YPTB0966		7-cyano-7-deazaguanine synthase QueC	WP_002208635.1	
YPTB0970			YPTB_RS05370	YPTB0970		Lrp/AsnC family transcriptional regulator	WP_002208630.1	
YPTB0979	tomB		YPTB_RS05415	YPTB0979		Hha toxicity modulator TomB	WP_002218472.1	
YPTB0981	ykgO		YPTB_RS05425	YPTB0981		type B 50S ribosomal protein L36	WP_002208618.1	
YPTB0990			YPTB_RS22480	YPTB0990		DUF454 family protein	WP_002228346.1	
YPTB0993			YPTB_RS05485	YPTB0993		YbaB/EbfC family nucleoid-associated protein	WP_002208604.1	
YPTB0994	recR		YPTB_RS05490	YPTB0994		recombination mediator RecR	WP_002208603.1	
YPTB1025			YPTB_RS05655	YPTB1025		SPFH/Band 7/PHB domain protein	WP_002208576.1	
YPTB1027			YPTB_RS05665	YPTB1027		SDR family oxidoreductase	WP_002208574.1	
YPTB1029			YPTB_RS05675	YPTB1029		ABC transporter ATP-binding protein	WP_011191895.1	
YPTB1034	ppiB		YPTB_RS05700	YPTB1034		peptidylprolyl isomerase B	WP_002208567.1	
YPTB1091	lipA	lip	YPTB_RS06015	YPTB1091		lipoyl synthase	WP_002210320.1	
YPTB1096	mrdB		YPTB_RS06040	YPTB1096		peptidoglycan glycosyltransferase MrdB	WP_002210325.1	
YPTB1097	mrdA		YPTB_RS06045	YPTB1097		peptidoglycan DD-transpeptidase MrdA	WP_011191932.1	
YPTB1098	rlmH		YPTB_RS06050	YPTB1098		23S rRNA (pseudouridine(1915)-N(3))-methyltransferase RlmH	WP_002210328.1	
YPTB1103	leuS		YPTB_RS06075	YPTB1103		leucine--tRNA ligase	WP_002210333.1	
YPTB1104			YPTB_RS06080	YPTB1104		zinc ribbon-containing protein	WP_002210335.1	
YPTB1105			YPTB_RS06085	YPTB1105		amino acid ABC transporter ATP-binding protein	WP_002210336.1	
YPTB1106	gltK		YPTB_RS06090	YPTB1106		glutamate/aspartate ABC transporter permease GltK	WP_002210337.1	
YPTB1110	corC		YPTB_RS06110	YPTB1110		CNNM family magnesium/cobalt transport protein CorC	WP_002210342.1	
YPTB1116			YPTB_RS06180	YPTB1116		HAD-IIA family hydrolase	WP_002210349.1	
YPTB1119	nagB	glmD	YPTB_RS06195	YPTB1119		glucosamine-6-phosphate deaminase	WP_002210352.1	
YPTB1125	fldA		YPTB_RS06225	YPTB1125		flavodoxin FldA	WP_002215386.1	
YPTB1135	smpB	smqB	YPTB_RS06280	YPTB1135		SsrA-binding protein SmpB	WP_002210714.1	
YPTB1141	grpE		YPTB_RS06310	YPTB1141		nucleotide exchange factor GrpE	WP_002224622.1	
YPTB1144	sdhD		YPTB_RS06325	YPTB1144		succinate dehydrogenase membrane anchor subunit	WP_002210723.1	
YPTB1145	sdhA		YPTB_RS06330	YPTB1145		succinate dehydrogenase flavoprotein subunit	WP_002210724.1	
YPTB1146			YPTB_RS06335	YPTB1146		succinate dehydrogenase iron-sulfur subunit	WP_002210725.1	
YPTB1147	sucA		YPTB_RS06340	YPTB1147		2-oxoglutarate dehydrogenase E1 component	WP_002210726.1	
YPTB1149	sucC		YPTB_RS06350	YPTB1149		ADP-forming succinate--CoA ligase subunit beta	WP_002210728.1	
YPTB1155	ybgC		YPTB_RS06380	YPTB1155		tol-pal system-associated acyl-CoA thioesterase	WP_002210734.1	
YPTB1157	tolR		YPTB_RS06390	YPTB1157		colicin uptake protein TolR	WP_002210736.1	
YPTB1159	tolB		YPTB_RS06400	YPTB1159		Tol-Pal system beta propeller repeat protein TolB	WP_002210738.1	
YPTB1166	gpmA	gpm	YPTB_RS06470	YPTB1166		2,3-diphosphoglycerate-dependent phosphoglycerate mutase	WP_002210746.1	
YPTB1169	galK	galA	YPTB_RS06485	YPTB1169		galactokinase	WP_011191950.1	
YPTB1172			YPTB_RS06500	YPTB1172		CPBP family intramembrane metalloprotease	WP_002210751.1	
YPTB1192	moaD	chlA4,chlM	YPTB_RS06610	YPTB1192		molybdopterin synthase sulfur carrier subunit	WP_002210773.1	
YPTB1209			YPTB_RS06700	YPTB1209		ABC transporter permease	WP_002220162.1	
YPTB1212	hlyD		YPTB_RS06715	YPTB1212		secretion protein HlyD	WP_011191973.1	
YPTB1253	nrdB	ftsB	YPTB_RS06920	YPTB1253		ribonucleotide-diphosphate reductase subunit beta	WP_002210817.1	
YPTB1297			YPTB_RS07190	YPTB1297		YejL family protein	WP_002208836.1	
YPTB1309	mepS		YPTB_RS07250	YPTB1309		bifunctional murein DD-endopeptidase/murein LD-carboxypeptidase	WP_002208822.1	
YPTB1316	yeiP		YPTB_RS07285	YPTB1316		elongation factor P-like protein YeiP	WP_002228019.1	
YPTB1330	fruK	fpk	YPTB_RS07355	YPTB1330		1-phosphofructokinase	WP_002208798.1	
YPTB1358			YPTB_RS07505	YPTB1358		GrxA family glutaredoxin	WP_002208765.1	
YPTB1361	potF		YPTB_RS07525	YPTB1361		spermidine/putrescine ABC transporter substrate-binding protein PotF	WP_002208762.1	
YPTB1362	potG		YPTB_RS07530	YPTB1362		putrescine ABC transporter ATP-binding subunit PotG	WP_002208760.1	
YPTB1376	artQ		YPTB_RS07610	YPTB1376		arginine ABC transporter permease ArtQ	WP_002211369.1	
YPTB1377	artJ		YPTB_RS07615	YPTB1377		arginine ABC transporter substrate-binding protein	WP_002211368.1	
YPTB1386	hcp		YPTB_RS07660	YPTB1386		hydroxylamine reductase	WP_011192048.1	
YPTB1391	macB		YPTB_RS07685	YPTB1391		macrolide ABC transporter ATP-binding protein/permease MacB	WP_002211351.1	
YPTB1394	clpA	lopD	YPTB_RS07700	YPTB1394		ATP-dependent Clp protease ATP-binding subunit ClpA	WP_011192049.1	
YPTB1395	infA		YPTB_RS07705	YPTB1395		translation initiation factor IF-1	WP_002211347.1	
YPTB1397	cydC	mdrH	YPTB_RS07715	YPTB1397		cysteine/glutathione ABC transporter ATP-binding protein/permease CydC	WP_002211344.1	
YPTB1403			YPTB_RS07750	YPTB1403		replication-associated recombination protein A	WP_002228009.1	
YPTB1408	pflB	pfl,YPO1383	YPTB_RS07775	YPTB1408		formate C-acetyltransferase	WP_002211332.1	
YPTB1414	serC		YPTB_RS07805	YPTB1414		3-phosphoserine/phosphohydroxythreonine transaminase	WP_011192055.1	
YPTB1416	cmk		YPTB_RS07815	YPTB1416		(d)CMP kinase	WP_002211324.1	
YPTB1417	rpsA	ssyF	YPTB_RS07820	YPTB1417		30S ribosomal protein S1	WP_011192057.1	
YPTB1428	mukF	kicB	YPTB_RS07885	YPTB1428		chromosome partition protein MukF	WP_002211310.1	
YPTB1436	asnS		YPTB_RS07925	YPTB1436		asparagine--tRNA ligase	WP_002211301.1	
YPTB1439	pyrD		YPTB_RS07940	YPTB1439		quinone-dependent dihydroorotate dehydrogenase	WP_002211296.1	
YPTB1442	rlmKL		YPTB_RS07955	YPTB1442		bifunctional 23S rRNA (guanine(2069)-N(7))-methyltransferase RlmK/23S rRNA (guanine(2445)-N(2))-methyltransferase RlmL	WP_011192066.1	
YPTB1445	pqiB		YPTB_RS07970	YPTB1445		intermembrane transport protein PqiB	WP_002211290.1	
YPTB1454	sulA	sfiA	YPTB_RS08020	YPTB1454		cell division inhibitor SulA	WP_011192069.1	
YPTB1524	mglC		YPTB_RS08390	YPTB1524		galactose/methyl galactoside ABC transporter permease MglC	WP_002211965.1	
YPTB1531			YPTB_RS08425	YPTB1531		LVIVD repeat-containing protein	WP_011192108.1	
YPTB1535	apbC		YPTB_RS08445	YPTB1535		iron-sulfur cluster carrier protein ApbC	WP_002211871.1	
YPTB1537	dcd	dus,paxA	YPTB_RS08455	YPTB1537		dCTP deaminase	WP_002211873.1	
YPTB1555			YPTB_RS08550	YPTB1555		bifunctional phosphoribosyl-AMP cyclohydrolase/phosphoribosyl-ATP diphosphatase HisIE	WP_002211889.1	
YPTB1557	hisA		YPTB_RS08560	YPTB1557		1-(5-phosphoribosyl)-5-[(5-phosphoribosylamino)methylideneamino]imidazole-4-carboxamide isomerase	WP_002211891.1	
YPTB1558	hisH		YPTB_RS08565	YPTB1558		imidazole glycerol phosphate synthase subunit HisH	WP_002211892.1	
YPTB1559	hisB		YPTB_RS08570	YPTB1559		bifunctional histidinol-phosphatase/imidazoleglycerol-phosphate dehydratase HisB	WP_011192120.1	
YPTB1615			YPTB_RS08905	YPTB1615		helix-turn-helix domain-containing protein	WP_002224436.1	
YPTB1647			YPTB_RS09075	YPTB1647		L-serine ammonia-lyase	WP_011192152.1	
YPTB1724	putP		YPTB_RS09480	YPTB1724		sodium/proline symporter PutP	WP_002211164.1	
YPTB1795	rdgC	yaiD		YPTB1795		possible recombination associated protein RdgC	CAH21034.1	
YPTB1921			YPTB_RS10550	YPTB1921		type 1 fimbrial protein	WP_002213971.1	
YPTB1940			YPTB_RS10650	YPTB1940		iron transporter	WP_002212053.1	
YPTB2001	prs		YPTB_RS10970	YPTB2001		ribose-phosphate diphosphokinase	WP_002211240.1	
YPTB2005	prfA	sueB,uar	YPTB_RS10995	YPTB2005		peptide chain release factor 1	WP_011192398.1	
YPTB2007			YPTB_RS11005	YPTB2007		SirB2 family protein	WP_002211234.1	
YPTB2008			YPTB_RS11010	YPTB2008		invasion regulator SirB1	WP_002211233.1	

YPTB2009	kdsA		YPTB_RS11015	YPTB2009		3-deoxy-8-phosphooctulonate synthase	WP_002211232.1	
YPTB2019			YPTB_RS11080	YPTB2019		phosphatase	WP_002211221.1	
YPTB2023	rimJ		YPTB_RS11100	YPTB2023		ribosomal protein S5-alanine N-acetyltransferase	WP_002211216.1	
YPTB2032	cmoB		YPTB_RS11145	YPTB2032		tRNA 5-methoxyuridine(34)/uridine 5-oxyacetic acid(34) synthase CmoB	WP_011192411.1	
YPTB2038			YPTB_RS11175	YPTB2038		YebC/PmpR family DNA-binding transcriptional regulator	WP_002211202.1	
YPTB2042	znuB		YPTB_RS11195	YPTB2042		zinc ABC transporter permease subunit ZnuB	WP_002211197.1	
YPTB2045	mepM		YPTB_RS11210	YPTB2045		murein DD-endopeptidase MepM	WP_002228437.1	
YPTB2047	pyk		YPTB_RS11220	YPTB2047		pyruvate kinase	WP_002211193.1	
YPTB2053			YPTB_RS11255	YPTB2053		RidA family protein	WP_002211186.1	
YPTB2059	minE		YPTB_RS11285	YPTB2059		cell division topological specificity factor MinE	WP_002211180.1	
YPTB2060	minD		YPTB_RS11290	YPTB2060		septum site-determining protein MinD	WP_002211179.1	
YPTB2064			YPTB_RS11310	YPTB2064		fumarylacetoacetate hydrolase family protein	WP_002211740.1	
YPTB2083	gapA		YPTB_RS11405	YPTB2083		glyceraldehyde-3-phosphate dehydrogenase	WP_002224141.1	
YPTB2084	msrB		YPTB_RS11410	YPTB2084		peptide-methionine (R)-S-oxide reductase MsrB	WP_002211677.1	
YPTB2089			YPTB_RS11435	YPTB2089		NAD(P)H nitroreductase	WP_002211673.1	
YPTB2090	selD	fdhB	YPTB_RS11440	YPTB2090		selenide, water dikinase SelD	WP_011192432.1	
YPTB2116			YPTB_RS11575	YPTB2116		YciI family protein	WP_002210643.1	
YPTB2125	trpA		YPTB_RS11620	YPTB2125		tryptophan synthase subunit alpha	WP_011192442.1	
YPTB2126	trpB		YPTB_RS11625	YPTB2126		tryptophan synthase subunit beta	WP_002210633.1	
YPTB2137			YPTB_RS11680	YPTB2137		YciK family oxidoreductase	WP_002210625.1	
YPTB2139			YPTB_RS11690	YPTB2139		YciN family protein	WP_002210623.1	
YPTB2144	ribA		YPTB_RS11720	YPTB2144		GTP cyclohydrolase II	WP_002227926.1	
YPTB2146			YPTB_RS11730	YPTB2146		LapA family protein	WP_002210616.1	
YPTB2147	lapB		YPTB_RS11735	YPTB2147		lipopolysaccharide assembly protein LapB	WP_011192449.1	
YPTB2156			YPTB_RS11780	YPTB2156		carbon starvation protein A	WP_011192451.1	
YPTB2160	nth		YPTB_RS11800	YPTB2160		endonuclease III	WP_002210602.1	
YPTB2167	rsxA		YPTB_RS11835	YPTB2167		electron transport complex subunit RsxA	WP_002210595.1	
YPTB2261	tpx		YPTB_RS12325	YPTB2261		thiol peroxidase	WP_002210984.1	
YPTB2263			YPTB_RS12335	YPTB2263		DUF2384 domain-containing protein	WP_002210982.1	
YPTB2266			YPTB_RS12350	YPTB2266		YcjX family protein	WP_002210979.1	
YPTB2273	sapB		YPTB_RS12390	YPTB2273		putrescine ABC transporter permease SapB	WP_002210972.1	
YPTB2274	sapC		YPTB_RS12395	YPTB2274		peptide ABC transporter permease SapC	WP_002210971.1	
YPTB2275	sapD		YPTB_RS12400	YPTB2275		peptide ABC transporter ATP-binding protein SapD	WP_002210970.1	
YPTB2283	tyrS		YPTB_RS12440	YPTB2283		tyrosine--tRNA ligase	WP_011192524.1	
YPTB2312	sufC		YPTB_RS12605	YPTB2312		Fe-S cluster assembly ATPase SufC	WP_011192535.1	
YPTB2322			YPTB_RS12660	YPTB2322		lipoate--protein ligase A	WP_011192540.1	
YPTB2323			YPTB_RS12665	YPTB2323		lipoprotein	WP_002216102.1	
YPTB2324	arnF		YPTB_RS12670	YPTB2324		4-amino-4-deoxy-L-arabinose-phosphoundecaprenol flippase subunit ArnF	WP_002211819.1	
YPTB2336	pheT		YPTB_RS12730	YPTB2336		phenylalanine--tRNA ligase subunit beta	WP_011192545.1	
YPTB2337	pheS		YPTB_RS12735	YPTB2337		phenylalanine--tRNA ligase subunit alpha	WP_011192546.1	
YPTB2339	rplT	pdzA	YPTB_RS12740	YPTB2339		50S ribosomal protein L20	WP_002211833.1	
YPTB2340	rpmI		YPTB_RS12745	YPTB2340		50S ribosomal protein L35	WP_002211834.1	
YPTB2341	infC	fit	YPTB_RS22885	YPTB2341		translation initiation factor IF-3	WP_002227898.1	
YPTB2353			YPTB_RS12815	YPTB2353		YniB family protein	WP_002211847.1	
YPTB2354	hxpB		YPTB_RS12820	YPTB2354		hexitol phosphatase HxpB	WP_002220277.1	
YPTB2389			YPTB_RS12995	YPTB2389		hypothetical protein	WP_002210862.1	
YPTB2396	cheZ		YPTB_RS13040	YPTB2396		protein phosphatase CheZ	WP_002210872.1	
YPTB2397	cheY		YPTB_RS13045	YPTB2397		chemotaxis response regulator CheY	WP_002210873.1	
YPTB2427	icd		YPTB_RS13205	YPTB2427		NADP-dependent isocitrate dehydrogenase	WP_002210910.1	
YPTB2432	purB		YPTB_RS13230	YPTB2432		adenylosuccinate lyase	WP_002230790.1	
YPTB2442	lolC		YPTB_RS13280	YPTB2442		lipoprotein-releasing ABC transporter permease subunit LolC	WP_011192589.1	
YPTB2447			YPTB_RS13305	YPTB2447		NAD(P)/FAD-dependent oxidoreductase	WP_002213097.1	
YPTB2448			YPTB_RS13310	YPTB2448		alpha/beta hydrolase YcfP	WP_002213095.1	
YPTB2467	mltG		YPTB_RS13410	YPTB2467		endolytic transglycosylase MltG	WP_011192605.1	
YPTB2470	acpP		YPTB_RS13425	YPTB2470		acyl carrier protein	WP_002220787.1	
YPTB2471	fabG		YPTB_RS13430	YPTB2471		3-oxoacyl-ACP reductase FabG	WP_002210935.1	
YPTB2474	plsX		YPTB_RS13445	YPTB2474		phosphate acyltransferase PlsX	WP_002210932.1	
YPTB2475	rpmF		YPTB_RS13450	YPTB2475		50S ribosomal protein L32	WP_002210931.1	
YPTB2549	glnP		YPTB_RS13840	YPTB2549		glutamine ABC transporter permease GlnP	WP_002210236.1	
YPTB2557	menC		YPTB_RS13875	YPTB2557		o-succinylbenzoate synthase	WP_011192654.1	
YPTB2558	menB		YPTB_RS13880	YPTB2558		1,4-dihydroxy-2-naphthoyl-CoA synthase	WP_002210245.1	
YPTB2577	nuoL		YPTB_RS13985	YPTB2577		NADH-quinone oxidoreductase subunit L	WP_011192667.1	
YPTB2578	nuoK		YPTB_RS13990	YPTB2578		NADH-quinone oxidoreductase subunit NuoK	WP_002210271.1	
YPTB2580	nuoI		YPTB_RS14000	YPTB2580		NADH-quinone oxidoreductase subunit NuoI	WP_002210273.1	
YPTB2581	nuoH		YPTB_RS14005	YPTB2581		NADH-quinone oxidoreductase subunit NuoH	WP_002210274.1	
YPTB2585	nuoC		YPTB_RS14025	YPTB2585		NADH-quinone oxidoreductase subunit C/D	WP_002210277.1	
YPTB2594			YPTB_RS14075	YPTB2594		YfbU family protein	WP_002210286.1	
YPTB2595			YPTB_RS14080	YPTB2595		DUF412 domain-containing protein	WP_011192676.1	
YPTB2597	ackA	ack	YPTB_RS14090	YPTB2597		acetate kinase	WP_011192678.1	
YPTB2600			YPTB_RS14105	YPTB2600		PTS sugar transporter subunit IIA	WP_011192680.1	
YPTB2601			YPTB_RS14110	YPTB2601		PTS sugar transporter subunit IIB	WP_002264679.1	
YPTB2602			YPTB_RS14115	YPTB2602		PTS ascorbate transporter subunit IIC	WP_002222248.1	
YPTB2609			YPTB_RS14150	YPTB2609		histidine ABC transporter permease HisQ	WP_002209736.1	
YPTB2612	purF		YPTB_RS14165	YPTB2612		amidophosphoribosyltransferase	WP_002209733.1	
YPTB2613	cvpA	dedE	YPTB_RS14170	YPTB2613		colicin V production protein	WP_002209732.1	
YPTB2617			YPTB_RS14190	YPTB2617		DedA family protein	WP_002209728.1	
YPTB2630			YPTB_RS14260	YPTB2630		sulfite exporter TauE/SafE family protein	WP_002209713.1	
YPTB2632	aroC		YPTB_RS14270	YPTB2632		chorismate synthase	WP_011192692.1	
YPTB2637	fadI	flrD,fre,fsrC,ubiB	YPTB_RS14295	YPTB2637		acetyl-CoA C-acyltransferase FadI	WP_002209704.1	
YPTB2640	mlaA		YPTB_RS14310	YPTB2640		phospholipid-binding lipoprotein MlaA	WP_002209701.1	
YPTB2714	cysK	cysZ	YPTB_RS14720	YPTB2714		cysteine synthase A	WP_011192736.1	
YPTB2715	ptsH	hpr	YPTB_RS14725	YPTB2715		phosphocarrier protein Hpr	WP_002208488.1	
YPTB2716	ptsI		YPTB_RS14730	YPTB2716		phosphoenolpyruvate-protein phosphotransferase PtsI	WP_002208490.1	
YPTB2761	napD		YPTB_RS14965	YPTB2761		chaperone NapD	WP_002208535.1	
YPTB2775	dapE	msgB	YPTB_RS15035	YPTB2775		succinyl-diaminopimelate desuccinylase	WP_002208549.1	
YPTB2779			YPTB_RS15055	YPTB2779		DUF441 domain-containing protein	WP_002208553.1	
YPTB2781	purC		YPTB_RS15065	YPTB2781		phosphoribosylaminoimidazolesuccinocarboxamide synthase	WP_002208555.1	
YPTB2785	bcp		YPTB_RS15085	YPTB2785		thioredoxin-dependent thiol peroxidase	WP_011192766.1	
YPTB2789			YPTB_RS15105	YPTB2789		AI-2E family transporter	WP_002208563.1	
YPTB2794	upp	uraP	YPTB_RS15130	YPTB2794		uracil phosphoribosyltransferase	WP_002209776.1	
YPTB2797	speG		YPTB_RS15145	YPTB2797		spermidine N1-acetyltransferase	WP_002209779.1	
YPTB2799	pstB	phoT	YPTB_RS15155	YPTB2799		phosphate ABC transporter ATP-binding protein PstB	WP_002209780.1	
YPTB2819			YPTB_RS15255	YPTB2819		YegP family protein	WP_002209797.1	
YPTB2838	bamB		YPTB_RS15360	YPTB2838		outer membrane protein assembly factor BamB	WP_011192801.1	
YPTB2853	iscX		YPTB_RS15435	YPTB2853		Fe-S cluster assembly protein IscX	WP_002209830.1	
YPTB2857	iscA		YPTB_RS15455	YPTB2857		iron-sulfur cluster assembly protein IscA	WP_002209834.1	
YPTB2859			YPTB_RS15465	YPTB2859		IscS subfamily cysteine desulfurase	WP_011192814.1	
YPTB2869			YPTB_RS15515	YPTB2869		serine hydroxymethyltransferase	WP_002211552.1	
YPTB2872	glnB		YPTB_RS15530	YPTB2872		nitrogen regulatory protein P-II	WP_002231018.1	
YPTB2873			YPTB_RS15535	YPTB2873		NAD+ synthase	WP_002211555.1	
YPTB2885			YPTB_RS15595	YPTB2885		YfhL family 4Fe-4S dicluster ferredoxin	WP_002211567.1	
YPTB2886			YPTB_RS15600	YPTB2886		holo-ACP synthase	WP_011192825.1	
YPTB2887	pdxJ		YPTB_RS15605	YPTB2887		pyridoxine 5'-phosphate synthase	WP_011192826.1	
YPTB2889	era	rbaA	YPTB_RS15615	YPTB2889		GTPase Era	WP_002214829.1	
YPTB2890	rnc		YPTB_RS15620	YPTB2890		ribonuclease III	WP_002209679.1	
YPTB2895	rseB		YPTB_RS15645	YPTB2895		sigma-E factor regulatory protein RseB	WP_002209674.1	
YPTB2897	rpoE	sigE	YPTB_RS15655	YPTB2897		RNA polymerase sigma factor RpoE	WP_002209672.1	
YPTB2915			YPTB_RS15750	YPTB2915		YbfA family protein	WP_002209653.1	
YPTB2929	chbA		YPTB_RS15840	YPTB2929		PTS N,N'-diacetylchitobiose transporter subunit IIA	WP_002212242.1	
YPTB2940			YPTB_RS15905	YPTB2940		urease accessory protein UreF	WP_011192843.1	
YPTB2942			YPTB_RS15915	YPTB2942		urease subunit alpha	WP_002212229.1	
YPTB2965	rnhA	dasF,herA,rnh,sdrA	YPTB_RS16035	YPTB2965		ribonuclease HI	WP_002210699.1	
YPTB2966			YPTB_RS16040	YPTB2966		class I SAM-dependent methyltransferase	WP_002210698.1	
YPTB2967	gloB		YPTB_RS16045	YPTB2967		hydroxyacylglutathione hydrolase	WP_011192852.1	
YPTB2973	metN		YPTB_RS16105	YPTB2973		methionine ABC transporter ATP-binding protein MetN	WP_011192854.1	
YPTB2974	metI		YPTB_RS16110	YPTB2974		methionine ABC transporter permease MetI	WP_002212160.1	
YPTB2975			YPTB_RS16115	YPTB2975		MetQ/NlpA family lipoprotein	WP_011192855.1	
YPTB2977	tsaA		YPTB_RS16125	YPTB2977		tRNA (N6-threonylcarbamoyladenosine(37)-N6)-methyltransferase TrmO	WP_002212157.1	
YPTB2978	proS	drpA	YPTB_RS16130	YPTB2978		proline--tRNA ligase	WP_011192856.1	
YPTB2987	accA		YPTB_RS16175	YPTB2987		acetyl-CoA carboxylase carboxyl transferase subunit alpha	WP_002212147.1	
YPTB2993	lpxD	firA,omsA	YPTB_RS16205	YPTB2993		UDP-3-O-(3-hydroxymyristoyl)glucosamine N-acyltransferase	WP_002212141.1	
YPTB2994	skp		YPTB_RS16210	YPTB2994		molecular chaperone Skp	WP_002212140.1	
YPTB2997	cdsA	cds	YPTB_RS16225	YPTB2997		phosphatidate cytidylyltransferase	WP_011192860.1	
YPTB2998	ispU		YPTB_RS16230	YPTB2998		(2E,6E)-farnesyl-diphosphate-specific ditrans,polycis-undecaprenyl-diphosphate synthase	WP_002212136.1	
YPTB3003	rpsB		YPTB_RS16255	YPTB3003		30S ribosomal protein S2	WP_002221800.1	
YPTB3012	queF		YPTB_RS16305	YPTB3012		NADPH-dependent 7-cyano-7-deazaguanine reductase QueF	WP_011192866.1	
YPTB3017			YPTB_RS16330	YPTB3017		transcriptional regulator GcvA	WP_002212117.1	
YPTB3023	argA		YPTB_RS16375	YPTB3023		amino-acid N-acetyltransferase	WP_002211624.1	
YPTB3033	thyA		YPTB_RS16425	YPTB3033		thymidylate synthase	WP_011192873.1	
YPTB3034	lgt	umpA	YPTB_RS16430	YPTB3034		prolipoprotein diacylglyceryl transferase	WP_002211383.1	
YPTB3035	ptsP		YPTB_RS16435	YPTB3035		phosphoenolpyruvate--protein phosphotransferase	WP_011192874.1	
YPTB3036	rppH		YPTB_RS16440	YPTB3036		RNA pyrophosphohydrolase	WP_002211381.1	
YPTB3042	aas		YPTB_RS16480	YPTB3042		bifunctional acyl-ACP--phospholipid O-acyltransferase/long-chain-fatty-acid--ACP ligase	WP_011192876.1	
YPTB3165	recJ		YPTB_RS17150	YPTB3165		single-stranded-DNA-specific exonuclease RecJ	WP_002209931.1	
YPTB3167	xerD		YPTB_RS17160	YPTB3167		site-specific tyrosine recombinase XerD	WP_002209933.1	
YPTB3181	gcvH		YPTB_RS17235	YPTB3181		glycine cleavage system protein GcvH	WP_002209948.1	
YPTB3182	gcvT		YPTB_RS17240	YPTB3182		glycine cleavage system aminomethyltransferase GcvT	WP_011192962.1	
YPTB3196	pgk		YPTB_RS17315	YPTB3196		phosphoglycerate kinase	WP_002209963.1	
YPTB3197	epd	gapB	YPTB_RS17320	YPTB3197		erythrose-4-phosphate dehydrogenase	WP_002209964.1	
YPTB3198	tkt		YPTB_RS17325	YPTB3198		transketolase	WP_011192968.1	
YPTB3205	endA	nucM	YPTB_RS17360	YPTB3205		deoxyribonuclease I	WP_002209973.1	
YPTB3210	aguB		YPTB_RS17390	YPTB3210		N-carbamoylputrescine amidase	WP_002209979.1	
YPTB3215			YPTB_RS17415	YPTB3215		YggT family protein	WP_011192975.1	
YPTB3217			YPTB_RS17425	YPTB3217		XTP/dITP diphosphatase	WP_011192977.1	
YPTB3218	hemW		YPTB_RS17430	YPTB3218		radical SAM family heme chaperone HemW	WP_002209987.1	
YPTB3222			YPTB_RS17450	YPTB3222		YggL family protein	WP_002209990.1	
YPTB3225			YPTB_RS17465	YPTB3225		oxidative damage protection protein	WP_002230648.1	
YPTB3230	mglA	rbsA		YPTB3230		ABC sugar transporter, fused ATP-binding domains	CAH22468.1	
YPTB3313	slyB	pcpY		YPTB3313		Putative outer membrane lipoprotein Pcp precursor	CAH22551.1	
YPTB3319	motA			YPTB3319		putative flagellar motor transmembrane channel protein	CAH22557.1	
YPTB3320	fliA	lafS		YPTB3320		Putative RNA polymerase sigma factor for flagellar operon	CAH22558.1	

YPTB3339	flgC	lfgC		YPTB3339		Putative flagellar basal-body rod protein.	CAH22577.1	
YPTB3340	flgB	lfgB		YPTB3340		putative flagellar basal-body rod protein	CAH22578.1	
YPTB3347	fliG	fla		YPTB3347		puative flagellar motor switch protein	CAH22585.1	
YPTB3352	fliN	mopA		YPTB3352		Flagellar switch protein	CAH22590.1	
YPTB3354	fliQ	mopD		YPTB3354		Putative flagellar assembly/export protein, fliQ	CAH22592.1	
YPTB3357	flhA	fhiA		YPTB3357		Putative flagellar biosynthesis/export membrane protein flhA, fhiA.	CAH22595.1	
YPTB3395	parE	nfxD	YPTB_RS18350	YPTB3395		DNA topoisomerase IV subunit B	WP_011193095.1	
YPTB3396	yqiA		YPTB_RS18355	YPTB3396		esterase YqiA	WP_011193096.1	
YPTB3398			YPTB_RS18365	YPTB3398		DUF1249 family protein	WP_002212183.1	
YPTB3407	hldE		YPTB_RS18420	YPTB3407		bifunctional D-glycero-beta-D-manno-heptose-7-phosphate kinase/D-glycero-beta-D-manno-heptose 1-phosphate adenylyltransferase HldE	WP_011193099.1	
YPTB3413	folB		YPTB_RS18450	YPTB3413		bifunctional dihydroneopterin aldolase/7,8-dihydroneopterin epimerase	WP_002212199.1	
YPTB3416	rpsU		YPTB_RS18465	YPTB3416		30S ribosomal protein S21	WP_001144069.1	
YPTB3476			YPTB_RS18815	YPTB3476		altronate dehydratase family protein	WP_011193131.1	
YPTB3478	uxaC		YPTB_RS18825	YPTB3478		glucuronate isomerase	WP_002210410.1	
YPTB3479			YPTB_RS18835	YPTB3479		MFS transporter	WP_002353699.1	
YPTB3485			YPTB_RS18870	YPTB3485		YqjD family protein	WP_002210418.1	
YPTB3488			YPTB_RS18885	YPTB3488		DoxX family protein	WP_002210421.1	
YPTB3491			YPTB_RS18900	YPTB3491		pirin family protein	WP_011193137.1	
YPTB3498	elbB	elb2	YPTB_RS18935	YPTB3498		isoprenoid biosynthesis glyoxalase ElbB	WP_002210143.1	
YPTB3503			YPTB_RS18965	YPTB3503		glutamate synthase small subunit	WP_002210138.1	
YPTB3506	sspA	pog,ssp	YPTB_RS18980	YPTB3506		stringent starvation protein A	WP_002210135.1	
YPTB3508	rplM		YPTB_RS18995	YPTB3508		50S ribosomal protein L13	WP_002210132.1	
YPTB3510			YPTB_RS19005	YPTB3510		DUF1043 family protein	WP_002210130.1	
YPTB3518	mlaE		YPTB_RS19045	YPTB3518		lipid asymmetry maintenance ABC transporter permease subunit MlaE	WP_002210122.1	
YPTB3523	lptC		YPTB_RS19070	YPTB3523		LPS export ABC transporter periplasmic protein LptC	WP_011193147.1	
YPTB3524	lptA		YPTB_RS19075	YPTB3524		lipopolysaccharide ABC transporter substrate-binding protein LptA	WP_002210117.1	
YPTB3525	lptB		YPTB_RS19080	YPTB3525		LPS export ABC transporter ATP-binding protein	WP_002210116.1	
YPTB3526	rpoN	glnF,ntrA	YPTB_RS19085	YPTB3526		RNA polymerase factor sigma-54	WP_002210115.1	
YPTB3527	hpf		YPTB_RS19090	YPTB3527		ribosome hibernation promoting factor	WP_004392031.1	
YPTB3528	ptsN	rpoP	YPTB_RS19095	YPTB3528		PTS IIA-like nitrogen regulatory protein PtsN	WP_002213952.1	
YPTB3531	pyrB		YPTB_RS19110	YPTB3531		aspartate carbamoyltransferase	WP_011193148.1	
YPTB3533	ridA		YPTB_RS19120	YPTB3533		2-iminobutanoate/2-iminopropanoate deaminase	WP_011193150.1	
YPTB3536	treB		YPTB_RS19135	YPTB3536		PTS trehalose transporter subunit IIBC	WP_011193151.1	
YPTB3538	rnk		YPTB_RS19145	YPTB3538		nucleoside diphosphate kinase regulator	WP_002210103.1	
YPTB3547	aaeA		YPTB_RS19190	YPTB3547		p-hydroxybenzoic acid efflux pump subunit AaeA	WP_002210094.1	
YPTB3548			YPTB_RS19195	YPTB3548		AaeX family protein	WP_002210093.1	
YPTB3549	aaeR		YPTB_RS19200	YPTB3549		HTH-type transcriptional activator AaeR	WP_011193155.1	
YPTB3558	tldD		YPTB_RS19245	YPTB3558		metalloprotease TldD	WP_002210082.1	
YPTB3561	rng		YPTB_RS19260	YPTB3561		ribonuclease G	WP_002210079.1	
YPTB3563	mreD		YPTB_RS19270	YPTB3563		rod shape-determining protein MreD	WP_002210077.1	
YPTB3565	mreB	envB,rodY	YPTB_RS19280	YPTB3565		rod shape-determining protein MreB	WP_002228205.1	
YPTB3570	aroQ		YPTB_RS19305	YPTB3570		type II 3-dehydroquinate dehydratase	WP_002210071.1	
YPTB3574	panF		YPTB_RS19325	YPTB3574		sodium/pantothenate symporter	WP_002210065.1	
YPTB3649	pgi		YPTB_RS19725	YPTB3649		glucose-6-phosphate isomerase	WP_002212085.1	
YPTB3660	aroE		YPTB_RS19825	YPTB3660		shikimate dehydrogenase	WP_002209026.1	
YPTB3663			YPTB_RS19840	YPTB3663		DUF494 family protein	WP_002209023.1	
YPTB3665	def	fms	YPTB_RS19850	YPTB3665		peptide deformylase	WP_002209021.1	
YPTB3668	trkA		YPTB_RS19865	YPTB3668		Trk system potassium transporter TrkA	WP_002209018.1	
YPTB3674	rpsD	ramA	YPTB_RS19895	YPTB3674		30S ribosomal protein S4	WP_002218949.1	
YPTB3675	rpsK		YPTB_RS19900	YPTB3675		30S ribosomal protein S11	WP_002218948.1	
YPTB3676	rpsM		YPTB_RS19905	YPTB3676		30S ribosomal protein S13	WP_002213346.1	
YPTB3677	rpmJ		YPTB_RS19910	YPTB3677		50S ribosomal protein L36	WP_002227352.1	
YPTB3678	secY	prlA	YPTB_RS19915	YPTB3678		preprotein translocase subunit SecY	WP_002213344.1	
YPTB3679	rplO		YPTB_RS19920	YPTB3679		50S ribosomal protein L15	WP_002213341.1	
YPTB3680	rpmD		YPTB_RS19925	YPTB3680		50S ribosomal protein L30	WP_002213339.1	
YPTB3681	rpsE		YPTB_RS19930	YPTB3681		30S ribosomal protein S5	WP_002213337.1	
YPTB3682	rplR		YPTB_RS19935	YPTB3682		50S ribosomal protein L18	WP_002213336.1	
YPTB3683	rplF		YPTB_RS19940	YPTB3683		50S ribosomal protein L6	WP_002213334.1	
YPTB3684	rpsH		YPTB_RS19945	YPTB3684		30S ribosomal protein S8	WP_002213332.1	
YPTB3685	rpsN		YPTB_RS19950	YPTB3685		30S ribosomal protein S14	WP_002213330.1	
YPTB3686	rplE		YPTB_RS19955	YPTB3686		50S ribosomal protein L5	WP_002213329.1	
YPTB3687	rplX		YPTB_RS19960	YPTB3687		50S ribosomal protein L24	WP_002213327.1	
YPTB3688	rplN		YPTB_RS19965	YPTB3688		50S ribosomal protein L14	WP_002213325.1	
YPTB3690	rpmC		YPTB_RS19975	YPTB3690		50S ribosomal protein L29	WP_002218942.1	
YPTB3691	rplP		YPTB_RS19980	YPTB3691		50S ribosomal protein L16	WP_002218940.1	
YPTB3692	rpsC		YPTB_RS19985	YPTB3692		30S ribosomal protein S3	WP_002221644.1	
YPTB3693	rplV	eryB	YPTB_RS19990	YPTB3693		50S ribosomal protein L22	WP_002223844.1	
YPTB3694	rpsS		YPTB_RS19995	YPTB3694		30S ribosomal protein S19	WP_002213430.1	
YPTB3695	rplB		YPTB_RS20000	YPTB3695		50S ribosomal protein L2	WP_002213425.1	
YPTB3696	rplW		YPTB_RS20005	YPTB3696		50S ribosomal protein L23	WP_002213423.1	
YPTB3697	rplD	eryA	YPTB_RS20010	YPTB3697		50S ribosomal protein L4	WP_002218934.1	
YPTB3698	rplC		YPTB_RS20015	YPTB3698		50S ribosomal protein L3	WP_002218932.1	
YPTB3699	rpsJ		YPTB_RS20020	YPTB3699		30S ribosomal protein S10	WP_001181005.1	
YPTB3704	rpsG		YPTB_RS20045	YPTB3704		30S ribosomal protein S7	WP_002212324.1	
YPTB3705	rpsL		YPTB_RS20050	YPTB3705		30S ribosomal protein S12	WP_002212323.1	
YPTB3706	tusB		YPTB_RS20055	YPTB3706		sulfurtransferase complex subunit TusB	WP_002212322.1	
YPTB3709			YPTB_RS20070	YPTB3709		transcriptional regulator	WP_002212319.1	
YPTB3711			YPTB_RS20080	YPTB3711		protein SlyX	WP_002212317.1	
YPTB3714	kefB	trkB	YPTB_RS20095	YPTB3714		glutathione-regulated potassium-efflux system protein KefB	WP_002212314.1	
YPTB3725			YPTB_RS20150	YPTB3725		YheU family protein	WP_011193222.1	
YPTB3729	crp	cap,csm	YPTB_RS20170	YPTB3729		cAMP-activated global transcriptional regulator CRP	WP_002212297.1	
YPTB3732			YPTB_RS20185	YPTB3732		aminodeoxychorismate synthase component II	WP_011193225.1	
YPTB3734	ppiA	rotA	YPTB_RS20195	YPTB3734		peptidylprolyl isomerase A	WP_002208878.1	
YPTB3743	cobA		YPTB_RS20240	YPTB3743		siroheme synthase CysG	WP_011193232.1	
YPTB3746	rpe		YPTB_RS20255	YPTB3746		ribulose-phosphate 3-epimerase	WP_002215694.1	
YPTB3750	aroK		YPTB_RS20275	YPTB3750		shikimate kinase AroK	WP_002208899.1	
YPTB3760	hslR		YPTB_RS20330	YPTB3760		ribosome-associated heat shock protein Hsp15	WP_002208910.1	
YPTB3764	ompR	kmt,ompB	YPTB_RS20350	YPTB3764		two-component system response regulator OmpR	WP_002208914.1	
YPTB3786	glgX		YPTB_RS20475	YPTB3786		glycogen debranching protein GlgX	WP_011193250.1	
YPTB3790	asd	usg-1		YPTB3790		aspartate semialdehyde dehydrogenase	CAH23028.1	
YPTB3791			YPTB_RS20500	YPTB3791		YhgN family NAAT transporter	WP_002209508.1	
YPTB3840	dppC		YPTB_RS20755	YPTB3840		dipeptide ABC transporter permease DppC	WP_002209563.1	
YPTB3847	uhpA		YPTB_RS20800	YPTB3847		transcriptional regulator UhpA	WP_002209570.1	
YPTB3914			YPTB_RS21135	YPTB3914		DNA-3-methyladenine glycosylase I	WP_002209626.1	
YPTB3915	glyQ		YPTB_RS21140	YPTB3915		glycine--tRNA ligase subunit alpha	WP_002209624.1	
YPTB3916	glyS		YPTB_RS21145	YPTB3916		glycine--tRNA ligase subunit beta	WP_002209623.1	
YPTB3920			YPTB_RS21165	YPTB3920		MltR family transcriptional regulator	WP_002209619.1	
YPTB3921			YPTB_RS21170	YPTB3921		YibL family ribosome-associated protein	WP_002209618.1	
YPTB3940	gyrB	acrB,nalC,parA,pcbA	YPTB_RS21280	YPTB3940		DNA topoisomerase (ATP-hydrolyzing) subunit B	WP_002209642.1	
YPTB3942	dnaN		YPTB_RS21295	YPTB3942		DNA polymerase III subunit beta	WP_002209645.1	
YPTB3945	rpmH	rimA,ssaF	YPTB_RS21305	YPTB3945		50S ribosomal protein L34	WP_002220736.1	
YPTB3946	rnpA		YPTB_RS21310	YPTB3946		ribonuclease P protein component	WP_002228153.1	
YPTB3947	yidD		YPTB_RS23380	YPTB3947		membrane protein insertion efficiency factor YidD	WP_002228756.1	
YPTB3950			YPTB_RS21325	YPTB3950		trans-2-enoyl-CoA reductase family protein	WP_002215588.1	
YPTB3960	pstB	phoT	YPTB_RS21375	YPTB3960		phosphate ABC transporter ATP-binding protein PstB	WP_002215562.1	
YPTB3961	pstA	phoT	YPTB_RS21380	YPTB3961		phosphate ABC transporter permease PstA	WP_002215560.1	
YPTB3965	glmU		YPTB_RS21400	YPTB3965		bifunctional UDP-N-acetylglucosamine diphosphorylase/glucosamine-1-phosphate N-acetyltransferase GlmU	WP_002215550.1	
YPTB3966	atpC	papG,uncC	YPTB_RS21405	YPTB3966		F0F1 ATP synthase subunit epsilon	WP_002215546.1	
YPTB3969	atpA	papA,uncA	YPTB_RS21420	YPTB3969		F0F1 ATP synthase subunit alpha	WP_002220758.1	
YPTB3970	atpH	papE,uncH	YPTB_RS21425	YPTB3970		F0F1 ATP synthase subunit delta	WP_002220760.1	
YPTB3971	atpF	papF,uncF	YPTB_RS21430	YPTB3971		F0F1 ATP synthase subunit B	WP_002220762.1	
YPTB3973	atpB	papD,uncB	YPTB_RS21440	YPTB3973		F0F1 ATP synthase subunit A	WP_002228150.1	

Table S2: Allelic profiles of the 35 isolates from 2020 in France.

Isolate	IP43168	IP43186	IP43211	IP43237	IP43278	IP43304	IP43305	IP43330	IP43355	IP43377	IP43382	IP43410	IP43453	IP43476	IP43492	IP43500	IP43579	IP43611	IP43692	IP43716	IP43951	IP43952	IP43989	IP44039	IP44042	IP44063	IP44094	IP44110	IP44154	IP44180	IP44214	IP44616	IP44623	IP44632	IP44652
Lineage	10	11	10	7	16	16	10	5	11	2	7	12	15	15	16	2	15	13	10	10	16	16	16	10	15	15	16	16	16	16	16	15	7	7	10
yeps_YPTB0001	1	1	1	1	1	1	1	1	1	1	1	1	1	1	1	1	1	3	1	1	1	1	1	1	1	1	1	1	1	1	1	1	1	1	1
yeps_YPTB0004	1	5	1	1	1	1	1	6	5	1	1	1	1	1	1	1	1	2	1	1	1	1	1	1	1	1	1	1	1	1	1	1	1	1	1
yeps_YPTB0006	1	6	1	1	1	1	1	7	6	1	1	1	1	1	1	1	1	3	1	1	1	1	1	1	1	1	1	1	1	1	1	1	1	1	1
yeps_YPTB0007	2	2	2	5	1	1	2	1	2	5	5	1	2	2	1	5	2	1	2	2	1	1	1	2	2	2	1	1	1	1	1	2	5	5	2
yeps_YPTB0008	1	2	1	2	1	1	1	6	2	8	2	1	1	1	1	8	1	2	1	1	1	1	1	1	1	1	1	1	1	1	1	1	2	2	1
yeps_YPTB0009	2	1	2	1	1	1	2	1	1	1	1	1	3	3	1	1	3	1	2	2	1	1	1	2	3	3	1	1	1	1	1	3	1	1	2
yeps_YPTB0020	1	1	1	2	1	1	1	2	1	2	2	1	1	1	1	2	1	2	1	1	1	1	1	1	1	1	1	1	1	1	1	1	2	2	1
yeps_YPTB0021	2	2	2	4	1	1	2	2	2	4	4	2	2	2	1	4	2	4	2	2	1	1	1	2	2	2	1	1	1	1	1	2	4	4	2
yeps_YPTB0023	1	1	1	5	1	1	1	1	1	5	5	1	1	1	1	5	1	3	1	1	1	1	1	1	1	1	1	1	1	1	1	1	5	5	1
yeps_YPTB0025	2	1	2	7	1	1	2	5	1	7	7	1	2	2	1	7	2	3	2	2	1	1	1	2	2	2	1	1	1	1	1	2	7	7	2
yeps_YPTB0026	1	4	1	7	1	1	1	5	4	7	7	1	1	1	1	7	1	1	1	1	1	1	1	1	1	1	1	1	1	1	1	1	7	7	1
yeps_YPTB0027	5	7	5	10	1	1	5	8	7	10	10	3	5	5	1	10	5	5	5	5	1	1	1	5	5	5	1	1	1	1	1	5	10	10	5
yeps_YPTB0029	2	2	2	5	1	1	2	3	2	5	5	1	2	2	1	5	2	2	2	2	1	1	1	2	2	2	1	1	1	1	1	2	5	5	2
yeps_YPTB0030	3	5	3	6	1	1	3	1	5	6	6	3	3	3	1	6	3	3	68	3	1	1	1	3	3	3	1	1	1	1	1	3	6	6	3
yeps_YPTB0031	1	3	1	7	1	1	1	6	4	7	7	2	1	1	1	7	1	1	1	1	1	1	1	1	1	1	1	1	1	1	1	1	7	7	1
yeps_YPTB0034	2	1	2	4	1	1	2	1	1	4	4	1	1	1	1	4	1	1	2	2	1	1	1	2	1	1	1	1	1	1	1	1	4	4	2
yeps_YPTB0035	3	7	3	6	1	1	3	8	7	6	6	1	3	3	1	6	3	6	3	3	1	1	1	3	3	3	1	1	1	1	1	3	6	6	3
yeps_YPTB0036	1	1	1	1	1	1	1	1	3	1	1	1	1	1	1	1	1	1	1	1	1	1	1	1	1	1	1	1	1	1	1	1	1	1	1
yeps_YPTB0038	4	8	4	5	1	1	4	9	8	5	5	3	4	4	1	5	4	5	4	4	1	1	1	4	4	4	1	1	1	1	1	4	5	5	4
yeps_YPTB0041	1	1	1	1	1	1	1	1	1	1	1	1	1	1	1	1	1	2	1	1	1	1	1	1	1	1	1	1	1	1	1	1	1	1	1
yeps_YPTB0042	1	3	1	2	1	1	1	6	3	2	2	1	1	1	1	2	1	3	1	1	1	1	1	1	1	1	1	1	1	1	1	1	2	2	1
yeps_YPTB0045	4	5	4	2	3	3	4	5	5	2	2	3	3	3	3	2	3	5	4	4	3	3	3	4	3	3	3	3	3	3	3	3	2	2	4
yeps_YPTB0046	1	1	1	1	1	1	1	1	1	3	1	1	1	1	1	3	1	1	1	1	1	1	1	1	1	1	1	1	1	1	1	1	1	1	1
yeps_YPTB0050	2	4	2	2	1	2	2	3	4	2	2	2	2	2	2	2	2	3	2	2	2	2	2	2	2	2	2	2	2	2	2	2	2	2	2
yeps_YPTB0051	3	6	3	1	1	1	3	4	6	1	1	1	1	1	1	1	1	4	3	3	1	1	1	3	1	1	1	1	1	1	1	1	1	1	3
yeps_YPTB0054	38	2	1	1	1	1	1	1	2	1	1	1	1	1	1	1	1	1	1	1	1	1	1	1	1	1	1	1	1	1	1	1	1	1	1
yeps_YPTB0056	1	7	1	7	1	1	1	3	7	7	7	3	1	1	1	7	1	3	1	1	1	1	1	1	1	1	1	1	1	1	1	1	7	7	1
yeps_YPTB0057	1	2	1	2	1	1	1	2	2	5	2	1	1	1	1	5	1	2	1	1	1	1	1	1	1	1	1	1	1	1	1	1	2	2	1
yeps_YPTB0058	1	5	1	2	1	1	1	2	5	6	2	1	1	1	1	6	1	2	1	1	1	1	1	1	1	1	1	1	1	1	1	1	2	2	1
yeps_YPTB0059	1	6	1	5	1	1	1	7	6	5	5	1	1	1	1	5	1	3	1	1	1	1	1	1	1	1	1	1	1	1	1	1	5	5	1
yeps_YPTB0060	1	8	1	12	1	1	1	4	9	11	12	1	1	6	1	11	6	4	1	1	1	1	1	1	1	1	1	1	1	1	1	1	12	12	1
yeps_YPTB0061	2	1	2	2	1	1	2	2	1	2	2	1	1	1	1	2	1	2	2	2	1	1	1	2	1	1	1	1	1	1	1	1	2	2	3
yeps_YPTB0062	1	1	1	1	1	1	1	2	1	1	1	9	1	1	1	1	1	1	1	1	1	1	1	1	1	1	1	1	1	1	1	1	1	1	1
yeps_YPTB0063	1	1	1	1	1	1	1	1	1	1	1	1	1	1	1	1	1	1	1	1	1	1	1	1	1	1	1	1	1	1	1	1	1	1	1
yeps_YPTB0066	1	5	1	6	1	1	1	1	5	6	6	2	3	3	1	6	3	1	1	1	1	1	1	1	3	3	1	1	1	1	1	3	6	6	1
yeps_YPTB0067	3	9	3	6	1	1	3	6	9	6	6	3	1	1	1	6	1	2	3	3	1	1	1	3	1	1	1	1	1	1	1	1	6	6	3
yeps_YPTB0068	13	8	5	12	1	1	5	9	8	10	12	3	1	1	1	10	1	2	5	5	1	1	1	5	1	1	1	1	1	1	1	1	12	12	5
yeps_YPTB0069	1	9	1	10	1	1	1	6	9	10	10	3	1	1	1	10	1	6	1	1	1	1	1	1	1	1	1	1	1	1	1	1	10	10	1
yeps_YPTB0071	1	2	1	3	1	1	1	2	2	2	3	1	1	1	1	2	1	2	1	1	1	1	1	1	1	1	1	1	1	1	1	1	3	3	1
yeps_YPTB0073	3	3	3	3	1	1	3	3	3	3	3	1	1	1	1	3	1	3	3	3	1	1	1	3	1	1	1	1	1	1	1	1	3	3	3
yeps_YPTB0075	4	3	4	3	1	3	4	4	3	3	3	3	3	3	3	3	3	3	4	4	3	3	3	4	3	3	3	3	3	3	3	3	3	3	4
yeps_YPTB0076	1	4	1	6	1	1	1	1	4	6	6	1	1	1	1	6	1	1	1	1	1	1	1	1	1	1	1	1	1	1	1	1	6	6	1
yeps_YPTB0078	2	1	2	4	1	1	2	2	1	4	4	1	1	1	1	4	1	3	2	2	1	1	1	2	1	1	1	1	1	1	1	1	4	4	2
yeps_YPTB0079	2	1	2	1	1	1	2	1	1	1	1	1	1	1	1	1	1	1	2	5	1	1	1	5	1	1	1	1	1	1	1	1	1	1	2
yeps_YPTB0081	1	2	1	2	1	1	1	2	2	2	2	1	1	1	1	2	1	2	1	1	1	1	1	1	1	1	1	1	1	1	1	1	2	2	1
yeps_YPTB0083	2	3	2	4	1	1	2	1	3	4	4	2	1	1	1	4	1	1	2	2	1	1	1	2	1	1	1	1	1	1	1	1	4	4	2
yeps_YPTB0084	2	2	2	4	1	1	2	1	2	4	4	2	1	1	1	4	1	3	2	2	1	1	1	2	1	1	1	1	1	1	1	1	4	4	2
yeps_YPTB0085	2	4	2	5	1	1	2	1	1	5	5	2	1	1	1	5	1	2	2	2	1	1	1	2	1	1	1	1	1	1	1	1	5	5	2
yeps_YPTB0087	2	1	2	1	1	1	2	1	1	1	1	1	1	1	1	1	1	1	2	2	1	1	1	2	1	1	1	1	1	1	1	1	1	1	2
yeps_YPTB0088	1	1	1	1	1	1	1	1	1	1	1	1	1	1	1	1	1	3	1	1	1	1	1	1	1	1	1	1	1	1	1	1	1	1	1
yeps_YPTB0090	3	1	3	1	1	1	3	6	1	1	1	2	1	1	1	1	1	1	3	3	1	1	1	3	1	1	1	1	1	1	1	1	1	1	3
yeps_YPTB0091	4	1	4	1	1	1	4	4	1	1	1	3	1	1	1	1	1	2	4	4	1	1	1	4	1	1	1	1	1	1	1	1	1	1	4
yeps_YPTB0095	1	1	1	1	1	1	1	1	1	1	1	1	1	1	1	1	1	1	1	1	1	1	1	1	1	1	1	1	1	1	1	1	1	1	1
yeps_YPTB0096	1	2	1	8	1	1	1	6	2	7	8	3	4	4	1	7	4	2	1	1	1	1	1	1	4	4	1	1	1	1	1	4	8	8	1
yeps_YPTB0098	1	1	1	2	1	1	1	1	1	2	2	2	2	2	1	2	2	2	1	1	1	1	1	1	2	2	1	1	1	1	1	2	2	2	1
yeps_YPTB0100	1	1	1	1	1	1	1	1	1	1	1	1	3	3	1	1	3	1	1	1	1	1	1	1	3	3	1	1	1	1	1	3	1	1	1
yeps_YPTB0102	1	1	1	1	1	1	1	1	1	1	1	1	1	1	1	1	1	1	1	1	1	1	1	1	1	1	1	1	1	1	1	1	1	1	1
yeps_YPTB0106	3	7	3	8	1	1	3	2	7	2	2	3	1	1	1	2	1	75	3	3	1	1	1	3	1	1	1	1	1	1	1	1	8	8	3
yeps_YPTB0115	4	9	4	10	1	1	4	10	9	10	10	3	1	1	1	10	1	5	4	4	1	1	1	4	1	1	1	1	1	1	1	1	10	10	4
yeps_YPTB0119	3	3	3	3	1	1	3	1	3	3	3	3	1	1	1	3	1	3	3	3	1	1	1	3	1	1	1	1	1	1	1	1	3	3	3
yeps_YPTB0120	3	5	3	3	1	1	3	3	5	3	3	3	1	1	1	3	1	1	3	3	1	1	1	3	1	1	1	1	1	1	1	1	3	3	3
yeps_YPTB0122	1	2	1	2	1	1	1	1	2	2	2	1	1	1	1	2	1	2	1	1	1	1	1	1	1	1	1	1	1	1	1	1	2	2	1
yeps_YPTB0123	1	1	1	1	1	1	1	1	1	1	1	1	1	1	1	1	1	1	1	1	1	1	1	1	1	1	1	1	1	1	1	1	1	1	1
yeps_YPTB0124	1	5	1	1	1	1	1	1	5	1	1	1	1	1	1	1	1	5	1	1	1	1	1	1	1	1	1	1	1	1	1	1	1	1	1
yeps_YPTB0128	2	2	2	2	1	1	2	2	2	5	2	1	1	1	1	5	1	3	2	2	1	1	1	2	1	1	1	1	1	1	1	1	2	2	2
yeps_YPTB0129	1	1	1	1	1	1	1	1	1	1	1	1	1	1	1	1	1	2	1	1	1	1	1	1	1	1	1	1	1	1	1	1	1	1	1
yeps_YPTB0130	1	5	1	8	1	1	1	6	5	1	8	37	1	1	1	1	1	3	1	1	1	1	1	1	1	1	1	1	1	1	1	1	8	8	1
yeps_YPTB0131	1	2	1	2	1	1	1	1	2	2	2	2	2	2	1	2	2	4	1	1	1	1	1	1	2	2	1	1	1	1	1	2	2	2	1
yeps_YPTB0135	1	1	1	1	1	1	1	1	1	1	1	1	1	1	1	1	1	1	1	1	1	1	1	1	1	1	1	1	1	1	1	1	1	1	1
yeps_YPTB0136	3	3	3	5	1	1	3	3	3	5	5	1	3	3	1	5	3	1	3	3	1	1	1	3	3	3	1	1	1	1	1	3	5	5	3
yeps_YPTB0144	2	1	2	1	1	1	2	1	1	1	1	1	2	2	1	1	2	1	2	2	1	1	1	2	2	2	1	1	1	1	1	2	1	1	2
yeps_YPTB0163	1	3	1	7	1	1	1	3	3	7	7	1	1	1	1	7	1	8	1	1	1	1	1	1	1	1	1	1	1	1	1	1	7	7	1
yeps_YPTB0165	5	6	5	5	1	5	5	7	6	5	5	2	1	1	5	5	1	5	5	5	5	5	5	5	1	1	5	5	5	5	5	1	5	5	5
yeps_YPTB0168	2	2	2	1	1	2	2	1	2	1	1	1	1	1	2	1	1	1	2	2	2	2	2	2	1	1	2	2	2	2	2	1	1	1	2
yeps_YPTB0169	1	5	1	4	1	1	1	1	5	4	4	1	1	1	1	4	1	4	1	1	1	1	1	1	1	1	1	1	1	1	1	1	4	4	1
yeps_YPTB0172	3	1	3	10	11	1	3	7	1	8	10	1	1	1	1	8	1	12	3	3	1	1	1	3	1	1	1	1	1	1	1	1	10	10	3
yeps_YPTB0174	1	5	1	1	1	1	1	3	5	1	1	3	1	1	1	1	1	1	1	1	1	1	1	1	1	1	1	1	1	1	1	1	1	1	1
yeps_YPTB0175	4	8	4	9	1	1	4	3	8	9	9	3	1	1	1	9	1	5	4	4	1	1	1	4	1	1	1	1	1	1	1	1	9	9	4
yeps_YPTB0176	4	8	4	3	1	1	4	3	8	3	3	3	1	1	1	3	1	5	4	4	1	1	1	4	1	1	1	1	1	1	1	1	3	3	4
yeps_YPTB0178	3	8	3	6	1	1	3	10	8	6	6	3	1	1	1	6	1	4	3	3	1	1	1	3	1	1	1	1	1	1	1	1	6	6	3
yeps_YPTB0183	3	6	3	2	1	1	3	2	6	2	2	2	1	1	1	2	1	4	3	3	1	1	1	21	1	1	1	1	1	1	1	24	2	2	3
yeps_YPTB0191	2	3	2	4	1	1	2	2	1	4	4	1	1	1	1	4	1	2	2	2	1	1	1	2	1	1	1	1	1	1	1	1	4	4	2
yeps_YPTB0192	3	6	3	11	1	1	3	9	7	10	11	1	1	1	1	10	1	3	3	3	1	1	1	3	1	1	1	1	1	1	1	1	11	11	3
yeps_YPTB0194	3	8	3	12	1	1	3	9	8	11	12	3	1	1	1	11	1	13	3	3	1	1	1	3	1	1	1	1	1	1	1	1	12	12	3
yeps_YPTB0198	3	9	3	13	1	1	3	10	9	12	13	3	6	6	1	12	6	7	3	3	1	1	1	3	6	6	1	1	1	1	1	6	13	13	3
yeps_YPTB0200	1	1	1	1	1	1	1	1	1	1	1	1	1	1	1	1	1	1	1	1	1	1	1	1	1	1	1	1	1	1	1	1	1	1	1
yeps_YPTB0201	1	3	1	4	1	1	1	1	3	1	4	1	1	1	1	1	1	1	1	1	1	1	1	1	1	1	1	1	1	1	1	1	4	4	1
yeps_YPTB0202	4	7	4	9	1	1	4	8	7	4	9	3	4	4	1	4	4	4	4	4	1	1	1	4	4	4	1	1	1	1	1	4	9	9	4
yeps_YPTB0203	1	1	1	2	1	1	1	2	1	2	2	2	1	1	1	2	1	1	1	1	1	1	1	1	1	1	1	1	1	1	1	1	2	2	1
yeps_YPTB0205	4	4	4	1	1	1	4	1	4	1	1	1	4	4	1	1	4	4	4	4	1	1	1	4	4	4	1	1	1	1	1	4	1	1	4
yeps_YPTB0208	3	1	3	9	1	1	3	6	1	8	9	25	3	3	1	8	3	3	3	3	1	1	1	3	3	3	1	1	1	1	1	3	9	9	3
yeps_YPTB0209	3	7	3	13	1	1	3	8	7	10	13	1	3	3	1	10	3	3	3	3	1	1	1	3	3	3	1	1	1	1	1	3	13	13	3
yeps_YPTB0210	3	7	3	10	1	1	3	8	7	9	10	1	3	3	1	9	3	3	3	3	1	1	1	3	3	75	1	1	1	1	1	3	10	10	3
yeps_YPTB0212	1	1	1	1	1	1	1	1	1	2	1	1	1	1	1	2	1	1	1	1	1	1	1	1	1	1	1	1	1	1	1	1	1	1	1
yeps_YPTB0213	1	3	4	3	1	1	1	3	3	5	3	1	1	1	1	5	1	1	1	1	1	1	1	1	1	1	1	1	1	1	1	1	3	3	1
yeps_YPTB0214	1	1	1	1	1	1	1	1	1	1	1	1	1	1	1	1	1	1	1	1	1	1	1	1	1	1	1	1	1	1	1	1	1	1	1
yeps_YPTB0217	3	2	3	4	1	1	3	2	2	4	4	1	1	1	1	4	1	3	3	3	1	1	1	3	1	1	1	1	1	1	1	1	4	4	3
yeps_YPTB0218	2	2	2	3	1	1	2	2	2	3	3	1	1	1	1	3	1	2	2	2	1	1	1	2	1	1	1	1	1	1	1	1	3	3	2
yeps_YPTB0220	2	2	2	5	1	1	2	2	2	5	5	1	1	1	1	5	1	2	2	2	1	1	1	2	1	1	1	1	1	1	1	1	5	5	2
yeps_YPTB0223	3	5	3	7	1	1	3	6	5	7	7	1	1	1	1	7	1	3	3	3	1	1	1	3	1	1	1	1	1	1	1	1	7	7	3
yeps_YPTB0225	3	1	3	1	1	26	3	1	1	1	1	1	1	1	26	1	1	3	3	3	26	26	26	3	1	1	26	26	26	26	26	1	1	1	3
yeps_YPTB0226	32	7	4	9	1	1	4	8	7	9	9	26	1	1	1	9	1	4	4	4	1	1	1	4	1	1	1	1	1	1	1	1	9	9	4
yeps_YPTB0230	1	7	1	8	1	1	1	1	7	8	8	3	1	1	1	8	1	5	1	1	1	1	1	1	1	1	1	1	1	1	1	1	8	8	1
yeps_YPTB0235	4	4	4	8	1	1	4	3		8	8	3	1	1	1	8	1	5	4	4	1	1	1	4	1	1	1	1	1	1	1	1	8	8	4
yeps_YPTB0242	2	5	2	1	1	1	2	6	5	1	1	1	1	1	1	1	1	3	2	2	1	1	1	2	1	1	1	1	1	1	1	1	1	1	2
yeps_YPTB0243	4	8	4	11	1	1	4	9	8	9	11	1	1	1	1	9	1	5	4	4	1	1	1	4	1	1	1	1	1	1	1	1	11	11	4
yeps_YPTB0246	1	2	1	1	1	1	1	1	2	1	1	3	1	1	1	1	1	4	1	1	1	1	1	1	1	1	1	1	1	1	1	1	1	1	1
yeps_YPTB0248	1	7	1	8	1	1	1	8	7	8	8	3	1	1	1	8	1	5	1	1	1	1	1	1	1	1	1	1	1	1	1	1	8	8	1
yeps_YPTB0249	2	6	2	2	1	1	2	2	6	2	2	2	1	1	1	2	1	3	2	2	1	1	1	2	1	1	1	1	1	1	1	1	2	2	2
yeps_YPTB0254	1	1	1	1	1	1	1	1	1	1	1	1	1	1	1	1	1	3	1	1	1	1	1	1	1	1	1	1	1	1	1	1	1	1	1
yeps_YPTB0255	3	4	3	1	1	1	3	1	4	1	1	1	1	1	1	1	1	1	3	3	1	1	1	3	1	1	1	1	1	1	1	1	1	1	3
yeps_YPTB0256	3	6	3	1	1	1	3	1	6	1	1	1	1	1	1	1	1	4	3	3	1	1	1	3	1	1	1	1	1	1	1	1	1	1	3
yeps_YPTB0257	3	1	3	4	1	1	3	1	1	4	4	1	1	1	1	4	1	1	3	3	1	1	1	3	1	1	1	1	1	1	1	1	4	4	3
yeps_YPTB0258	1	1	1	1	1	1	1	1	1	1	1	1	1	1	1	1	1	1	1	1	1	1	1	1	1	1	1	1	1	1	1	1	1	1	1
yeps_YPTB0259	2	5	2	2	1	1	2	6	5	2	2	1	1	1	1	2	1	2	2	2	1	1	1	2	1	1	1	1	1	1	1	1	2	2	2
yeps_YPTB0260	1	3	1	1	1	1	1	1	3	1	1	12	1	1	1	1	1	1	1	1	1	1	1	1	1	1	1	1	1	1	1	1	1	1	1
yeps_YPTB0268	1	1	3	1	1	1	1	1	1	1	1	1	1	1	1	1	1	1	1	1	1	1	1	1	1	1	1	1	1	1	1	1	1	1	1
yeps_YPTB0269	1	1	1	1	1	1	1	1	1	1	1	1	1	1	1	1	1	1	1	1	1	1	1	1	1	1	1	1		1	1	1	1	1	1
yeps_YPTB0273	2	4	2	2	1	1	2	2	4	32	2	1	1	1	1	2	1	3	2	2	1	1	1	2	1	1	1	1	1	1	1	1	2	2	2
yeps_YPTB0274	1	3	1	1	1	1	1	3	3	1	1	1	1	1	1	1	1	2	1	1	1	1	1	1	1	1	1	1	1	1	1	1	1	1	1
yeps_YPTB0276	4	1	4	1	1	1	4	1	1	1	1	1	1	1	1	1	1	1	4	4	1	1	1	4	1	1	1	1	1	1	1	1	1	1	4
yeps_YPTB0281	1	1	1	1	1	1	1	1	1	1	1	1	1	1	1	1	1	1	1	1	1	1	1	1	1	1	1	1	1	1	1	1	1	1	1
yeps_YPTB0283	3	8	3	8	1	1	3	9	8	8	8	1	6	6	1	8	6	7	3	3	1	1	1	3	6	6	1	1	1	1	1	6	8	8	3
yeps_YPTB0286	1	4	1	6	1	1	1	1	4	6	6	3	1	1	1	6	1	1	1	1	1	1	1	1	1	1	1	1	1	1	1	1	6	6	1
yeps_YPTB0287	1	2	1	1	1	1	1	1	2	1	1	3	1	1	1	1	1	1	1	1	1	1	1	1	1	1	1	1	1	1	1	1	1	1	1
yeps_YPTB0288	4	7	4	8	1	1	4	8	7	8	8	3	1	1	1	8	1	4	4	4	1	1	1	4	1	1	1	1	1	1	1	1	8	8	4
yeps_YPTB0289	4	5	4	1	1	1	4	1	5	1	1	2	1	1	1	1	1	4	4	4	1	1	1	4	1	1	1	1	1	1	1	1	1	1	4
yeps_YPTB0291	1	2	1	1	1	1	1	1	2	1	1	1	1	1	1	1	1	1	1	1	1	1	1	1	1	1	1	1	1	1	1	1	1	1	1
yeps_YPTB0292	1	1	1	4	1	1	1	1	1	4	4	1	1	1	1	4	1	1	1	1	1	1	1	1	1	1	1	1	1	1	1	1	4	4	1
yeps_YPTB0294	1	5	1	7	1	1	1	6	5	7	7	2	3	3	1	7	3	1	1	1	1	1	1	1	3	3	1	1	1	1	1	3	7	7	1
yeps_YPTB0297	1	1	1	1	1	1	1	1	1	1	1	1	1	1	1	1	1	1	1	1	1	1	1	1	1	1	1	1	1	1	1	1	1	1	1
yeps_YPTB0299	8	5	3	3	1	1	3	7	5	3	3	3	1	1	1	3	1	3	3	3	1	1	1	3	1	1	1	1	1	1	1	1	3	3	3
yeps_YPTB0310	1	1	1	5	1	1	1	3	1	6	5	1	1	1	1	6	1	3	1	1	1	1	1	1	1	1	1	1	1	1	1	1	5	5	1
yeps_YPTB0311	3	7	3	11	1	1	3	9	8	10	11	115	1	1	1	10	1	3	3	3	1	1	1	3	1	1	1	1	1	1	1	1	11	11	3
yeps_YPTB0315	3	6	3	8	1	1	3	1	6	7	8	1	1	1	1	7	1	3	3	3	1	1	1	3	1	1	1	1	1	1	1	1	8	8	3
yeps_YPTB0316	2	4	2	1	1	1	2	2	5	2	1	1	1	1	1	2	1	2	2	2	1	1	1	2	1	1	1	1	1	1	1	1	1	1	2
yeps_YPTB0317	1	1	1	4	1	1	1	3	1	1	4	1	1	1	1	1	1	1	1	1	1	1	1	1	1	1	1	1	1	1	1	1	4	4	1
yeps_YPTB0318	1	1	1	3	1	1	1	1	1	1	3	1	1	1	1	1	1	1	1	1	1	1	1	1	1	1	1	1	1	1	1	1	3	3	1
yeps_YPTB0319	3	5	3	11	1	1	3	6	5	8	11	1	9	1	1	8	1	3	3	3	1	1	1	3	1	1	1	1	1	1	1	1	11	11	3
yeps_YPTB0321	2	5	2	8	1	2	2	6	5	2	8	3	4	4	2	2	4	2	2	2	2	2	2	2	4	4	2	2	2	2	2	4	8	8	2
yeps_YPTB0325	3	1	3	7	1	1	3	1	1	6	7	2	2	2	1	6	2	4	3	3	1	1	1	3	2	2	1	1	1	1	1	2	7	7	3
yeps_YPTB0326	4	1	4	9	1	1	4	1	1	8	9	1	1	1	1	8	1	4	3	3	1	1	1	3	1	1	1	1	1	1	1	1	9	9	4
yeps_YPTB0328	1	1	1	2	1	1	1	1	1	2	2	1	1	1	1	2	1	1	1	1	1	1	1	1	1	1	1	1	1	1	1	1	2	2	1
yeps_YPTB0335	2	2	2	4	1	1	2	1	2	4	4	1	1	1	1	4	1	2	2	2	1	1	1	2	1	1	1	1	1	1	1	1	4	4	2
yeps_YPTB0336	2	2	2	5	1	1	2	1	2	5	5	1	1	1	1	5	1	2	2	2	1	1	1	2	1	1	1	1	1	1	1	1	5	5	2
yeps_YPTB0338	4	4	4	4	3	3	4	3	4	4	4	3	3	3	3	4	3	4	4	4	3	3	3	4	3	3	3	3	3	3	3	3	4	4	4
yeps_YPTB0339	3	1	3	6	1	1	3	1	1	6	6	1	1	1	1	6	1	3	3	3	1	1	1	3	1	1	1	1	1	1	1	1	6	6	3
yeps_YPTB0340	1	1	1	1	1	2	1	1	1	1	1	2	2	2	2	1	2	1	1	1	2	2	2	1	2	2	2	2	2	2	2	2	1	1	1
yeps_YPTB0341	1	1	1	1	1	1	1	1	1	1	1	1	2	1	1	1	1	1	1	1	1	1	1	1	1	1	1	1	1	1	1	1	1	1	1
yeps_YPTB0342	1	2	1	2	1	1	1	5	2	2	2	3	2	2	1	2	2	2	1	1	1	1	1	1	2	2	1	1	1	1	1	2	2	2	1
yeps_YPTB0347	1	9	1	12	1	4	1	10	9	12	12	3	4	4	4	12	4	7	1	1	4	4	4	1	4	4	4	4	4	4	4	4	12	12	1
yeps_YPTB0348	1	6	1	7	1	4	1	3	6	7	7	3	4	4	4	7	4	1	1	1	4	4	4	1	4	4	4	4	4	4	4	4	7	7	1
yeps_YPTB0349	1	4	1	4	1	1	1	1	4	4	4	1	1	1	1	4	1	2	1	1	1	1	1	1	1	1	1	1	1	1	1	1	4	4	1
yeps_YPTB0350	3	8	3	10	1	1	3	2	8	10	10	1	1	1	1	10	1	4	3	3	1	1	1	3	1	1	1	1	1	1	1	1	10	10	3
yeps_YPTB0352	1	1	1	1	1	1	1	2	1	1	1	1	1	1	1	1	1	1	1	1	1	1	1	1	1	1	1	1	1	1	1	1	1	1	1
yeps_YPTB0354	1	1	1	1	1	1	1	1	1	1	1	1	1	1	1	1	1	1	1	1	1	1	1	1	1	1	1	1	1	1	1	1	1	1	1
yeps_YPTB0355	1	7	1	3	1	1	1	3	7	8	3	1	1	1	1	8	1	3	1	1	1	1	1	1	1	1	1	1	1	1	1	1	3	3	1
yeps_YPTB0356	1	1	1	1	1	1	1	1	1	1	1	1	1	1	1	1	1	1	1	1	1	1	1	1	1	1	1	1	1	1	1	1	1	1	1
yeps_YPTB0367	3	2	3	3	1	1	3	3	2	3	3	3	1	1	1	3	1	3	3	3	1	1	1	3	1	1	1	1	1	1	1	1	3	3	3
yeps_YPTB0369	1	1	1	2	1	1	1	2	1	2	2	2	1	1	1	2	1	2	1	1	1	1	1	1	1	1	1	1	1	1	1	1	2	2	1
yeps_YPTB0371	1	2	1	1	1	1	1	1	2	1	1	1	1	1	1	1	1	1	1	1	1	1	1	1	1	1	1	1	1	1	1	1	1	1	1
yeps_YPTB0378	1	1	1	1	1	1	1	1	1	1	1	1	1	1	1	1	1	1	1	1	1	1	1	1	1	1	1	1	1	1	1	1	1	1	1

yeps_YPTB0379	10	11	10	1	1	1	10	12	11	14	1	3	1	1	1	14	1	5	10	10	1	1	1	10	1	1	1	1	1	1	1	1	1	1	10
yeps_YPTB0381	1	1	1	2	1	1	1	1	1	2	2	1	1	1	1	2	1	2	1	1	1	1	1	1	1	1	1	1	1	1	1	1	2	2	1
yeps_YPTB0382	3	5	3	9	1	1	3	6	37	9	9	3	3	3	1	9	3	1	3	3	1	1	1	3	3	3	1	1	1	1	1	3	9	9	3
yeps_YPTB0383	4	6	4	6	1	1	4	2	6	6	6	3	3	3	1	6	3	1	4	4	1	1	1	4	3	3	1	1	1	1	1	3	6	6	4
yeps_YPTB0384	2	6	2	7	1	1	2	2	6	7	7	3	3	3	1	7	3	1	2	2	1	1	1	2	3	3	1	1	1	1	1	3	7	7	2
yeps_YPTB0385	5	9	5	12	1	1	5	3	9	12	12	3	3	3	1	12	3	1	5	5	1	1	1	5	3	3	1	1	1	1	1	3	12	12	5
yeps_YPTB0386	2	6	2	2	1	32	2	6	6	2	2	3	3	3	32	2	3	1	2	2	32	32	32	2	3	3	32	32	32	32	32	3	2	2	2
yeps_YPTB0387	2	5	2	6	1	1	2	2	5	6	6	2	2	2	1	6	2	1	2	2	1	1	1	2	2	2	1	1	1	1	1	2	6	6	2
yeps_YPTB0391	1	1	1	2	1	1	1	1	1	2	2	1	1	1	1	2	1	1	1	1	1	1	1	1	1	1	1	1	1	1	1	1	2	2	1
yeps_YPTB0396	8	7	8	10	1	1	8	9	7	10	10	1	1	1	1	10	1	5	8	8	1	1	1	8	1	1	1	1	1	1	1	1	10	10	8
yeps_YPTB0397	3	7	3	3	1	1	3	1	7	3	3	1	1	1	1	3	1	4	3	3	1	1	1	3	1	1	1	1	1	1	1	1	3	3	3
yeps_YPTB0400	1	1	1	1	1	1	1	1	1	1	1	1	1	1	1	1	1	1	1	1	1	1	1	1	1	1	1	1	1	1	1	1	1	1	1
yeps_YPTB0401	8	6	8	9	1	1	8	1	6	9	9	1	1	7	1	9	7	5	4	4	1	1	1	4	1	1	1	1	1	1	1	1	9	9	8
yeps_YPTB0409	1	4	1	2	1	1	1	2	4	2	2	1	1	1	1	2	1	1	1	1	1	1	1	1	1	1	1	1	1	1	1	1	2	2	1
yeps_YPTB0410	1	3	1	1	1	1	1	1	3	1	1	1	1	1	1	1	1	1	1	1	1	1	1	1	1	1	1	1	1	1	1	1	1	1	1
yeps_YPTB0414	1	5	1	1	1	1	1	5	5	1	1	1	1	1	1	1	1	1	1	1	1	1	1	1	1	1	1	1	1	1	1	1	1	1	1
yeps_YPTB0415	1	7	1	10	12	1	1	8	7	10	10	3	3	3	1	10	3	13	1	1	1	1	1	1	3	3	1	1	1	1	1	3	10	10	1
yeps_YPTB0416	1	1	1	2	1	1	1	4	1	2	2	1	1	1	1	2	1	3	1	1	1	1	1	1	1	1	1	1	1	1	1	1	2	2	1
yeps_YPTB0417	1	3	1	2	1	1	1	4	3	2	2	1	1	1	1	2	1	2	1	1	1	1	1	1	1	1	1	1	1	1	1	1	2	2	1
yeps_YPTB0420	4	6	4	10	3	3	4	8	6	71	10	3	3	3	3	9	3	5	4	4	3	3	3	4	3	3	3	3	3	3	3	3	10	10	4
yeps_YPTB0421	1	1	1	2	1	1	1	1	1	2	2	1	1	1	1	2	1	1	1	1	1	1	1	1	1	1	1	1	1	1	1	1	2	2	1
yeps_YPTB0426	2	1	2	1	1	1	2	1	1	1	1	1	1	1	1	1	1	2	2	2	1	1	1	2	1	1	1	1	1	1	1	1	1	1	2
yeps_YPTB0428	3	3	3	3	1	1	3	3	3	3	3	1	1	1	1	3	1	3	3	3	1	1	1	3	1	1	1	1	1	1	1	1	3	3	3
yeps_YPTB0430	3	1	3	1	1	1	3	6	1	1	1	1	1	1	1	1	1	1	3	3	1	1	1	3	1	1	1	1	1	1	1	1	1	1	3
yeps_YPTB0431	1	1	1	1	1	1	1	1	1	1	1	1	1	1	1	1	1	1	1	1	1	1	1	1	1	1	1	1	1	1	1	1	1	1	1
yeps_YPTB0435	9	1	2	1	1	1	2	1	1	1	1	1	1	1	1	1	1	1	2	2	1	1	1	2	1	1	1	1	1	1	1	1	1	1	1
yeps_YPTB0436	3	4	3	5	1	1	3	5	4	5	5	1	1	1	1	5	1	1	3	3	1	1	1	3	1	1	1	1	1	1	1	1	5	5	3
yeps_YPTB0437	1	2	1	4	1	1	1	2	2	4	4	1	1	1	1	4	1	2	1	1	1	1	1	1	1	1	1	1	1	1	1	1	4	4	1
yeps_YPTB0442	1	4	1	6	1	1	1	5	4	6	6	1	1	1	1	6	1	3	1	1	1	1	1	1	1	1	1	1	1	1	1	1	6	6	1
yeps_YPTB0443	2	3	2	3	1	1	2	3	21	3	3	1	1	1	1	3	1	2	2	2	1	1	1	2	1	1	1	1	1	1	1	1	3	3	2
yeps_YPTB0445	1	4	1	7	1	1	1	5	4	6	7	2	2	2	1	6	2	1	1	1	1	1	1	1	2	2	1	1	1	1	1	2	7	7	1
yeps_YPTB0446	1	7	1	11	1	1	1	8	7	10	11	3	3	3	1	10	3	4	1	1	1	1	1	1	3	3	1	1	1	1	1	3	11	11	1
yeps_YPTB0447	1	2	1	7	1	1	1	3	2	6	7	3	3	3	1	6	3	3	1	1	1	1	1	1	3	3	1	1	1	1	1	3	7	7	1
yeps_YPTB0448	1	1	1	2	1	1	1	1	1	1	2	1	1	1	1	1	1	1	1	1	1	1	1	1	1	1	1	1	1	1	1	1	2	2	1
yeps_YPTB0449	1	1	1	2	1	1	1	1	1	1	2	1	1	1	1	1	1	1	1	1	1	1	1	1	1	1	1	1	1	1	1	1	2	2	1
yeps_YPTB0451	1	1	1	4	1	1	1	1	1	1	4	1	1	1	1	1	1	1	1	1	1	1	1	1	1	1	1	1	1	1	1	1	4	4	1
yeps_YPTB0452	2	2	2	2	1	1	2	2	2	2	2	3	3	3	1	2	3	2	2	2	1	1	1	2	3	3	1	1	1	1	1	3	2	2	2
yeps_YPTB0454	1	2	1	2	1	1	1	2	2	2	2	2	2	2	1	2	2	2	1	1	1	1	1	1	2	2	1	1	1	1	1	2	2	2	1
yeps_YPTB0455	1	1	1	1	1	1	1	1	1	1	1	1	1	1	1	1	1	1	1	1	1	1	1	1	1	1	1	1	1	1	1	1	1	1	1
yeps_YPTB0456	1	1	1	1	1	1	1	1	1	1	1	1	1	1	1	1	1	3	1	1	1	1	1	1	1	1	1	1	1	1	1	1	1	1	1
yeps_YPTB0467	1	5	1	7	1	1	1	6	5	7	7	1	1	1	1	7	1	1	8	8	1	1	1	8	16	16	1	1	1	1	1	1	7	7	1
yeps_YPTB0468	3	2	3	6	1	1	3	2	2	6	6	1	1	1	1	6	1	3	3	3	1	1	1	3	1	1	1	1	1	1	1	1	6	6	3
yeps_YPTB0469	2	2	2	2	1	1	2	2	2	6	2	1	1	1	1	6	1	2	2	2	1	1	1	2	1	1	1	1	1	1	1	1	2	2	4
yeps_YPTB0474	1	6	1	9	1	1	1	7	6	9	9	3	1	1	1	9	1	4	1	1	1	1	1	1	1	1	1	1	1	1	1	1	9	9	1
yeps_YPTB0477	2	1	2	2	1	1	2	1	1	2	2	1	1	1	1	2	1	1	2	2	1	1	1	2	1	1	1	1	1	1	1	1	2	2	2
yeps_YPTB0479	2	2	2	3	1	1	2	1	2	3	3	2	1	1	1	3	1	3	2	2	1	1	1	2	1	1	1	1	1	1	1	1	3	3	2
yeps_YPTB0482	1	4	1	6	1	1	1	5	4	1	6	1	1	1	1	1	1	1	1	1	1	1	1	1	1	1	1	1	1	1	1	1	6	6	1
yeps_YPTB0491	3	8	3	3	1	1	3	9	8	11	3	3	12	1	1	11	1	4	3	3	1	1	1	3	1	1	1	1	1	1	1	1	3	3	3
yeps_YPTB0493	2		2	2	1	1	2	5	2	2	2	2	1	1	1	2	1	3	2	2	1	1	1	2	1	1	1	1	1	1	62	1	2	2	2
yeps_YPTB0495	3	1	3	8	1	1	3	1	1	8	8	3	1	1	1	8	1	4	3	3	1	1	1	3	1	1	1	1	1	1	1	1	8	8	3
yeps_YPTB0496	1	4	1	5	1	1	1	1	4	1	5	1	1	1	1	1	1	2	1	1	1	1	1	1	1	1	1	1	1	1	1	1	5	5	1
yeps_YPTB0497	1	4	6	4	1	1	1	1	4	5	4	1	1	1	1	5	1	2	1	1	1	1	1	1	1	1	1	1	1	1	1	1	4	4	1
yeps_YPTB0498	2	1	2	4	1	1	2	2	1	4	4	2	1	1	1	4	1	3	2	2	1	1	1	2	1	1	1	1	1	1	1	1	4	4	2
yeps_YPTB0499	5	9	5	14	1	1	5	11	9	18	14	3	1	1	1	13	1	6	5	5	1	1	1	5	1	1	1	1	1	1	1	1	14	14	5
yeps_YPTB0502	6	7	6	11	1	1	6	1	7	10	11	3	1	1	1	10	16	5	4	4	1	1	1	4	1	1	1	1	1	1	1	1	11	11	6
yeps_YPTB0503	4	8	4	8	1	1	4	9	8	8	8	3	1	1	1	8	1	5	4	4	1	1	1	4	1	1	1	1	1	1	1	1	8	8	4
yeps_YPTB0504	1	6	1	9	1	1	1	7	6	9	9	3	1	1	1	9	1	4	1	1	1	1	1	1	1	1	1	1	1	1	1	1	9	9	1
yeps_YPTB0505	1	4	1	4	1	1	1	3	4	4	4	3	1	1	1	4	1	4	1	1	1	1	1	1	1	1	1	1	1	1	1	1	4	4	1
yeps_YPTB0507	1	6	1	9	1	1	1	7	6	9	9	3	1	1	1	9	1	4	1	1	1	1	1	1	1	1	1	1	1	1	1	1	9	9	1
yeps_YPTB0508	1	1	1	8	1	1	1	5	1	7	8	1	1	1	1	7	1	2	1	1	1	1	1	1	1	1	1	1	1	1	1	1	8	8	1
yeps_YPTB0509	1	4	1	7	1	1	1	5	4	7	7	1	1	1	1	7	1	2	1	1	1	1	1	1	1	1	1	1	1	1	1	1	7	7	1
yeps_YPTB0510	1	6	12	10	1	1	1	7	6	9	10	1	1	1	1	9	1	11	1	1	1	1	1	1	1	1	1	1	1	1	1	1	10	10	1
yeps_YPTB0511	3	7	3	6	1	1	3	3	7	6	6	3	1	5	1	6	5	4	3	3	1	1	1	3	1	1	1	1	1	1	1	1	6	6	3
yeps_YPTB0512	3	4	3	7	1	1	3	3	4	7	7	3	1	1	1	7	1	4	3	3	1	1	1	3	1	1	1	1	1	1	1	1	7	7	3
yeps_YPTB0513	9	8	9	13	1	1	9	11	10	13	13	3	1	1	1	13	1	5	9	9	1	1	1	9	1	1	1	1	1	1	1	1	13	13	9
yeps_YPTB0516	4	1	4	1	1	1	4	8	1	10	1	3	1	1	1	10	1	5	4	4	1	1	1	4	1	1	1	1	1	1	1	1	1	1	4
yeps_YPTB0517	4	8	4	11	1	1	4	3	8	9	11	3	10	4	1	9	4	5	4	4	1	1	1	4	4	4	1	1	1	1	1	4	11	11	4
yeps_YPTB0518	1	1	1	1	1	1	1	1	1	1	1	2	1	1	1	1	1	2	1	1	1	1	1	1	1	1	1	1	1	1	1	1	1	1	1
yeps_YPTB0519	1	6	1	9	1	1	1	1	6	8	9	3	1	1	1	8	1	4	1	1	1	1	1	1	1	1	1	1	1	1	1	1	9	9	1
yeps_YPTB0520	9	7	5	6	1	1	5	8	7	6	6	3	1	1	1	6	1	3	5	5	1	1	1	5	1	1	1	1	1	1	1	1	6	6	5
yeps_YPTB0521	3	6	3	8	9	1	3	7	6	8	8	2	1	1	1	8	1	1	3	3	1	1	1	3	1	1	1	1	1	1	1	1	8	8	3
yeps_YPTB0522	1	6	1	9	1	1	1	10	6	8	9	2	1	1	1	8	1	3	1	1	1	1	1	1	1	1	1	1	1	1	1	1	9	9	1
yeps_YPTB0526	3	1	3	6	1	1	3	3	1	6	6	1	1	1	1	6	1	1	3	3	1	1	1	3	1	1	1	1	1	1	1	1	6	6	3
yeps_YPTB0529	11	10	11	14	1	1	11	2	88	14	14	3	4	4	1	14	4	6	11	11	1	1	1	11	4	4	1	1	1	1	1	4	14	14	11
yeps_YPTB0533	3	6	3	3	1	1	3	8	6	3	3	3	1	1	1	3	1	4	3	3	1	1	1	3	1	1	1	1	1	1	1	1	3	3	3
yeps_YPTB0541	3	2	3	2		1	3	3	2	2	2	1	1	1	1	2	1	3	3	3	1	1	1	3	1	1	1	1	1	1	1	1	2	2	3
yeps_YPTB0542	3	4	3	4	1	1	3	3	4	4	4	1	1	1	1	4	1	3	3	3	1	1	1	3	1	1	1	1	1	1	1	1	4	4	3
yeps_YPTB0546	1	3	1	1	1	1	1	1	3	1	1	1	1	1	1	1	1	1	1	1	1	1	1	1	1	1	1	1	1	1	1	1	1	1	1
yeps_YPTB0547	1	2	1	1	1	1	1	1	2	1	1	1	1	1	1	1	1	1	1	1	1	1	1	1	1	1	1	1	1	1	1	1	1	1	1
yeps_YPTB0549	3	6	3	8	1	9	3	5	7	8	8	1	1	1	9	8	1	3	3	3	9	9	9	3	1	1	9	9	9	9	9	1	8	8	3
yeps_YPTB0550	1	7	1	1	10	1	1	3	7	1	1	3	63	1	1	1	1	1	1	1	1	1	1	1	1	1	1	1	1	1	1	1	1	1	9
yeps_YPTB0551	4	7	4	3	1	10	4	8	7	3	3	3	1	1	10	3	1	9	4	4	10	10	10	4	1	1	10	10	10	10	10	1	3	3	4
yeps_YPTB0552	4	8	4	12	1	4	4	9	8	11	12	3	1	1	4	11	1	5	4	4	4	4	4	4	1	1	4	4	4	4	4	1	12	12	4
yeps_YPTB0554	4	2	4	3	1	1	4	8	2	10	3	3	1	1	1	10	1	5	4	4	1	1	1	4	1	1	1	1	1	1	1	1	3	3	4
yeps_YPTB0564	1	1	1	1	1	1	1	1	1	1	1	1	1	1	1	1	1	1	1	1	1	1	1	1	1	1	1	1	1	1	1	1	1	1	1
yeps_YPTB0565	4	8	4	9	1	1	4	5	8	9	9	3	1	1	1	9	1	5	4	4	1	1	1	4	1	1	1	1	1	1	1	1	9	9	4
yeps_YPTB0575	3	1	3	6	1	1	3	1	1	6	6	1	1	1	1	6	1	1	3	3	1	1	1	3	1	1	1	1	1	1	1	1	6	6	3
yeps_YPTB0581	1	7	1	9	1	1	1	2	7	9	9	3	3	3	1	9	3	4	1	1	1	1	1	1	3	3	1	1	1	1	1	3	9	9	1
yeps_YPTB0582	1	9	1	11	1	1	1	1	9	11	11	3	7	7	1	11	7	6	1	1	1	1	1	1	7	7	1	1	1	1	1	7	11	11	1
yeps_YPTB0585	2	2	2	2	1	1	2	2	2	2	2	2	2	2	1	2	2	1	2	2	1	1	1	2	2	2	1	1	1	1	1	2	2	2	2
yeps_YPTB0586	4	7	4	10	1	5	4	9	7	10	10	3	1	1	5	10	1	5	4	4	5	5	5	4	1	1	5	5	5	5	5	1	10	10	4
yeps_YPTB0587	9	8	9	11	1	51	9	10	8	11	11	3	7	7	51	11	7	5	9	9	51	51	51	9	7	7	51	51	51	51	51	7	11	11	9
yeps_YPTB0588	3	7	3	1	1	1	3	8	7	1	1	1	1	1	1	1	1	4	3	3	1	1	1	3	1	1	1	1	1	1	1	1	1	1	3
yeps_YPTB0590	7	6	4	8	1	1	4	6	6	2	8	3	1	1	1	2	1	2	4	4	1	1	1	4	1	1	1	1	1	1	1	82	8	8	7
yeps_YPTB0591	3	4	3	2	3	3	3	2	4	6	2	2	3	3	3	6	3	2	3	3	3	3	3	3	3	3	3	3	3	3	3	3	2	2	3
yeps_YPTB0593	1	1	1	1	1	1	1	2	1	1	1	1	1	1	1	1	1	1	1	1	1	1	1	1	1	1	1	1	1	1	1	1	1	1	1
yeps_YPTB0595	3	3	3	8	1	1	3	4	3	7	8	1	1	1	1	7	1	3	3	3	1	1	1	3	1	1	1	1	1	1	1	1	8	8	3
yeps_YPTB0597	1	1	1	7	8	1	1	5	1	7	7	1	1	1	1	7	1	1	1	1	1	1	1	1	1	1	1	1	1	1	1	1	7	7	1
yeps_YPTB0600	1	1	1	1	1	1	1	1	1	3	1	1	1	1	1	3	1	1	1	1	1	1	1	1	1	1	1	1	1	1	1	1	1	1	1
yeps_YPTB0611	4	6	4	7	1	1	4	6	6	7	7	3	1	1	1	7	1	4	4	4	1	1	1	4	1	1	1	1	1	1	1	1	7	7	4
yeps_YPTB0612	1	1	1	1	1	1	1	1	1	1	1	1	1	1	1	1	1	1	1	1	1	1	1	1	1	1	1	1	1	1	1	1	1	1	1
yeps_YPTB0613	1	4	1	1	1	1	1	1	1	1	1	1	1	1	1	1	1	5	1	1	1	1	1	1	1	1	1	1	1	1	1	1	1	1	1
yeps_YPTB0615	1	1	1	1	1	1	1	1	1	1	1	1	1	1	1	1	1	1	1	1	1	1	1	1	1	1	1	1	1	1	1	1	1	1	1
yeps_YPTB0617	5	8	5	10	1	1	5	9	8	10	10	3	1	1	1	10	1	6	5	5	1	1	1	5	1	1	1	1	1	1	1	1	10	10	5
yeps_YPTB0618	3	1	3	3	1	1	3	1	1	3	3	1	1	1	1	3	1	1	3	3	1	1	1	3	1	1	1	1	1	1	1	1	3	3	3
yeps_YPTB0619	1	1	1	1	1	1	1	5	1	1	1	3	1	1	1	1	1	1	1	1	1	1	1	1	1	1	1	1	1	1	1	1	1	1	1
yeps_YPTB0623	4	6	4	10	1	1	4	7	6	9	10	3	1	1	1	9	1	1	4	4	1	1	1	4	1	1	1	1	1	1	1	1	10	10	4
yeps_YPTB0624	9	8	9	13	1	1	81	11	10	13	13	3	1	1	1	13	1	6	9	9	1	1	1	9	1	1	1	1	1	1	1	1	13	13	9
yeps_YPTB0625	3	6	3	9	1	1	3	7	6	9	9	3	1	1	1	9	1	1	3	3	1	1	1	3	1	1	1	1	1	1	1	1	9	9	3
yeps_YPTB0626	1	3	1	1	1	1	1	1	3	1	1	1	1	1	1	1	1	3	1	1	1	1	1	1	14	14	1	1	1	1	1	14	1	1	1
yeps_YPTB0627	1	1	1	4	1	1	1	1	1	4	4	1	1	1	1	4	1	1	1	1	1	1	1	1	1	1	1	1	1	1	1	1	4	4	1
yeps_YPTB0631	1	1	1	5	1	1	1	1	1	5	5	1	1	1	1	5	1	1	1	1	1	1	1	1	1	1	1	1	1	1	1	1	5	5	1
yeps_YPTB0634	2	4	2	8	1	1	2	2	4	8	8	2	1	1	1	8	1	4	2	2	1	1	1	2	1	1	1	1	1	1	1	1	8	8	2
yeps_YPTB0635	3	7	3	9	1	1	3	3	7	8	9	3	1	1	1	8	1	4	3	3	1	1	1	3	1	1	1	1	1	1	1	1	9	9	3
yeps_YPTB0637	3	3	3	5	1	1	3	2	3	5	5	2	1	1	1	5	1	2	3	3	1	1	1	3	1	1	1	1	1	1	1	1	5	5	3
yeps_YPTB0641	1	2	1	2	1	1	1	1	2	2	2	1	1	1	1	2	1	1	1	1	1	1	1	1	1	1	1	1	1	1	1	1	2	2	1
yeps_YPTB0643	1	2	1	1	1	1	1	1	2	1	1	3	1	1	1	1	1	2	1	1	1	1	1	1	1	1	1	1	1	1	1	1	1	1	1
yeps_YPTB0644	1	1	1	1	1	1	1	1	1	1	1	1	1	1	1	1	1	1	1	1	1	1	1	1	1	1	1	1	1	1	1	1	1	1	1
yeps_YPTB0645	3	5	3	7	1	1	3	6	5	7	7	3	1	1	1	7	1	4	3	3	1	1	1	3	1	1	1	1	1	1	1	1	7	7	3
yeps_YPTB0647	4	7	4	10	1	1	4	8	7	10	10	3	1	1	1	10	1	5	4	4	1	1	1	4	1	1	1	1	1	1	1	1	10	10	4
yeps_YPTB0649	4	9	4	14	1	1	4	10	9	12	14	3	1	1	1	12	1	7	4	4	1	1	1	4	1	1	1	1	1	1	1	1	14	14	4
yeps_YPTB0651	3	4	3	5	1	1	3	1	4	5	5	2	1	1	1	5	1	1	3	3	1	1	1	3	1	1	1	1	1	1	1	1	5	5	3
yeps_YPTB0652	1	1	1	2	1	1	1	2	1	2	2	2	1	1	1	2	1	1	1	1	1	1	1	1	1	1	1	1	1	1	1	1	2	2	4
yeps_YPTB0653	4	4	4	6	1	1	4	7	4	8	6	3	1	1	1	8	1	5	4	4	1	1	1	4	1	1	1	1	1	1	1	1	6	6	4
yeps_YPTB0654	2	5	2	3	1	1	2	2	6	3	3	2	1	1	1	3	1	3	2	2	1	1	1	2	1	1	1	1	1	1	1	1	3	3	2
yeps_YPTB0660	2	6	2	6	1	1	2	2	6	6	6	2	1	1	1	6	1	3	2	2	1	1	1	2	1	1	1	1	1	1	1	1	6	6	2
yeps_YPTB0661	3	8	3	9	1	1	3	3	8	9	9	3	1	1	1	9	1	5	4	4	1	1	1	4	1	1	1	1	1	1	1	1	9	9	3
yeps_YPTB0662	3	8	3	9	1	1	3	3	8	9	9	3	1	1	1	9	1	4	3	3	1	1	1	3	1	1	1	1	1	1	1	1	9	9	3
yeps_YPTB0668	3	8	3	11	1	1	3	9	8	11	11	2	1	1	1	11	1	4	3	3	1	1	1	3	1	1	1	1	1	1	1	1	11	11	3
yeps_YPTB0669	4	3	4	3	1	1	4	3	3	3	3	3	1	1	1	3	1	3	4	4	1	1	1	4	1	1	1	1	1	1	1	1	3	3	4
yeps_YPTB0670	3	2	3	3	1	1	3	2	2	3	3	3	1	1	1	3	1	2	3	3	1	1	1	3	1	1	1	1	1	1	1	1	3	3	3
yeps_YPTB0680	4	4	4	9	1	1	4	4	4	8	9	3	1	1	1	8	1	5	4	4	1	1	1	4	1	1	1	1	1	1	1	1	9	9	4
yeps_YPTB0681	1	1	1	1	1	1	1	1	1	1	1	1	1	1	1	1	1	1	1	1	1	1	1	1	1	1	1	1	1	1	1	1	1	1	1
yeps_YPTB0682	3	6	3	8	1	1	3	7	6	8	8	1	1	1	1	8	1	4	3	3	1	1	1	3	1	1	1	1	1	1	1	1	8	8	3
yeps_YPTB0683	1	6	1	8	1	1	1	7	6	8	8	3	1	1	1	8	1	4	1	1	1	1	1	1	1	1	1	1	1	1	1	1	8	8	1
yeps_YPTB0684	1	7	1	2	1	1	1	3	7	2	2	3	1	1	1	2	1	6	1	1	1	1	1	1	1	1	1	1	1	1	1	1	2	2	1
yeps_YPTB0686	3	6	3	10	1	1	3	7	6	9	10	3	1	1	1	9	1	4	3	3	1	1	1	3	1	1	1	1	1	1	1	1	10	10	3
yeps_YPTB0687	2	6	2	8	1	1	2	3	6	8	8	3	1	1	1	8	1	6	2	2	1	1	1	2	1	1	1	1	1	1	1	1	8	8	2
yeps_YPTB0688	3	6	3	10	1	1	3	9	8	10	10	1	1	1	1	10	1	4	3	3	1	1	1	3	1	1	1	1	1	1	1	1	10	10	3
yeps_YPTB0689	1	1	1	6	1	1	1	1	1	6	6	1	1	1	1	6	1	3	1	1	1	1	1	1	1	1	1	1	1	1	1	1	6	6	1
yeps_YPTB0694	1	1	1	5	1	1	1	4	1	5	5	1	1	1	1	5	1	1	1	1	1	1	1	1	1	1	1	1	1	1	1	1	5	5	1
yeps_YPTB0695	2	3	2	2	1	1	2	1	3	2	2	1	1	1	1	2	1	1	2	2	1	1	1	2	1	1	1	1	1	1	1	1	2	2	2
yeps_YPTB0696	3	6	3	1	1	1	3	5	6	1	1	2	1	1	1	1	1	4	3	3	1	1	1	3	1	1	1	1	1	1	1	1	1	1	3
yeps_YPTB0706	2	4	2	7	1	1	2	5	4	7	7	21	1	1	1	7	1	2	2	2	1	1	1	2	1	1	1	1	1	1	1	1	7	7	2
yeps_YPTB0708	1	3	1	1	1	1	1	4	3	1	1	1	1	1	1	1	1	2	1	1	1	1	1	1	1	1	1	1	1	1	1	1	1	1	1
yeps_YPTB0710	4	1	4	1	1	1	4	1	1	1	1	1	1	1	1	1	1	1	4	4	1	1	1	4	1	1	1	1	1	1	1	1	1	1	4
yeps_YPTB0717	1	1	1	1	1	1	1	1	1	1	1	1	1	1	1	1	1	2	1	1	1	1	1	1	1	1	1	1	1	1	1	1	1	1	1
yeps_YPTB0718	4	6	4	7	1	1	4	6	6	7	7	3	1	1	1	7	1	1	4	4	1	1	1	4	1	1	1	1	1	1	1	1	7	7	4
yeps_YPTB0720	1	1	1	4	1	1	1	7	6	4	4	1	1	1	1	4	1	4	1	1	1	1	1	1	1	1	1	1	1	1	1	1	4	4	1
yeps_YPTB0721	1	2	1	1	1	1	1	1	2	1	1	1	1	1	1	1	1	1	1	1	1	1	1	1	1	1	1	1	1	1	1	1	1	1	1
yeps_YPTB0722	8	7	8	5	1	1	8	9	7	5	5	1	1	1	1	5	1	5	8	8	1	1	1	8	1	1	1	1	1	1	1	1	5	5	8
yeps_YPTB0724	4	4	4	1	1	1	4	6	4	1	1	3	1	1	1	1	1	1	4	4	1	1	1	4	1	1	1	1	1	1	1	1	1	1	4
yeps_YPTB0725	3	2	3	2	1	1	3	2	5	2	2	1	1	1	1	2	1	2	3	3	1	1	1	3	1	1	1	1	1	1	1	1	2	2	3
yeps_YPTB0727	2	6	2	5	1	1	2	5	6	5	5	1	1	1	1	5	1	3	2	2	1	1	1	2	1	1	1	1	1	1	1	1	5	5	2
yeps_YPTB0728	3	4	3	4	1	1	3	4	4	4	4	3	1	1	1	4	1	2	3	3	1	1	1	3	1	1	1	1	1	1	1	1	4	4	3
yeps_YPTB0729	3	2	3	2	1	1	3	2	2	2	2	3	1	1	1	2	1	3	3	3	1	1	1	3	1	1	1	1	1	1	1	1	2	2	3
yeps_YPTB0730	3	3	3	3	1	1	3	1	3	3	3	3	1	1	1	3	1	4	3	3	1	1	1	3	1	1	1	1	1	1	1	1	3	3	3
yeps_YPTB0738	1	7	1	10	1	1	1	8	7	10	10	3	1	1	1	10	1	4	1	1	1	1	1	1	1	1	1	1	1	1	1	1	10	10	1
yeps_YPTB0739	1	5	1	7	1	1	1	6	5	7	7	1	1	1	1	7	1	3	1	1	1	1	1	1	15	15	1	1	1	1	1	15	7	7	1
yeps_YPTB0745	1	1	1	2	1	1	1	2	1	2	2	1	1	1	1	2	1	1	1	1	1	1	1	1	1	1	1	1	1	1	1	1	2	2	1
yeps_YPTB0746	1	1	1	1	1	1	1	3	1	1	1	1	1	1	1	1	1	1	1	1	1	1	1	1	1	1	1	1	1	1	1	1	1	1	1
yeps_YPTB0748	3	6	3	8	1	1	3	7	6	8	8	1	1	1	1	8	1	4	3	3	1	1	1	3	1	1	1	1	1	1	1	1	8	8	3
yeps_YPTB0749	2	2	2	7	1	1	2	5	2	2	7	3	1	1	1	2	1	8	2	2	1	1	1	2	1	1	1	1	1	1	1	1	7	7	6
yeps_YPTB0753	4	3	4	1	1	1	4	6	3	1	1	3	1	1	1	1	1	4	4	4	1	1	1	4	1	1	1	1	1	1	1	1	1	1	4
yeps_YPTB0754	3	6	3	1	1	1	3	7	6	1	1	1	1	1	1	1	1	3	3	3	1	1	1	3	1	1	1	1	1	1	1	1	1	1	3
yeps_YPTB0755	1	2	1	9	1	1	1	7	2	9	9	3	1	1	1	9	1	1	1	1	1	1	1	1	1	1	1	1	1	1	1	1	9	9	1

yeps_YPTB0757	2	1	2	1	1	1	2	3	1	1	1	1	1	1	1	1	1	2	2	2	1	1	1	2	1	1	1	1	1	1	1	1	1	1	2
yeps_YPTB0758	1	1	1	1	1	1	1	1	1	1	1	1	1	1	1	1	1	1	1	1	1	1	1	1	1	1	1	1	1	1	1	1	1	1	1
yeps_YPTB0760	5	8	5	5	1	1	5	10	8	11	5	1	1	1	1	11	1	5	5	5	1	1	1	5	1	1	1	1	1	1	1	1	5	5	5
yeps_YPTB0763	1	1	1	1	1	1	1	1	1	1	1	1	1	1	1	1	1	1	1	1	1	1	1	1	1	1	1	1	1	1	1	1	1	1	1
yeps_YPTB0766	1	9	1	12	1	1	1	6	8	11	12	3	1	1	1	11	1	1	1	1	1	1	1	1	1	1	1	1	1	1	1	1	12	12	1
yeps_YPTB0767	1	3	1	1	1	1	1	3	3	3	1	3	1	1	1	3	1	1	1	1	1	1	1	1	1	1	1	1	1	1	1	1	1	1	1
yeps_YPTB0769	1	1	3	1	1	1	3	1	1	1	1	1	1	1	1	1	1	1	2	2	1	1	1	2	1	1	1	1	1	1	1	1	1	1	1
yeps_YPTB0771	1	1	1	1	1	1	1	1	1	1	1	1	1	1	1	1	1	1	1	1	1	1	1	1	1	1	1	1	1	1	1	1	1	1	1
yeps_YPTB0772	5	1	5	1	1	1	5	1	1	1	1	3	1	1	1	1	1	5	5	5	1	1	1	5	1	1	1	1	1	1	1	1	1	1	5
yeps_YPTB0773	4	6	4	9	1	1	4	6	7	6	9	3	1	1	1	6	1	4	4	4	1	1	1	4	1	1	1	1	1	1	1	1	9	9	4
yeps_YPTB0775	1	1	1	1	1	1	1	1	1	1	1	1	1	1	1	1	1	1	1	1	1	1	1	1	1	1	1	1	1	1	1	1	1	1	1
yeps_YPTB0779	1	1	1	1	1	1	1	1	1	1	1	1	1	1	1	1	1	1	1	1	1	1	1	1	17	1	1	1	1	1	1	1	1	1	1
yeps_YPTB0780	1	3	1	1	1	1	1	4	3	1	1	1	1	1	1	1	1	1	1	1	1	1	1	1	1	1	1	1	1	1	1	1	1	1	1
yeps_YPTB0781	1	1	1	1	1	1	1	4	1	1	1	2	1	1	1	1	1	1	1	1	1	1	1	1	1	1	1	1	1	1	1	1	1	1	1
yeps_YPTB0782	3	2	3	8	1	1	3	11	2	8	8	1	1	1	1	8	1	4	3	3	1	1	1	3	1	1	1	1	1	1	1	1	8	8	3
yeps_YPTB0783	1	4	1	5	1	1	1	1	4	5	5	1	1	1	1	5	1	2	1	1	1	1	1	1	1	1	1	1	1	1	1	1	5	5	1
yeps_YPTB0792	1	7	1	7	1	1	1	1	7	7	7	1	1	1	1	7	1	5	1	1	1	1	1	1	1	1	1	1	1	1	1	1	7	7	1
yeps_YPTB0794	1	1	1	1	1	1	1	1	1	1	1	1	3	3	1	1	3	1	1	1	1	1	1	1	3	3	1	1	1	1	1	3	1	1	1
yeps_YPTB0795	1	1	1	1	1	1	1	1	7	1	1	1	3	3	1	1	3	4	1	1	1	1	1	1	3	3	1	1	1	1	1	3	1	1	1
yeps_YPTB0798	1	7	1	9	1	1	1	3	7	9	9	3	4	4	1	9	4	5	1	1	1	1	1	1	4	4	1	1	1	1	1	4	9	9	1
yeps_YPTB0800	1	7	1	10	1	1	1	8	7	10	10	3	1	1	1	10	1	11	1	1	1	1	1	1	1	1	1	1	1	1	1	1	10	10	1
yeps_YPTB0802	5	5	5	3	1	4	5	5	5	3	3	3	4	4	4	3	4	3	5	5	4	4	4	5	4	4	4	4	4	4	4	4	3	3	5
yeps_YPTB0803	3	7	3	10	1	1	3	8	7		10	3	1	1	1	10	1	4	3	3	1	1	1	3	1	1	1	1	1	1	1	1	10	10	3
yeps_YPTB0806	1	1	1	5	1	1	1	4	1	5	5	3	3	3	1	5	3	4	1	1	1	1	1	1	3	3	1	1	1	1	1	3	5	5	1
yeps_YPTB0807	5	9	5	9	1	1	5	13	9	11	9	3	4	4	1	11	4	6	5	5	1	1	1	5	4	4	1	1	1	1	1	4	9	9	5
yeps_YPTB0811	1	7	1	12	1	1	1	8	7	10	12	3	3	3	1	10	3	4	1	1	1	1	1	1	3	3	1	1	1	1	1	3	12	12	1
yeps_YPTB0812	1	7	1	11	1	1	1	9	7	11	11	3	3	3	1	11	3	4	1	1	1	1	1	1	3	3	1	1	1	1	1	3	11	11	1
yeps_YPTB0813	1	1	1	1	1	1	1	4	1	1	1	1	1	1	1	1	1	1	1	1	1	1	1	1	1	1	1	1	1	1	1	1	1	1	1
yeps_YPTB0814	10	8	4	8	1	1	4	8	8	8	8	3	3	3	1	8	3	4	4	4	1	1	1	4	3	3	1	1	1	1	1	3	8	8	4
yeps_YPTB0816	3	5	3	7	1	1	3	5	5	5	7	1	1	1	1	5	1	4	3	3	1	1	1	3	1	1	1	1	1	1	1	1	7	7	3
yeps_YPTB0819	4	6	4	9	1	1	4	7	6	9	9	3	1	1	1	9	1	4	4	4	1	1	1	4	1	1	1	1	1	1	1	1	9	9	4
yeps_YPTB0820	3	3	3	6	1	1	3	2	5	6	6	3	1	1	1	6	1	3	3	3	1	1	1	3	1	1	1	1	1	1	1	1	6	6	3
yeps_YPTB0821	3	5	3	5	1	1	3	7	5	5	5	1	1	1	1	5	1	3	3	3	1	1	1	3	1	1	1	1	1	1	1	1	5	5	3
yeps_YPTB0822	1	3	1	3	1	1	1	1	3	3	3	1	1	1	1	3	1	1	1	1	1	1	1	1	1	1	1	1	1	1	1	1	3	3	1
yeps_YPTB0825	14	8	4	12	1	1	4	10	8	12	12	3	13	6	1	12	6	4	4	4	1	1	1	4	1	1	1	1	1	1	1	1	12	12	4
yeps_YPTB0828	1	1	2	1	1	1	2	1	1	1	1	1	1	1	1	1	1	1	2	2	1	1	1	2	1	1	1	1	1	1	1	1	1	1	2
yeps_YPTB0829	1	7	1	13	1	1	1	9	7	11	13	3	1	1	1	11	1	1	1	1	1	1	1	1	1	1	1	1	1	1	1	1	13	13	12
yeps_YPTB0832	1	1	1	7	1	1	1	6	1	7	7	1	1	1	1	7	1	4	1	1	1	1	1	1	1	1	1	1	1	1	1	1	7	7	1
yeps_YPTB0836	1	1	1	2	1	1	1	1	1	2	2	1	1	1	1	2	1	2	1	1	1	1	1	1	1	1	1	1	1	1	1	1	2	2	1
yeps_YPTB0850	3	2	3	2	1	1	3	4	2	2	2	3	3	3	1	2	3	2	3	3	1	1	1	3	3	3	1	1	1	1	1	3	2	2	3
yeps_YPTB0853	1	6	1	4	1	1	1	7	6	4	4	3	4	4	1	4	4	3	1	1	1	1	1	1	4	4	1	1	1	1	1	4	4	4	1
yeps_YPTB0856	3	6	3	1	1	1	3	7	6	1	1	3	1	1	1	1	1	3	3	3	1	1	1	3	1	1	1	1	1	1	1	1	1	1	3
yeps_YPTB0860	1	6	1	9	1	1	1	8	6	9	9	1	1	1	1	9	1	3	1	1	1	1	1	1	1	1	1	1	1	1	1	1	9	9	10
yeps_YPTB0862	4	8	4	11	1	1	4	9	8	11	11	3	1	1	1	11	1	5	4	4	1	1	1	4	1	1	1	1	1	1	1	1	11	11	4
yeps_YPTB0863	1	3	1	4	1	1	1	1	3	4	4	1	1	1	1	4	1	2	1	1	1	1	1	1	1	1	1	1	1	1	1	1	4	4	1
yeps_YPTB0866	4	8	4	9	1	1	4	4	8	9	9	3	1	1	1	9	1	5	4	4	1	1	1	4	1	1	1	1	1	1	1	1	9	9	4
yeps_YPTB0867	1	3	1	4	1	1	1	1	3	4	4	3	7	1	1	4	1	4	1	1	1	1	1	1	1	1	1	1	1	1	1	1	4	4	1
yeps_YPTB0868	1	5	1	4	1	1	1	6	5	4	4	3	1	1	1	4	1	4	1	1	1	1	1	1	1	1	1	1	1	1	1	1	4	4	1
yeps_YPTB0869	1	4	1	6	1	1	1	5	4	6	6	3	1	1	1	6	1	1	1	1	1	1	1	1	1	1	1	1	1	1	1	1	6	6	1
yeps_YPTB0881	4	8	4	12	1	1	4	9	8	11	12	1	1	5	1	11	5	4	4	4	1	1	1	4	1	1	1	1	1	1	1	1	12	12	4
yeps_YPTB0883	4	1	4	10	1	1	4	8	52	10	10	3	1	1	1	10	1	11	4	4	1	1	1	4	1	1	1	1	1	1	1	1	10	10	4
yeps_YPTB0884	2	1	2	1	1	1	2	2	1	1	1	2	1	1	1	1	1	1	2	2	1	1	1	2	1	1	1	1	1	1	1	1	1	1	2
yeps_YPTB0885	3	4	3	5	1	1	3	1	4	5	5	1	1	1	1	5	1	1	3	3	1	1	1	3	1	1	1	1	1	1	1	1	5	5	3
yeps_YPTB0886	3	6	3	8	1	1	3	7	6	8	8	1	1	1	1	8	1	4	3	3	1	1	1	3	1	1	1	1	1	1	1	1	8	8	3
yeps_YPTB0888	1	1	1	1	1	1	1	1	1	1	1	1	1	1	1	1	1	1	1	1	1	1	1	1	1	1	1	1	1	1	1	1	1	1	1
yeps_YPTB0891	1	1	1	1	1	1	1	1	1	1	1	1	1	1	1	1	1	1	1	1	1	1	1	1	1	1	1	1	1	1	1	1	1	1	1
yeps_YPTB0893	4	8	4	8	1	1	4	4	8	8	8	3	1	1	1	8	1	4	4	4	1	1	1	4	1	1	1	1	1	1	1	1	8	8	4
yeps_YPTB0912	1	1	1	1	1	1	1	1	1	1	1	1	2	2	1	1	2	1	1	1	1	1	1	1	2	2	1	1	1	1	1	2	1	1	1
yeps_YPTB0914	10	3	3	3	1	1	3	31	3	3	3	1	3	3	183	3	3	3	3	3	1	1	1	28	3	3	1	1	1	1	50	28	180	3	3
yeps_YPTB0916	1	3	1	7	1	1	1	1	3	7	7	1	1	1	1	7	1	8	1	1	1	1	1	1	1	1	1	1	1	1	1	1	7	7	1
yeps_YPTB0917	1	1	1	1	1	1	1	1	1	1	1	1	1	1	1	1	1	1	1	1	1	1	1	1	1	1	1	1	1	1	1	1	1	1	1
yeps_YPTB0918	3	7	3	4	1	1	3	3	7	4	4	1	1	1	1	4	1	4	3	3	1	1	1	3	1	1	1	1	1	1	1	1	4	4	3
yeps_YPTB0923	3	5	3	5	1	1	3	1	15	5	5	1	1	1	1	5	1	1	3	3	1	1	1	3	1	1	1	1	1	1	1	1	5	5	3
yeps_YPTB0927	1	7	1	9	1	1	1	8	7	9	9	3	1	1	1	9	1	5	1	1	1	1	1	1	1	1	1	1	1	1	1	1	9	9	1
yeps_YPTB0929	1	1	1	1	1	1	1	1	1	1	1	1	1	1	1	1	1	1	1	1	1	1	1	1	1	1	1	1	1	1	1	1	1	1	1
yeps_YPTB0935	1	1	1	1	1	1	1	1	1	1	1	1	1	1	1	1	1	1	1	1	1	1	1	1	1	1	1	1	1	1	1	1	1	1	1
yeps_YPTB0937	3	5	3	5	1	1	3	6	5	5	5	3	1	1	1	5	1	3	3	3	1	1	1	3	1	1	1	1	1	1	1	1	5	5	3
yeps_YPTB0938	3	1	3	6	1	1	3	1	1	6	6	2	1	1	1	6	1	4	3	3	1	1	1	3	1	1	1	1	1	1	1	1	6	6	3
yeps_YPTB0939	4	1	4	7	1	1	4	7	1	7	7	3	1	1	1	7	1	4	4	4	1	1	1	4	1	1	1	1	1	1	1	1	7	7	4
yeps_YPTB0940	3	5	3	7	1	1	3	6	5	7	7	2	1	1	1	7	1	4	3	3	1	1	1	3	1	1	1	1	1	1	1	1	7	7	3
yeps_YPTB0941	1	1	1	1	1	1	1	1	1	1	1	1	1	1	1	1	1	1	1	1	1	1	1	1	1	1	1	1	1	1	1	1	1	1	1
yeps_YPTB0942	1	1	1	1	1	1	1	1	1	1	1	1	1	1	1	1	1	1	1	1	1	1	1	1	1	1	1	1	1	1	1	1	1	1	1
yeps_YPTB0943	8	6	4	7	1	1	4	7	6	7	7	3	1	1	1	7	1	5	4	4	1	1	1	4	1	1	1	1	1	1	1	1	7	7	4
yeps_YPTB0944	3	3	3	3	1	1	3	3	3	3	3	3	1	1	1	3	1	3	3	3	1	1	1	3	1	1	1	1	1	1	1	1	3	3	3
yeps_YPTB0945	4	2	4	2	1	1	4	2	2	2	2	3	1	1	1	2	1	2	4	4	1	1	1	4	1	1	1	1	1	1	1	1	2	2	4
yeps_YPTB0946	1	2	1	2	1	1	1	2	2	3	2	1	1	1	1	3	1	2	1	1	1	1	1	1	1	1	1	1	1	1	1	1	2	2	1
yeps_YPTB0949	2	3	2	2	1	1	2	2	2	2	2	2	1	1	1	2	1	2	2	2	1	1	1	2	1	1	1	1	1	1	1	1	2	2	2
yeps_YPTB0954	1	4	1	5	1	1	1	1	4	5	5	1	1	1	1	5	1	2	1	1	1	1	1	1	1	1	1	1	1	1	1	1	5	5	1
yeps_YPTB0956	1	1	1	1	1	1	1	1	1	2	1	1	1	1	1	2	1	1	1	1	1	1	1	1	1	1	1	1	1	1	1	1	1	1	1
yeps_YPTB0957	1	1	1	1	1	1	1	1	1	1	1	1	1	1	1	1	1	1	1	1	1	1	1	1	1	1	1	1	1	1	1	1	1	1	1
yeps_YPTB0965	1	2	1	1	1	1	1	3	2	4	1	1	1	1	1	4	1	1	1	1	1	1	1	1	1	1	1	1	1	1	1	1	1	1	1
yeps_YPTB0967	4	8	4	11	1	1	4	9	8	11	11	3	1	1	1	11	1	4	4	4	1	1	1	4	1	1	1	1	1	1	1	1	11	11	4
yeps_YPTB0968	5	7	5	8	5	5	26	3	7	8	8	3	5	5	5	8	5	5	5	5	5	5	5	5	5	5	5	5	5	5	5	5	8	8	5
yeps_YPTB0973	1	1	1	1	1	1	1	1	1	1	1	1	1	1	1	1	1	1	1	1	1	1	1	1	1	1	1	1	1	1	1	1	1	1	1
yeps_YPTB0974	2	1	2	5	1	1	2	4	1	5	5	1	1	1	1	5	1	2	2	2	1	1	1	2	1	1	1	1	1	1	1	1	5	5	2
yeps_YPTB0975	3	3	3	3	1	1	3	4	3	3	3	3	1	1	1	3	1	3	3	3	1	1	1	3	1	1	1	1	1	1	1	1	3	3	3
yeps_YPTB0976	1	4	1	1	1	1	1	6	4	7	1	1	1	1	1	7	1	1	1	1	1	1	1	1	1	1	1	1	1	1	1	1	1	1	1
yeps_YPTB0986	1	1	1	5	1	1	1	3	1	5	5	1	1	1	1	5	1	1	1	1	1	1	1	1	1	1	1	1	1	1	1	1	5	5	1
yeps_YPTB0992	3	7	5	9	1	1	5	3	7	9	9	3	4	4	1	9	4	3	5	5	1	1	1	5	4	4	1	1	1	1	1	4	9	9	3
yeps_YPTB1024	2	6	2	2	1	1	2	2	6	2	2	2	1	1	1	2	1	2	2	2	1	1	1	2	1	1	1	1	1	1	1	1	2	2	2
yeps_YPTB1026	3	3	3	2	1	1	3	4	3	2	2	1	1	1	1	2	1	3	3	3	1	1	1	3	1	1	1	1	1	1	1	1	2	2	3
yeps_YPTB1028	2	2	2	2	1	1	2	2	2	4	2	1	1	1	1	4	1	2	2	2	1	1	1	2	1	1	1	1	1	1	1	1	2	2	2
yeps_YPTB1030	4	8	4	14	1	1	4	13	8	12	14	3	1	1	1	12	1	4	95	4	1	1	1	4	1	1	1	1	1	1	1	1	14	14	4
yeps_YPTB1031	5	8	5	13	1	1	5	10	8	12	13	3	1	1	1	12	1	5	5	5	1	1	1	5	1	1	1	1	1	1	1	1	13	13	5
yeps_YPTB1032	4	3	4	3	1	1	4	5	3	3	3	3	1	1	1	3	1	4	4	4	1	1	1	4	1	1	1	1	1	1	1	1	3	3	4
yeps_YPTB1035	11	7	4	10	1	1	4	8	7	10	10	3	1	1	1	10	1	12	4	4	1	1	1	4	1	1	1	1	1	1	1	1	10	10	4
yeps_YPTB1036	1	1	1	1	1	1	1	1	1	1	1	1	1	1	1	1	1	1	1	1	1	1	1	1	1	1	1	1	1	1	1	1	1	1	1
yeps_YPTB1037	1	1	1	2	1	1	1	1	1	2	2	1	1	1	1	2	1	2	1	1	1	1	1	1	1	1	1	1	1	1	1	1	2	2	1
yeps_YPTB1038	1		1	1	1	1	1	1	2	1	1	1	1	1	1	1	1	1	1	1	1	1	1	1	1	1	1	1	1	1	1	1	1	1	1
yeps_YPTB1040	4		4	7	1	1	4	8	7	10	7	3	1	1	1	10	1	5	4	4	1	1	1	4	1	1	1	1	1	1	1	1	7	7	4
yeps_YPTB1041	1		1	1	1	1	1	1	1	1	1	1	1	1	1	1	1	1	1	1	1	1	1	1	1	1	1	1	1	1	1	1	1	1	1
yeps_YPTB1043	3		3		1	1	3	2	3	2		3	1	1	1	2	1	3	3	3	1	1	1	3	1	1	1	1	1	1	1	1			3
yeps_YPTB1044	1		1	3	1	1	1	1	3	3	3	3	1	1	1	3	1	4	1	1	1	1	1	1	1	1	1	1	1	1	1	1	3	3	1
yeps_YPTB1045	3		3	8	1	1	3	3	6	8	8	1	1	1	1	8	1	3	3	3	1	1	1	3	1	1	1	1	1	1	1	1	8	8	3
yeps_YPTB1047	3		3	10	1	1	3	9	7	10	10	1	1	1	1	10	1	3	3	3	1	1	1	3	1	1	1	1	1	1	1	1	10	10	3
yeps_YPTB1048	3	7	3	10	1	1	3	8	7	9	10	1	5	5	1	9	5	3	3	3	1	1	1	3	5	5	1	1	1	1	1	5	10	10	3
yeps_YPTB1050	1	1	1	1	1	1	1	3	1	1	1	1	1	1	1	1	1	1	1	1	1	1	1	1	1	1	1	1	1	1	1	1	1	1	1
yeps_YPTB1051	2	5		4	1	1	2	4	5	4	4	3	1	1	1	4	1	2	2	2	1	1	1	2	10	10	1	1	1	1	1	10	4	4	2
yeps_YPTB1053	4	7	4	7	1	1	4	1	7	7	7	3	1	1	1	7	1	4	4	4	1	1	1	4	1	1	1	1	1	1	1	1	7	7	4
yeps_YPTB1054	5		5	10	1	1	5	9	8	10	10	3	1	1	1	10	1	5	5	5	1	1	1	5	1	1	1	1	1	1	1	1	10	10	5
yeps_YPTB1056	1	2	1	3	1	1	1	1	1	2	2	3	1	1	1	2	1	2	1	1	1	1	1	1	1	1	1	1	1	1	1	1	3	3	1
yeps_YPTB1057	4	7	4	13	1	59	4	10	7	12	13	1	1	1	59	12	1	4	4	4	59	59	59	4	1	1	59	59	59	59	59	1	13	13	4
yeps_YPTB1068	5	8	5	7	1	5	5	10	9	7	7	3	1	1	5	7	1	12	5	5	5	5	5	5	1	1	5	5	5	5	5	1	7	7	5
yeps_YPTB1071	4	7	4	11	1	4	4	9	7	11	11	3	1	1	4	11	1	4	4	4	4	4	4	4	1	1	4	4	4	4	4	1	11	11	4
yeps_YPTB1073	3	7	3	9	1	3	3	8	7	9	9	1	1	1	3	9	1	4	3	3	3	3	3	3	1	1	3	3	3	3	3	1	9	9	3
yeps_YPTB1074	1	3	1	4	1	1	1	1	1	4	4	1	1	1	1	4	1	1	1	1	1	1	1	1	1	1	1	1	1	1	1	1	4	4	1
yeps_YPTB1075	3	6	3	7	1	3	3	3	6	7	7	3	1	1	3	7	1	8	3	3	3	3	3	3	1	1	3	3	3	3	3	1	7	7	3
yeps_YPTB1076	1	2	1	8	1	1	1	6	2	8	8	3	1	1	1	8	1	4	1	1	1	1	1	1	1	1	1	1	1	1	1	1	8	8	1
yeps_YPTB1082	3	7	3	1	1	1	3	8	7	1	1	3	1	1	1	1	1	4	3	3	1	1	1	3	1	1	1	1	1	1	1	1	1	1	3
yeps_YPTB1083	2	5	2	2	1	1	2	2	5	2	2	2	1	1	1	2	1	3	2	2	1	1	1	2	1	1	1	1	1	1	1	1	2	2	2
yeps_YPTB1086	1	1	1	1	1	1	1	1	1	1	1	1	1	1	1	1	1	1	1	1	1	1	1	1	1	1	1	1	1	1	1	1	1	1	1
yeps_YPTB1087	1	1	1	1	1	1	1	1	1	1	1	1	1	1	1	1	1	1	1	1	1	1	1	1	1	1	1	1	1	1	1	1	1	1	1
yeps_YPTB1089	1	4	1	2	1	1	1	3	4	2	2	3	1	1	1	2	1	6	1	1	1	1	1	1	1	1	1	1	1	1	1	1	2	2	1
yeps_YPTB1090	1	1	1	1	1	1	1	1	1	1	1	1	1	1	1	1	1	1	1	1	1	1	1	1	1	1	1	1	1	1	1	1	1	1	1
yeps_YPTB1092	1	5	1	6	1	1	1	1	5	6	6	1	1	1	1	6	1	3	1	1	1	1	1	1	1	1	1	1	1	1	1	1	6	6	1
yeps_YPTB1093	1	1	1	1	1	1	1	1	1	1	1	1	1	1	1	1	1	1	1	1	1	1	1	1	1	1	1	1	1	1	1	1	1	1	1
yeps_YPTB1094	1	8	1	4	1	1	1	4	8	4	4	3	4	4	1	4	4	5	1	1	1	1	1	1	4	4	1	1	1	1	1	4	4	4	1
yeps_YPTB1095	1	1	1	4	1	1	1	2	1	2	4	2	2	2	1	2	2	3	1	1	1	1	1	1	2	2	1	1	1	1	1	2	4	4	1
yeps_YPTB1099	2	1	2	1	1	1	2	2	1	3	1	1	1	1	1	3	17	2	2	2	1	1	1	2	1	1	1	1	1	1	1	1	1	1	2
yeps_YPTB1100	1	3	1	1	1	1	1	1	3	1	1	1	1	1	1	1	1	2	1	1	1	1	1	1	1	1	1	1	1	1	1	1	1	1	1
yeps_YPTB1101	1	4	1	5	1	1	1	1	4	5	5	3	3	3	1	5	3	1	1	1	1	1	1	1	3	3	1	1	1	1	1	3	5	5	1
yeps_YPTB1102	1	1	1	2	1	1	1	1	1	2	2	1	1	1	1	2	1	1	1	1	1	1	1	1	1	1	1	1	1	1	1	1	2	2	1
yeps_YPTB1107	1	1	1	2	1	1	1	1	1	2	2	1	1	1	1	2	1	1	1	1	1	1	1	1	1	1	1	1	1	1	1	1	2	2	1
yeps_YPTB1109	1	6	1	2	1	1	1	2	6	2	2	2	2	2	1	2	2	2	1	1	1	1	1	1	2	2	1	1	1	1	1	2	2	2	1
yeps_YPTB1111	2	1	2	2	1	1	2	5	1	2	2	1	1	1	1	2	1	3	2	2	1	1	1	2	1	1	1	1	1	1	1	1	2	2	2
yeps_YPTB1117	4	3	4	3	1	1	4	7	3	3	3	3	1	1	1	3	1	5	4	4	1	1	1	4	1	1	1	1	1	1	1	1	3	3	4
yeps_YPTB1118	4	4	4	9	1	1	4	8	4	9	9	3	1	1	1	9	1	6	4	4	1	1	1	4	1	1	1	1	1	1	1	1	9	9	4
yeps_YPTB1123	4	7	4	1	1	1	4	9	7	1	1	3	1	1	1	1	1	4	4	4	1	1	1	4	1	1	1	1	1	1	1	1	1	1	4
yeps_YPTB1124	1	1	1	1	1	1	1	1	1	1	1	1	1	1	1	1	1	1	1	1	1	1	1	1	1	1	1	1	1	1	1	1	1	1	1
yeps_YPTB1126	1	1	1	1	1	1	1	1	1	1	1	1	1	1	1	1	1	1	1	1	1	1	1	1	1	1	1	1	1	1	1	1	1	1	1
yeps_YPTB1136	1	3	1	1	1	1	1	1	3	1	1	1	1	1	1	1	1	1	1	1	1	1	1	1	1	1	1	1	1	1	1	1	1	1	1
yeps_YPTB1137	1	1	1	1	1	1	1	1	1	1	1	1	1	1	1	1	1	1	1	1	1	1	1	1	1	1	1	1	1	1	1	1	1	1	1
yeps_YPTB1138	3	3	3	3	1	1	3	3	3	3	3	3	1	1	1	3	1	3	3	3	1	1	1	3	1	1	1	1	1	1	1	1	3	3	3
yeps_YPTB1140	4	2	1	3	1	1	1	2	2	3	3	2	1	1	1	3	1	2	1	1	1	1	1	1	1	1	1	1	1	1	1	1	3	3	1
yeps_YPTB1142	1	1	1	1	1	1	1	6	1	1	1	1	1	1	1	1	1	1	1	1	1	1	1	1	1	1	1	1	1	1	1	1	1	1	1
yeps_YPTB1143	1	1	1	1	1	1	1	1	1	1	1	1	1	1	1	1	1	1	1	1	1	1	1	1	1	1	1	1	1	1	1	1	1	1	1
yeps_YPTB1152	3	5	3	2	1	1	3	6	5	2	2	2	1	1	1	2	1	2	3	3	1	1	1	3	1	1	1	1	1	1	1	1	2	2	3
yeps_YPTB1153	1	1	1	1	1	1	1	1	1	1	1	1	1	1	1	1	1	1	1	1	1	1	1	1	1	1	1	1	1	1	1	1	1	1	1
yeps_YPTB1154	2	3	2	2	1	1	2	4	3	2	2	1	1	1	1	2	1	2	2	2	1	1	1	2	1	1	1	1	1	1	1	1	2	2	2
yeps_YPTB1156	1	5	1	1	1	1	1	1	5	1	1	1	1	1	1	1	1	4	1	1	1	1	1	1	1	1	1	1	1	1	1	1	1	1	1
yeps_YPTB1160	1	1	1	1	1	1	1	1	1	1	1	1	1	1	1	1	1	1	1	1	1	1	1	1	1	1	1	1	1	1	1	1	1	1	1
yeps_YPTB1167	1	1	1	1	1	1	1	2	1	1	1	1	1	1	1	1	1	1	1	1	1	1	1	1	1	1	1	1	1	1	1	1	1	1	1
yeps_YPTB1168	2	4	2	2	1	1	2	2	4	6	2	2	1	1	1	6	1	3	2	2	1	1	1	2	1	1	1	1	1	1	1	1	2	2	2
yeps_YPTB1170	3	1	3	1	1	1	3	1	1	1	1	1	1	1	1	1	1	1	3	3	1	1	1	3	1	1	1	1	1	1	1	1	1	1	3
yeps_YPTB1171	7	6	7	2	1	1	7	1	6	2	2	3	1	1	1	2	1	2	4	4	1	1	1	4	1	1	1	1	1	1	1	1	2	2	7
yeps_YPTB1173	59	8	4	10	1	1	4	9	8	10	10	3	1	1	1	10	1	5	4	4	1	1	1	4	1	1	1	1	1	1	1	1	10	10	4
yeps_YPTB1174	1	4	1	1	1	1	1	1	4	1	1	1	1	1	1	1	1	1	1	1	1	1	1	1	1	1	1	1	1	1	1	1	1	1	1
yeps_YPTB1175	1	1	1	1	1	1	1	1	1	1	1	1	1	1	1	1	1	1	1	1	1	1	1	1	1	1	1	1	1	1	1	1	1	1	1
yeps_YPTB1176	1	1	1	3	1	1	1	3	1	3	3	2	1	1	1	3	1	3	1	1	1	1	1	1	1	1	1	1	1	1	1	1	3	3	1
yeps_YPTB1178	11	8	5	10	1	1	5	9	8	10	10	3	1	1	1	10	1	14	5	5	1	1	1	5	1	1	1	1	1	1	1	1	10	10	5
yeps_YPTB1179	3	7	3	10	1	1	3	8	7	10	10	1	1	1	1	10	1	4	3	3	1	1	1	3	1	1	1	1	1	1	1	1	10	10	3
yeps_YPTB1180	3	7	3	9	1	1	3	8	7	9	9	1	1	1	1	9	1	4	3	3	1	1	1	3	1	1	1	1	1	1	1	1	9	9	3
yeps_YPTB1182	1	1	1	1	1	1	1	1	1	1	1	1	1	1	1	1	1	1	1	1	1	1	1	1	1	1	1	1	1	1	1	1	1	1	1
yeps_YPTB1183	4	9	4	11	1	1	4	3	9	11	11	3	1	1	1	11	1	5	4	4	1	1	1	33	1	1	1	1	1	1	1	1	11	11	4
yeps_YPTB1184	4	6	4	36	1	1	4	7	6	8	8	3	1	1	1	8	1	9	4	4	1	1	1	4	1	1	1	1	1	1	1	1	8	8	4
yeps_YPTB1185	1	4	1	1	1	1	1	5	4	1	1	3	1	1	1	1	1	1	1	1	1	1	1	1	1	1	1	1	1	1	1	1	1	1	1

yeps_YPTB1186	3	3	3	5	1	1	3	3	3	5	5	3	1	1	1	5	1	3	3	3	1	1	1	3	1	1	1	1	1	1	1	1	5	5	3
yeps_YPTB1187	3	7	3	10	1	1	3	7	7	10	10	1	1	1		10	1	4	3	3	1	1	1	3	1	1	1	1	1	1	1	1	10	10	3
yeps_YPTB1189	3	5	3	6	1	1	3	6	5	6	6	2	1	1	1	6	1	3	3	3	1	1	1	3	1	1	1	1	1	1	1	1	6	6	3
yeps_YPTB1193	1	1	1	1	1	1	1	1	1	1	1	2	1	1	1	1	1	1	1	1	1	1	1	1	1	1	1	1	1	1	1	1	1	1	1
yeps_YPTB1197	1	1	1	2	1	1	1	1	1	2	2	1	1	1	1	2	1	3	1	1	1	1	1	1	1	1	1	1	1	1	1	1	2	2	1
yeps_YPTB1199	1	5	1	6	1	1	1	1	5	6	6	1	1	1	1	6	1	1	1	1	1	1	1	1	1	1	1	1	1	1	1	1	6	6	1
yeps_YPTB1201	1	5	1	6	1	1	1	4	5	6	6	3	4	4	1	6	4	1	1	1	1	1	1	1	4	4	1	1	1	1	1	4	6	6	1
yeps_YPTB1202	5	7	5	9	1	1	5	8	7	9	9	3	4	4	1	9	4	1	5	5	1	1	1	5	4	4	1	1	1	1	1	4	9	9	5
yeps_YPTB1203	2	4	2	3	1	1	2	3	4	3	3	2	3	3	1	3	3	1	2	2	1	1	1	2	3	3	1	1	1	1	1	3	3	3	2
yeps_YPTB1204	8	7	8	10	1	1	32	9	7	10	10	3	4	4	1	10	4	1	8	8	1	1	1	8	4	4	1	1	1	1	1	4	10	10	8
yeps_YPTB1205	5	7	5	10	1	1	5	3	7	9	10	3	5	5	1	9	5	1	5	5	1	1	1	5	5	5	1	1	1	1	1	5	10	10	5
yeps_YPTB1207	1	1	1	4	1	1	1	1	1	4	4	1	1	1	1	4	1	1	1	1	1	1	1	1	1	1	1	1	1	1	1	1	4	4	1
yeps_YPTB1208	2	2	2	3	1	1	2	2	2	3	3	2	1	1	1	3	1	1	2	2	1	1	1	2	1	1	1	1	1	1	1	1	3	3	2
yeps_YPTB1211	2	5	2	2	1	1	2	6	5	2	2	3	4	4	1	2	4	1	2	2	1	1	1	2	4	4	1	1	1	1	1	4	2	2	2
yeps_YPTB1220	3	3	3	1	1	1	3	2	3	2	1	2	2	2	1	2	2	1	3	3	1	1	1	3	2	2	1	1	1	1	1	2	1	1	3
yeps_YPTB1221	2	2	2	2	1	1	2	1	2	6	2	3	4	4	1	6	4	1	2	2	1	1	1	2	4	4	1	1	1	1	1	4	2	2	2
yeps_YPTB1222	1	1	1	1	1	1	1	2	1	1	1	1	1	1	1	1	1	1	1	1	1	1	1	1	1	1	1	1	1	1	1	1	1	1	1
yeps_YPTB1229	4	8	4	12	1	1	4	10	8	12	12	4	1	1	1	12	1	5	4	4	1	1	1	4	1	1	1	1	1	1	1	1	12	12	4
yeps_YPTB1231	1	1	1	4	1	1	1	2	1	4	4	1	1	1	1	4	1	4	1	1	1	1	1	1	1	1	1	1	1	1	1	1	4	4	1
yeps_YPTB1250	1	6	1	7	1	1	1	3	6	7	7	3	1	1	1	7	1	1	1	1	1	1	1	1	1	1	1	1	1	1	1	1	7	7	1
yeps_YPTB1251	1	1	1	1	1	1	1	1	1	1	1	1	1	1	1	1	1	1	1	1	1	1	1	1	1	1	1	1	1	1	1	1	1	1	1
yeps_YPTB1252	1	1	1	1	1	1	1	1	1	1	1	1	1	1	1	1	1	1	1	1	1	1	1	1	1	1	1	1	1	1	1	1	1	1	1
yeps_YPTB1255	1	1	1	1	1	1	1	4	1	1	1	1	1	1	1	1	1	1	5	5	1	1	1	5	1	1	1	1	1	1	1	1	1	1	1
yeps_YPTB1257	5	9	5	10	1	1	5	4	9	10	10	3	1	1	1	10	1	6	5	5	1	1	1	5	1	1	1	1	1	1	1	1	10	10	5
yeps_YPTB1258	1	1	1	1	1	1	1	1	1	1	1	1	1	1	1	1	1	1	1	1	1	1	1	1	1	1	1	1	1	1	1	1	1	1	1
yeps_YPTB1259	1	5	1	8	1	1	1	1	5	6	8	1	1	1	1	6	1	1	1	1	1	1	1	1	1	1	1	1	1	1	1	1	8	8	1
yeps_YPTB1263	5	8	5	11	1	1	5	9	8	11	11	3	1	1	1	11	1	1	5	5	1	1	1	5	1	1	1	1	1	1	1	1	11	11	5
yeps_YPTB1268	1	1	1	1	1	1	1	1	1	1	1	1	1	1	1	1	1	1	1	1	1	1	1	1	1	1	1	1	1	1	1	1	1	1	1
yeps_YPTB1270	4	8	4	7	1	1	4	9	8	7	7	3	1	1	1	7	1	5	4	4	1	1	1	4	1	1	1	1	1	1	1	20	7	7	4
yeps_YPTB1272	2	1	2	1	1	1	2	3	1	1	1	1	1	1	1	1	1	2	2	2	1	1	1	2	1	1	1	1	1	1	1	1	1	1	2
yeps_YPTB1273	2	1	2	4	1	1	2	2	1	3	4	1	1	1	1	3	1	1	2	2	1	1	1	2	1	1	1	1	1	1	1	1	4	4	2
yeps_YPTB1274	1	2	1	4	1	1	1	2	2	4	4	1	1	1	1	4	1	2	1	1	1	1	1	1	1	1	1	1	1	1	1	1	4	4	1
yeps_YPTB1276	1	1	1	1	1	1	1	1	1	1	1	1	1	1	1	1	1	4	1	1	1	1	1	1	1	1	1	1	1	1	1	1	1	1	1
yeps_YPTB1279	1	1	1	1	1	1	1	1	1	1	1	1	1	1	1	1	1	3	1	1	1	1	1	1	1	1	1	1	1	1	1	1	1	1	1
yeps_YPTB1283	3	3	3	4	1	1	3	3	3	4	4	3	3	3	1	4	3	1	3	3	1	1	1	3	3	3	1	1	1	1	1	3	4	4	3
yeps_YPTB1285	3	8	5	8	1	1	5	11	8	8	8	3	3	3	1	8	3	1	5	5	1	1	1	5	3	3	1	1	1	1	1	3	8	8	5
yeps_YPTB1286	1	6	1	8	1	1	1	3	6	8	8	1	1	1	1	8	1	1	1	1	1	1	1	1	1	1	1	1	1	1	1	1	8	8	1
yeps_YPTB1289	1	4	1	8	1	1	1	4	4	7	8	1	4	4	1	7	4	2	1	1	1	1	1	1	4	4	1	1	1	1	1	4	8	8	1
yeps_YPTB1290	1	1	1	1	1	1	1	6	1	1	1	1	1	1	1	1	1	1	1	1	1	1	1	1	1	1	1	1	1	1	1	1	1	1	1
yeps_YPTB1300	3	7	3	11	1	1	3	9	7	11	11	1	1	1	1	11	1	2	3	3	1	1	1	3	1	1	1	1	1	1	1	1	11	11	3
yeps_YPTB1301	3	2	3	7	1	1	3	2	2	7	7	1	1	1	1	7	1	4	3	3	1	1	1	3	1	1	1	1	1	1	1	1	7	7	3
yeps_YPTB1304	5	58	5	8	1	1	5	10	9	8	8	3	1	1	1	8	1	6	5	5	1	1	1	5	1	1	1	1	1	1	1	1	8	8	5
yeps_YPTB1305	1	3	1	2	1	1	1	1	3	2	2	1	1	1	1	2	1	1	1	1	1	1	1	1	1	1	1	1	1	1	1	1	2	2	1
yeps_YPTB1306	1	5	1	6	1	1	1	1	5	6	6	1	1	1	1	6	1	3	1	1	1	1	1	1	1	1	1	1	1	1	1	1	6	6	1
yeps_YPTB1307	4	8	4	12	1	1	4	9	8	11	12	3	1	1	1	11	1	7	4	4	1	1	1	4	1	1	1	1	1	1	1	1	12	12	4
yeps_YPTB1310	1	3	1	2	1	1	1	5	3	2	2	3	1	1	1	2	1	2	1	1	1	1	1	1	1	1	1	1	1	1	1	1	2	2	1
yeps_YPTB1311	1	8	1	2	1	1	1	9	8	11	2	3	3	3	1	11	3	4	1	1	1	1	1	1	3	3	1	1	1	1	1	3	2	2	1
yeps_YPTB1312	1	1	1	1	1	1	1	2	1	1	1	1	1	1	1	1	1	1	1	1	1	1	1	1	1	1	1	1	1	1	1	1	1	1	1
yeps_YPTB1313	1	3	1	2	1	1	1	2	3	2	2	2	2	2	1	2	2	2	1	1	1	1	1	1	2	2	1	1	1	1	1	2	2	2	1
yeps_YPTB1314	1	6	1	1	1	1	1	7	6	8	1	3	3	3	1	8	3	4	1	1	1	1	1	1	3	3	1	1	1	1	1	3	1	1	1
yeps_YPTB1315	1	7	1	9	1	1	1	3	7	9	9	3	3	3	1	9	3	4	1	1	1	1	1	1	3	3	1	1	1	1	1	3	9	9	1
yeps_YPTB1317	1	1	1	1	1	1	1	6	1	1	1	1	1	1	1	1	1	1	1	1	1	1	1	1	1	1	1	1	1	1	1	1	1	1	1
yeps_YPTB1318	2	2	2	2	1	1	2	1	2	2	2	1	1	1	1	2	1	1	2	2	1	1	1	2	1	1	1	1	1	1	1	1	2	2	2
yeps_YPTB1329	3	3	3	9	1	1	3	1	3	9	9	3	1	1	1	9	1	5	3	3	1	1	1	3	1	1	1	1	1	1	1	1	9	9	3
yeps_YPTB1342	1	6	1	5	1	1	1	1	6	5	5	1	1	1	1	5	1	3	1	1	1	1	1	1	1	1	1	1	1	1	1	1	5	5	1
yeps_YPTB1343	1	3	1	2	1	1	1	4	3	2	2	1	1	1	1	2	1	2	1	1	1	1	1	1	1	1	1	1	1	1	1	1	2	2	1
yeps_YPTB1347	1	7	1	10	1	1	1	9	7	10	10	4	3	3	1	10	3	5	1	1	1	1	1	1	3	3	1	1	1	1	1	58	10	10	1
yeps_YPTB1348	1	1	1	4	1	1	1	1	1	4	4	2	1	1	1	4	1	1	1	1	1	1	1	1	1	1	1	1	1	1	1	1	4	4	1
yeps_YPTB1355	4	4	4	4	1	1	4	4	4	4	4	3	1	1	1	4	1	4	4	4	1	1	1	4	1	1	1	1	1	1	1	1	4	4	4
yeps_YPTB1356	4	5	4	7	1	1	4	8	5	7	7	3	1	1	1	7	1	5	4	4	1	1	1	4	1	1	1	1	1	1	1	1	7	7	4
yeps_YPTB1359	1	1	1	1	1	1	1	1	1	1	1	1	1	1	1	1	1	1	1	1	1	1	1	1	1	1	1	1	1	1	1	1	1	1	1
yeps_YPTB1360	1	1	1	1	1	1	1	1	1	1	1	1	1	1	1	1	1	1	1	1	1	1	1	1	1	1	1	1	1	1	1	1	1	1	1
yeps_YPTB1365	2	1	2	1	1	1	2	4	1	1	1	3	1	1	1	1	1	2	2	2	1	1	1	2	1	1	1	1	1	1	1	1	1	1	2
yeps_YPTB1366	10	6	3	2	1	1	3	8	6	2	2	2	1	1	1	2	1	3	3	3	1	1	1	3	1	1	1	1	1	1	1	1	2	2	3
yeps_YPTB1369	4	8	4	12	1	1	4	1	8	11	12	3	1	1	1	11	1	4	4	4	1	1	1	4	1	1	1	1	1	1	1	1	12	12	4
yeps_YPTB1370	5	9	5	11	1	1	5	10	9	11	11	3	7	7	1	11	7	5	5	5	1	1	1	5	7	7	1	1	1	1	1	7	11	11	5
yeps_YPTB1371	2	1	2	5	1	1	2	4	1	5	5	1	1	1	1	5	1	2	2	2	1	1	1	2	1	1	1	1	1	1	1	1	5	5	2
yeps_YPTB1372	1	4	1	1	1	1	1	1	4	1	1	1	1	1	1	1	1	1	1	1	1	1	1	1	1	1	1	1	1	1	1	1	1	1	1
yeps_YPTB1374	4	6	4	7	1	1	4	7	6	7	7	3	1	1	1	7	1	4	4	4	1	1	1	4	1	1	1	1	1	1	1	1	7	7	4
yeps_YPTB1375	4	6	4	2	1	1	4	7	6	2	2	1	1	1	1	2	1	4	4	4	1	1	1	4	1	1	1	1	1	1	1	1	2	2	4
yeps_YPTB1378	3	4	3	4	1	1	3	4	4	4	4	1	1	1	1	4	1	4	3	3	1	1	1	3	1	1	1	1	1	1	1	1	4	4	3
yeps_YPTB1379	1	4	1	6	1	1	1	5	4	6	6	1	1	1	1	6	1	1	1	1	1	1	1	1	25	1	1	1	1	1	1	1	6	6	1
yeps_YPTB1380	1	1	1	1	1	20	1	3	1	1	1	1	1	1	20	1	1	1	1	1	20	20	20	1	1	1	20	20	20	20	20	1	1	1	1
yeps_YPTB1382	3	4	3	8	1	1	3	7	4	8	8	3	1	1	1	8	1	4	3	3	1	1	1	3	1	1	1	1	1	1	1	1	8	8	3
yeps_YPTB1385	3	2	3	5	1	1	3	1	2	5	5	1	1	1	1	5	1	2	3	3	1	1	1	3	1	1	1	1	1	1	1	1	5	5	3
yeps_YPTB1388	1	1	1	1	1	1	1	8	1	1	1	1	1	1	1	1	1	1	1	1	1	1	1	1	1	1	1	1	1	1	1	1	1	1	1
yeps_YPTB1389	1	5	1	6	1	1	1	1	5	6	6	1	1	1	1	6	1	1	1	1	1	1	1	1	1	1	1	1	1	1	1	1	6	6	1
yeps_YPTB1392	1	1	1	1	1	1	1	1	1	1	1	1	1	1	1	1	1	1	1	1	1	1	1	1	1	1	1	1	1	1	1	1	1	1	1
yeps_YPTB1393	1	1	1	1	1	1	1	1	1	1	1	1	1	1	1	1	1	1	1	1	1	1	1	1	1	1	1	1	1	1	1	1	1	1	1
yeps_YPTB1396	3	1	3	2	1	1	3	3	1	2	2	1	1	1	1	2	1	4	3	3	1	1	1	3	1	1	1	1	1	1	1	1	2	2	3
yeps_YPTB1402	1	2	1	1	1	1	1	1	2	3	1	1	1	1	1	3	1	1	1	1	1	1	1	1	1	1	1	1	1	1	1	1	1	1	1
yeps_YPTB1404	1	3	1	30	1	1	1	1	3	1	1	2	1	1	1	1	1	1	1	1	1	1	1	1	1	1	1	1	1	1	1	1	1	1	1
yeps_YPTB1415	1	5	1	3	1	1	1	1	5	6	3	2	3	3	1	6	3	3	1	1	1	1	1	1	3	3	1	1	1	1	1	3	3	3	1
yeps_YPTB1419	1	7	1	9	1	1	33	8	7	5	9	29	5	5	1	5	5	5	1	1	1	1	1	1	5	5	1	1	1	1	1	5	9	9	1
yeps_YPTB1420	1	1	1	6	1	1	1	1	1	6	6	1	3	3	1	6	3	4	1	1	1	1	1	1	3	3	1	1	1	1	1	3	6	6	1
yeps_YPTB1421	1	1	1	3	1	1	1	1	1	3	3	1	1	1	1	3	1	2	1	1	1	1	1	1	1	1	1	1	1	1	1	1	3	3	1
yeps_YPTB1423	1	1	1	1	1	1	1	1	1	1	1	1	1	1	1	1	1	2	1	1	1	1	1	1	1	1	1	1	1	1	1	1	1	1	1
yeps_YPTB1424	1	1	1	1	1	1	1	1	1	1	1	1	1	1	1	1	1	1	1	1	1	1	1	1	1	1	1	1	1	1	1	1	1	1	1
yeps_YPTB1425	1	4	1	1	1	1	1	1	5	1	1	1	1	1	1	1	1	2	1	1	1	1	1	1	1	1	1	1	1	1	1	1	1	1	1
yeps_YPTB1426	1	4	1	7	1	1	1	4	4	7	7	1	1	1	1	7	1	3	1	1	1	1	1	1	1	1	1	1	1	1	1	1	7	7	1
yeps_YPTB1427	1	3	1	4	1	1	1	1	3	4	4	1	1	1	1	4	1	1	1	1	1	1	1	1	1	1	1	1	1	1	1	1	4	4	5
yeps_YPTB1429	1	1	1	1	1	1	1	1	1	1	1	1	1	1	1	1	1	1	1	1	1	1	1	1	1	1	1	1	1	1	1	1	1	1	1
yeps_YPTB1437	2	2	3	2	1	1	3	5	2	2	2	1	1	1	1	2	1	4	3	3	1	1	1	3	1	1	1	1	1	1	1	1	2	2	2
yeps_YPTB1438	4	2	4	13	9	9	43	11	2	13	13	3	3	3	9	13	3	15	4	4	9	9	9	4	3	3	9	9	9	9	9	3	13	13	4
yeps_YPTB1440	1	1	1	1	1	1	1	1	1	1	1	1	1	1	1	1	1	1	1	1	1	1	1	1	1	1	1	1	1	1	1	1	1	1	1
yeps_YPTB1443	1	8	1	10	1	1	1	9	8	10	10	3	4	4	1	10	4	5	1	1	1	1	1	1	4	4	1	1	1	1	1	4	10	10	1
yeps_YPTB1450	1	1	1	1	1	1	1	1	1	1	1	1	1	18	1	1	1	1	1	1	1	1	1	1	1	1	1	1	1	1	1	1	1	1	1
yeps_YPTB1451	1	3	1	1	1	1	1	4	3	1	1	1	1	44	1	1	1	2	1	1	1	1	1	1	1	1	1	1	1	1	1	1	1	1	1
yeps_YPTB1456	49	7	1	10	1	1	1	8	7	9	10	3	3	3	1	9	3	4	1	1	1	1	1	1	3	3	1	1	1	1	1	3	10	10	1
yeps_YPTB1457	4	2	4	2	1	4	4	2	2	2	2	2	3	3	4	2	3	2	4	4	4	4	4	4	3	3	4	4	4	4	4	3	2	2	4
yeps_YPTB1459	1	2	1	1	1	1	1	1	2	1	1	1	1	1	1	1	1	1	1	1	1	1	1	1	1	1	1	1	1	1	1	1	1	1	1
yeps_YPTB1462	1	1	1	1	1	1	1	1	1	1	1	1	1	1	1	1	1	1	1	1	1	1	1	1	1	1	1	1	1	1	1	1	1	1	1
yeps_YPTB1464	1	1	1	1	1	1	1	1	1	1	1	1	1	1	1	1	1	1	1	1	1	1	1	1	1	1	1	1	1	1	1	1	1	1	1
yeps_YPTB1465	1	1	1	1	1	1	1	1	1	1	1	1	1	1	1	1	1	2	1	1	1	1	1	1	1	1	1	1	1	1	1	1	1	1	1
yeps_YPTB1478	1	1	1		1	1	1	4	1	3	3	3	1	1	1	3	1	4	1	1	1	1	1	1	1	1	1	1	1	1	1	1	3	3	1
yeps_YPTB1479	1	3	1		1	1	1	1	3	1	1	1	1	1	1	1	1	1	1	1	1	1	1	1	1	1	1	1	1	1	1	1	1	1	1
yeps_YPTB1480	1	1	1		1	1	1	1	1	1	1	1	1	1	1	1	1	1	1	1	1	1	1	1	1	1	1	1	1	1	1	1	1	1	1
yeps_YPTB1481	2	2	2		1	1	2	3	2	2	2	1	1	1	1	2	1	3	2	2	1	1	1	2	1	1	1	1	1	1	1	1	2	2	2
yeps_YPTB1517	4	7	4	8	1	1	4	6	7	8	8	1	1	1	1	8	1	4	4	4	1	1	1	4	1	1	1	1	1	1	1	1	8	8	4
yeps_YPTB1518	4	6	4	1	1	1	4	4	6	1	1	3	1	1	1	1	1	4	4	4	1	1	1	4	18	18	1	1	1	1	1	18	1	1	4
yeps_YPTB1520	2	2	2	4	1	1	2	2	2	4	4	1	1	1	1	4	1	2	2	2	1	1	1	2	1	1	1	1	1	1	1	1	4	4	2
yeps_YPTB1521	4	8	4	9	1	1	4	8	8	9	9	3	1	1	1	9	1	4	4	4	1	1	1	4	1	1	1	1	1	1	1	1	9	9	4
yeps_YPTB1522	1	1	1	5	1	1	1	1	1	5	5	1	1	1	1	5	1	1	1	1	1	1	1	1	1	1	1	1	1	1	1	1	5	5	1
yeps_YPTB1526	2	2	2	6	1	1	2	5	4	6	6	1	1	1	1	6	1	2	2	2	1	1	1	2	1	1	1	1	1	1	1	1	6	6	2
yeps_YPTB1527	2	2	2	4	1	1	2	2	2	4	4	3	1	1	1	4	1	2	2	2	1	1	1	2	1	1	1	1	1	1	1	1	4	4	2
yeps_YPTB1528	3	4	3	6	1	1	3	2	4	6	6	2	2	2	1	6	2	3	3	3	1	1	1	3	2	2	1	1	1	1	1	2	6	6	3
yeps_YPTB1536	2	1	2	1	1	1	2	1	1	1	1	1	1	1	1	1	1	1	2	2	1	1	1	2	1	1	1	1	1	1	1	1	1	1	2
yeps_YPTB1538	1	2	1	2	1	1	1	5	2	2	2	1	1	1	1	2	1	3	1	1	1	1	1	1	1	1	1	1	1	1	1	1	2	2	1
yeps_YPTB1542	3	6	3	9	1	1	3	7	6	9	9	1	1	1	1	9	1	3	3	3	1	1	1	3	1	1	1	1	1	1	1	1	9	9	3
yeps_YPTB1543	1	1	1	1	1	1	1	1	1	1	1	3	1	1	1	1	1	1	1	1	1	1	1	1	1	1	1	1	1	1	1	1	1	1	1
yeps_YPTB1548	3	6	3	7	1	1	3	7	6	7	7	2	1	1	1	7	1	3	3	3	1	1	1	3	1	1	1	1	1	1	1	1	7	7	3
yeps_YPTB1552	4	6	4	7	1	1	4	7	6	7	7	3	1	1	1	7	1	4	4	4	1	1	1	4	1	1	1	1	1	1	1	1	7	7	4
yeps_YPTB1556	10	7	3	9	1	1	3	8	7	9	9	1	1	1	1	9	1	4	3	3	1	1	1	3	1	1	1	1	1	1	1	1	9	9	3
yeps_YPTB1560	2	8	9	10	1	1	9	1	8	10	10	3	1	6	1	10	6	4	2	2	1	1	1	2	1	1	1	1	1	1	1	1	10	10	2
yeps_YPTB1561	4	8	4	1	1	1	4	1	8	1	1	3	1	1	1	1	1	10	4	4	1	1	1	4	1	1	1	1	1	1	1	1	1	1	4
yeps_YPTB1562	4	6	4	6	1	1	4	1	6	6	6	3	1	1	1	6	1	3	4	4	1	1	1	4	1	1	1	1	1	1	1	1	6	6	4
yeps_YPTB1563	3	4	3	3	1	1	3	4	4	3	3	3	1	1	1	3	1	3	3	3	1	1	1	3	1	1	1	1	1	1	1	1	3	3	3
yeps_YPTB1574	3	4	3	3	1	1	3	5	4	3	3	1	1	1	1	3	1	2	3	3	1	1	1	3	1	1	1	1	1	1	1	1	3	3	3
yeps_YPTB1575	1	4	1	7	1	1	1	5	4	7	7	1	1	1	1	7	1	3	1	1	1	1	1	1	1	1	1	1	1	1	1	1	7	7	1
yeps_YPTB1577	3	6	3	8	1	1	3	7	6	8	8	1	1	1	1	8	1	3	3	3	1	1	1	3	1	1	1	1	1	1	1	1	8	8	3
yeps_YPTB1579	4	7	4	1	1	1	4	8	7	1	1	3	1	1	1	1	1	11	4	4	1	1	1	4	1	1	1	1	1	1	1	1	1	1	4
yeps_YPTB1580	9	6	5	8	1	1	5	7	6	8	8	3	1	1	1	8	1	6	5	5	1	1	1	5	1	1	1	1	1	1	1	1	8	8	5
yeps_YPTB1604	3	4	3	4	1	1	3	4	4	4	4	1	1	1	1	4	1	4	3	3	1	1	1	3	1	1	1	1	1	1	1	1	4	4	3
yeps_YPTB1605	1	1	23	1	1	1	1	1	1	1	1	1	1	1	1	1	1	1	1	1	1	1	1	1	1	1	1	1	1	1	1	1	1	1	1
yeps_YPTB1609	4	7	4	5	1	1	4	4	7	5	5	1	1	1	1	5	1	5	4	4	1	1	1	4	1	1	1	1	1	1	1	1	5	5	4
yeps_YPTB1611	1	1	1	1	1	1	1	1	4	1	1	1	1	1	1	1	1	3	1	1	1	1	1	1	1	1	1	1	1	1	1	1	1	1	1
yeps_YPTB1612	3	1	3	1	1	1	3	4	1	1	1	1	1	1	1	1	1	3	3	3	1	1	1	3	1	1	1	1	1	1	1	1	1	1	3
yeps_YPTB1613	1	4	1	1	1	1	1	1	4	1	1	1	1	1	1	1	1	2	1	1	1	1	1	1	1	1	1	1	1	1	1	1	1	1	1
yeps_YPTB1633	1	1	1	1	1	1	1	3	1	1	1	1	1	1	1	1	1	1	1	1	1	1	1	1	1	1	1	1	1	1	1	1	1	1	1
yeps_YPTB1634	1	3	1	4	1	1	1	1	3	4	4	1	1	1	1	4	1	1	1	1	1	1	1	1	1	1	1	1	1	1	1	1	4	4	1
yeps_YPTB1635	4	8	4	11	1	1	4	1	8	11	11	1	1	1	1	11	1	4	4	4	1	1	1	4	1	1	1	1	1	1	1	1	11	11	4
yeps_YPTB1641	2	4	2	3	1	1	2	3	4	3	3	2	1	1	1	3	1	2	2	2	1	1	1	2	1	1	1	1	1	1	1	1	3	3	2
yeps_YPTB1642	1	1	1	2	1	1	1	1	1	2	2	1	1	1	1	2	1	1	1	1	1	1	1	1	1	1	1	1	1	1	1	1	2	2	1
yeps_YPTB1645	2	6	2	8	1	1	2	9	6	8	8	3	1	1	1	8	1	5	2	2	1	1	1	2	1	1	1	1	1	1	1	1	8	8	2
yeps_YPTB1646	1	1	1	1	1	1	1	1	1	1	1	1	1	1	1	1	1	1	1	1	1	1	1	1	1	1	1	1	1	1	1	1	1	1	1
yeps_YPTB1648	1	1	1	1	1	1	1	4	1	1	1	1	1	1	1	1	1	2	1	1	1	1	1	1	1	1	1	1	1	1	1	1	1	1	1
yeps_YPTB1649	1	4	1	1	1	1	1	5	4	1	1	1	1	1	1	1	1	2	1	1	1	1	1	1	1	1	1	1	1	1	1	1	1	1	1
yeps_YPTB1650	1	2	1	2	1	1	1	2	2	2	2	2	1	1	1	2	1	2	1	1	1	1	1	1	1	1	1	1	1	1	1	1	2	2	1
yeps_YPTB1652	1	6	1	4	1	1	1	4	6	4	4	2	1	1	1	4	1	1	1	1	1	1	1	1	1	1	1	1	1	1	1	1	4	4	1
yeps_YPTB1653	1	1	1	1	1	1	1	1	1	1	1	1	1	1	1	1	1	2	1	1	1	1	1	1	1	1	1	1	1	1	1	1	1	1	1
yeps_YPTB1654	1	1	1	1	1	1	1	1	1	1	1	1	1	1	1	1	1	1	1	1	1	1	1	1	1	1	1	1	1	1	1	1	1	1	1
yeps_YPTB1655	1	1	1	1	1	1	1	1	1	1	1	1	1	1	1	1	1	1	1	1	1	1	1	1	1	1	1	1	1	1	1	1	1	1	1
yeps_YPTB1669	1	1	1	2	1	1	1	1	1	2	2	1	1	1	1	2	1	2	1	1	1	1	1	1	1	1	1	1	1	1	1	1	2	2	1
yeps_YPTB1670	1	1	1	2	1	1	1	1	1	2	2	14	1	1	1	2	1	2	1	1	1	1	1	1	1	1	1	1	1	1	1	1	2	2	1
yeps_YPTB1674	1	1	1	1	1	1	1	1	1	1	1	1	1	1	1	1	1	1	1	1	1	1	1	1	1	1	1	1	1	1	1	1	1	1	1
yeps_YPTB1676	1	6	1	7	1	1	1	6	6	1	7	1	1	1	1	1	1	1	1	1	1	1	1	1	1	1	1	1	1	1	1	1	7	7	1
yeps_YPTB1677	1	1	1	1	1	1	1	6	1	1	1	3	1	1	1	1	1	2	1	1	1	1	1	1	1	1	1	1	1	1	1	1	1	1	1
yeps_YPTB1678	1	1	1	1	1	1	1	1	1	1	1	1	1	1	1	1	1	4	1	1	1	1	1	1	1	1	1	1	1	1	1	1	1	1	1
yeps_YPTB1679	1	7	1	8	1	1	1	1	7	8	8	3	1	1	1	8	1	4	1	1	1	1	1	1	1	1	1	1	1	1	1	1	8	8	1
yeps_YPTB1682	1	7	1	10	1	1	1	1	8	10	10	3	1	1	1	10	1	4	1	1	1	1	1	1	1	1	1	1	1	1	1	1	10	10	1
yeps_YPTB1683	4	7	4	2	4	4	4	9	7	2	2	3	4	4	4	2	4	5	4	4	4	4	4	4	4	4	4	4	4	4	4	4	2	2	4
yeps_YPTB1684	59	9	1	13	89	1	1	10	9	12	13	3	1	1	1	12	1	5	1	1	1	1	1	1	1	1	1	1	1	1	1	1	13	13	1
yeps_YPTB1686	1	5	1	2	1	1	1	2	5	2	2	3	1	1	1	2	1	2	1	1	1	1	1	1	1	1	1	1	1	1	1	1	2	2	1
yeps_YPTB1687	1	8	1	13	1	1	1	2	9	12	13	3	1	1	1	12	1	5	1	1	1	1	1	1	1	1	1	1	1	1	1	1	13	13	1
yeps_YPTB1688	1	7	1	9	1	1	1	3	1	9	9	3	1	1	1	9	1	4	1	1	1	1	1	1	1	1	1	1	1	1	1	1	9	9	1
yeps_YPTB1691	3	6	3	10	1	1	3	7	6	9	10	1	1	1	1	9	1	2	3	3	1	1	1	3	1	1	1	1	1	1	1	1	10	10	3

yeps_YPTB1693	3	5	3	4	1	1	3	6	5	7	4	1	1	1	1	7	1	4	3	3	1	1	1	3	1	1	1	1	1	1	1	1	4	4	3
yeps_YPTB1696	5	9	5	3	1	1	5	10	9	3	3	3	1	1	1	3	1	6	5	5	1	1	1	5	1	1	1	1	1	1	1	1	3	3	5
yeps_YPTB1697	1	2	1	1	1	1	1	1	2	1	1	2	1	1	1	1	1	1	1	1	1	1	1	1	1	1	1	1	1	1	1	1	1	1	1
yeps_YPTB1699	1	2	1	2	1	1	1	2	2	2	2	3	1	1	1	2	1	2	1	1	1	1	1	1	1	1	1	1	1	1	1	1	2	2	1
yeps_YPTB1701	1	3	1	5	1	1	1	2	3	5	5	3	1	1	1	5	1	4	1	1	1	1	1	1	1	1	1	1	1	1	1	1	5	5	1
yeps_YPTB1703	4	6	4	8	1	1	4	5	6	8	8	3	1	1	1	8	1	2	4	4	1	1	1	4	1	1	1	1	1	1	1	1	8	8	4
yeps_YPTB1704	2	3	2	3	1	1	2	3	3	3	3	1	1	1	1	3	1	2	2	2	1	1	1	2	1	1	1	1	1	1	1	1	3	3	2
yeps_YPTB1705	1	1	1	1	1	1	1	1	1	1	1	1	1	1	1	1	1	1	1	1	1	1	1	1	1	1	1	1	1	1	1	1	1	1	1
yeps_YPTB1708	1	1	1	1	1	1	1	1	1	6	1	2	1	1	1	6	1	1	1	1	1	1	1	1	1	1	1	1	1	1	1	1	1	1	1
yeps_YPTB1709	3	1	3	9	1	1	3	7	6	8	9	1	1	1	1	8	1	3	3	3	1	1	1	3	1	1	1	1	1	1	1	1	9	9	3
yeps_YPTB1710	1	1	1	1	1	1	1	1	1	1	1	1	1	1	1	1	1	1	1	1	1	1	1	1	1	1	1	1	1	1	1	1	1	1	1
yeps_YPTB1711	1	1	1	2	1	1	1	2	1	2	2	1	1	1	1	2	1	1	1	1	1	1	1	1	1	25	1	1	1	1	1	1	2	2	1
yeps_YPTB1712	1	1	1	1	1	1	1	1	1	1	1	1	1	1	1	1	1	1	1	1	1	1	1	1	1	1	1	1	1	1	1	1	1	1	1
yeps_YPTB1713	1	5	1	6	1	1	1	1	5	6	6	1	1	1	1	6	1	1	1	1	1	1	1	1	1	1	1	1	1	1	1	1	6	6	1
yeps_YPTB1714	23	5	1	7	1	1	1	5	5	7	7	1	1	1	1	7	1	1	1	1	1	1	1	1	1	1	1	1	1	1	1	1	7	7	1
yeps_YPTB1716	2	4	2	2	1	1	2	5	4	2	2	1	1	1	1	2	1	2	2	2	1	1	1	2	1	1	1	1	1	1	1	1	2	2	2
yeps_YPTB1718	1	2	1	1	1	1	1	3	2	1	1	1	1	1	1	1	1	1	1	1	1	1	1	1	1	1	1	1	1	1	1	1	1	1	1
yeps_YPTB1719	6	7	6	1	1	1	6	8	7	1	1	1	1	1	1	1	1	1	6	6	1	1	1	6	1	1	1	1	1	1	1	1	1	1	6
yeps_YPTB1722	1	2	1	2	1	1	1	2	2	2	2	1	1	1	1	2	1	3	1	1	1	1	1	1	1	1	1	1	1	1	1	1	2	2	1
yeps_YPTB1725	8	6	8	9	1	1	8	6	6	9	9	1	1	1	1	9	1	4	3	3	1	1	1	3	1	1	1	1	1	1	1	1	9	9	8
yeps_YPTB1728	2	4	2	5	1	1	2	3	4	5	5	1	1	1	1	5	1	3	2	2	1	1	1	2	1	1	1	1	1	1	1	1	5	5	2
yeps_YPTB1729	3	7	3	13	1	1	3	10	7	12	13	1	1	1	1	12	1	4	3	3	1	1	1	3	1	1	1	1	1	1	1	1	13	13	3
yeps_YPTB1912	4	8	4	12	1	1	4	9	8	11	12	3	1	1	1	11	1	5	4	4	1	1	1	4	1	1	1	1	1	1	1	1	12	12	4
yeps_YPTB1915	5	5	5	2	1	1	5	1	5	6	2	3	1	1	1	6	1	1	5	5	1	1	1	5	1	1	1	1	1	1	1	1	2	2	5
yeps_YPTB1916	4	2	4	3	1	1	4	1	2	5	3	3	1	1	1	5	1	4	4	4	1	1	1	4	1	1	1	1	1	1	1	1	3	3	4
yeps_YPTB1917	3	2	3	5	1	1	3	1	2	5	5	1	1	1	1	5	1	3	3	3	1	1	1	3	1	1	1	1	1	1	1	1	5	5	3
yeps_YPTB1919	5	10	5	13	3	3	5	12	10	13	13	3	3	7	3	13	7	5	5	5	3	3	3	5	3	3	3	3	3	3	3	3	13	13	5
yeps_YPTB1920	1	1	1	1	1	1	1	3	1	1	1	1	1	1	1	1	1	1	1	1	1	1	1	1	1	1	1	1	1	1	1	1	1	1	1
yeps_YPTB1926	3	4	3	6	1	1	3	4	4	5	6	1	1	1	1	5	1	3	3	3	1	1	1	3	17	17	1	1	1	1	1	17	6	6	3
yeps_YPTB1927	2	4	2	7	1	1	2	2	4	7	7	1	1	1	1	7	1	2	2	2	1	1	1	2	1	1	1	1	1	1	1	1	7	7	2
yeps_YPTB1928	2	3	2	6	1	1	2	4	3	6	6	1	1	1	1	6	1	1	2	2	1	1	1	2	1	1	1	1	1	1	1	1	6	6	2
yeps_YPTB1929	3	5	3	8	1	1	3	6	5	8	8	1	1	1	1	8	1	1	3	3	1	1	1	3	1	1	1	1	1	1	1	1	8	8	3
yeps_YPTB1931	3		3	6	1	1	3	1	5	6	6	1	1	1	1	6	1	1	3	3	1	1	1	3	1	1	1	1	1	1	1	1	6	6	3
yeps_YPTB1933	3	3	3	6	1	1	3	5	3	3	6	1	1	1	1	3	1	7	3	3	1	1	1	3	1	1	1	1	1	1	1	1	6	6	3
yeps_YPTB1944	2	4	2	6	1	1	2	5	4	6	6	1	1	1	1	6	1	1	2	2	1	1	1	2	1	1	1	1	1	1	1	1	6	6	2
yeps_YPTB1946	1	3	1	3	1	1	1	3	3	3	3	3	1	1	1	3	1	3	1	1	1	1	1	1	1	1	1	1	1	1	1	1	3	3	1
yeps_YPTB1950	3	7	3	11	1	1	3	9	8	11	11	1	1	1	1	11	1	4	3	3	1	1	1	3	1	1	1	1	1	1	1	1	11	11	3
yeps_YPTB1951	4	7	4	4	1	1	4	2	7	4	4	1	1	1	1	4	1	4	4	4	1	1	1	4	1	1	1	1	1	1	1	1	4	4	4
yeps_YPTB1952	4	7	4	4	1	1	4	3	7	4	4	3	1	1	1	4	1	3	4	4	1	1	1	4	1	1	1	1	1	1	1	1	4	4	4
yeps_YPTB1954	1	1	1	3	1	1	1	1	1	3	3	1	1	1	1	3	1	3	1	1	1	1	1	1	1	1	1	1	1	1	1	1	3	3	1
yeps_YPTB1957	5	10	5	15	1	1	5	8	11	14	15	3	1	1	1	14	1	8	5	5	1	1	1	5	1	1	1	1	1	1	1	1	15	15	5
yeps_YPTB1965	3	6	3	8	1	1	3	1	6	8	8	3	1	1	1	8	1	1	3	3	1	1	1	3	1	1	1	1	1	1	1	1	8	8	3
yeps_YPTB1968	1	2	1	7	1	1	1	6	2	7	7	1	1	1	1	7	1	2	1	1	1	1	1	1	1	1	1	1	1	1	1	1	7	7	1
yeps_YPTB1969	1	2	1	2	1	1	1	2	2	2	2	1	1	1	1	2	1	2	1	1	1	1	1	1	1	1	1	1	1	1	1	1	2	2	1
yeps_YPTB1972	5	9	5	11	1	1	5	6	9	11	11	3	1	1	1	11	1	12	5	5	1	1	1	5	1	1	1	1	1	1	1	1	11	11	5
yeps_YPTB1974	4	9	4	11	1	1	4	10	9	11	11	3	1	1	1	11	1	5	4	4	1	1	1	4	1	1	1	1	1	1	1	1	11	11	4
yeps_YPTB1975	3	7	3	10	1	1	3	8	7	10	10	1	1	1	1	10	1	4	3	3	1	1	1	3	1	1	1	1	1	1	1	1	10	10	3
yeps_YPTB1977	2	2	2	2	1	1	2	2	2	2	2	1	1	1	1	2	1	1	2	2	1	1	1	2	1	1	1	1	1	1	1	1	2	2	2
yeps_YPTB1978	3	7	3	6	1	1	3	8	7	6	6	1	1	1	1	6	1	4	3	3	1	1	1	3	1	1	1	1	1	1	1	1	6	6	3
yeps_YPTB1983	2	1	2	1	1	1	2	1	1	1	1	1	1	1	1	1	1	2	2	2				2	1	1						1	1	1	2
yeps_YPTB1984	1	1	1	1	1	1	1	1	1	1	1	1	1	1	1	1	1	1	1	1				1	1	1						1	1	1	1
yeps_YPTB1985	3	3	3	1	1	1	3	3	3	1	1	1	1	1	1	1	1	3	3	3				3	1	1						1	1	1	3
yeps_YPTB1986	3	3	3	3	1	1	3	5	3	3	3	1	1	1	1	3	1	3	3	3				3	1	1						1	3	3	3
yeps_YPTB1987	2	3	2	4	1	1	2	1	3	4	4	1	1	1	1	4	1	2	2	2				2	1	1						1	4	4	2
yeps_YPTB1990	2	4	2	2	1	1	2	5	4	2	2	1	1	1	1	2	1	2	2	2	1	1	1	2	1	1	1	1	1	1	1	1	2	2	2
yeps_YPTB1991		2	2	4	1	1	2	3	2	4	4	1	1	1	1	4	1	2	2	2	1	1	1	2	1	1	1	1	1	1	1	1	4	4	2
yeps_YPTB1992	3	1	3	10	1	1	3	7	1	10	10	1	1	5	1	10	5	3	3	3	1	1	1	3	1	1	1	1	1	1	1	1	10	10	3
yeps_YPTB1993	3	6	3	8	1	1	3	7	6	8	8	1	1	1	1	8	1	3	3	3	1	1	1	3	1	1	1	1	1	1	1	1	8	8	3
yeps_YPTB1998	3	3	3	7	3	3	3	6	3	7	7	3	3	3	3	7	3	3	3	3	3	3	3	3	3	3	3	3	3	3	3	3	7	7	3
yeps_YPTB2000	3	2	3	2	1	1	3	2	2	2	2	1	1	1	1	2	1	2	3	3	1	1	1	3	1	1	1	1	1	1	1	1	2	2	3
yeps_YPTB2003	4	7	4	1	1	1	4	3	7	1	1	3	1	1	1	1	1	5	4	4	1	1	1	4	1	1	1	1	1	1	1	1	1	1	4
yeps_YPTB2004	3	6	3	5	1	1	3	3	6	5	5	3	1	1	1	5	1	5	3	3	1	1	1	3	1	1	1	1	1	1	1	1	5	5	3
yeps_YPTB2006	1	5	1	1	1	1	1	1	5	1	1	1	1	1	1	1	1	1	1	1	1	1	1	1	1	1	1	1	1	1	1	1	1	1	1
yeps_YPTB2014	1	6	1	8	1	1	1	9	8	11	8	3	3	3	1	11	3	12	1	1	1	1	1	1	3	3	1	1	1	1	1	3	8	8	1
yeps_YPTB2015	1	6	1	6	1	1	1	7	6	6	6	3	3	3	1	6	3	4	1	1	1	1	1	1	3	3	1	1	1	1	1	3	6	6	1
yeps_YPTB2020	1	3	1	4	1	1	1	3	3	4	4	3	3	3	1	4	3	3	1	1	1	1	1	1	3	3	1	1	1	1	1	3	4	4	1
yeps_YPTB2021	5	1	1	4	1	1	1	1	1	4	4	1	1	1	1	4	1	1	1	1	1	1	1	1	1	1	1	1	1	1	1	1	4	4	1
yeps_YPTB2029	4	8	4	11	1	3	4	10	9	11	11	3	3	3	3	11	3	6	4	4	3	3	3	4	3	3	3	3	3	3	3	3	11	11	4
yeps_YPTB2033	3	4	3	2	1	1	3	3	4	2	2	1	1	1	1	2	1	2	3	3	1	1	1	3	1	1	1	1	1	1	1	1	2	2	3
yeps_YPTB2034	2	2	2	2	1	1	2	2	2	2	2	1	1	1	1	2	1	2	2	2	1	1	1	2	1	1	1	1	1	1	1	1	2	2	2
yeps_YPTB2037	1	1	1	2	1	1	1	1	1	2	2	1	1	1	1	2	1	2	1	1	1	1	1	1	1	1	1	1	1	1	1	1	2	2	1
yeps_YPTB2039	1	1	1	1	1	1	1	1	1	1	1	1	1	1	1	1	1	1	1	1	1	1	1	1	1	1	1	1	1	1	1	1	1	1	1
yeps_YPTB2041	3	6	3	9	1	1	3	7	6	8	9	1	1	1	1	8	1	4	3	3	1	1	1	3	1	1	1	1	1	1	1	1	9	9	3
yeps_YPTB2043	3	4	3	4	1	1	3	4	4	4	4	1	1	1	1	4	1	4	3	3	1	1	1	3	1	1	1	1	1	1	1	31	4	4	3
yeps_YPTB2044	1	4	1	2	1	1	1	5	4	2	2	1	1	1	1	2	1	2	1	1	1	1	1	1	1	1	1	1	1	1	1	1	2	2	1
yeps_YPTB2048	1	4	1	1	1	1	1	1	4	1	1	1	1	1	1	1	1	3	1	1	1	1	1	1	1	1	1	1	1	1	1	1	1	1	1
yeps_YPTB2049	1	3	1	2	1	1	1	1	3	2	2	1	1	1	1	2	1	1	1	1	1	1	1	1	1	1	1	1	1	1	1	1	2	2	1
yeps_YPTB2054	9	7	3	7	1	1	3	3	38	7	7	1	1	1	1	7	1	3	3	3	1	1	1	3	1	1	1	1	1	1	1	1	7	7	3
yeps_YPTB2055	3	4	3	4	1	1	3	3	4	4	4	15	1	1	1	4	1	3	3	3	1	1	1	3	1	1	1	1	1	1	1	1	4	4	3
yeps_YPTB2062	1	1	1	1	1	1	1	1	1	1	1	1	1	1	1	1	1	1	1	1	1	1	1	1	1	1	1	1	1	1	1	1	1	1	1
yeps_YPTB2063	7	4	6	4	1	1	6	8	4	4	4	1	1	1	1	4	1	4	6	6	1	1	1	6	1	1	1	1	1	1	1	1	4	4	7
yeps_YPTB2065	1	1	1	1	1	1	1	1	1	1	1	1	1	1	1	1	1	1	1	1	1	1	1	1	1	1	1	1	1	1	1	1	1	1	1
yeps_YPTB2076	4	7	4	10	1	1	4	8	7	10	10	3	1	1	1	10	1	2	4	4	1	1	1	4	1	1	1	1	1	1	1	1	10	10	4
yeps_YPTB2085	2	3	2	1	1	1	2	2	3	1	1	1	1	1	1	1	1	2	2	2	1	1	1	2	1	1	1	1	1	1	1	1	1	1	2
yeps_YPTB2087	3	7	3	7	1	1	3	8	7	7	7	1	1	1	1	7	1	4	3	3	1	1	1	3	1	1	1	1	1	1	1	1	7	7	3
yeps_YPTB2088	2	2	2	2	1	1	2	5	2	2	2	1	1	1	1	2	1	2	2	2	1	1	1	2	1	1	1	1	1	1	1	1	2	2	2
yeps_YPTB2091	4	7	4	12	8	8	4	10	7	12	12	3	4	4	8	12	4	2	4	4	8	8	8	4	4	4	8	8	8	8	8	4	12	12	4
yeps_YPTB2119	2	1	2	1	1	1	2	2	1	1	1	1	1	1	1	1	1	1	2	2	1	1	1	2	1	1	1	1	1	1	1	1	1	1	2
yeps_YPTB2124	1	1	1	1	1	1	1	1	1	1	1	1	1	1	1	1	1	1	1	1	1	1	1	1	1	1	1	1	1	1	1	1	1	1	1
yeps_YPTB2127	1	7	1	9	1	1	1	1	7	6	9	3	1	1	1	6	1	1	1	1	1	1	1	1	24	24	1	1	1	1	1	24	9	9	1
yeps_YPTB2130	1	3	1	8	1	1	1	7	3	8	8	1	1	1	1	8	1	3	1	1	1	1	1	1	1	1	1	1	1	1	1	1	8	8	1
yeps_YPTB2136	1	1	1	1	1	1	1	1	1	4	1	1	1	1	1	4	1	2	1	1	1	1	1	1	1	1	1	1	1	1	1	1	1	1	1
yeps_YPTB2138	1	1	1	1	1	1	1	1	1	1	1	1	1	1	1	1	1	38	1	1	1	1	1	1	1	1	1	1	1	1	1	1	1	1	1
yeps_YPTB2153	1	3	1	1	1	1	1	1	3	2	1	1	1	1	1	2	1	1	1	1	1	1	1	1	1	1	1	1	1	1	1	1	1	1	1
yeps_YPTB2154	1	1	1	4	1	1	1	1	1	3	4	1	1	1	1	3	1	1	1	1	1	1	1	1	1	1	1	1	1	1	1	1	4	4	1
yeps_YPTB2155	4	5	3	5	1	1	3	5	5	5	5	1	1	1	1	5	1	4	3	3	1	1	1	3	1	1	1	1	1	1	1	1	5	5	3
yeps_YPTB2157	4	7	4	10	1	1	4	9	7	10	10	1	1	1	1	10	1	5	4	4	1	1	1	4	1	1	1	1	1	1	1	1	10	10	4
yeps_YPTB2158	1	5	1	5	1	1	1	7	5	5	5	3	1	1	1	5	1	4	1	1	1	1	1	1	1	1	1	1	1	1	1	1	5	5	1
yeps_YPTB2159	4	7	4	10	1	1	4	8	7	9	10	3	1	1	1	9	1	5	4	4	1	1	1	4	1	1	1	1	1	1	1	1	10	10	4
yeps_YPTB2161	8	7	8	12	1	1	8	10	7	11	12	1	1	1	1	11	1	4	8	8	1	1	1	8	1	1	1	1	1	1	1	1	12	12	8
yeps_YPTB2162	4	4	4	3	1	1	4	5	4	3	3	3	1	1	1	3	1	1	4	4	1	1	1	4	1	1	1	1	1	1	1	1	3	3	4
yeps_YPTB2168	1	1	1	1	1	1	1	4	1	1	1	1	1	1	1	1	1	1	1	1	1	1	1	1	1	1	1	1	1	1	1	1	1	1	1
yeps_YPTB2170	5	4	5	6	1	34	5	6	4	7	6	1	1	1	34	7	1	1	5	5	34	34	34	5	1	1	34	34	34	34	34	1	6	6	5
yeps_YPTB2173	3	6	3	1	1	1	3	3	6	1	1	1	1	1	1	1	1	1	3	3	1	1	1	3	1	1	1	1	1	1	1	1	1	1	3
yeps_YPTB2175	1	6	1	7	1	1	1	1	6	7	7	3	1	1	1	7	1	2	1	1	1	1	1	1	1	1	1	1	1	1	1	1	7	7	1
yeps_YPTB2176	3	1	3	8	1	48	3	1	1	7	8	1	1	1	48	7	1	6	3	3	48	48	48	3	1	1	48	48	48	48	48	1	8	8	3
yeps_YPTB2177	3	3	3	1	1	1	3	4	3	1	1	3	1	1	1	1	1	2	3	3	1	1	1	3	1	1	1	1	1	1	1	1	1	1	3
yeps_YPTB2178	1	4	1	7	1	1	1	5	4	6	7	1	1	1	1	6	1	3	58	1	1	1	1	1	1	1	1	1	1	1	1	1	7	7	1
yeps_YPTB2179	1	2	1	7	1	1	1	5	2	7	7	1	1	1	1	7	1	4	1	1	1	1	1	1	1	1	1	1	1	1	1	1	7	7	1
yeps_YPTB2184	1	6	1	9	1	1	1	7	6	9	9	3	1	1	1	9	1	4	1	1	1	1	1	1	1	1	1	1	1	1	1	1	9	9	1
yeps_YPTB2200	3	2	3	6	1	1	3	5	2	6	6	1	1	1	1	6	1	4	3	3	1	1	1	3	1	1	1	1	1	1	1	1	6	6	3
yeps_YPTB2206	1	2	1	1	1	1	1	1	2	1	1	3	3	3	1	1	3	5	1	1	1	1	1	1	3	3	1	1	1	1	1	3	1	1	1
yeps_YPTB2209	1	2	1	5	1	1	1	4	2	5	5	2	1	1	1	5	1	2	1	1	1	1	1	1	1	1	1	1	1	1	1	1	5	5	1
yeps_YPTB2210	1	4	1	4	1	1	1	7	4	4	4	2	3	3	1	4	3	4	1	1	1	1	1	1	3	3	1	1	1	1	1	3	4	4	1
yeps_YPTB2211	1	4	1	9	1	1	1	6	4	8	9	2	3	3	1	8	3	4	1	1	1	1	1	1	3	3	1	1	1	1	1	3	9	9	1
yeps_YPTB2217	1	1	1	2	1	1	1	1	1	2	2	3	3	3	1	2	3	1	1	1	1	1	1	1	3	3	1	1	1	1	1	3	2	2	1
yeps_YPTB2218	1	3	1	6	1	1	1	5	3	6	6	3	3	3	1	6	3	1	1	1	1	1	1	1	3	3	1	1	1	1	1	3	6	6	1
yeps_YPTB2221	1	4	1	1	1	4	1	1	4	1	1	3	4	4	4	1	4	1	1	1	4	4	4	1	4	4	4	4	4	4	4	4	1	1	5
yeps_YPTB2222	1	2	1	1	1		1	1	2	1	1	1	1	1		1	1	1	1	1				1	1	1						1	1	1	1
yeps_YPTB2225	1	8	1	11	1	4	1	9	8	11	11	3	4	4	4	11	4	5	1	1	4	4	4	1	4	4	4	4	4	4	4	4	11	11	1
yeps_YPTB2226	1	3	1	5	1	3	1	3	3	4	5	1	3	3	3	4	3	3	1	1	3	3	3	1	3	3	3	3	3	3	3	3	5	5	1
yeps_YPTB2227	1	1	1	2	1	3	1	3	1	2	2	1	3	3	3	2	3	1	1	1	3	3	3	1	3	3	3	3	3	3	3	3	2	2	1
yeps_YPTB2228	1	8	9	4	1	4	9	4	8	4	4	3	4	4	4	4	4	5	9	9	4	4	4	9	4	4	4	4	4	4	4	4	4	4	1
yeps_YPTB2229	1	1	1	1	1	1	1	1	1	1	1	1	3	1	1	1	1	1	1	1	1	1	1	1	1	1	1	1	1	1	1	1	1	1	1
yeps_YPTB2230	1	2	1	6	1	3	1	2	2	5	6	2	3	3	3	5	3	2	1	1	3	3	3	1	3	3	3	3	3	3	3	3	6	6	1
yeps_YPTB2231	1	4	1	4	1	4	1	9	8	10	4	3	6	6	4	10	6	5	1	1	4	4	4	1	6	6	4	4	4	4	4	6	4	4	1
yeps_YPTB2232	1	7	1	9	1	6	1	7	7	9	9	3	6	6	6	9	6	5	1	1	6	6	6	1	6	6	6	6	6	6	6	6	9	9	1
yeps_YPTB2239	3	1	3	1	1	1	3	5	1	1	1	3	1	1	1	1	1	6	3	3	1	1	1	3	1	1	1	1	1	1	1	1	1	1	3
yeps_YPTB2241	5	8	5	12	13	1	5	10	9	12	12	3	1	1	1	12	1	5	5	5	1	1	1	5	1	1	1	1	1	1	1	1	12	12	5
yeps_YPTB2242	1	1	1	1	1	1	1	1	1	1	1	1	1	1	1	1	1	1	1	1	1	1	1	1	1	1	1	1	1	1	1	11	1	1	1
yeps_YPTB2246	1	1	1	2	1	1	1	1	1	2	2	1	1	1	1	2	1	1	1	1	1	1	1	1	1	1	1	1	1	1	1	1	2	2	1
yeps_YPTB2248	3	3	3	3	1	1	3	5	3	3	3	1	1	1	1	3	1	3	3	3	1	1	1	3	1	1	1	1	1	1	1	1	3	3	3
yeps_YPTB2251	1	3	1	4	1	1	1	1	3	4	4	1	1	1	1	4	1	3	1	1	1	1	1	1	1	1	1	1	1	1	1	1	4	4	1
yeps_YPTB2252	1	1	1	1	1	1	1	1	1	1	1	1	1	1	1	1	1	1	1	1	1	1	1	1	1	1	1	1	1	1	1	1	1	1	1
yeps_YPTB2254	1	3	1	4	1	1	1	1	3	4	4	1	1	1	1	4	1	2	1	1	1	1	1	1	1	1	1	1	1	1	1	1	4	4	1
yeps_YPTB2258	3	3	3	7	1	1	3	1	3	7	7	1	1	1	1	7	1	3	3	3	1	1	1	3	1	1	1	1	1	1	1	1	7	7	3
yeps_YPTB2262	1	6	1	2	1	1	1	8	6	2	2	3	3	3	1	2	3	4	1	1	1	1	1	1	3	3	1	1	1	1	1	3	2	2	1
yeps_YPTB2264	1	1	1	1	1	1	1	2	1	1	1	1	1	1	1	1	1	3	1	1	1	1	1	1	1	1	1	1	1	1	1	1	1	1	1
yeps_YPTB2265	1	5	1	7	1	1	1	6	5	7	7	3	4	4	1	7	4	2	1	1	1	1	1	1	4	4	1	1	1	1	1	4	7	7	1
yeps_YPTB2267	1	2	1	1	1	1	1	1	2	1	1	1	1	1	1	1	1	1	1	1	1	1	1	1	1	1	1	1	1	1	1	1	1	1	1
yeps_YPTB2268	1	1	1	1	1	1	1	1	1	1	1	2	1	1	1	1	1	1	1	1	1	1	1	1	1	1	1	1	1	1	1	1	1	1	1
yeps_YPTB2269	1	1	1	1	1	1	1	1	1	1	1	1	1	1	1	1	1	1	1	1	1	1	1	1	1	1	1	1	1	1	1	1	1	1	1
yeps_YPTB2270	1	7	1	8	1	1	1	1	7	8	8	3	4	4	1	8	4	1	1	1	1	1	1	1	4	4	1	1	1	1	1	4	8	8	1
yeps_YPTB2276	4	6	4	2	1	4	4	7	6	2	2	3	1	1	4	2	1	5	4	4	4	4	4	4	1	1	4	4	4	4	4	1	2	2	4
yeps_YPTB2279	3	5	3	1	1	3	3	2	5	1	1	1	1	1	3	1	1	2	3	3	3	3	3	3	1	1	3	3	3	3	3	1	1	1	3
yeps_YPTB2280	1	1	1	1	1	1	1	1	1	1	1	1	1	1	1	1	1	1	2	2	1	1	1	2	1	1	1	1	1	1	1	1	1	1	1
yeps_YPTB2281	1	1	1	7	1	1	1	1	1	7	7	1	1	1	1	7	1	5	1	1	1	1	1	1	1	1	1	1	1	1	1	1	7	7	1
yeps_YPTB2282	1	1	1	1	1	1	1	4	1	1	1	1	1	1	1	1	1	1	1	1	1	1	1	1	1	1	1	1	1	1	1	1	1	1	1
yeps_YPTB2284	1	1	1	4	1	1	1	1	1	3	4	1	1	1	1	3	1	1	1	1	1	1	1	1	1	1	1	1	1	1	1	1	4	4	1
yeps_YPTB2285	6	3	3	3	1	3	3	4	3	5	3	1	1	1	3	5	1	3	3	3	3	3	3	3	1	1	3	3	3	3	3	1	3	3	3
yeps_YPTB2292	3	3	3	8	1	1	3	6	3	8	8	3	1	1	1	8	1	4	3	3	1	1	1	3	1	1	1	1	1	1	1	1	8	8	3
yeps_YPTB2293	3	3	3	3	1	1	3	6	3	3	3	3	1	1	1	3	1	4	3	3	1	1	1	3	1	1	1	1	1	1	1	1	3	3	3
yeps_YPTB2295	1	1	1	1	1	1	1	1	1	1	1	1	1	1	1	1	1	2	1	1	1	1	1	1	1	1	1	1	1	1	1	1	1	1	1
yeps_YPTB2298	3	1	3	6	1	1	3	3	1	6	6	3	1	1	1	6	1	1	3	3	1	1	1	3	1	1	1	1	1	1	1	1	6	6	3
yeps_YPTB2299	3	7	3	5	1	1	3	1	7	5	5	3	1	1	1	5	1	4	3	3	1	1	1	3	1	1	1	1	1	1	1	1	5	5	3
yeps_YPTB2300	4	8	4	10	1	1	4	9	8	10	10	3	1	1	1	10	1	5	4	4	1	1	1	4	1	1	1	1	1	1	1	1	10	10	4
yeps_YPTB2303	1	2	1	8	1	1	1	4	2	8	8	3	1	1	1	8	1	5	1	1	1	1	1	1	1	1	1	1	1	1	1	1	8	8	1
yeps_YPTB2304	4	7	4	4	1	1	4	1	7	4	4	1	1	1	1	4	1	1	4	4	1	1	1	4	1	1	1	1	1	1	1	1	4	4	4
yeps_YPTB2309	3	6	3	1	1	1	3	7	6	1	1	1	1	1	1	1	1	1	3	3	1	1	1	3	1	1	1	1	1	1	1	1	1	1	3
yeps_YPTB2310	8	7	8	6	1	1	8	9	7	6	6	1	1	1	1	6	1	4	8	8	1	1	1	8	1	1	1	1	1	1	1	1	6	6	8
yeps_YPTB2313	4	4	4	4	1	1	4	6	4	4	4	1	1	1	1	4	1	4	4	4	1	1	1	4	1	1	1	1	1	1	1	1	4	4	4
yeps_YPTB2314	1	1	1	2	1	1	1	1	1	2	2	1	1	1	1	2	1	1	1	1	1	1	1	1	1	1	1	1	1	1	1	1	2	2	1
yeps_YPTB2382	3	3	3	4		2	3	3	3	4	4	2	2	2	2	4	2	1	3	3	2	2	2	3	2	2	2	2	2	2	2	2	4	4	3
yeps_YPTB2387	1	1	1	1	1	1	1	1	1	1	1	1	1	1	1	1	1	1	1	1	1	1	1	1	1	1	1	1	1	1	1	1	1	1	1
yeps_YPTB2388	2	4	2	2	1	2	2	1	4	2	2	2	2	2	2	2	2	1	2	2	2	2	2	2	2	2	2	2	2	2	2	2	2	2	2
yeps_YPTB2393	1	177	1	4	6	3	1	7	5	4	4	1	3	3	3	182	41	4	1	1	30	3	3	1	3	3	41	3	3	3	3	3	4	4	1

yeps_YPTB2395	1	6	1	8	1	1	1	4	7	8	8	3	1	1	1	8	1	4	1	1	1	1	1	1	1	1	1	1	1	1	1	1	8	8	1
yeps_YPTB2398	4	4	4	4	1	4	4	3	4	4	4	3	4	4	4	4	4	5	4	4	4	4	4	4	4	4	4	4	4	4	4	4	4	4	4
yeps_YPTB2399	1	9	1	11	1	4	1	10	9	11	11	3	4	4	4	11	4	7	1	1	4	4	4	1	4	4	4	4	4	4	4	4	11	11	1
yeps_YPTB2401	1	7	1	8	1	3	1	4	7	8	8	1	3	3	3	8	3	4	1	1	3	3	3	1	3	3	3	3	3	3	3	3	8	8	1
yeps_YPTB2413	1	1	1	2	1	1	1	1	1	2	2	1	1	1	1	2	1	1	1	1	1	1	1	1	1	1	1	1	1	1	1	1	2	2	1
yeps_YPTB2418	1	1	1	5	1	1	1	1	1	5	5	2	1	1	1	5	1	3	1	1	1	1	1	1	1	1	1	1	1	1	1	1	5	5	1
yeps_YPTB2419	4	1	4	6	1	4	4	3	26	6	6	3	1	1	4	6	1	5	4	4	4	4	4	4	1	1	4	4	4	4	4	1	6	6	4
yeps_YPTB2420	1	1	1	1	1	1	1	1	1	1	1	1	1	1	1	1	1	1	1	1	1	1	1	1	1	1	1	1	1	1	1	1	1	1	1
yeps_YPTB2421	8	6	8	9	1	8	8	2	6	1	9	3	1	1	8	1	1	6	8	8	8	8	8	8	1	1	8	8	8	8	8	1	9	9	8
yeps_YPTB2425	1	1	1	1	1	1	1	1	1	1	1	1	1	1	1	1	1	2	1	1	1	1	1	1	1	1	1	1	1	1	1	1	1	1	1
yeps_YPTB2430	3	6	3	7	1	1	3	6	6	7	7	1	1	1	1	7	1	4	3	3	1	1	1	3	1	1	1	1	1	1	1	1	7	7	3
yeps_YPTB2435	3	7	3	8	1	1	3	3	7	8	8	3	1	1	1	8	1	4	3	3	1	1	1	3	1	1	1	1	1	1	1	1	8	8	3
yeps_YPTB2438	3	2	3	5	1	1	3	3	2	5	5	3	6	31	1	5	1	3	3	3	1	1	1	3	1	1	1	1	1	1	1	1	5	5	3
yeps_YPTB2439	3	1	3	6	1	1	3	6	1	6	6	3	1	1	1	6	1	5	3	3	1	1	1	3	1	1	1	1	1	1	1	1	6	6	3
yeps_YPTB2440	4	9	4	5	1	4	4	5	9	5	5	3	1	7	4	5	7	5	4	4	4	4	4	4	1	1	4	4	4	4	4	1	5	5	4
yeps_YPTB2441	3	1	3	1	1	3	3	1	1	1	1	2	1	1	3	1	1	2	3	3	3	3	3	3	1	1	3	3	3	3	3	1	1	1	3
yeps_YPTB2449	1	3	1	5	1	1	1	2	3	5	5	3	1	1	1	5	1	2	1	1	1	1	1	1	1	1	1	1	1	1	1	1	5	5	1
yeps_YPTB2451	2	1	2	1	7	1	2	2	1	6	1	1	1	1	1	6	1	2	2	2	1	1	1	2	1	1	1	1	1	1	1	1	1	1	2
yeps_YPTB2452	1	1	1	4	1	1	1	2	1	4	4	1	1	1	1	4	1	2	1	1	1	1	1	1	1	1	1	1	1	1	1	1	4	4	1
yeps_YPTB2453	1	1	1	1	1	1	1	1	1	1	1	1	1	1	1	1	1	1	1	1	1	1	1	1	1	1	1	1	1	1	1	1	1	1	1
yeps_YPTB2463	4	8	4	9	1	1	4	3	8	9	9	3	1	1	1	9	1	3	4	4	1	1	1	4	1	1	1	1	1	1	1	1	9	9	4
yeps_YPTB2464	3	1	3	3	1	1	3	3	1	5	3	3	1	1	1	5	1	3		3	1	1	1	3	1	1	1	1	1	1	1	1	3	3	3
yeps_YPTB2465	4	4	4	7	1	1	4	4	4	7	7	3	1	1	1	7	1	5	4	4	1	1	1	4	1	1	1	1	1	1	1	1	7	7	4
yeps_YPTB2466	3	6	3	4	1	1	3	4	6	4	4	3	1	1	1	4	1	4	3	3	1	1	1	3	1	1	1	1	1	1	1	1	4	4	3
yeps_YPTB2469	4	5	4	3	1	1	4	3	25	3	3	3	1	1	1	3	1	3	4	4	1	1	1	4	1	1	1	1	1	1	1	1	3	3	4
yeps_YPTB2472	4	8	4	1	1	1	4	9	8	1	1	3	1	1	1	1	1	5	4	4	1	1	1	4	1	1	1	1	1	1	1	1	1	1	4
yeps_YPTB2473	2	1	2	2	1	1	2	1	1	2	2	1	1	1	1	2	1	3	2	2	1	1	1	2	1	1	1	1	1	1	1	1	2	2	2
yeps_YPTB2476	1	1	1	1	1	1	1	3	1	1	1	1	1	1	1	1	1	1	1	1	1	1	1	1	1	1	1	1	1	1	1	1	1	1	1
yeps_YPTB2480	1	1	1	1	1	1	1	1	1	1	1	1	1	1	1	1	1	1	1	1	1	1	1	1	1	1	1	1	1	1	1	1	1	1	1
yeps_YPTB2481	1	1	1	2	1	1	1	1	1	2	2	1	1	1	1	2	1	2	1	1	1	1	1	1	1	1	1	1	1	1	1	1	2	2	1
yeps_YPTB2482	3	6	3	8	1	1	3	7	6	8	8	1	1	1	1	8	1	4	3	3	1	1	1	3	1	1	1	1	1	1	1	1	8	8	3
yeps_YPTB2483	1	1	1	1	1	1	1	1	1	1	1	1	1	1	1	1	1	1	1	1	1	1	1	1	1	1	1	1	1	1	1	1	1	1	1
yeps_YPTB2484	1	1	1	1	1	1	1	1	1	1	1	1	1	1	1	1	1	1	1	1	1	1	1	1	1	1	1	1	1	1	1	1	1	1	1
yeps_YPTB2488	4	6	4	3	1	1	4	3	6	3	3	3	1	1	1	3	1	3	4	4	1	1	1	4	1	1	1	1	1	1	1	1	3	3	4
yeps_YPTB2489	1	3	1	1	1	1	1	1	3	1	1	1	1	1	1	1	1	4	1	1	1	1	1	1	1	1	1	1	1	1	1	1	1	1	1
yeps_YPTB2503	5	4	5	1	1	1	5	5	4	1	1	1	1	1	1	1	1	1	5	5	1	1	1	5	1	1	1	1	1	1	1	1	1	1	5
yeps_YPTB2504	4	5	4	8	1	1	4	7	5	8	8	3	1	1	1	8	1	3	4	4	1	1	1	4	1	1	1	1	1	1	1	1	8	8	4
yeps_YPTB2505	3	4	3	6	1	1	3	5	4	6	6	1	1	1	1	6	1	3	3	3	1	1	1	3	1	1	1	1	1	1	1	1	6	6	3
yeps_YPTB2506	4	6	4	8	1	1	4	7	6	8	8	1	1	1	1	8	1	5	4	4	1	1	1	4	1	1	1	1	1	1	1	1	8	8	4
yeps_YPTB2507	3	6	3	3	1	1	3	7	6	3	3	1	1	1	1	3	1	4	3	3	1	1	1	3	1	1	1	1	1	1	1	1	3	3	3
yeps_YPTB2508	4	7	4	11	1	1	4	8	7	10	11	1	1	1	1	10	1	5	4	4	1	1	1	4	1	1	1	1	1	1	1	1	11	11	4
yeps_YPTB2513	3	6	3	9	1	1	3	7	6	8	9	1	1	1	1	8	1	4	3	3	1	1	1	3	1	1	1	1	1	1	1	1	9	9	3
yeps_YPTB2514	1	5	1	10	1	1	1	6	5	8	10	1	1	1	1	8	1	3	1	1	1	1	1	1	1	1	1	1	1	1	1	1	10	10	1
yeps_YPTB2516	1	3	1	3	1	1	1	1	3	3	3	1	1	1	1	3	1	3	1	1	1	1	1	1	1	1	1	1	1	1	1	1	3	3	1
yeps_YPTB2517	3	3	3	4	1	1	3	5	3	4	4	1	1	1	1	4	1	4	3	3	1	1	1	3	1	1	1	1	1	1	1	1	4	4	3
yeps_YPTB2518	3	4	3	4	1	1	3	4	4	4	4	1	1	1	1	4	1	4	3	3	1	1	1	3	1	1	1	1	1	1	1	1	4	4	3
yeps_YPTB2519	3	1	3	2	1	1	3	6	1	2	2	1	1	1	1	2	1	2	3	3	1	1	1	3	1	1	1	1	1	1	1	1	2	2	3
yeps_YPTB2520	1	5	1	6	1	1	1	1	5	6	6	1	1	1	1	6	1	3	1	1	1	1	1	1	1	1	1	1	1	1	1	1	6	6	1
yeps_YPTB2535	1	6	1	8	1	1	1	7	6	8	8	1	1	34	1	8	1	2	1	1	1	1	1	1	1	1	1	1	1	1	1	1	8	8	1
yeps_YPTB2536	1	7	1	10	1	1	1	8	7	10	10	1	1	1	1	10	1	3	1	1	1	1	1	1	1	1	1	1	1	1	1	1	10	10	1
yeps_YPTB2537	50	8	3	5	1	3	3	9	8	5	5	1	1	1	3	5	1	4	3	3	3	3	3	3	1	1	3	3	3	3	3	1	5	5	3
yeps_YPTB2541	3	6	3	8	1	3	3	6	6	8	8	3	3	3	3	8	3	1	3	3	3	3	3	3	3	3	3	3	3	3	3	3	8	8	3
yeps_YPTB2542	1	1	1	3	1	1	1	5	1	3	3	1	1	1	1	3	1	3	1	1	1	1	1	1	1	1	1	1	1	1	1	1	3	3	1
yeps_YPTB2543	1	6	1	8	1	1	1	6	6	8	8	3	3	3	1	8	3	4	1	1	1	1	1	1	3	3	1	1	1	1	1	3	8	8	1
yeps_YPTB2544	1	1	1	8	1	1	1	1	1	8	8	3	3	3	1	8	3	4	1	37	1	1	1	1	3	3	1	1	1	1	1	3	8	8	1
yeps_YPTB2546	1	1	1	1	1	1	1	1	1	1	1	1	1	1	1	1	1	2	1	1	1	1	1	1	1	1	1	1	1	1	1	1	1	1	1
yeps_YPTB2547	1	6	1	8	1	1	1	1	6	8	8	1	1	1	1	8	1	9	1	1	1	1	1	1	1	1	1	1	1	1	1	1	8	8	1
yeps_YPTB2548	1	1	1	7	1	1	1	1	1	7	7	1	1	1	1	7	1	3	1	1	1	1	1	1	1	1	1	1	1	1	1	1	7	7	1
yeps_YPTB2550	1	6	1	8	1	1	1	7	6	8	8	1	1	1	1	8	1	3	1	1	1	1	1	1	1	1	1	1	1	1	1	1	8	8	1
yeps_YPTB2556	4	9	4	12	1	4	4	10	9	12	12	3	3	3	4	12	3	5	4	4	4	4	4	4	3	3	4	4	4	4	4	3	12	12	4
yeps_YPTB2559	5	9	5	5	1	5	5	10	9	5	5	3	12	7	5	5	7	5	5	5	5	5	5	5	4	4	5	5	5	5	5	4	5	5	5
yeps_YPTB2560	5	8	5	12	1	5	5	10	9	12	12	3	4	4	5	12	4	1	5	5	5	5	5	5	4	4	5	5	5	5	5	89	12	12	5
yeps_YPTB2561	5	3	5	3	1	5	5	8	3	3	3	3	4	4	5	3	4	1	5	5	5	5	5	5	4	4	5	5	5	5	5	4	3	3	5
yeps_YPTB2572	1	4	1	7	1	1	1	5	4	7	7	1	1	1	1	7	1	1	1	1	1	1	1	1	1	1	1	1	1	1	1	1	7	7	1
yeps_YPTB2575	4	7	4	10	1	1	4	8	7	10	10	1	1	1	1	10	1	4	30	4	1	1	1	4	1	1	1	1	1	1	1	1	10	10	4
yeps_YPTB2576	4	7	4	11	3	3	4	9	7	11	11	30	3	3	3	11	3	4	4	4	3	3	3	4	3	3	3	3	3	3	3	3	11	11	4
yeps_YPTB2579	3	2	3	5	1	1	3	2	2	5	5	1	1	1	1	5	1	3	3	3	1	1	1	3	1	1	1	1	1	1	1	1	5	5	3
yeps_YPTB2582	4	7	4	10	1	79	4	18	7	121	10	1	1	1	79	10	1	12	4	11	79	79	79	11	1	1	79	79	79	79	79	1	10	10	4
yeps_YPTB2583	13	7	3	12	1	52	3	8	7	10	12	1	5	5	52	10	5	3	3	3	52	52	52	3	5	5	52	52	52	52	52	5	12	12	3
yeps_YPTB2584	1	4	1	6	1	1	1	5	4	6	6	1	1	1	1	6	1	1	1	1	1	1	1	1	1	1	1	1	1	1	1	1	6	6	1
yeps_YPTB2593	1	1	1	6	1	1	1	5	1	6	6	3	1	1	1	6	1	1	1	1	1	1	1	1	1	1	1	1	1	1	1	1	6	6	1
yeps_YPTB2596	1	2	1	1	1	1	1	1	2	1	1	1	1	1	1	1	1	1	1	1	1	1	1	1	1	1	1	1	1	1	1	1	1	1	1
yeps_YPTB2603	3	3	3	6	1	1	3	5	3	6	6	1	3	3	1	6	3	2	3	3	1	1	1	3	3	3	1	1	1	1	1	3	6	6	3
yeps_YPTB2605	1	1	1	1	1	1	1	2	1	1	1	1	1	1	1	1	1	1	1	1	1	1	1	1	1	1	1	1	1	1	1	1	1	1	1
yeps_YPTB2606	4	4	4	7	1	1	4	4	4	7	7	3	4	4	1	7	4	4	4	4	1	1	1	4	4	4	1	1	1	1	1	4	7	7	4
yeps_YPTB2611	1	2	1	1	1	1	1	1	2	1	1	1	1	1	1	1	1	1	1	1	1	1	1	1	1	1	1	1	1	1	1	1	1	1	1
yeps_YPTB2616	2	1	2	2	1	1	2	2	1	2	2	1	1	1	1	2	1	1	2	2	1	1	1	2	1	1	1	1	1	1	1	1	2	2	2
yeps_YPTB2621	1	1	1	1	1	1	1	1	1	1	1	1	1	1	1	1	1	1	1	1	1	1	1	1	1	1	1	1	1	1	1	1	1	1	1
yeps_YPTB2628	1	1	1	1	1	1	1	1	1	1	1	1	1	1	1	1	1	1	1	1	1	1	1	1	1	1	1	1	1	1	1	1	1	1	1
yeps_YPTB2631	1	7	1	8	1	1	1	8	7	8	8	1	5	5	1	8	5	6	1	1	1	1	1	1	5	5	1	1	1	1	1	5	8	8	1
yeps_YPTB2633	2	4	2	6	1	1	2	2	4	6	6	3	1	1	1	6	1	2	2	2	1	1	1	2	1	1	1	1	1	1	1	1	6	6	2
yeps_YPTB2634	1	4	1	1	1	1	1	1	4	1	1	1	1	1	1	1	1	1	1	1	1	1	1	1	1	1	1	1	1	1	1	1	1	1	1
yeps_YPTB2635	1	1	1	3	1	1	1	1	1	3	3	1	1	1	1	3	1	1	1	1	1	1	1	1	1	1	1	1	1	1	1	1	3	3	1
yeps_YPTB2638	1	1	1	1	1	1	1	1	1	1	1	1	1	1	1	1	1	1	1	1	1	1	1	1	1	1	1	1	1	1	1	1	1	1	1
yeps_YPTB2641	9	8	4	5	1	1	4	3	5	5	5	3	1	1	1	5	1	5	4	4	1	1	1	4	1	1	1	1	1	1	1	1	5	5	4
yeps_YPTB2642	1	1	1	1	1	1	1	1	1	1	1	3	1	1	1	1	1	1	1	1	1	1	1	1	1	1	1	1	1	1	1	1	1	1	1
yeps_YPTB2643	1	1	1	1	1	1	1	3	1	1	1	1	1	1	1	1	1	1	1	1	1	1	1	1	1	1	1	1	1	1	1	1	1	1	1
yeps_YPTB2644	1	6	1	1	1	1	1	8	6	1	1	1	1	1	1	1	1	4	1	1	1	1	1	1	1	1	1	1	1	1	1	1	1	1	1
yeps_YPTB2645	1	1	1	7	1	1	1	5	1	1	7	2	1	1	1	1	1	1	1	1	1	1	1	1	1	1	1	1	1	1	1	1	7	7	1
yeps_YPTB2647	4	6	4	4	1	1	4	3	6	4	4	3	1	1	1	4	1	5	4	4	1	1	1	4	1	1	1	1	1	1	1	1	4	4	4
yeps_YPTB2648	3	3	3	3	1	1	3	4	3	3	3	3	1	1	1	3	1	3	3	3	1	1	1	3	1	1	1	1	1	1	1	1	3	3	3
yeps_YPTB2649	3	7	3	3	1	1	3	8	7	3	3	3	1	1	1	3	1	3	3	3	1	1	1	3	1	1	1	1	1	1	1	1	3	3	3
yeps_YPTB2650	3	5	3	6	1	1	3	4	5	6	6	1	1	1	1	6	1	4	3	3	1	1	1	3	1	1	1	1	1	1	1	1	6	6	3
yeps_YPTB2651	4	6	4	1	1	1	4	7	6	1	1	3	1	1	1	1	1	1	4	4	1	1	1	4	1	1	1	1	1	1	1	1	1	1	4
yeps_YPTB2654	2	2	2	2	1	1	2	2	2	2	2	1	1	1	1	2	1	2	2	2	1	1	1	2	1	1	1	1	1	1	1	1	2	2	2
yeps_YPTB2655	2	2	2	4	1	1	2	3	2	4	4	1	1	1	1	4	1	2	2	2	1	1	1	2	1	1	1	1	1	1	1	1	4	4	2
yeps_YPTB2661	1	7	1	8	1	1	1	4	7	8	8	3	1	1	1	8	1	4	1	1	1	1	1	1	1	1	1	1	1	1	1	1	8	8	1
yeps_YPTB2662	1	6	1	7	1	1	1	3	6	7	7	3	1	1	1	7	1	4	1	1	1	1	1	1	1	1	1	1	1	1	1	1	7	7	1
yeps_YPTB2673	3	1	3	10	1	1	3	2	1	9	10	3	1	1	1	9	1	7	4	4	1	1	1	4	1	1	1	1	1	1	1	1	10	10	3
yeps_YPTB2674	2	5	2	3	1	1	2	1	5	7	3	2	1	1	1	7	1	3	2	2	1	1	1	2	1	1	1	1	1	1	1	1	3	3	2
yeps_YPTB2675	1	1	1	5	1	1	1	1	1	5	5	1	1	1	1	5	1	2	1	1	1	1	1	1	1	1	1	1	1	1	1	1	5	5	1
yeps_YPTB2676	4	8	9	12	1	1	9	1	8	11	12	3	1	1	1	11	1	5	4	4	1	1	1	4	1	1	1	1	1	1	1	1	12	12	4
yeps_YPTB2678	4	3	4	10	1	1	4	9	3	10	10	3	1	1	1	10	1	5	4	4	1	1	1	4	1	1	1	1	1	1	1	1	10	10	11
yeps_YPTB2679	1	1	1	2	1	1	1	1	1	1	2	2	1	1	1	1	1	2	1	1	1	1	1	1	1	1	1	1	1	1	1	1	2	2	1
yeps_YPTB2689	2	2	2	2	1	1	2	2	2	2	2	1	1	1	1	2	1	1	2	2	1	1	1	2	1	1	1	1	1	1	1	1	2	2	2
yeps_YPTB2693	3	5	3	1	1	1	3	5	5	1	1	3	1	1	1	1	1	4	3	3	1	1	1	3	1	1	1	1	1	1	1	1	1	1	3
yeps_YPTB2697	1	1	1	1	1	1	1	1	1	1	1	1	1	1	1	1	1	1	1	1	1	1	1	1	1	1	1	1	1	1	1	1	1	1	1
yeps_YPTB2698	7	4	3	5	1	1	3	6	4	5	5	1	1	1	1	5	1	4	3	3	1	1	1	3	1	1	1	1	1	1	1	1	5	5	3
yeps_YPTB2700	3	7	3	7	1	7	3	3	7	7	7	3	3	5	7	7	5	3	3	3	7	7	7	3	3	3	7	7	7	7	7	3	7	7	3
yeps_YPTB2701	4	5	4	5	1	1	4	3	5	5	5	3	4	4	1	5	4	3	4	4	1	1	1	4	4	4	1	1	1	1	1	4	5	5	4
yeps_YPTB2703	1	2	1	4	1	1	1	3	2	4	4	1	1	1	1	4	1	1	1	1	1	1	1	1	1	1	1	1	1	1	1	1	4	4	1
yeps_YPTB2704	2	2	2	2	1	1	2	2	2	2	2	2	2	2	1	2	2	1	2	2	1	1	1	2	2	2	1	1	1	1	1	2	2	2	2
yeps_YPTB2711	1	7	1	11	1	1	1	8	7	11	11	3	1	1	1	10	1	4	1	1	1	1	1	1	1	1	1	1	1	1	1	1	11	11	1
yeps_YPTB2713	1	3	1	4	1	1	1	1	3	4	4	1	1	1	1	4	1	1	1	1	1	1	1	1	1	1	1	1	1	1	1	1	4	4	1
yeps_YPTB2717	1	1	1	1	1	1	1	1	1	1	1	1	1	1	1	1	1	1	1	1	1	1	1	1	1	1	1	1	1	1	1	1	1	1	1
yeps_YPTB2718	1	6	1	7	1	1	1	8	7	7	7	2	1	1	1	7	1	3	1	1	1	1	1	1	1	1	1	1	1	1	1	1	7	7	1
yeps_YPTB2719	1	1	1	1	1	1	1	1	1	1	1	1	1	1	1	1	1	4	1	1	1	1	1	1	1	1	1	1	1	1	1	1	1	1	1
yeps_YPTB2721	1	7	1	10	1	1	1	8	7	10	10	1	5	5	1	10	5	3	1	1	1	1	1	1	5	5	1	1	1	1	1	5	10	10	1
yeps_YPTB2737	1	5	1	6	1	1	1	1	5	6	6	3	1	1	1	6	1	4	1	1	1	1	1	1	1	1	1	1	1	1	1	1	6	6	1
yeps_YPTB2739	1	46	1	8	1	1	1	1	6	7	8	1	1	1	1	7	1	3	1	1	1	1	1	1	1	1	1	1	1	1	1	1	8	8	1
yeps_YPTB2741	1	5	1	8	1	1	1	7	6	8	8	3	1	1	1	8	1	4	1	1	1	1	1	1	1	1	1	1	1	1	1	1	8	8	1
yeps_YPTB2742	1	3	1	1	1	1	1	5	3	1	1	3	1	1	1	1	1	1	1	1	1	1	1	1	1	1	1	1	1	1	1	1	1	1	1
yeps_YPTB2743	1	7	1	1	1	1	1	9	7	1	1	3	1	1	1	1	1	4	1	1	1	1	1	1	1	1	1	1	1	1	1	1	1	1	1
yeps_YPTB2744	1	5	1	1	1	1	1	6	1	1	1	2	1	1	1	1	1	1	1	1	1	1	1	1	1	1	1	1	1	1	1	1	1	1	1
yeps_YPTB2755	1	2	1	1	1	1	1	1	2	1	1	1	1	1	1	1	1	1	1	1	1	1	1	1	1	1	1	1	1	1	1	1	1	1	1
yeps_YPTB2756	1	8	1	11	1	1	1	12	8	11	11	3	7	7	1	11	7	5	1	1	1	1	1	1	7	7	1	1	1	1	1	7	11	11	1
yeps_YPTB2757	1	8	1	9	1	1	1	7	8	9	9	2	2	2	1	9	6	4	1	1	1	1	1	1	2	2	1	1	1	1	1	2	9	9	1
yeps_YPTB2758	3	1	3	3	1	1	3	3	1	3	3	3	5	5	1	3	5	1	3	3	1	1	1	3	5	5	1	1	1	1	1	5	3	3	3
yeps_YPTB2759	4	1	4	1	1	1	4	1	1	1	1	1	1	1	1	1	1	1	4	4	1	1	1	4	1	1	1	1	1	1	1	1	1	1	4
yeps_YPTB2764	1	1	1	1	1	1	1	1	1	1	1	2	1	1	1	1	1	1	1	1	1	1	1	1	1	1	1	1	1	1	1	1	1	1	1
yeps_YPTB2765	5	9	5	13	1	1	5	11	9	13	13	4	6	6	1	13	6	6	5	5	1	1	1	5	6	6	1	1	1	1	1	6	13	13	5
yeps_YPTB2766	1	1	1	1	1	1	1	1	1	1	1	1	1	1	1	1	1	3	1	1	1	1	1	1	1	1	1	1	1	1	1	1	1	1	1
yeps_YPTB2773	1	1	1	1	1	1	1	1	1	1	1	1	1	1	1	1	1	1	1	1	1	1	1	1	1	1	1	1	1	1	1	1	1	1	1
yeps_YPTB2774	1	3	1	5	1	1	1	1	3	5	5	1	1	1	1	5	1	3	1	1	1	1	1	1	1	1	1	1	1	1	1	1	5	5	1
yeps_YPTB2776	1	1	1	1	1	1	1	1	1	1	1	1	1	1	1	1	1	1	1	1	1	1	1	1	1	1	1	1	1	1	1	1	1	1	1
yeps_YPTB2777	1	1	1	1	1	1	1	2	1	1	1	1	1	1	1	1	1	1	1	1	1	1	1	1	1	1	1	1	1	1	1	1	1	1	1
yeps_YPTB2782	3	8	3	10	1	1	3	9	8	10	10	3	7	7	1	10	7	7	3	3	1	1	1	3	7	7	1	1	1	1	1	7	10	10	3
yeps_YPTB2783	1	3	1	2	1	1	1	4	3	2	2	1	3	3	1	2	3	2	1	1	1	1	1	1	3	3	1	1	1	1	1	3	2	2	1
yeps_YPTB2784	1	1	1	2	1	1	1	1	1	2	2	1	1	1	1	2	1	1	1	1	1	1	1	1	1	1	1	1	1	1	1	1	2	2	1
yeps_YPTB2786	1	2	1		1	1	1	1	2	1	1	1	1	1	1	1	1	4	1		1	1	1	1	1	1	1	1	1	1	1	1	1	1	1
yeps_YPTB2791	1	1	1	1	1	1	1	3	1	1	1	1	1	1	1	1	1	1	1	1	1	1	1	1	1	1	1	1	1	1	1	1	1	1	1
yeps_YPTB2792	4	5	4	5	1	1	4	2	5	5	5	3	2	2	1	5	2	1	4	4	1	1	1	4	2	2	1	1	1	1	1	2	5	5	4
yeps_YPTB2793	3	4	3	4	1	1	3	3	4	4	4	3	3	3	1	4	3	1	24	3	1	1	1	3	3	3	1	1	1	1	1	3	4	4	3
yeps_YPTB2795	5	8	5	4	1	1	5	1	8	4	4	3	4	4	1	4	4	1	5	5	1	1	1	5	4	4	1	1	1	1	1	4	4	4	5
yeps_YPTB2796	5	9	5	13	1	1	5	10	9	13	13	3	4	4	1	13	4	6	5	5	1	1	1	5	4	4	1	1	1	1	1	4	13	13	5
yeps_YPTB2800	2	6	2	10	1	1	2	8	77	10	10	2	3	3	1	10	3	1	2	2	1	1	1	2	3	3	1	1	1	1	1	3	10	10	2
yeps_YPTB2801	5	8	15	14	1	1	5	12	8	14	14	3	4	4	1	14	4	1	5	5	1	1	1	5	4	4	1	1	1	1	1	4	14	14	5
yeps_YPTB2802	4	7	4	10	1	1	4	8	7	10	10	3	1	1	1	10	1	5	4	4	1	1	1	4	1	1	1	1	1	1	1	1	10	10	4
yeps_YPTB2803	4	8	4	12	1	1	4	10	8	12	12	3	1	1	1	12	1	5	4	4	1	1	1	4	1	1	1	1	1	1	1	1	12	12	4
yeps_YPTB2804	1	1	1	1	1	1	1	1	1	1	1	1	1	1	1	1	1	1	1	1	1	1	1	1	1	1	1	1	1	1	1	1	1	1	1
yeps_YPTB2805	4	10	4	5	1	1	4	11	10	13	5	3	7	7	1	13	7	5	4	4	1	1	1	4	7	7	1	1	1	1	1	7	5	5	4
yeps_YPTB2808	3	8	3	12	14	1	3	10	8	12	12	3	1	1	1	12	1	4	3	3	1	1	1	3	1	33	1	1	1	1	1	1	12	12	3
yeps_YPTB2812	12	7	4	10	1	1	4	8	7	10	65	3	1	1	1	10	1	5	4	4	1	1	1	4	1	1	1	1	1	1	1	1	10	10	4
yeps_YPTB2814	5	10	5	13	1	1	5	11	10	13	13	3	4	4	1	13	4	8	5	5	1	1	1	5	4	4	1	1	1	1	1	4	13	13	5
yeps_YPTB2815	5	10	5	15	1	1	5	11	10	14	15	3	4	4	1	14	4	1	5	5	1	1	1	5	4	4	1	1	1	1	1	4	15	15	5
yeps_YPTB2816	5	9	5	9	1	1	5	5	9	10	9	3	5	5	1	10	5	6	5	5	1	1	1	5	5	5	1	1	1	1	1	5	9	9	5
yeps_YPTB2818	4	4	4	4	1	1	4	3	4	4	4	3	4	4	1	4	4	1	4	4	1	1	1	4	18	4	1	1	1	1	1	4	4	4	4
yeps_YPTB2821	4	5	4	5	1	1	4	2	5	5	5	3	3	3	1	5	3	1	4	4	1	1	1	4	3	3	1	1	1	1	1	3	5	5	4
yeps_YPTB2822	1	1	1	1	1	1	1	5	1	1	1	1	1	1	1	1	1	1	1	1	1	1	1	1	1	1	1	1	1	1	1	1	1	1	1
yeps_YPTB2824	3	5	3	5	1	1	3	6	5	5	5	2	1	1	1	5	1	2	3	3	1	1	1	3	1	1	1	1	1	1	1	1	5	5	3
yeps_YPTB2833	4	7	4	7	1	1	4	8	7	10	7	3	11	1	1	10	1	4	4	91	1	1	1	4	1	1	1	1	1	1	1	1	7	7	4
yeps_YPTB2835	2	1	2	1	1	1	2	2	1	1	1	1	1	1	1	1	1	2	2	2	1	1	1	2	1	1	1	1	1	1	1	1	1	1	2
yeps_YPTB2839	1	3	1	1	1	1	1	1	3	1	1	1	1	1	1	1	1	1	1	1	1	1	1	1	1	1	1	1	1	1	1	1	1	1	1
yeps_YPTB2840	3	1	3	1	1	1	3	6	1	1	1	1	8	1	1	1	1	3	3	3	1	1	1	3	1	1	1	1	1	1	1	1	1	1	3
yeps_YPTB2841	3	3	3	3	1	1	3	3	3	3	3	1	1	1	1	3	1	3	3	3	1	1	1	3	1	1	1	1	1	1	1	1	3	3	3
yeps_YPTB2845	1	1	1	1	1	1	1	6	1	1	1	3	1	1	1	1	1	1	1	1	1	1	1	1	1	1	1	1	1	1	1	1	1	1	1

yeps_YPTB2852	1	6	1	9	1	1	1	7	6	9	9	3	1	1	1	9	1	1	1	1	1	1	1	1	1	1	1	1	1	1	1	1	9	9	1
yeps_YPTB2856	1	2	1	1	1	1	1	2	2	1	1	2	1	1	1	1	1	2	1	1	1	1	1	1	1	1	1	1	1	1	1	1	1	1	1
yeps_YPTB2858	1	3	1	3	1	1	1	5	3	3	3	2	1	1	1	3	1	3	1	1	1	1	1	1	1	1	1	1	1	1	1	1	3	3	1
yeps_YPTB2860	1	1	1	1	1	1	1	1	1	1	1	1	1	1	1	1	1	1	1	1	1	1	1	1	1	1	1	1	1	1	1	1	1	1	1
yeps_YPTB2862	1	1	1	1	1	1	1	3	1	1	1	1	1	1	1	1	1	2	1	1	1	1	1	1	1	1	1	1	1	1	1	1	1	1	1
yeps_YPTB2863	1	1	1	1	1	1	16	3	1	4	1	1	1	1	1	4	1	1	1	1	1	1	1	1	1	1	1	1	1	1	1	1	1	1	1
yeps_YPTB2864	2	2	2	2	2	22	2	2	2	2	2	2	2	2	22	2	2	2	2	2	22	22	22	2	2	2	22	22	22	22	22	2	2	2	2
yeps_YPTB2870	4	1	4	9	1	1	4	1	1	1	9	3	1	1	1	1	1	5	4	4	1	1	1	4	1	1	1	1	1	1	1	1	9	9	4
yeps_YPTB2874	4	7	4	7	1	1	4	8	7	7	7	3	1	1	1	7	1	4	4	4	1	1	1	4	1	1	1	1	1	1	1	1	7	7	4
yeps_YPTB2880	3	7	3	4	1	1	3	8	7	4	4	1	1	1	1	4	1	4	3	3	1	1	1	3	1	1	1	1	1	1	1	1	4	4	3
yeps_YPTB2882	2	6	2	3	1	1	2	7	6	3	3	1	1	1	1	3	1	3	2	2	1	1	1	2	1	1	1	1	1	1	1	1	3	3	2
yeps_YPTB2885	1	6	1	8	1	1	7	1	6	8	8	3	1	1	1	8	1	1	1	7	1	1	1	7	1	1	1	1	1	1	1	1	8	8	1
yeps_YPTB2888	4	3	4	3	1	1	4	3	3	3	3	3	1	1	1	3	1	3	4	4	1	1	1	4	1	1	1	1	1	1	1	1	3	3	4
yeps_YPTB2891	3	5	3	5	1	1	3	2	5	5	5	2	2	2	1	5	2	3	3	3	1	1	1	3	2	2	1	1	1	1	1	2	5	5	3
yeps_YPTB2892	1	5	1	6	1	1	1	4	5	6	6	3	3	3	1	6	3	4	1	1	1	1	1	1	3	3	1	1	1	1	1	3	6	6	1
yeps_YPTB2896	1	2	1	5	1	1	1	4	2	1	5	1	1	1	1	1	1	1	1	1	1	1	1	1	1	1	1	1	1	1	1	1	5	5	1
yeps_YPTB2901	3	6	3	3	1	1	3	7	6	3	3	3	3	3	1	3	3	3	3	3	1	1	1	3	3	3	1	1	1	1	1	3	3	3	3
yeps_YPTB2903	1	1	1	1	1	1	1	1	1	1	1	1	1	1	1	1	1	1	1	1	1	1	1	1	1	1	1	1	1	1	1	1	1	1	1
yeps_YPTB2904	1	4	1	3	1	1	1	4	4	3	3	3	4	4	1	3	4	4	1	1	1	1	1	1	4	4	1	1	1	1	1	4	3	3	1
yeps_YPTB2906	1	9	1	11	1	1	1	4	40	10	11	3	4	4	1	10	4	5	1	1	1	1	1	1	4	4	1	1	1	1	1	4	11	11	1
yeps_YPTB2908	1	1	1	1	1	1	1	1	1	5	1	1	1	1	1	5	1	3	1	1	1	1	1	1	1	1	1	1	1	1	1	1	1	1	1
yeps_YPTB2910	4	1	4	1	1	1	4	1	1	7	1	3	3	3	1	7	3	1	4	4	1	1	1	4	3	3	1	1	1	1	1	3	1	1	4
yeps_YPTB2911	1	5	1	3	1	1	1	1	5	3	3	1	1	1	1	3	1	3	44	1	1	1	1	1	1	1	1	1	1	1	1	1	3	3	1
yeps_YPTB2912	8	1	1	1	1	1	1	1	1	1	1	3	3	3	1	1	3	4	1	1	1	1	1	1	3	3	1	1	1	1	1	3	1	1	1
yeps_YPTB2913	1	7	1	10	1	1	1	8	7	10	10	3	3	3	1	10	3	4	1	1	1	1	1	1	3	3	1	1	1	1	1	3	10	10	1
yeps_YPTB2917	1	10	1	14	1	1	1	11	10	13	14	3	7	7	1	13	7	5	1	1	1	1	1	1	7	7	1	1	1	1	1	7	14	14	1
yeps_YPTB2918	1	5	1	8	1	1	1	6	5	8	8	3	4	4	1	8	4	2	1	1	1	1	1	1	4	4	1	1	1	1	1	4	8	8	1
yeps_YPTB2922	1	1	1	2	1	1	1	1	1	2	2	1	1	1	1	2	1	1	1	1	1	1	1	1	1	1	1	1	1	1	1	1	2	2	1
yeps_YPTB2923	3	5	3	11	1	3	3	8	5	10	11	3	4	4	3	10	4	5	3	3	3	3	3	3	4	4	3	3	3	3	3	4	11	11	3
yeps_YPTB2924	1	5	1	1	1	1	1	2	5	1	1	3	4	4	1	1	4	1	1	1	1	1	1	1	4	4	1	1	1	1	1	4	1	1	1
yeps_YPTB2925	1	6	1	3	1	1	1	2	6	3	3	3	1	1	1	3	1	3	1	1	1	1	1	1	1	1	1	1	1	1	1	1	3	3	1
yeps_YPTB2926	1	4	1	1	1	1	1	1	4	1	1	1	1	1	1	1	1	1	1	1	1	1	1	1	1	1	1	1	1	1	1	1	1	1	1
yeps_YPTB2927	1	6	1	8	1	1	1	7	6	8	8	1	1	1	1	8	1	3	1	1	1	1	1	1	1	1	1	1	1	1	1	1	8	8	1
yeps_YPTB2928	1	5	1	5	1	1	1	6	5	5	5	1	1	1	1	5	1	3	1	1	1	1	1	1	1	1	1	1	1	1	1	1	5	5	1
yeps_YPTB2936	1	5	1	7	1	1	1	3	5	7	7	1	1	1	1	7	1	8	1	1	1	1	1	1	1	1	1	1	1	1	1	1	7	7	1
yeps_YPTB2937	1	6	1	7	1	1	1	7	6	7	7	3	1	1	1	7	1	1	1	1	1	1	1	1	1	1	1	1	1	1	1	1	7	7	1
yeps_YPTB2938	1	5	1	8	1	1	1	6	5	8	8	1	1	1	1	8	1	3	1	1	1	1	1	1	1	1	1	1	1	1	1	1	8	8	1
yeps_YPTB2943	1	5	1	6	1	1	1	2	5	6	6	2	1	1	1	6	1	1	1	1	1	1	1	1	1	1	1	1	1	1	1	1	6	6	1
yeps_YPTB2964	1	3	1	2	1	1	1	4	3	2	2	1	1	1	1	2	1	1	1	1	1	1	1	1	1	1	1	1	1	1	1	1	2	2	1
yeps_YPTB2969	1	3	1	1	1	1	1	1	3	1	1	1	1	1	1	1	1	1	1	1	1	1	1	1	1	1	1	1	1	1	1	1	1	1	1
yeps_YPTB2976	1	4	1	3	1	1	1	1	4	5	3	3	1	1	1	5	1	1	1	1	1	1	1	1	1	1	1	1	1	1	1	1	3	3	1
yeps_YPTB2979	1	1	1	1	1	1	1	1	1	1	1	3	1	1	1	1	1	1	1	1	1	1	1	1	1	1	1	1	1	1	1	1	1	1	1
yeps_YPTB2980	1	4	1	1	1	1	1	1	4	1	1	1	1	1	1	1	1	1	1	1	1	1	1	1	1	1	1	1	1	1	1	1	1	1	1
yeps_YPTB2981	1	1	1	3	1	1	1	1	1	3	3	1	1	1	1	3	1	1	1	1	1	1	1	1	1	1	1	1	1	1	1	1	3	3	1
yeps_YPTB2983	1	1	1	1	1	1	1	1	1	1	1	1	1	1	1	1	1	1	1	1	1	1	1	1	1	1	1	1	1	1	1	1	1	1	1
yeps_YPTB2984	1	2	1	1	1	1	1	1	2	1	1	1	1	1	1	1	1	2	1	1	1	1	1	1	1	1	1	1	1	1	1	1	1	1	1
yeps_YPTB2985	4	3	4	7	1	1	4	7	3	7	7	3	1	1	1	7	1	5	4	4	1	1	1	4	1	1	1	1	1	1	1	1	7	7	4
yeps_YPTB2986	2	2	2	1	1	1	2	1	2	1	1	2	1	1	1	1	1	3	2	2	1	1	1	2	1	1	1	1	1	1	1	1	1	1	2
yeps_YPTB2988	3	7	3	12	1	1	3	7	7	10	12	1	1	1	1	10	1	4	3	3	1	1	1	3	1	1	1	1	1	1	1	1	12	12	3
yeps_YPTB2989	1	1	1	4	1	1	1	1	1	4	4	1	1	1	1	4	1	2	1	1	1	1	1	1	1	1	1	1	1	1	1	1	4	4	1
yeps_YPTB2991	1	6	1	1	1	1	1	7	6	1	1	1	1	1	1	1	1	3	1	1	1	1	1	1	1	1	1	1	1	1	1	1	1	1	1
yeps_YPTB2995	1	2	1	2	1	1	1	1	2	2	2	1	1	1	1	2	1	2	1	1	1	1	1	1	1	1	1	1	1	1	1	1	2	2	1
yeps_YPTB2996	1	1	1	2	1	1	1	2	1	2	2	1	1	1	1	2	1	5	1	1	1	1	1	1	1	1	1	1	1	1	1	1	2	2	1
yeps_YPTB2999	6	5	6	2	1	1	6	5	5	2	2	1	1	1	1	2	1	7	6	6	1	1	1	6	1	1	1	1	1	1	1	1	2	2	6
yeps_YPTB3000	1	1	1	1	1	1	1	3	1	1	1	1	1	1	1	1	1	1	1	1	1	1	1	1	1	1	1	1	1	1	1	1	1	1	1
yeps_YPTB3001	1	1	1	1	1	1	1	1	1	1	1	1	1	1	1	1	1	1	1	1	1	1	1	1	1	1	1	1	1	1	1	1	1	1	1
yeps_YPTB3002	1	1	1	1	1	1	1	1	1	1	1	1	1	1	1	1	1	2	1	1	1	1	1	1	1	1	1	1	1	1	1	1	1	1	1
yeps_YPTB3007	2	1	2	1	1	1	2	2	1	1	1	1	1	1	1	1	1	1	2	2	1	1	1	2	1	1	1	1	1	1	1	1	1	1	2
yeps_YPTB3008	5	7	5	8	1	4	5	6	7	8	8	3	4	4	4	8	4	6	5	5	4	4	4	5	4	4	4	4	4	4	4	4	8	8	5
yeps_YPTB3009	2	2	2	5	1	1	2	4	2	2	5	2	1	1	1	2	1	2	2	2	1	1	1	2	1	1	1	1	1	1	1	1	5	5	2
yeps_YPTB3016	1	1	1	1	1	1	1	1	1	1	1	1	1	1	1	1	1	1	1	1	1	1	1	1	1	1	1	1	1	1	1	1	1	1	1
yeps_YPTB3019	1	3	1	3	1	1	1	5	3	3	3	3	3	3	1	3	3	3	1	1	1	1	1	1	3	3	1	1	1	1	1	3	3	3	1
yeps_YPTB3027	2	8	2	10	1	1	2	2	8	10	10	1	1	1	1	10	1	14	2	2	1	1	1	2	1	1	1	1	1	1	1	1	10	10	2
yeps_YPTB3028	1	1	1	1	1	1	1	1	1	1	1	1	1	1	1	1	1	1	1	1	1	1	1	1	1	1	1	1	1	1	1	1	1	1	1
yeps_YPTB3029	1	1	1	5	1	1	1	1	1	1	5	1	1	1	1	1	1	1	1	1	1	1	1	1	1	1	1	1	1	1	1	1	5	5	1
yeps_YPTB3030	1	5	1	1	1	1	1	2	5	1	1	1	1	1	1	1	1	1	1	1	1	1	1	1	1	1	1	1	1	1	1	1	1	1	1
yeps_YPTB3031	5	3	1		1	1	1	1	3	4		1	1	1	1	4	1	1	1	1	1	1	1	1	1	1	1	1	1	1	1	1			1
yeps_YPTB3044	3	4	3	6	1	1	3	5	4	6	6	1	1	1	1	6	1	2	3	3	1	1	1	3	1	1	1	1	1	1	1	1	6	6	3
yeps_YPTB3045	3	7	3	9	1	1	3	3	7	3	9	1	1	1	1	3	1	4	3	3	1	1	1	3	1	1	1	1	1	1	1	1	9	9	3
yeps_YPTB3046	3	6	3	9	3	3	3	7	6	8	9	3	3	3	3	8	3	4	3	3	3	3	3	3	3	3	3	3	3	3	3	3	9	9	3
yeps_YPTB3048	1	4	1	5	1	1	1	2	4	2	5	1	1	1	1	2	1	3	1	1	1	1	1	1	1	1	1	1	1	1	1	1	5	5	1
yeps_YPTB3049	1	4	1	2	1	1	1	3	4	2	2	1	1	1	1	2	1	3	1	1	1	1	1	1	1	1	1	1	1	1	1	1	2	2	1
yeps_YPTB3076	4	6	4	8	1	1	4	7	6	8	8	3	4	4	1	8	4	4	4	4	1	1	1	4	4	4	1	1	1	1	1	4	8	8	4
yeps_YPTB3077	5	8	5	10	1	1	5	2	8	10	10	3	7	7	1	10	7	5	5	5	1	1	1	5	7	7	1	1	1	1	1	7	10	10	5
yeps_YPTB3078	2	3	2	2	1	1	2	2	3	2	2	2	1	1	1	2	1	2	2	2	1	1	1	2	1	1	1	1	1	1	1	1	2	2	2
yeps_YPTB3079	5	8	5	5	1	1	5	7	8	9	5	3	1	1	1	9	1	5	5	5	1	1	1	5	1	1	1	1	1	1	1	1	5	5	5
yeps_YPTB3080	4	7	4	4	1	1	4	8	7	10	4	3	3	3	1	10	3	4	4	4	1	1	1	4	3	3	1	1	1	1	1	3	4	4	4
yeps_YPTB3081	3	3	3	7	1	1	3	3	3	6	7	1	1	1	1	6	1	3	3	3	1	1	1	3	1	1	1	1	1	1	1	1	7	7	3
yeps_YPTB3082	6	9	6	11	1	1	6	6	9	10	11	3	3	3	1	10	3	6	6	6	1	1	1	6	3	3	1	1	1	1	1	3	11	11	6
yeps_YPTB3083	1	2	1	6	1	1	1	1	2	5	6	2	1	1	1	5	1	1	1	1	1	1	1	1	1	1	1	1	1	1	1	1	6	6	1
yeps_YPTB3087	4	2	4	8	1	1	4	7	2	8	8	3	1	1	1	8	1	4	4	4	1	1	1	4	1	1	1	1	1	1	1	1	8	8	4
yeps_YPTB3088	3	5	3	5	1	1	3	3	5	6	5	1	1	1	1	6	1	3	3	3	1	1	1	3	1	1	1	1	1	1	1	1	5	5	3
yeps_YPTB3089	1	5	1	4	1	32	1	1	5	6	4	1	1	1	32	6	1	1	1	1	32	32	32	1	1	1	32	32	32	32	32	1	4	4	1
yeps_YPTB3097	4	8	4	8	1	1	4	8	8	8	8	3	3	3	1	8	3	5	4	4	1	1	1	4	3	3	1	1	1	1	1	3	8	8	4
yeps_YPTB3098	1	5	1	7	1	1	1	1	5	2	7	2	2	2	1	2	2	1	3	3	1	1	1	3	2	2	1	1	1	1	1	2	7	7	6
yeps_YPTB3099	1	1	6	2	1	1	1	4	1	2	2	1	2	2	1	2	2	5	1	1	1	1	1	1	2	2	1	1	1	1	1	2	2	2	1
yeps_YPTB3100	1	4	1	4	1	1	1	5	4	4	4	3	3	3	1	4	3	3	1	1	1	1	1	1	3	3	1	1	1	1	1	3	4	4	1
yeps_YPTB3101	1	6	1	5	1	1	1	4	6	5	5	2	2	2	1	5	2	4	1	10	1	1	1	10	2	2	1	1	1	1	1	2	5	5	1
yeps_YPTB3103	5	7	5	11	1	1	5	9	7	11	11	3	3	3	1	11	3	5	5	5	1	1	1	5	3	3	1	1	1	1	1	3	11	11	5
yeps_YPTB3117	3	4	3	6	1	1	3	5	4	6	6	1	1	1	1	6	1	3	3	3	1	1	1	3	1	1	1	1	1	1	1	1	6	6	3
yeps_YPTB3118	2	2	2	1	1	1	2	2	2	1	1	1	2	2	1	1	2	2	2	2	1	1	1	2	11	11	1	1	1	1	1	2	1	1	2
yeps_YPTB3166	1	6	1	1	1	1	1	1	6	7	1	1	5	5	1	7	5	1	1	1	1	1	1	1	5	5	1	1	1	1	1	5	1	1	1
yeps_YPTB3168	2	3	4	3	1	1	2	3	3	3	3	2	3	3	1	3	3	1	2	2	1	1	1	2	3	3	1	1	1	1	1	3	3	3	2
yeps_YPTB3171	3	1	3	1	1	1	3	1	1	1	1	1	1	1	1	1	1	1	3	3	1	1	1	3	1	1	1	1	1	1	1	1	1	1	3
yeps_YPTB3172	1	1	1	1	1	1	1	1	1	1	1	3	3	3	1	1	3	1	1	1	1	1	1	1	3	3	1	1	1	1	1	3	1	1	1
yeps_YPTB3173	1	1	1	1	1	1	1	1	1	1	1	1	1	1	1	1	1	1	1	1	1	1	1	1	1	1	1	1	1	1	1	1	1	1	1
yeps_YPTB3174	5	5	5	7	1	1	5	1	5	7	7	3	5	5	1	7	5	1	5	5	1	1	1	5	5	5	1	1	1	1	1	5	7	7	5
yeps_YPTB3175	2	3		1	1	1	2	1	3	1	1	1	1	1	1	1	1	1	2	2	1	1	1	2	1	1	1	1	1	1	1	1	1	1	2
yeps_YPTB3176	3	2	3	6	1	1	3	3	40	6	6	21	3	3	1	6	3	1	3	3	1	1	1	3	3	3	1	1	1	1	1	3	6	6	3
yeps_YPTB3184	4	7	4	9	1	1	4	8	7	9	9	3	3	3	1	9	3	1	4	4	1	1	1	4	3	3	1	1	1	1	1	3	9	9	4
yeps_YPTB3185	4	7	4	7	1	1	4	8	7	7	7	3	3	3	1	7	3	1	4	4	1	1	1	4	3	3	1	1	1	1	1	3	7	7	4
yeps_YPTB3186	1	1	1	3	1	1	1	1	1	3	3	2	2	2	1	3	2	1	1	1	1	1	1	1	2	2	1	1	1	1	1	2	3	3	1
yeps_YPTB3187	1	1	1	1	1	1	1	3	1	1	1	1	2	2	1	1	2	1	1	1	1	1	1	1	2	2	1	1	1	1	1	2	1	1	1
yeps_YPTB3190	1	1	1	1	1	1	1	1	1	1	1	3	1	1	1	1	1	5	1	1	1	1	1	1	1	1	1	1	1	1	1	1	1	1	1
yeps_YPTB3191	1	10	1	12	1	1	1	11	10	12	12	3	1	1	1	12	1	6	1	1	1	1	1	1	1	1	1	1	1	1	1	1	12	12	1
yeps_YPTB3192	1	1	1	1	1	1	1	1	1	1	1	1	1	1	1	1	12	3	1	1	1	1	1	1	1	1	1	1	1	1	1	1	1	1	1
yeps_YPTB3203	3	5	3	7	1	1	3	1	5	7	7	1	1	1	1	7	1	3	3	3	1	1	1	3	1	1	1	1	1	1	1	1	7	7	3
yeps_YPTB3206	3	5	3	1	1	1	3	2	5	1	1	1	1	1	1	1	1	3	3	3	1	1	1	3	1	1	1	1	1	1	1	1	1	1	3
yeps_YPTB3207	2	5	2	7	1	1	2	6	5	7	7	1	1	1	1	7	1	3	2	2	1	1	1	2	1	1	1	1	1	1	1	1	7	7	2
yeps_YPTB3208	1	1	1	1	1	1	1	1	1	1	1	1	1	1	1	1	1	1	1	1	1	1	1	1	1	1	1	1	1	1	1	1	1	1	1
yeps_YPTB3209	2	2	2	2	1	1	2	2	2	2	2	2	1	1	1	2	1	3	2	2	1	1	1	2	1	1	1	1	1	1	1	1	2	2	2
yeps_YPTB3211	5	39	5	13	1	1	5	11	10	12	13	3	4	4	1	12	4	8	5	5	1	1	1	5	4	4	1	1	1	1	1	4	13	13	5
yeps_YPTB3212	1	6	1	1	1	1	1	7	6	8	1	3	1	1	1	8	1	1	1	1	1	1	1	1	1	1	1	1	1	1	1	1	1	1	1
yeps_YPTB3213	1	1	1	7	1	1	1	4	1	1	7	1	1	1	1	1	1	1	1	1	1	1	1	1	1	1	1	1	1	1	1	1	7	7	1
yeps_YPTB3214	1	6	1	9	1	1	1	7	6	8	9	3	3	3	1	8	3	1	1	1	1	1	1	1	3	3	1	1	1	1	1	3	9	9	1
yeps_YPTB3216	1	1	1	7	1	1	1	1	1	6	7	2	1	1	1	6	1	1	1	1	1	1	1	1	1	1	1	1	1	1	1	1	7	7	1
yeps_YPTB3221	1	6	1	1	1	1	1	1	15	1	1	1	1	1	1	1	1	1	1	1	1	1	1	1	1	1	1	1	1	1	1	1	1	1	1
yeps_YPTB3223	1	2	1	6	1	1	1	5	2	6	6	1	1	1	1	6	1	3	1	1	1	1	1	1	1	1	1	1	1	1	1	1	6	6	1
yeps_YPTB3231	1	5	1	4	1	1	1	7	5	4	4	1	1	1	1	4	1	4	1	1	1	1	1	1	1	1	1	1	1	1	1	1	4	4	1
yeps_YPTB3258	1	1	1	1	1	1	1	1	1	1	1	1	1	1	1	1	1	1	1	1	1	1	1	1	1	1	1	1	1	1	1	1	1	1	2
yeps_YPTB3261	1	6	1	2	1	1	1	3	6	2	2	3	1	1	1	2	1	5	1	1	1	1	1	1	1	1	1	1	1	1	1	1	2	2	1
yeps_YPTB3262	1	9	1	11	1	1	1	3	9	11	11	3	1	1	1	11	1	6	1	1	1	1	1	1	1	1	1	1	1	1	1	1	11	11	1
yeps_YPTB3263	1	4	1	1	1	1	1	5	4	1	1	1	1	1	1	1	1	1	1	1	1	1	1	1	1	1	1	1	1	1	1	1	1	1	1
yeps_YPTB3264	1	6	1	1	1	1	1	7	6	1	1	3	1	1	1	1	1	4	1	1	1	1	1	1	1	1	1	1	1	1	1	1	1	1	1
yeps_YPTB3265	1	8	1	12	1	50	1	9	8	11	12	3	1	1	50	11	1	5	1	1	50	50	50	1	1	1	50	50	50	50	50	1	12	12	1
yeps_YPTB3266	1	6	1	11	1	1	1	7	6	9	11	3	1	1	1	9	1	4	1	1	1	1	1	1	1	1	1	1	1	1	1	1	11	11	1
yeps_YPTB3267	1	7	1	11	1	3	1	3	7	9	11	3	1	79	3	9	1	5	1	1	3	3	3	1	1	1	3	3	3	3	3	1	11	11	1
yeps_YPTB3269	5	8	5	9	1	4	5	1	8	9	9	3	1	1	4	9	1	6	5	5	4	4	4	5	1	1	4	4	4	4	4	1	9	9	5
yeps_YPTB3270	1	7	1	9	1	4	1	8	7	9	9	3	1	1	4	9	1	5	1	1	4	4	4	1	1	1	4	4	4	4	4	1	9	9	1
yeps_YPTB3290	3	6	3	8	1	1	3	7	6	8	8	1	1	1	1	8	1	4	3	3	1	1	1	3	1	1	1	1	1	1	1	1	8	8	3
yeps_YPTB3291	5	8	5	11	4	4	5	9	8	11	11	3	4	4	4	11	4	6	5	5	4	4	4	5	4	4	4	4	4	4	4	4	11	11	5
yeps_YPTB3294	4	7	4	9	1	1	4	8	7	9	9	3	1	1	1	9	1	5	4	4	1	1	1	4	1	1	1	1	1	1	1	1	9	9	4
yeps_YPTB3295	4	6	4	9	1	1	4	7	6	9	9	3	1	1	1	9	1	2	4	4	1	1	1	4	1	1	1	1	1	1	1	1	9	9	4
yeps_YPTB3355	1	4	1	5	1	1	1	1	4	5	5	3	1	1	1	5	1	25	1	1	1	1	1	1	1	1	1	1	1	1	1	1	5	5	1
yeps_YPTB3382	1	5	1	6	1	1	1	3	5	6	6	3	1	1	1	6	1	1	1	1	1	1	1	1	1	1	1	1	1	1	1	1	6	6	1
yeps_YPTB3384	4	6	4	10	1	1	4	9	6	10	10	3	1	1	1	10	1	5	4	4	1	1	1	4	1	1	1	1	1	1	1	1	10	10	4
yeps_YPTB3385	2	6	2	2	1	1	2	7	6	2	2	3	1	1	1	2	1	4	2	2	1	1	1	2	1	1	1	1	1	1	1	1	2	2	2
yeps_YPTB3386	1	3	1	6	1	1	1	4	3	6	6	1	1	1	1	6	1	1	1	1	1	1	1	1	1	1	1	1	1	1	1	1	6	6	1
yeps_YPTB3387	4	7	4	10	1	1	4	8	7	10	10	3	1	1	1	10	1	5	4	4	1	1	1	4	1	1	1	1	1	1	1	1	10	10	4
yeps_YPTB3388	4	9	4	11	1	1	4	10	9	11	11	3	1	1	1	11	1	7	4	4	1	1	1	4	1	1	1	1	1	1	1	1	11	11	4
yeps_YPTB3390	1	4	1	11	1	1	1	9	4	11	11	1	1	1	1	11	1	3	1	1	1	1	1	1	1	1	1	1	1	1	1	1	11	11	1
yeps_YPTB3391	1	2	1	5	1	1	1	4	2	5	5	1	1	1	1	5	1	3	1	1	1	1	1	1	1	1	1	1	1	1	1	1	5	5	1
yeps_YPTB3392	1	7	1	10	1	1	1	8	7	10	10	1	1	1	1	10	1	4	1	1	1	1	1	1	1	1	1	1	1	1	1	1	10	10	1
yeps_YPTB3393	1	1	1	1	1	1	1	3	1	1	1	1	1	1	1	1	1	1	1	1	1	1	1	1	1	1	1	1	1	1	1	1	1	1	1
yeps_YPTB3394	1	3	1	6	1	1	1	5	3	6	6	1	1	1	1	6	1	3	1	1	1	1	1	1	1	1	1	1	1	1	1	1	6	6	1
yeps_YPTB3399	1	1	1	1	1	1	1	4	1	1	1	1	1	1	1	1	1	1	1	1	1	1	1	1	1	1	1	1	1	1	1	1	1	1	1
yeps_YPTB3402	3	2	3	1	1	51	3	5	2	1	1	3	1	1	51	1	1	2	3	3	51	51	51	3	1	1	51	51	51	51	51	1	1	1	3
yeps_YPTB3403	1	6	1	2	1	1	1	8	6	2	2	2	1	1	1	2	1	3	1	1	1	1	1	1	1	1	1	1	1	1	1	1	2	2	1
yeps_YPTB3404	1	3	1	1	1	1	1	6	3	1	1	1	4	4	1	1	4	3	1	1	1	1	1	1	4	4	1	1	1	1	1	4	1	1	1
yeps_YPTB3408	1	5	1	1	1	1	1	6	5	1	1	1	1	1	1	1	1	4	1	1	1	1	1	1	1	1	1	1	1	1	1	1	1	1	1
yeps_YPTB3409	1	6	1	12	1	3	1	8	7	10	12	3	3	3	3	10	3	4	1	1	3	3	3	1	3	3	3	3	3	3	3	3	12	12	1
yeps_YPTB3410	1	2	1	3	1	1	1	2	2	2	3	1	1	1	1	2	1	3	1	1	1	1	1	1	1	1	1	1	1	1	1	1	3	3	1
yeps_YPTB3411	1	3	1	5	1	1	1	4	51	1	5	1	1	1	1	1	1	2	1	1	1	1	1	1	1	1	1	1	1	1	1	1	5	5	1
yeps_YPTB3412	2	1	2	1	1	1	2	1	1	1	1	1	1	1	1	1	1	3	2	2	1	1	1	2	1	1	1	1	1	1	1	1	1	1	2
yeps_YPTB3414	2	4	2	4	1	1	2	1	4	4	4	1	1	1	1	4	1	3	2	2	1	1	1	2	1	1	1	1	1	1	1	1	4	4	2
yeps_YPTB3415	7	6	8	7	1	1	8	1	6	7	7	2	1	1	1	7	1	1	7	7	1	1	1	7	1	1	1	1	1	1	1	1	7	7	7
yeps_YPTB3417	3	4	3	7	1	1	3	6	4	2	7	1	1	1	1	2	1	2	3	3	1	1	1	3	1	1	1	1	1	1	1	1	7	7	3
yeps_YPTB3434	1	1	1	1	1	1	1	1	1	1	1	1	1	1	1	1	1	1	1	1	1	1	1	1	1	1	1	1	1	1	1	1	1	1	1
yeps_YPTB3439	5	9	5	12	1	1	5	10	9	12	12	118	5	5	1	12	5	6	5	5	1	1	1	5	5	5	1	1	1	1	1	5	12	12	5
yeps_YPTB3440	5	1	5	4	1	1	5	4	1	4	4	3	5	5	1	4	5	1	5	5	1	1	1	5	5	5	1	1	1	1	1	5	4	4	5
yeps_YPTB3441	4	4	4	4	1	4	4	6	21	4	4	3	4	4	4	4	4	4	4	4	4	4	4	4	4	4	4	4	4	4	4	4	4	4	4
yeps_YPTB3442	1	7	1	9	1	5	1	8	7	9	9	3	1	1	5	9	1	5	1	1	5	5	5	1	1	1	5	5	5	5	5	1	9	9	1
yeps_YPTB3443	1	4	1	8	1	4	1	7	4	8	8	3	1	1	4	8	1	4	1	1	4	4	4	1	1	1	4	4	4	4	4	1	8	8	1
yeps_YPTB3444	1	6	1	8	1	3	1	2	6	8	8	2	1	1	3	8	1	3	1	1	3	3	3	1	1	1	3	3	3	3	3	1	8	8	1
yeps_YPTB3445	1	10	1	16	1	17	1	13	10	15	16	3	8	8	17	15	8	6	1	1	17	17	17	1	8	8	17	17	17	17	17	8	16	16	12
yeps_YPTB3446	1	6	1	8	1	1	1	7	6	8	8	1	1	1	1	8	1	1	1	1	1	1	1	1	1	1	1	1	1	1	1	1	8	8	1

yeps_YPTB3447	1	6	1	9	1	1	1	7	6	9	9	1	1	1	1	9	1	3	1	1	1	1	1	1	1	1	1	1	1	1	1	1	9	9	1
yeps_YPTB3448	1	6	1	10	1	1	1	7	6	9	10	3	1	1	1	9	1	4	1	1	1	1	1	1	1	1	1	1	1	1	1	1	10	10	1
yeps_YPTB3468	2	3	2	5	1	1	2	3	3	2	5	2	1	29	1	2	1	3	2	2	1	1	1	2	1	1	1	1	1	1	1	1	5	5	2
yeps_YPTB3469	4	7	4	10	1	1	4	8	7	10	10	3	1	1	1	10	1	5	4	4	1	1	1	4	1	1	1	1	1	1	1	1	10	10	4
yeps_YPTB3472	1	4	1	1	1	1	1	1	4	1	1	3	1	1	1	1	1	1	1	1	1	1	1	1	1	1	1	1	1	1	1	1	1	1	1
yeps_YPTB3473	1	1	1	1	1	35	1	5	1	1	1	3	3	3	35	1	3	1	1	1	35	35	35	1	3	3	35	35	35	35	35	3	1	1	1
yeps_YPTB3475	1	5	1	3	1	1	1	3	5	3	3	3	3	3	1	3	3	1	1	1	1	1	1	1	3	3	1	1	1	1	1	3	3	3	1
yeps_YPTB3477	1	6	1	8	1	1	1	10	6	8	8	3	3	3	1	8	3	1	1	1	1	1	1	1	3	3	1	1	1	1	1	3	8	8	1
yeps_YPTB3480	1	1	1	1	1	1	1	4	1	1	1	1	1	1	1	1	1	2	1	1	1	1	1	1	1	1	1	1	1	1	1	1	1	1	1
yeps_YPTB3481	1	1	1	1	1	1	1	4	1	31	1	1	1	1	1	1	1	1	1	1	1	1	1	1	1	1	1	1	1	1	1	1	1	1	1
yeps_YPTB3482	1	1	1	1	1	1	1	1	1	1	1	1	1	1	1	1	1	1	1	1	1	1	1	1	1	1	1	1	1	1	1	1	1	1	1
yeps_YPTB3483	1	5	1	1	1	1	1	1	5	1	1	1	1	1	1	1	1	1	1	1	1	1	1	1	1	1	1	1	1	1	1	1	1	1	1
yeps_YPTB3484	1	1	1	1	1	1	1	1	1	1	1	1	1	1	1	1	1	1	1	1	1	1	1	1	1	1	1	1	1	1	1	1	1	1	1
yeps_YPTB3486	1	2	1	2	1	1	1	2	2	2	2	1	1	1	1	2	1	2	1	1	1	1	1	1	1	1	1	1	1	1	1	1	2	2	1
yeps_YPTB3487	2	2	2	2	1	1	2	2	2	2	2	2	1	1	1	2	1	2	2	2	1	1	1	2	1	1	1	1	1	1	1	1	2	2	2
yeps_YPTB3489	3	6	3	3	1	1	3	8	6	3	3	1	1	1	1	3	1	1	3	3	1	1	1	3	1	1	1	1	1	1	1	1	3	3	3
yeps_YPTB3490	3	6	3	7	1	1	3	2	6	7	7	2	1	1	1	7	1	2	3	3	1	1	1	3	1	1	1	1	1	1	1	1	7	7	3
yeps_YPTB3494	1	1	1	1	1	1	1	1	1	1	1	1	1	1	1	1	1	1	1	1	1	1	1	1	1	1	1	1	1	1	1	1	1	1	1
yeps_YPTB3495	3	5	3	1	1	1	3	6	5	1	1	1	1	1	1	1	1	4	3	3	1	1	1	3	1	1	1	1	1	1	1	1	1	1	3
yeps_YPTB3496	3	3	3	5	1	1	3	3	3	5	5	1	1	1	1	5	1	4	3	3	1	1	1	3	1	1	1	1	1	1	1	1	5	5	3
yeps_YPTB3497	3	7	3	3	1	1	3	4	7	3	3	3	1	1	1	3	1	4	3	3	1	1	1	3	1	1	1	1	1	1	1	1	3	3	3
yeps_YPTB3499	2	2	2	2	2	2	2	2	2	2	2	2	2	2	2	2	2	2	2	2	2	2	2	2	2	2	2	2	2	2	2	2	2	2	2
yeps_YPTB3500	3	8	3	10	1	1	3	9	8	10	10	2	1	1	1	10	1	4	3	3	1	1	1	3	1	1	1	1	1	1	1	1	10	10	3
yeps_YPTB3501	3	3	3	5	1	1	3		3	5	5	1	1	1	1	5	1	2	3	3	1	1	1	3	1	1	1	1	1	1	1	1	5	5	3
yeps_YPTB3505	2	2	2	3	1	2	2	3	2	3	3	3	1	1	2	3	1	2	2	2	2	2	2	2	1	1	2	2	2	2	2	1	3	3	2
yeps_YPTB3509	1	1	1	5	1	1	1	1	1	5	5	1	1	1	1	5	1	3	1	1	1	1	1	1	1	1	1	1	1	1	1	1	5	5	1
yeps_YPTB3511	1	2	1	2	1	1	1	5	2	2	2	1	1	1	1	2	1	1	1	1	1	1	1	1	1	1	1	1	1	1	1	1	2	2	1
yeps_YPTB3512	1	2	1	2	1	1	1	1	2	2	2	1	1	1	1	2	1	3	1	1	1	1	1	1	1	1	1	1	1	1	1	1	2	2	1
yeps_YPTB3513	1	7	1	10	1	1	1	8	7	10	10	3	1	6	1	10	6	4	1	1	1	1	1	1	1	1	1	1	1	1	1	1	10	10	1
yeps_YPTB3514	1	1	1	1	1	1	1	1	1	1	1	1	1	1	1	1	1	1	1	1	1	1	1	1	1	1	1	1	1	1	1	1	1	1	1
yeps_YPTB3516	2	1	2	1	1	1	2	1	1	1	1	2	1	1	1	1	1	3	2	2	1	1	1	2	1	1	1	1	1	1	1	1	1	1	4
yeps_YPTB3517	3	3	3	3	1	3	3	2	3	3	3	3	3	3	3	3	3	3	3	3	3	3	3	3	3	3	3	3	3	3	3	3	3	3	3
yeps_YPTB3519	1	4	5	2	1	1	1	2	4	2	2	1	1	1	1	2	1	1	1	1	1	1	1	1	1	1	1	1	1	1	1	1	2	2	1
yeps_YPTB3520	1	1	1	1	1	1	1	3	1	1	1	2	1	1	1	1	1	1	1	1	1	1	1	1	1	1	1	1	1	1	1	1	1	1	1
yeps_YPTB3521	1	4	1	1	1	1	1	1	4	1	1	2	1	1	1	1	1	3	1	1	1	1	1	1	1	1	1	1	1	1	1	1	1	1	1
yeps_YPTB3522	1	2	1	2	1	1	1	1	2	2	2	3	1	1	1	2	1	1	1	1	1	1	1	1	1	1	1	1	1	1	1	1	2	2	1
yeps_YPTB3529	3	1	3	1	1	3	3	1	1	1	1	1	1	1	3	1	1	4	3	3	3	3	3	3	1	1	3	3	3	3	3	1	1	1	3
yeps_YPTB3530	1	2	1	2	1	1	1	2	2	2	2	3	1	1	1	2	1	2	1	1	1	1	1	1	1	1	1	1	1	1	1	1	2	2	1
yeps_YPTB3532	2	2	2	2	1	2	2	4	2	2	2	3	1	1	2	2	1	2	2	2	2	2	2	2	1	1	2	2	2	2	2	1	2	2	2
yeps_YPTB3541	3	7	3	9	1	1	3	8	7	9	9	27	1	1	1	9	1	4	3	3	1	1	1	3	1	1	1	1	1	1	1	1	9	9	3
yeps_YPTB3542	1	4	1	5	1	1	1	4	4	5	5	3	1	1	1	5	1	3	1	1	1	1	1	1	1	1	1	1	1	1	1	1	5	5	1
yeps_YPTB3543	1	1	1	2	1	1	1	1	1	2	2	1	1	1	1	2	1	1	1	1	1	1	1	1	1	1	1	1	1	1	1	1	2	2	1
yeps_YPTB3544	1	2	1	2	1	1	1	5	2	2	2	1	1	1	1	2	1	2	1	1	1	1	1	1	1	1	1	1	1	1	1	1	2	2	1
yeps_YPTB3546	1	7	1	9	1	3	1	8	7	9	9	1	1	1	3	9	1	3	1	1	3	3	3	1	1	1	3	3	3	3	3	1	9	9	1
yeps_YPTB3566	9	8	9	12	1	1	9	8	8	11	12	3	1	1	1	11	1	5	9	9	1	1	1	9	1	1	1	1	1	1	1	1	12	12	9
yeps_YPTB3567	10	7	1	9	1	1	1	8	7	9	9	3	1	1	1	9	1	4	1	1	1	1	1	1	1	1	1	1	1	1	1	1	9	9	1
yeps_YPTB3571	1	7	1	8	1	1	1	1	7	8	8	1	1	1	1	8	1	4	1	1	1	1	1	1	1	1	1	1	1	1	1	1	8	8	1
yeps_YPTB3579	1	3	1	2	1	1	1	5	3	2	2	3	1	1	1	2	1	4	1	1	1	1	1	1	1	1	1	1	1	1	1	1	2	2	1
yeps_YPTB3580	9	43	9		1	1	9	11	8	12	12	3	1	1	1	12	1	5	9	9	1	1	1	9	1	1	1	1	1	1	1	1	12	12	9
yeps_YPTB3581	3	1	3	1	1	1	3	2	1	1	1	2	1	1	1	1	1	1	3	3	1	1	1	3	1	1	1	1	1	1	1	1	1	1	3
yeps_YPTB3582	4	6	4	6	1	1	4	7	6	6	6	3	1	1	1	6	1	5	4	4	1	1	1	4	1	1	1	1	1	1	1	1	6	6	4
yeps_YPTB3589	1	4	1	7	1	1	1	5	4	7	7	1	1	1	1	7	1	1	1	1	1	1	1	1	1	1	1	1	1	1	1	1	7	7	1
yeps_YPTB3590	3	6	3	8	1	1	3	7	6	8	8	1	1	1	1	8	1	1	3	3	1	1	1	3	1	1	1	1	1	1	1	1	8	8	3
yeps_YPTB3591	2	1	2	7	1	1	2	5	1	7	7	1	1	1	1	7	1	1	2	2	1	1	1	2	1	1	1	1	1	1	1	1	7	7	2
yeps_YPTB3592	1	8	1	10	1	1	1	9	8	10	10	3	1	1	1	10	1	4	1	1	1	1	1	1	1	1	1	1	1	1	1	1	10	10	1
yeps_YPTB3593	1	9	1	9	1	1	1	10	9	9	9	3	1	1	1	9	1	5	1	1	1	1	1	1	1	1	1	1	1	1	1	1	9	9	1
yeps_YPTB3595	1	3	1	8	1	1	1	1	3	8	8	3	1	1	1	8	1	1	1	1	1	1	1	1	1	1	1	1	1	1	1	1	8	8	1
yeps_YPTB3596	4	6	4	10	1	4	4	8	6	10	10	3	1	1	4	10	1	5	4	4	4	4	4	4	1	1	4	4	4	4	4	1	10	10	4
yeps_YPTB3597	3	8	3	11	1	3	3	9	8	11	11	1	1	1	3	11	1	4	3	3	3	3	3	3	1	1	3	3	3	3	3	1	11	11	3
yeps_YPTB3598	61	9	3	9	1	3	3	10	9	9	9	1	1	1	3	9	1	6	3	3	3	3	3	3	1	1	3	3	3	3	3	1	9	9	3
yeps_YPTB3599	4	8	4	10	1	4	4	9	8	10	10	3	1	1	4	10	1	3	4	4	4	4	4	4	1	1	4	4	4	4	4	1	10	10	4
yeps_YPTB3603	5	5	5	10	1	31	5	8	5	10	10	3	1	1	31	10	1	5	5	5	31	31	31	5	1	1	31	31	31	31	31	1	10	10	5
yeps_YPTB3604	3	5	3	7	1	1	3	6	5	7	7	1	1	1	1	7	1	4	3	3	1	1	1	3	1	1	1	1	1	1	1	1	7	7	3
yeps_YPTB3607	4	7	4	10	1	1	4	7	7	10	10	3	1	1	1	10	1	4	4	4	1	1	1	4	1	1	1	1	1	1	1	1	10	10	4
yeps_YPTB3608	1	7	1	12	1	1	1	9	8	11	12	3	1	1	1	11	1	4	1	1	1	1	1	1	1	1	1	1	1	1	1	1	12	12	1
yeps_YPTB3609	3	1	3	1	1	1	3	1	1	1	1	3	1	1	1	1	1	3	3	3	1	1	1	3	1	1	1	1	1	1	1	1	1	1	3
yeps_YPTB3610	5	3	5	3	1	1	5	3	3	3	3	3	1	1	1	3	1	1	5	5	1	1	1	5	1	1	1	1	1	1	1	1	3	3	5
yeps_YPTB3611	4	42	4	11	1	1	4	9	8	11	11	3	1	1	1	11	1	5	4	4	1	1	1	4	1	1	1	1	1	1	1	1	11	11	4
yeps_YPTB3626	4	9	4	13	1	1	4	10	9	12	13	3	1	1	40	12	1	5	4	4	1	1	1	90	40	91	1	1	1	1	1	40	13	13	92
yeps_YPTB3628	1	2	1	5	1	1	1	3	2	4	5	2	1	1	1	4	1	2	1	1	1	1	1	1	1	1	1	1	1	1	1	1	5	5	1
yeps_YPTB3629	4	7	4	11	1	1	4	8	7	10	11	3	1	1	1	10	1	5	4	4	1	1	1	4	1	1	1	1	1	1	1	1	11	11	4
yeps_YPTB3631	4	7	4	12	1	1	4	10	7	12	12	3	1	1	1	12	1	4	4	4	1	1	1	4	1	1	1	1	1	1	1	1	12	12	4
yeps_YPTB3632	4	5	7	4	1	37	7	5	5	4	4	3	1	1	37	4	1	4	4	4	37	37	37	4	1	1	37	37	37	37	37	1	4	4	9
yeps_YPTB3633	4	7	4	1	1	8	4	9	7	1	1	3	1	1	8	1	1	4	4	4	8	8	8	4	1	1	8	8	8	8	8	1	1	1	4
yeps_YPTB3635	5	8	4	12	1	63	4	10	8	12	12	3	1	1	63	12	1	5	4	4	63	63	63	4	1	1	63	63	63	63	63	1	12	12	5
yeps_YPTB3636	19	5	2	7	1	1	2	1	5	1	7	1	1	1	1	1	1	2	2	2	1	1	1	2	1	1	1	1	1	1	1	1	7	7	2
yeps_YPTB3637	4	6	4	9	1	1	4	7	6	9	9	3	1	1	1	9	1	4	4	4	1	1	1	4	1	1	1	1	1	1	1	1	9	9	4
yeps_YPTB3638	3	5	3	8	1	1	3	7	5	8	8	1	1	1	1	8	1	3	3	3	1	1	1	3	1	1	1	1	1	1	1	1	8	8	3
yeps_YPTB3639	4	1	4	8	1	1	4	7	1	8	8	3	1	1	1	8	1	4	4	4	1	1	1	4	1	1	1	1	1	1	1	1	8	8	4
yeps_YPTB3642	2	4	2	2	1	1	2	6	4	2	2	1	1	1	1	2	1	2	2	2	1	1	1	2	1	1	1	1	1	1	1	1	2	2	2
yeps_YPTB3643	3	2	3	3	1	1	3	6	2	3	3	1	1	1	1	3	1	3	3	3	1	1	1	3	1	1	1	1	1	1	1	1	3	3	3
yeps_YPTB3645	4	6	4	9	1	1	4	8	6	9	9	3	1	1	1	9	1	4	4	4	1	1	1	4	1	1	1	1	1	1	1	1	9	9	4
yeps_YPTB3646	5	9	5	11	1	1	5	3	9	11	11	3	1	1	1	11	7	5	5	5	1	1	1	5	1	1	1	1	1	1	1	1	11	11	5
yeps_YPTB3647	3	2	3	2	1	1	3	2	2	2	2	2	1	1	1	2	1	3	3	3	1	1	1	3	1	1	1	1	1	1	1	1	2	2	3
yeps_YPTB3648	2	1	2	5	1	1	2	1	1	5	5	1	1	1	1	5	1	2	2	2	1	1	1	2	1	1	1	1	1	1	1	1	5	5	2
yeps_YPTB3650	1	1	1	5	1	1	1	1	1	5	5	3	1	6	1	5	6	4	1	1	1	1	1	1	1	1	1	1	1	1	1	1	5	5	1
yeps_YPTB3653	4	8	4	11	1	1	4	9	68	11	11	3	12	3	1	11	3	14	4	4	1	1	1	4	3	3	1	1	1	1	1	3	11	11	4
yeps_YPTB3654	1	1	1	3	1	1	1	1	1	3	3	1	1	1	1	3	1	3	1	1	1	1	1	1	1	1	1	1	1	1	1	1	3	3	1
yeps_YPTB3661	1	1	1	1	1	1	1	1	1	1	1	1	1	1	1	1	1	1	1	1	1	1	1	1	1	1	1	1	1	1	1	1	1	1	1
yeps_YPTB3662	2	3	2	1	1	1	2	2	3	1	1	2	1	1	1	1	1	1	2	2	1	1	1	2	1	1	1	1	1	1	1	1	1	1	2
yeps_YPTB3666	1	1	1	1	1	1	1	1	1	1	1	1	1	1	1	1	1	1	1	1	1	1	1	1	1	1	1	1	1	1	1	1	1	1	1
yeps_YPTB3669	1	1	1	1	1	1	1	1	1	1	1	1	1	1	1	1	1	1	1	1	1	1	1	1	1	1	1	1	1	1	1	1	1	1	1
yeps_YPTB3670	2	2	2	2	2	2	2	2	2	2	2	2	2	2	2	2	2	2	2	2	2	2	2	2	2	2	2	2	2	2	2	2	2	2	2
yeps_YPTB3671	1	1	1	1	1	1	1	1	1	1	1	1	1	1	1	1	1	1	1	1	1	1	1	1	1	1	1	1	1	1	1	1	1	1	1
yeps_YPTB3672	1	1	1	1	1	1	1	1	1	1	1	1	1	1	1	1	1	1	1	1	1	1	1	1	1	1	1	1	1	1	1	1	1	1	1
yeps_YPTB3673	1	1	1	1	1	1	1	1	1	1	1	1	1	1	1	1	1	1	1	1	1	1	1	1	1	1	1	1	1	1	1	1	1	1	1
yeps_YPTB3703	1	2	1	1	1	1	1	3	2	1	1	1	1	1	1	1	1	1	1	1	1	1	1	1	1	1	1	1	1	1	1	1	1	1	1
yeps_YPTB3707	2	2	2	2	1	1	2	2	2	2	2	1	1	1	1	2	1	2	2	2	1	1	1	2	1	1	1	1	1	1	1	1	2	2	2
yeps_YPTB3708	1	3	1	4	1	1	1	1	3	4	4	1	1	1	1	4	1	1	1	1	1	1	1	1	1	1	1	1	1	1	1	1	4	4	1
yeps_YPTB3713	2	3	2	1	1	2	2	2	1	1	1	2	2	2	2	1	2	2	2	2	2	2	2	2	2	2	2	2	2	2	2	2	1	1	2
yeps_YPTB3715	1	3	1	4	1	1	1	1	3	4	4	1	1	1	1	4	1	1	1	1	1	1	1	1	1	1	1	1	1	1	1	1	4	4	1
yeps_YPTB3716	5	5	5	9	1	1	5	7	5	9	9	3	4	4	1	9	4	5	5	5	1	1	1	5	4	4	1	1	1	1	1	4	9	9	10
yeps_YPTB3719	2	2	2	2	1	1	2	6	2	2	2	1	1	1	1	2	1	2	2	2	1	1	1	2	1	1	1	1	1	1	1	1	2	2	2
yeps_YPTB3720	3	2	3	2	36	1	3	5	2	2	2	1	1	1	1	2	1	3	3	3	1	1	1	3	1	1	1	1	1	1	1	1	2	2	3
yeps_YPTB3721	2	3	2	2	1	1	2	5	3	2	2	3	3	3	1	2	3	2	2	2	1	1	1	2	3	3	1	1	1	1	1	3	2	2	2
yeps_YPTB3723	8	2	3	7	1	1	3	5	2	6	7	1	1	1	1	6	1	1	3	3	1	1	1	3	1	1	1	1	1	1	1	1	7	7	3
yeps_YPTB3724	4	8	4	10	1	4	4	6	8	10	10	3	4	4	4	10	4	1	4	4	4	4	4	4	4	4	4	4	4	4	4	4	10	10	4
yeps_YPTB3726	1	1	1	3	1	1	1	1	1	3	3	3	1	1	1	3	1	1	1	1	1	1	1	1	1	1	1	1	1	1	1	1	3	3	1
yeps_YPTB3727	1	1	1	1	1	1	1	1	1	1	1	1	1	1	1	1	1	1	1	1	1	1	1	1	1	1	1	1	1	1	1	1	1	1	1
yeps_YPTB3728	1	4	1	1	1	3	1	1	4	1	1	1	3	3	3	1	3	1	1	1	3	3	3	1	3	3	3	3	3	3	3	3	1	1	1
yeps_YPTB3735	1	6	1	8	1	3	1	1	6	8	8	1	3	3	3	8	3	4	1	1	3	3	3	1	3	3	3	3	3	3	3	3	8	8	1
yeps_YPTB3736	4	7	4	9	1	3	4	8	7	9	9	3	3	3	3	9	3	1	4	4	3	3	3	4	3	3	3	3	3	3	3	3	9	9	4
yeps_YPTB3737	3	8	3	10	1	4	3	9	8	10	10	3	4	4	4	10	4	1	3	3	4	4	4	3	4	4	4	4	4	4	4	4	10	10	3
yeps_YPTB3739	4	5	4	12	1	4	4	10	5	5	12	3	4	4	4	5	4	5	4	4	4	4	4	4	4	4	4	4	4	4	4	4	12	12	4
yeps_YPTB3740	1	8	1	11	1	4	1	3	8	10	11	3	4	4	4	10	4	106	1	1	4	4	4	1	4	4	4	4	4	4	4	4	11	11	1
yeps_YPTB3741	1	1	1	1	1	1	1	1	1	1	1	1	1	1	1	1	1	1	1	1	1	1	1	1	1	1	1	1	1	1	1	1	1	1	1
yeps_YPTB3742	1	5	1	7	1	1	1	6	5	7	7	1	1	1	1	7	1	3	1	1	1	1	1	1	1	1	1	1	1	1	1	1	7	7	1
yeps_YPTB3743	1	6	1	9	1	3	1	7	6	9	9	1	3	3	3	9	3	4	1	1	3	3	3	1	3	3	3	3	3	3	3	3	9	9	1
yeps_YPTB3744	1	1	1	3	1	3	1	1	1	6	3	3	3	3	3	6	3	4	1	1	3	3	3	1	3	3	3	3	3	3	3	3	3	3	1
yeps_YPTB3745	3	6	3	2	1	3	3	7	6	2	2	3	3	3	3	2	3	2	8	8	3	3	3	8	3	3	3	3	3	3	3	3	2	2	3
yeps_YPTB3747	3	2	3	4	1	1	3	5	2	4	4	1	1	1	1	4	1	3	3	3	1	1	1	3	1	1	1	1	1	1	1	1	4	4	3
yeps_YPTB3748	1	2	1	2	1	1	1	2	2	2	2	1	1	1	1	2	1	2	1	1	1	1	1	1	1	1	1	1	1	1	1	1	2	2	1
yeps_YPTB3754	3	5	3	5	1	1	3	1		5	5	1	1	1	1	5	1	4	3	3	1	1	1	3	1	1	1	1	1	1	1	1	5	5	3
yeps_YPTB3755	3	6	3	5	1	1	3	7		5	5	1	1	1	1	5	1	4	3	3	1	1	1	3	1	1	1	1	1	1	1	1	5	5	3
yeps_YPTB3757	1	2	1	1	1	1	1	1	2	1	1	1	1	1	1	1	1	1	1	1	1	1	1	1	1	1	1	1	1	1	1	1	1	1	1
yeps_YPTB3758	3	8	3	10	1	1	3	9	8	10	10	1	1	1	1	10	1	4	3	3	1	1	1	3	1	1	1	1	1	1	1	1	10	10	3
yeps_YPTB3759	1	4	1	5	1	1	1	1	4	1	5	1	1	1	1	1	1	1	1	1	1	1	1	1	1	1	1	1	1	1	1	1	5	5	1
yeps_YPTB3761	1	4	1	1	1	1	1	5	4	1	1	1	1	23	1	1	1	3	1	1	1	1	1	1	1	1	1	1	1	1	1	1	1	1	1
yeps_YPTB3765	3	4	3	4	1	1	3	4	4	4	4	1	1	1	1	4	1	4	3	3	1	1	1	3	1	1	1	1	1	1	1	1	4	4	3
yeps_YPTB3769	1	2	1	2	1	1	1	2	2	2	2	1	1	1	1	2	1	2	1	1	1	1	1	1	1	1	1	1	1	1	1	1	2	2	1
yeps_YPTB3770	1	2	1	1	1	1	1	1	2	1	1	1	1	1	1	1	1	1	1	1	1	1	1	1	1	1	1	1	1	1	1	1	1	1	1
yeps_YPTB3771	1	4	1	2	1	1	1	5	4	2	2	1	1	1	1	2	1	2	1	1	1	1	1	1	1	1	1	1	1	1	1	1	2	2	1
yeps_YPTB3772	1	2	1	3	1	1	1	3	2	3	3	1	1	1	1	3	1	3	1	1	1	1	1	1	1	1	1	1	1	1	1	1	3	3	1
yeps_YPTB3773	1	1	1	1	1	1	1	1	1	1	1	1	1	1	1	1	1	1	1	1	1	1	1	1	1	1	1	1	1	1	1	1	1	1	1
yeps_YPTB3774	1	93	1	6	1	1	1	2	2	6	6	1	1	1	1	6	1	2	1	1	1	1	1	1	1	1	1	1	1	1	1	1	6	6	1
yeps_YPTB3775	4	8	4	2	1	3	4	9	8	10	2	3	3	3	3	10	3	6	4	4	3	3	3	4	3	3	3	3	3	3	3	3	2	2	4
yeps_YPTB3776	1	10	1	14	1	4	1	12	10	14	14	3	4	4	4	14	4	8	1	1	4	4	4	1	4	4	4	4	4	4	4	4	14	14	1
yeps_YPTB3777	1	1	1	1	1	1	1	4	1	1	1	3	1	1	1	1	1	4	1	1	1	1	1	1	1	1	1	1	1	1	1	1	1	1	1
yeps_YPTB3778	1	2	1	2	1	2	1	2	2	2	2	1	2	2	2	2	2	3	1	1	2	2	2	1	2	2	2	2	2	2	2	2	2	2	1
yeps_YPTB3783	5	8	5	10	1	1	5	9	8	10	10	3	4	4	1	10	4	6	5	5	1	1	1	5	4	4	1	1	1	1	1	4	10	10	5
yeps_YPTB3784	1	7	1	9	1	1	1	8	7	9	9	3	3	3	1	9	3	6	1	1	1	1	1	1	3	3	1	1	1	1	1	3	9	9	1
yeps_YPTB3785	1	4	1	3	1	1	1	3	4	6	3	3	1	1	1	6	1	3	1	1	1	1	1	1	1	1	1	1	1	1	1	1	3	3	1
yeps_YPTB3788	1	9	1	12	1	1	1	10	9	12	12	1	1	1	1	12	1	6	1	1	1	1	1	1	1	1	1	1	1	1	1	1	12	12	1
yeps_YPTB3796	3	6	3	7	1	3	3	3	6	8	7	3	4	4	3	8	4	1	3	3	3	3	3	3	4	4	3	3	3	3	3	4	7	7	3
yeps_YPTB3797	1	7	8	12	1	1	1	9	7	11	12	3	4	4	1	11	4	5	1	1	1	1	1	1	4	4	1	1	1	1	1	4	12	12	1
yeps_YPTB3798	12	8	9	11	1	1	1	10	8	11	11	3	4	4	1	11	4	5	1	1	1	1	1	1	4	4	1	1	1	1	1	4	11	11	1
yeps_YPTB3799	1	2	1	4	1	1	1	4	2	4	4	3	4	4	1	4	4	5	1	1	1	1	1	1	4	4	1	1	1	1	1	4	4	4	1
yeps_YPTB3802	1	5	1	6	1	1	1	6	5	6	6	1	1	1	1	6	1	3	1	1	1	1	1	1	1	1	1	1	1	1	1	1	6	6	1
yeps_YPTB3803	1	1	1	5	1	1	1	1	1	5	5	1	3	3	1	5	3	2	1	1	1	1	1	1	3	3	1	1	1	1	1	3	5	5	1
yeps_YPTB3804	1	5	1	6	1	1	1	5	5	6	6	3	3	3	1	6	3	2	1	1	1	1	1	1	3	3	1	1	1	1	1	3	6	6	1
yeps_YPTB3805	1	7	1	12	1	1	1	9	7	11	12	3	4	4	1	11	4	5	1	1	1	1	1	1	4	4	1	1	1	1	1	4	12	12	1
yeps_YPTB3806	1	6	1	9	1	1	1	7	6	9	9	3	3	3	1	9	3	12	1	1	1	1	1	1	3	3	1	1	1	1	1	3	9	9	1
yeps_YPTB3812	1	3	1	4	1	1	1	3	3	5	4	3	3	3	1	5	3	4	1	1	1	1	1	1	3	3	1	1	1	1	1	3	4	4	1
yeps_YPTB3813	1	7	1	9	1	1	1	8	7	9	9	3	4	4	1	9	4	5	1	1	1	1	1	1	4	4	1	1	1	1	1	4	9	9	1
yeps_YPTB3814	1	1	1	10	1	1	1	7	1	10	10	1	3	3	1	10	3	11	1	1	1	1	1	1	3	3	1	1	1	1	1	3	10	10	1
yeps_YPTB3815	1	1	1	9	1	1	1	1	1	8	9	1	3	3	1	8	3	5	1	1	1	1	1	1	3	3	1	1	1	1	1	3	9	9	1
yeps_YPTB3818	1	8	1	5	1	1	1	10	8	5	5	3	4	4	1	5	4	5	1	1	1	1	1	1	4	4	1	1	1	1	1	4	5	5	1
yeps_YPTB3820	1	7	1	12	1	1	1	10	8	12	12	3	3	3	1	12	3	4	1	1	1	1	1	1	3	3	1	1	1	1	1	3	12	12	1
yeps_YPTB3821	1	7	1	10	1	1	1	8	7	10	10	3	4	4	1	10	4	5	1	1	1	1	1	1	4	4	1	1	1	1	1	4	10	10	1
yeps_YPTB3829	1	3	1	3	1	3	1	5	3	3	3	3	3	3	3	3	3	1	1	1	3	3	3	1	3	3	3	3	3	3	3	3	3	3	1
yeps_YPTB3830	2	5	2	2	1	3	2	2	5	2	2	2	3	3	3	2	3	2	2	2	3	3	3	2	3	3	3	3	3	3	3	3	2	2	2
yeps_YPTB3832	1	8	1	12	1	4	1	9	8	12	12	3	4	4	4	12	4	5	1	1	4	4	4	1	4	4	4	4	4	4	4	4	12	12	1
yeps_YPTB3834	4	5	4	8	1	3	4	7	5	8	8	3	3	3	3	8	3	4	4	4	3	3	3	4	3	3	3	3	3	3	3	3	8	8	6
yeps_YPTB3838	1	3	1	9	1	4	1	8	3	9	9	3	4	4	4	9	4	5	1	1	4	4	4	1	4	4	4	4	4	4	4	4	9	9	1
yeps_YPTB3839	1	7	1	4	1	3	1	8	7	4	4	1	3	3	3	4	3	4	1	1	3	3	3	1	3	3	3	3	3	3	3	3	4	4	1
yeps_YPTB3841	1	9	1	1	1	3	1	10	9	11	1	1	3	3	3	11	3	7	1	1	3	3	3	1	3	3	3	3	3	3	3	3	1	1	1

yeps_YPTB3842	4	8	4	8	1	1	4	5	8	9	8	3	4	4	1	9	4	5	4	4	1	1	1	4	4	4	1	1	1	1	1	4	8	8	4
yeps_YPTB3846	1	4	6	9	1	1	6	7	4	9	9	4	1	1	1	9	1	5	1	1	1	1	1	1	1	1	1	1	1	1	1	1	9	9	1
yeps_YPTB3848	1	2	1	4	1	1	1	1	2	4	4	2	1	1	1	4	1	3	1	1	1	1	1	1	1	1	1	1	1	1	1	1	4	4	1
yeps_YPTB3850	4	8	4	5	1	1	4	5	8	5	5	3	1	1	1	5	1	5	4	4	1	1	1	4	1	1	1	1	1	1	1	1	5	5	4
yeps_YPTB3856	4	3	4	8	1	1	4	4	3	8	8	3	1	1	1	8	1	5	4	4	1	1	1	4	1	1	1	1	1	1	1	1	8	8	4
yeps_YPTB3857	3	1	3	6	1	1	3	1	1	6	6	2	1	1	1	6	1	4	3	3	1	1	1	3	1	1	1	1	1	1	1	1	6	6	3
yeps_YPTB3858	3	6	3	7	1	1	3	3	6	7	7	3	1	1	1	7	1	1	3	3	1	1	1	3	1	1	1	1	1	1	1	1	7	7	3
yeps_YPTB3859	4	7	4	3	1	1	4	4	7	3	3	3	1	1	1	3	1	4	4	4	1	1	1	4	1	1	1	1	1	1	1	1	3	3	4
yeps_YPTB3888	3	7	3	10	1	1	3	9	7	10	10	1	1	1	1	10	1	4	3	3	1	1	1	3	1	1	1	1	1	1	1	1	10	10	3
yeps_YPTB3904	1	4	1	2	1	1	1	1	4	2	2	1	1	1	1	2	1	2	1	1	1	1	1	1	1	1	1	1	1	1	1	1	2	2	1
yeps_YPTB3905	1	2	1	1	1	1	1	1	2	1	1	1	1	1	1	1	1	1	1	1	1	1	1	1	1	1	1	1	1	1	1	1	1	1	1
yeps_YPTB3906	4	8	4	12	1	1	4	10	8	12	12	3	1	1	1	12	1	5	4	4	1	1	1	4	1	1	1	1	1	1	1	1	12	12	4
yeps_YPTB3910	3	6	3	4	1	1	3	7	6	4	4	1	1	1	1	4	1	4	3	3	1	1	1	3	1	1	1	1	1	1	1	1	4	4	3
yeps_YPTB3911	2	2	2	5	1	1	2	5	2	5	5	3	1	1	1	5	1	2	2	2	1	1	1	2	1	1	1	1	1	1	1	1	5	5	2
yeps_YPTB3912	3	6	3	3	1	1	3	5	6	3	3	3	1	1	1	3	1	3	3	3	1	1	1	3	1	1	1	1	1	1	1	1	3	3	3
yeps_YPTB3913	1	1	1	1	1	1	1	7	1	1	1	3	1	1	1	1	1	4	1	8	1	1	1	8	1	1	1	1	1	1	1	1	1	1	1
yeps_YPTB3919	4	8	4	11	1	1	4	10	9	11	11	3	1	1	1	11	1	5	4	4	1	1	1	4	1	1	1	1	1	1	1	1	11	11	4
yeps_YPTB3922	1	4	1	2	1	1	1	1	4	2	2	1	1	1	1	2	1	2	1	1	1	1	1	1	1	1	1	1	1	1	1	1	2	2	1
yeps_YPTB3925	1	2	1	2	1	1	1	3	2	2	2	1	1	1	1	2	1	4	1	1	1	1	1	1	1	1	1	1	1	1	1	1	2	2	1
yeps_YPTB3926	4	7	4	5	1	1	4	8	7	5	5	3	1	1	1	5	1	5	4	4	1	1	1	4	1	1	1	1	1	1	1	1	5	5	4
yeps_YPTB3929	1	1	1	3	1	1	1	6	1	3	3	2	1	1	1	3	1	3	1	1	1	1	1	1	1	1	1	1	1	1	1	1	3	3	1
yeps_YPTB3930	4	4	4	5	1	1	4	5	4	5	5	3	1	1	1	5	1	5	4	4	1	1	1	4	1	1	1	1	1	1	1	1	5	5	4
yeps_YPTB3931	2	3	2	5	1	1	2	4	3	5	5	1	1	1	1	5	1	1	2	2	1	1	1	2	1	1	1	1	1	1	1	1	5	5	2
yeps_YPTB3941	3	6	3	8	1	1	3	6	6	7	8	1	1	1	1	7	1	4	3	3	1	1	1	3	1	1	1	1	1	1	1	1	8	8	3
yeps_YPTB3943	1	7	1	9	1	1	1	1	7	9	9	1	1	1	1	9	1	4	1	1	1	1	1	1	1	1	1	1	1	1	1	1	9	9	1
yeps_YPTB3944	1	1	1	1	1	1	1	1	1	1	1	1	1	1	1	1	1	1	1	1	1	1	1	1	1	1	1	1	1	1	1	1	1	1	1
yeps_YPTB3948	1	3	1	6	1	1	1	53	54	6	6	1	1	1	1	6	1	2	1	1	1	1	1	1	1	1	1	1	1	1	1	1	6	6	1
yeps_YPTB3949	3	3	3	7	1	1	3	8	3	7	7	1	1	1	1	7	1	4	3	3	1	1	1	3	1	1	1	1	1	1	1	1	7	7	3
yeps_YPTB3967	3	7	3	7	1	1	3	7	7	7	7	1	1	1	1	7	1	4	3	3	1	1	1	3	1	1	1	1	1	1	1	1	7	7	3
yeps_YPTB3968	2	1	2	1	1	1	2	1	1	1	1	1	1	1	1	1	1	1	2	2	1	1	1	2	1	1	1	1	1	1	1	1	1	1	2
yeps_YPTB3972	1	1	1	1	1	1	1	1	1	1	1	1	1	1	1	1	1	1	1	1	1	1	1	1	1	1	1	1	1	1	1	1	1	1	1
x_1	118	118	118	113	1	1	118	116	118	113	113	113	118	118	1	113	118	115	118	118	1	1	1	118	118	118	1	1	1	1	1	118	113	113	118
x_2	1	1	1	1	1	1	1	1	1	1	1	1	1	1	1	1	1	1	1	1	1	1	1	1	1	1	1	1	1	1	1	1	1	1	1
x_3	1	1	1	1	1	1	1	1	1	1	1	1	1	1	1	1	1	1	1	1	1	1	1	1	1	1	1	1	1	1	1	1	1	1	1
x_4	1	1	1	1	1	1	1	1	1	1	1	1	1	1	1	1	1	1	1	1	1	1	1	1	1	1	1	1	1	1	1	1	1	1	1
x_5	1	1	1	1	1	1	1	1	1	1	1	1	1	1	1	1	1	1	1	1	1	1	1	1	1	1	1	1	1	1	1	1	1	1	1
x_6	69	1	69	1	1	1	69	1	1	1	1	1	1	1	1	1	1	70	69	69	1	1	1	69	1	1	1	1	1	1	1	1	1	1	69
yeps_x_1	2	1	2	3	1	1	2	1	1	3	3	1	2	2	1	3	2	1	2	2	1	1	1	2	2	2	1	1	1	1	1	2	3	3	2
yeps_x_10	1	1	1	1	1	1	1	1	1	1	1	1	1	1	1	1	1	1	1	1	1	1	1	1	1	1	1	1	1	1	1	1	1	1	1
yeps_x_11	1	1	1	1	1	1	1	1	1	1	1	1	1	1	1	1	1	1	1	1	1	1	1	1	1	1	1	1	1	1	1	1	1	1	1
yeps_x_12	4	28	4	8	1	4	4	4	6	8	8	3	1	1	4	8	1	4	4	4	4	4	4	4	1	1	4	4	4	4	4	1	8	8	4
yeps_x_13	1	1	1	3	1	1	1	1	1	2	3	1	1	1	1	2	1	2	1	1	1	1	1	1	1	1	1	1	1	1	1	1	3	3	1
yeps_x_14	1	1	1	1	1	1	1	1	1	1	1	1	1	1	1	1	1	2	1	1	1	1	1	1	1	1	1	1	1	1	1	1	1	1	1
yeps_x_15	1		1	1	1	1	1	1	1	1	1	1	1	1	1	1	1	1	1	1	1	1	1	1	1	1	1	1	1	1	1	1	1	1	1
yeps_x_16	1	2	1	17	1	1	1	1	2	1	1	1	1	1	1	1	1	1	1	1	1	1	1	1	1	1	1	1	1	1	1	1	1	1	1
yeps_x_17	1	2	1	1	1	1	1	1	2	1	1	1	1	1	1	1	1	1	1	1	1	1	1	1	1	1	1	1	1	1	1	1	1	1	1
yeps_x_18	1	1	1	4	1	1	1	1	1	4	4	3	1	1	1	4	1	1	1	1	1	1	1	1	1	1	1	1	1	1	1	1	4	4	1
yeps_x_19	1	4	1	1	1	1	1	2	2	1	1	2	1	1	1	1	1	1	1	1	1	1	1	1	1	1	1	1	1	1	1	1	1	1	1
yeps_x_2	2	1	2	3	1	1	2	1	1	3	3	1	2	2	1	3	2	2	2	2	1	1	1	2	2	2	1	1	1	1	1	2	3	3	2
yeps_x_20	1	1	1	1	1	1	1	1	1	1	1	2	1	1	1	1	1	1	1	1	1	1	1	1	1	1	1	1	1	1	1	1	1	1	1
yeps_x_21	1	1	1	1	1	1	1	1	1	1	1	1	1	1	1	1	1	1	1	1	1	1	1	1	1	1	1	1	1	1	1	1	1	1	1
yeps_x_22	1	1	1	1	1	1	1	2	1	1	1	1	1	1	1	1	1	1	1	1	1	1	1	1	1	1	1	1	1	1	1	1	1	1	1
yeps_x_23	1	1	1	1	1	1	1	1	1	1	1	1	1	1	1	1	1	1	1	1	1	1	1	1	1	1	1	1	1	1	1	1	1	1	1
yeps_x_24	1	2	1	1	1	1	1	1	2	1	1	1	1	1	1	1	1	1	1	1	1	1	1	1	1	1	1	1	1	1	1	1	1	1	1
yeps_x_25	1	1	1	1	1	1	1	1	1	1	1	1	1	1	1	1	1	1	1	1	1	1	1	1	1	1	1	1	1	1	1	1	1	1	1
yeps_x_26	1	3	1	2	1	1	1	4	3	2	2	1	1	1	1	2	1	1	1	1	1	1	1	1	1	1	1	1	1	1	1	1	2	2	1
yeps_x_27	1	1	1	1	1	1	1	1	1	1	1	1	1	1	1	1	1	1	1	1	1	1	1	1	1	1	1	1	1	1	1	1	1	1	1
yeps_x_28	3	1	3	1	1	1	3	1	1	4	1	2	1	1	1	4	1	1	3	3	1	1	1	3	1	1	1	1	1	1	1	1	1	1	3
yeps_x_29	1	1	1	1	1	1	1	1	1	1	1	1	1	1	1	1	1	1	1	1	1	1	1	1	1	1	1	1	1	1	1	1	1	1	1
yeps_x_3	1	4	1	4	1	1	1	1	4	4	4	1	1	1	1	4	1	3	1	1	1	1	1	1	1	1	1	1	1	1	1	1	4	4	1
yeps_x_30	1	1	1	1	1	1	1	2	1	1	1	1	1	1	1	1	1	1	1	1				1	1	1						1	1	1	1
yeps_x_31	1	1	1	1	1	1	1	1	1	1	1	1	1	1	1	1	1	1	1	1	1	1	1	1	1	1	1	1	1	1	1	1	1	1	1
yeps_x_32	1	1	1	1	1	1	1	1	1	1	1	1	1	1	1	1	1	1	1	1	1	1	1	1	1	1	1	1	1	1	1	1	1	1	1
yeps_x_33	3	1	1	2	1	1	1	2	1	2	2	1	1	1	1	2	1	2	1	1	1	1	1	1	1	1	1	1	1	1	1	1	2	2	1
yeps_x_34	2	2	2	2	1	1	2	2	2	2	2	2	1	1	1	2	1	2	2	2	1	1	1	2	1	1	1	1	1	1	1	1	2	2	2
yeps_x_35	1	1	1	1	1	1	1	1	1	1	1	1	1	1	1	1	1	1	1	1	1	1	1	1	1	1	1	1	1	1	1	1	1	1	1
yeps_x_36	1	1	1	1	1	2	1	2	1	1	1	1	2	2	2	1	2	1	1	1	2	2	2	1	2	2	2	2	2	2	2	2	1	1	1
yeps_x_37	1	1	1	1	1	1	1	1	1	1	1	1	1	1	1	1	1	1	1	1	1	1	1	1	1	1	1	1	1	1	1	1	1	1	1
yeps_x_38	1	1	1	1	1	1	1	1	1	1	1	1	1	1	1	1	1	1	1	1	1	1	1	1	1	1	1	1	1	1	1	1	1	1	1
yeps_x_39	1	1	1	1	1	1	1	3	1	1	1	1	1	1	1	1	1	3	1	1	1	1	1	1	1	1	1	1	1	1	1	1	1	1	1
yeps_x_4	1	1	1	1	1	1	1	1	1	1	1	1	1	1	1	1	1	1	1	1	1	1	1	1	1	1	1	1	1	1	1	1	1	1	1
yeps_x_40	1	1	1	2	1	1	1	1	1	2	2	1	1	1	1	2	1	1	1	1	1	1	1	1	1	1	1	1	1	1	1	1	2	2	1
yeps_x_41	1	1	1	1	1	1	1	1	1	1	1	1	1	1	1	1	1	1	1	1	1	1	1	1	1	1	1	1	1	1	1	1	1	1	1
yeps_x_42	1	2	1	3	1	1	1	5	2	3	3	1	1	1	1	3	1	3	1	1	1	1	1	1	1	1	1	1	1	1	1	1	3	3	1
yeps_x_43	1	1	1	1	1	1	1	2	1	1	1	1	1	1	1	1	1	1	1	1	1	1	1	1	1	1	1	1	1	1	1	1	1	1	1
yeps_x_44	1	1	1	1	1	1	1	2	1	1	1	1	1	1	1	1	1	3	1	1	1	1	1	1	1	1	1	18	1	14	1	1	1	1	1
yeps_x_45	1	6	1	6	1	1	1	6	6	6	6	3	3	3	1	6	3	4	1	1	1	1	1	1	3	3	1	1	1	1	1	3	6	6	1
yeps_x_46	2	1	2	2	1	1	2	2	1	2	2	2	2	2	1	2	2	2	2	2	1	1	1	2	2	2	1	1	1	1	1	2	2	2	2
yeps_x_47	1	1	1	3	1	1	1	1	1	3	3	1	1	1	1	3	1	2	1	1	1	1	1	1	1	1	1	1	1	1	1	1	3	3	1
yeps_x_48	2	3	2	1	1	1	2	1	3	1	1	2	2	2	1	1	2	1	2	2	1	1	1	2	2	2	1	1	1	1	1	2	1	1	2
yeps_x_49	1	1	1	1	1	1	1	1	1	1	1	1	1	1	1	1	1	1	1	1	1	1	1	1	1	1	1	1	1	1	1	1	1	1	1
yeps_x_5	1	1	1	1	1	1	1	1	1	3	1	1	1	1	1	3	1	1	1	1	1	1	1	1	1	1	1	1	1	1	1	1	1	1	1
yeps_x_50	1	1	1	1	1	1	1	3	1	3	1	1	1	1	1	3	1	2	1	1	1	1	1	1	1	1	1	1	1	1	1	1	1	1	1
yeps_x_51	2	1	2	1	1	1	2	2	1	1	1	1	1	1	1	1	1	1	2	2	1	1	1	2	1	1	1	1	1	1	1	1	1	1	2
yeps_x_52	1	2	1	1	1	22	1	3	2	1	1	1	2	2	22	1	2	1	1	1	22	22	22	1	2	2	22	22	22	22	22	2	1	1	1
yeps_x_53	1	3	1	1	1	1	1	1	1	1	1	1	1	1	1	1	1	1	1	1	1	1	1	1	1	1	1	1	1	1	1	1	1	1	1
yeps_x_54	1	1	1	1	1	1	1	1	1	1	1	1	1	1	1	1	1	1	1	1	1	1	1	1	1	1	1	1	1	1	1	1	1	1	1
yeps_x_55	1	1	1	1	1	1	1	1	1	1	1	2	1	1	1	1	1	1	1	1	1	1	1	1	1	1	1	1	1	1	1	1	1	1	1
yeps_x_56	1	1	1	1	1	1	1	1	1	1	1	1	1	1	1	1	1	1	1	1	1	1	1	1	1	1	1	1	1	1	1	1	1	1	1
yeps_x_57	1	1	1	1	1	1	1	1	1	1	1	1	1	1	1	1	1	1	1	1	1	1	1	1	1	1	1	1	1	1	1	1	1	1	1
yeps_x_58	1	1	1	1	1	1	1	1	1	1	1	1	1	1	1	1	1	1	1	1	1	1	1	1	1	1	1	1	1	1	1	1	1	1	1
yeps_x_59	1	1	1	2	1	2	1	1	1	2	2	2	2	2	2	2	2	2	1	1	2	2	2	1	2	2	2	2	2	2	2	2	2	2	1
yeps_x_6	1	1	1	1	1	1	1	1	1	1	1	1	1	1	1	1	1	1	1	1	1	1	1	1	1	1	1	1	1	1	1	1	1	1	1
yeps_x_60	1	1	1	1	1	1	1	1	1	1	1	1	1	1	1	1	1	1	1	1	1	1	1	1	1	1	1	1	1	1	1	1	1	1	1
yeps_x_61	1	1	1	1	1	1	1		1	1	1	1	1	1	1	1	1	1	1	1	1	1	1	1	1	1	1	1	1	1	1	1	1	1	1
yeps_x_62	4	8	4	3	1	1	4	10	8	3	3	3	1	1	1	3	1	5	4	4	1	1	1	4	1	1	1	1	1	1	1	1	3	3	4
yeps_x_63	1	1	1	1	1	1	1	1	1	1	1	1	1	1	1	1	1	1	1	1	1	1	1	1	1	1	1	1	1	1	1	1	1	1	1
yeps_x_64	11	9	11	15	1	1	11	8	9	14	15	3	1	1	1	14	1	7	4	4	1	1	1	4	1	1	1	1	1	1	1	1	15	15	11
yeps_x_65	1	1	1	2	1	1	1	1	1	2	2	1	1	1	1	2	1	2	1	1	1	1	1	1	1	1	1	1	1	1	1	1	2	2	1
yeps_x_66	1	1	1	1	1	1	1	1	1	1	1	1	1	1	1	1	1	1	1	1	1	1	1	1	1	1	1	1	1	1	1	1	1	1	1
yeps_x_7	1	1	1	1	1	1	1	1	1	1	1	1	1	1	1	1	1	1	1	1	1	1	1	1	1	1	1	1	1	1	1	1	1	1	1
yeps_x_8	1	2	1	1	1	1	1	1	2	1	1	1	1	1	1	1	1	1	1	1	1	1	1	1	1	1	1	1	1	1	1	1	1	1	1
yeps_x_9	1	1	1	1	1	1	1	1	1	1	1	1	1	1	1	1	1	2	1	1	1	1	1	1	1	1	1	1	1	1	1	1	1	1	1
YPTB0002	1	1	1	1	1	1	1	72	1	1	1	1	1	1	1	1	1	1	1	1	1	1	1	1	1	1	1	1	1	1	1	1	1	1	1
YPTB0003	1	1	1	118	1	1	1	1	1	1	118	119	1	1	1	1	1	91	1	1	1	1	1	1	1	1	1	1	1	1	1	1	118	118	1
YPTB0028	79	79	79	81	1	1	79	79	79	81	81	1	79	79	1	81	79	79	79	79	1	1	1	79	79	79	1	1	1	1	1	79	81	81	79
YPTB0033	132	135	132	180	1	1	132	136	135	137	180	1	132	132	1	137	132	132	132	132	1	1	1	132	132	132	1	1	1	1	1	132	180	180	132
YPTB0037	73	73	73	73	1	1	73	73	73	73	73	74	73	73	1	73	73	73	73	73	1	1	1	73	73	73	1	1	1	1	1	73	73	73	73
YPTB0044	70	70	70	70	1	1	70	70	70	70	70	70	1	1	1	70	1	70	70	70	1	1	1	70	1	1	1	1	1	1	1	1	70	70	70
YPTB0047	1	1	1	1	1	1	1	1	1	1	1	1	1	1	1	1	1	1	1	1	1	1	1	1	1	1	1	1	1	1	1	1	1	1	1
YPTB0055	1	1	1	1	1	1	1	1	1	1	1	1	1	1	1	1	1	1	1	1	1	1	1	1	1	1	1	1	1	1	1	1	1	1	1
YPTB0070	1	1	1	1	1	1	1	1	1	1	1	87	1	1	1	1	1	1	1	1	1	1	1	1	1	1	1	1	1	1	1	1	1	1	1
YPTB0086	1	126	1	1	1	1	1	126	126	1	1	1	1	1	1	1	1	1	1	1	1	1	1	1	1	1	1	1	1	1	1	1	1	1	1
YPTB0089	1	1	1	1	1	1	1	1	1	1	1	1	1	1	1	1	1	1	1	1	1	1	1	1	1	1	1	1	1	1	1	1	1	1	1
YPTB0097	1	110	1	1	1	1	1	110	110	1	1	108	1	1	1	1	1	1	1	1	1	1	1	1	1	1	1	1	1	1	1	1	1	1	1
YPTB0101	126	129	126	126	1	1	184	131	129	126	126	126	1	1	1	126	1	127	126	126	1	1	1	126	1	1	1	1	1	1	1	1	126	126	126
YPTB0104	1	39	1	1	1	1	1	39	39	1	1	1	1	1	1	1	1	1	1	1	1	1	1	1	1	1	1	1	1	1	1	1	1	1	1
YPTB0121	111	1	111	110	1	1	111	114	1	110	110	111	1	1	1	110	1	1	111	111	1	1	1	111	1	1	1	1	1	1	1	1	110	110	111
YPTB0132	1	1	1	1	1	1	1	1	1	1	1	1	1	1	1	1	1	1	1	1	1	1	1	1	1	1	1	1	1	1	1	1	1	1	1
YPTB0134	1	124	1	121	1	1	1	123	124	121	121	169	1	1	1	121	1	118	1	1	1	1	1	1	1	1	1	1	1	1	1	1	121	121	1
YPTB0137	127	131	127	132	1	1	127	134	131	132	132	127	127	127	1	132	127	1	127	127	1	1	1	127	127	127	1	1	1	1	1	127	132	132	127
YPTB0166	1	1	1	47	1	1	1	1	1	47	47	1	1	1	1	47	1	1	1	1	1	1	1	1	1	1	1	1	1	1	1	1	47	47	1
YPTB0167	83	1	83	1	1	83	83	1	1	1	1	83	1	1	83	1	1	83	83	83	83	83	83	83	1	1	83	83	83	83	83	1	1	1	83
YPTB0171	112	112	112	112	1	1	112	112	112	112	112	1	1	1	1	112	1	112	112	112	1	1	1	112	1	1	1	1	1	1	1	1	112	112	112
YPTB0177	1	112	1	110	1	1	1	114	112	110	110	1	1	1	1	110	1	1	1	1	1	1	1	1	1	1	1	1	1	1	1	1	110	110	1
YPTB0182	1	107	1	105	1	1	1	108	107	105	105	106	1	1	1	105	1	105	1	1	1	1	1	1	1	1	1	1	1	1	1	1	105	105	1
YPTB0190	85	89	85	90	1	1	85	88	89	90	90	1	1	1	1	90	1	86	85	85	1	1	1	85	1	1	1	1	1	1	1	1	90	90	85
YPTB0193	1	89	1	112	1	1	1	1	111	92	112	1	1	1	1	92	1	90	1	1	1	1	1	1	1	1	1	1	1	1	1	1	112	112	1
YPTB0219	1	1	1	60	1	1	1	1	1	60	60	1	1	1	1	60	1	1	1	1	1	1	1	1	1	1	1	1	1	1	1	1	60	60	1
YPTB0222	75	1	75	77	1	1	75	79	1	77	77	1	1	1	1	77	1	75	75	75	1	1	1	75	1	1	1	1	1	1	1	1	77	77	75
YPTB0224	75	75	75	78	1	1	75	80	75	78	78	1	1	1	1	78	1	75	75	75	1	1	1	75	1	1	1	1	1	1	1	1	78	78	75
YPTB0227	102	105	102	106	1	1	102	102	105	106	106	101	1	1	1	106	1	102	102	102	1	1	1	102	1	1	1	1	1	1	1	1	106	106	102
YPTB0228	114	117	114	119	1	1	114	118	117	119	119	1	1	1	1	119	1	114	114	114	1	1	1	114	1	1	1	1	1	1	1	1	119	119	114
YPTB0229	94	98	94	98	1	1	94	99	98	98	98	1	1	1	1	98	1	95	94	94	1	1	1	94	1	1	1	1	1	1	1	1	98	98	94
YPTB0240	1	1	1	1	1	1	1	1	1	1	1	1	1	1	1	1	1	1	1	1	1	1	1	1	1	1	1	1	1	1	1	1	1	1	1
YPTB0247	95	98	95	1	1	1	95	102	292	100	1	94	1	1	1	100	1	96	95	95	1	1	1	95	1	1	1	1	1	1	1	1	1	1	95
YPTB0250	1	1	1	1	1	1	1	1	1	1	1	1	1	1	1	1	1	1	1	1	1	1	1	1	1	1	1	1	1	1	1	1	1	1	1
YPTB0261	102	105	102	102	1	1	102	105	105	102	102	1	1	1	1	102	1	102	102	102	1	1	1	102	1	1	1	1	1	1	1	1	102	102	102
YPTB0262	101	101	101	101	1	1	101	101	101	101	101	1	1	1	1	101	1	1	101	101	1	1	1	101	1	1	1	1	1	1	1	1	101	101	101
YPTB0264	105	1	105	1	1	1	105	104	1	1	1	1	1	1	1	1	1	106	105	105	1	1	1	105	1	1	1	1	1	1	1	1	1	1	105
YPTB0265	83	83	83	1	1	1	83	83	83	1	1	1	1	1	1	1	1	83	83	83	1	1	1	83	1	1	1	1	1	1	1	1	1	1	83
YPTB0266	114	115	114	1	1	1	114	114	115	1	1	1	1	1	1	1	1	114	114	114	1	1	1	114	1	1	1	1	1	1	1	1	1	1	114
YPTB0267	135	491	135	135	1	1	135	133	138	139	135	134	1	1	1	139	1	136	135	135	1	1	1	135	1	1	1	1	1	1	1	1	135	135	135
YPTB0270	272	91	91	91	1	1	91	91	91	91	91	91	1	1	1	91	1	91	91	91	1	1	1	91	1	1	1	1	1	1	1	1	91	91	91
YPTB0277	1	1	1	1	1	1	1	1	1	1	1	1	1	1	1	1	1	1	1	1	1	1	1	1	1	1	1	1	1	1	1	1	1	1	1
YPTB0278	1	1	1	1	1	1	1	1	1	1	1	1	1	67	1	1	67	1	1	1	1	1	1	1	1	1	1	1	1	1	1	1	1	1	1
YPTB0279	1	1	1	1	1	1	1	1	1	1	1	1	1	1	1	1	1	1	1	1	1	1	1	1	1	1	1	1	1	1	1	1	1	1	1
YPTB0280	1	1	1	1	1	1	1	1	1	1	1	1	1	1	1	1	1	1	1	1	1	1	1	1	1	1	1	1	1	1	1	1	1	1	1
YPTB0282	38	38	38	38	1	1	38	1	38	38	38	1	1	1	1	38	1	38	38	38	1	1	1	38	1	1	1	1	1	1	1	1	38	38	38
YPTB0296	1	1	1	1	1	1	1	1	1	1	1	1	1	1	1	1	1	1	1	1	1	1	1	1	1	1	1	1	1	1	1	1	1	1	1
YPTB0337	127	1	1	1	1	1	1	1	1	1	1	1	1	1	1	1	1	1	1	1	1	1	1	1	1	1	1	1	1	1	1	1	1	1	1
YPTB0370	73	74	73	73	1	1	73	75	74	73	73	73	1	1	1	73	1	73	73	73	1	1	1	73	1	1	1	1	1	1	1	1	73	73	73
YPTB0373	1	1	1	1	1	1	1	1	1	1	1	53	1	1	1	1	1	1	1	1	1	1	1	1	1	1	1	1	1	1	1	1	1	1	1
YPTB0375	120	123	120	124	1	1	120	126	123	124	124	120	1	1	1	124	1	121	120	120	1	1	1	120	1	1	1	1	1	1	1	1	124	124	120
YPTB0376	121	123	121	122	1	1	121	126	123	122	122	120	1	1	1	122	1	122	121	121	1	1	1	121	1	1	1	1	1	1	1	1	122	122	121
YPTB0377	129	130	129	128	1	1	129	128	130	128	128	125	161	1	1	128	1	127	129	129	1	1	1	129	1	1	1	1	1	1	1	1	128	128	129
YPTB0402	103	106	103	107	1	1	103	109	106	107	107	1	1	1	1	107	1	104	103	103	1	1	1	103	1	1	1	1	1	1	1	1	107	107	103
YPTB0404	1	31	1	1	1	1	1	1	31	1	1	1	1	1	1	1	1	1	1	1	1	1	1	1	1	1	1	1	1	1	1	1	1	1	1
YPTB0408	1	75	1	1	1	1	1	78	75	1	1	1	1	1	1	1	1	75	1	1	1	1	1	1	1	1	1	1	1	1	1	1	1	1	1
YPTB0411	1	1	1	1	1	1	1	1	1	1	1	1	1	1	1	1	1	1	1	1	1	1	1	1	1	1	1	1	1	1	1	1	1	1	1
YPTB0412	83	86	83	1	1	1	83	83	86	1	1	1	1	1	1	1	1	84	83	83	1	1	1	83	1	1	1	1	1	1	1	1	1	1	83
YPTB0424	101	101	101	101	1	1	101	1	101	101	101	1	1	1	1	101	1	101	101	101	1	1	1	101	1	1	1	1	1	1	1	1	101	101	101
YPTB0425	1	1	1	1	1	1	1	1	1	1	1	1	1	1	1	1	1	1	1	1	1	1	1	1	1	1	1	1	1	1	1	1	1	1	1
YPTB0438	1	1	1	1	1	1	1	1	1	1	1	1	1	1	1	1	1	1	1	1	1	1	1	1	1	1	1	1	1	1	1	1	1	1	1
YPTB0439	1	1	1	1	1	1	1	1	1	1	1	1	1	1	1	1	1	1	1	1	1	1	1	1	1	1	1	1	1	1	1	1	1	1	1
YPTB0440	1	1	1	1	1	1	1	1	1	1	1	1	1	1	1	1	1	1	1	1	1	1	1	1	1	1	1	1	1	1	1	1	1	1	1

YPTB0441	54	54	54	54	1	1	54	54	54	54	54	1	1	1	1	54	1	54	54	54	1	1	1	54	1	1	1	1	1	1	1	1	54	54	54
YPTB0444	1	79	1	80	1	1	1	78	78	80	80	1	1	1	1	80	1	78	1	1	1	1	1	1	1	1	1	1	1	1	1	1	80	80	1
YPTB0450	1	115	1	120	1	1	1	118	115	1	120	112	118	118	1	1	118	1	1	1	1	1	1	1	118	118	1	1	1	1	1	118	120	120	1
YPTB0463	105	104	105	1	1	1	105	1	104	1	1	1	1	1	1	1	1	1	105	105	1	1	1	105	1	1	1	1	1	1	1	1	1	1	105
YPTB0464	1	1	1	1	1	1	1	1	1	1	1	1	1	1	1	1	1	1	1	1	1	1	1	1	1	1	1	1	1	1	1	1	1	1	1
YPTB0465	1	1	1	1	1	1	1	1	1	1	1	1	1	1	1	1	1	1	1	1	1	1	1	1	1	1	1	1	1	1	1	1	1	1	1
YPTB0470	119	118	119	119	1	1	119	118	118	119	119	1	1	1	1	119	1	119	119	119	1	1	1	119	1	1	1	1	1	1	1	1	119	119	119
YPTB0471	1	1	1	1	1	1	1	1	1	1	1	1	1	1	1	1	1	1	1	1	1	1	1	1	1	1	1	1	1	1	1	1	1	1	1
YPTB0472	1	47	1	47	1	1	1	1	47	47	47	1	1	1	1	47	1	47	1	1	1	1	1	1	1	1	1	1	1	1	1	1	47	47	1
YPTB0473	40	1	40	41	1	1	40	40	1	41	41	1	1	1	1	41	1	41	40	40	1	1	1	40	1	1	1	1	1	1	1	1	41	41	40
YPTB0475	89	1	89	1	1	1	89	91	1	1	1	1	1	1	1	1	1	90	89	89	1	1	1	89	1	1	1	1	1	1	1	1	1	1	89
YPTB0476	102	103	102	101	1	1	102	100	103	101	101	101	1	1	1	101	1	101	102	102	1	1	1	102	1	1	1	1	1	1	1	1	101	101	102
YPTB0478	1	1	1	1	1	1	1	1	1	1	1	1	1	1	1	1	1	1	1	1	1	1	1	1	1	1	1	1	1	1	1	1	1	1	1
YPTB0481	1	1	1	1	1	1	1	1	1	1	1	1	1	1	1	1	1	1	1	1	1	1	1	1	1	1	1	1	1	1	1	1	1	1	1
YPTB0483	1	1	1	1	1	1	1	1	1	1	1	1	1	1	1	1	1	1	1	1	1	1	1	1	1	1	1	1	1	1	1	1	1	1	1
YPTB0485	1	1	1	1	1	1	1	1	1	1	1	1	1	1	1	1	1	1	1	1	1	1	1	1	1	1	1	1	1	1	1	1	1	1	1
YPTB0494	105	1	105	105	1	1	105	1	1	105	105	105	1	1	1	105	1	105	105	105	1	1	1	105	1	1	1	1	1	1	1	1	105	105	105
YPTB0523	120	119	120	119	1	1	120	124	119	119	119	1	1	1	1	119	1	121	358	120	1	1	1	120	1	1	1	1	1	1	1	1	119	119	120
YPTB0530	1	66	1	68	1	1	1	1	66	68	68	1	1	1	1	68	1	1	1	1	1	1	1	1	1	1	1	1	1	1	1	1	68	68	1
YPTB0531	1	115	1	1	1	1	1	116	115	1	1	1	1	1	1	1	1	1	1	1	1	1	1	1	1	1	1	1	1	1	1	1	1	1	1
YPTB0532	1	108	1	1	1	1	1	108	108	1	1	108	108	108	1	1	108	108	1	1	1	1	1	1	108	108	1	1	1	1	1	108	1	1	1
YPTB0548	1	104	1	105	1	1	1	1	104	105	105	103	1	1	1	105	1	1	1	1	1	1	1	1	1	1	1	1	1	1	1	1	105	105	1
YPTB0573	73	77	73	76	1	1	73	77	77	76	76	74	1	1	1	76	1	75	73	73	1	1	1	73	1	1	1	1	1	1	1	1	76	76	73
YPTB0574	80	79	80	1	1	1	80	79	79	1	1	79	1	1	1	1	1	81	80	80	1	1	1	80	1	1	1	1	1	1	1	1	1	1	80
YPTB0576	1	81	1	1	1	79	1	81	81	1	1	79	79	79	79	1	79	1	1	1	79	79	79	1	79	79	79	79	79	79	79	79	1	1	1
YPTB0577	1	1	1	30	1	1	1	1	1	30	30	1	1	1	1	30	1	29	1	1	1	1	1	1	1	1	1	1	1	1	1	1	30	30	1
YPTB0583	133	135	133	133	133	133	133	133	135	133	133	132	133	133	133	133	133	134	133	133	133	133	133	133	133	133	133	133	133	133	133	133	133	133	133
YPTB0598	1	1	1	108	1	1	1	1	1	82	108	1	1	1	1	82	1	1	1	1	1	1	1	1	1	1	1	1	1	1	1	1	108	108	1
YPTB0599	1	1	1	90	1	1	1	1	1	91	90	1	1	1	1	91	1	1	1	1	1	1	1	1	1	1	1	1	1	1	1	1	90	90	1
YPTB0603	97	98	97	99	1	1	97	100	98	99	99	96	1	1	1	99	1	97	97	97	1	1	1	97	1	1	1	1	1	1	1	1	99	99	97
YPTB0604	422	135	132	136	1	1	132	132	135	136	136	131	1	1	1	136	1	132	178	132	1	1	1	132	1	1	1	1	1	1	1	1	136	136	132
YPTB0616	93	94	93	94	1	1	93	1	94	94	94	1	1	1	1	94	1	93	93	93	1	1	1	93	1	1	1	1	1	1	1	1	94	94	93
YPTB0620	1	106	1	106	1	1	1	1	106	106	106	103	1	1	1	106	1	1	1	1	1	1	1	1	1	1	1	1	1	1	1	1	106	106	1
YPTB0622	152	117	118	117	1	1	118	117	154	117	117	117	1	1	1	117	1	117	118	118	1	1	1	118	1	1	1	1	1	1	1	1	117	117	118
YPTB0632	1	1	1	1	1	1	1	1	1	1	1	1	1	1	1	1	1	54	1	1	1	1	1	1	1	1	1	1	1	1	1	1	1	1	1
YPTB0633	86	85	86	1	1	1	86	1	85	1	1	1	1	1	1	1	1	1	86	86	1	1	1	86	1	1	1	1	1	1	1	1	1	1	86
YPTB0671	124	128	124	129	1	1	124	131	128	129	129	123	1	1	1	129	1	125	124	124	1	1	1	124	1	1	1	1	1	1	1	1	129	129	124
YPTB0685	1	104	1	105	1	1	1	101	104	105	105	101	1	1	1	105	1	102	1	1	1	1	1	1	1	1	1	1	1	1	1	1	105	105	1
YPTB0690	107	106	107	107	1	1	107	107	106	107	107	106	1	1	1	107	1	106	107	107	1	1	1	107	1	1	1	1	1	1	1	1	107	107	107
YPTB0692	1	1	1	1	1	1	1	1	1	1	1	1	1	1	1	1	1	1	1	1	1	1	1	1	1	1	1	1	1	1	1	1	1	1	1
YPTB0693	92	1	92	92	1	1	92	92	1	92	92	92	1	1	1	92	1	92	92	92	1	1	1	92	1	1	1	1	1	1	1	1	92	92	92
YPTB0701	81	81	81	81	1	1	81	81	81	81	81	81	1	1	1	81	1	81	81	81	1	1	1	81	1	1	1	1	1	1	1	1	81	81	81
YPTB0703	102	103	102	102	1	1	102	102	103	102	102	102	1	1	1	102	1	102	102	102	1	1	1	102	1	1	1	1	1	1	1	1	102	102	102
YPTB0712	1	1	1	1	1	1	1	1	1	1	1	1	1	1	1	1	1	1	1	1	1	1	1	1	1	1	1	1	1	1	1	1	1	1	1
YPTB0713	133	137	133	139	1	1	133	141	137	139	139	132	1	1	1	139	1	134	133	133	1	1	1	133	1	1	1	1	1	1	1	1	139	139	133
YPTB0719	91	89	91	93	1	1	91	89	89	93	93	90	1	1	1	93	1	92	91	91	1	1	1	91	1	1	1	1	1	1	1	1	93	93	91
YPTB0723	1	87	1	1	1	1	1	1	87	1	1	1	1	1	1	1	1	1	1	1	1	1	1	1	1	1	1	1	1	1	1	1	1	1	1
YPTB0726	96	97	96	99	1	1	96	97	97	99	99	95	1	1	1	99	1	97	96	96	1	1	1	96	1	1	1	1	1	1	1	1	99	99	96
YPTB0731	71	73	71	72	1	1	71	72	73	72	72	72	1	1	1	72	1	71	71	71	1	1	1	71	1	1	1	1	1	1	1	1	72	72	71
YPTB0734	1	1	1	1	1	1	1	1	1	1	1	1	1	1	1	1	1	1	1	1	1	1	1	1	1	1	1	1	1	1	1	1	1	1	1
YPTB0744	1	1	1	1	1	1	1	1	1	1	1	1	1	1	1	1	1	1	1	1	1	1	1	1	1	1	1	1	1	1	1	1	1	1	1
YPTB0747	92	95	92	95	1	1	92	95	95	95	95	1	1	1	1	95	1	93	92	92	1	1	1	92	1	1	1	1	1	1	1	1	95	95	92
YPTB0751	180	137	134	139	1	1	134	140	137	139	139	133	1	1	1	139	1	134	134	134	1	1	1	134	1	1	1	1	1	1	1	1	139	139	134
YPTB0765	1	107	1	107	1	1	1	108	107	107	107	107	1	1	1	107	1	1	1	1	1	1	1	1	1	1	1	1	1	1	1	1	107	107	1
YPTB0768	1	1	1	1	1	1	1	67	1	1	1	1	1	1	1	1	1	1	1	1	1	1	1	1	1	1	1	1	1	1	1	1	1	1	1
YPTB0774	1	1	1	1	1	1	1	1	1	1	1	1	1	1	1	1	1	1	1	1	1	1	1	1	1	1	1	1	1	1	1	1	1	1	1
YPTB0833	113	1	113	117	1	1	113	118	1	117	117	1	1	1	1	117	1	114	113	113	1	1	1	113	1	1	1	1	1	1	1	1	117	117	113
YPTB0834	1	1	1	1	1	1	1	1	1	1	1	1	1	1	1	1	1	1	1	1	1	1	1	1	1	1	1	1	1	1	1	1	1	1	1
YPTB0835	1	61	1	61	1	1	1	59	61	61	61	1	1	1	1	61	1	59	1	1	1	1	1	1	1	1	1	1	1	1	1	1	61	61	1
YPTB0842	1	119	1	120	1	1	1	121	119	120	120	115	1	1	1	120	1	116	1	1	1	1	1	1	1	1	1	1	1	1	1	1	120	120	1
YPTB0851	1	135	189	136	1	1	1	138	135	136	136	132	132	132	1	136	132	183	1	1	1	1	1	1	132	132	1	1	1	1	1	132	136	136	1
YPTB0857	1	70	1	1	1	1	1	70	70	1	1	1	1	1	1	1	1	1	1	1	1	1	1	1	1	1	1	1	1	1	1	1	1	1	1
YPTB0858	1	1	1	77	1	1	1	77	1	77	77	1	1	1	1	77	1	1	1	1	1	1	1	1	1	1	1	1	1	1	1	1	77	77	1
YPTB0873	121	122	121		1	1	121	126	122	124	124	120	1	1	1	124	1	121	121	121	1	1	1	121	1	1	1	1	1	1	1	1	124	124	121
YPTB0876	1	100	120	101	1	1	120	1	100	101	101	1	1	1	1	101	1	99	1	1	1	1	1	1	1	1	1	1	1	1	1	1	101	101	1
YPTB0887	1	111	1	145	1	1	1	1	111	112	145	1	1	1	1	112	1	109	1	1	1	1	1	1	1	1	1	1	1	1	1	1	145	145	1
YPTB0889	91	1	91	91	1	1	91	91	1	91	91	1	1	1	1	91	1	91	91	91	1	1	1	91	1	1	1	1	1	1	1	1	91	91	91
YPTB0890	79	79	79	81	1	1	79	79	79	81	81	79	1	1	1	81	1	79	79	79	1	1	1	79	1	1	1	1	1	1	1	1	81	81	79
YPTB0892	108	107	108	110	1	1	108	107	107	110	110	108	139	1	1	110	1	107	108	108	1	1	1	108	1	1	1	1	1	1	1	140	110	110	108
YPTB0894	1	1	1	1	1	1	1	1	1	1	1	1	1	1	1	1	1	1	1	1	1	1	1	1	1	1	1	1	1	1	1	1	1	1	1
YPTB0903	1	1	1	1	1	1	1	1	1	1	1	1	1	1	1	1	1	1	1	1	1	1	1	1	1	1	1	1	1	1	1	1	1	1	1
YPTB0920	109	111	109	147	1	1	109	113	111	109	147	1	1	1	1	109	1	110	109	109	1	1	1	109	1	1	1	1	1	1	1	1	147	147	109
YPTB0925	67	67	67	69	1	1	67	82	67	69	69	67	1	1	1	69	1	67	67	67	1	1	1	67	1	1	1	1	1	1	1	1	69	69	67
YPTB0930	340	1	120	123	1	1	120	119	1	123	123	1	1	1	1	123	1	161	412	120	1	1	1	120	1	1	1	1	1	1	1	1	123	123	120
YPTB0933	1	1	1	1	1	1	1	1	1	1	1	1	1	1	1	1	1	1	1	1	1	1	1	1	150	1	1	1	1	1	1	1	1	1	1
YPTB0934	126	1	126	1	1	1	126	125	1	1	1	1	1	1	1	1	1	1	126	126	1	1	1	126	1	1	1	1	1	1	1	1	1	1	126
YPTB0936	1	1	1	1	1	1	1	1	1	1	1	1	1	1	1	1	1	1	1	1	1	1	1	1	1	1	1	1	1	1	1	1	1	1	1
YPTB0950	1	1	1	1	1	1	1	76	1	1	1	1	1	1	1	1	1	1	1	1	1	1	1	1	1	1	1	1	1	1	1	1	1	1	1
YPTB0951	1	126	1	125	1	1	1	128	126	125	125	125	1	1	1	125	1	1	1	1	1	1	1	1	1	1	1	1	1	1	1	1	125	125	159
YPTB0955	1	82	1	1	1	1	1	81	82	1	1	1	1	1	1	1	1	81	1	1	1	1	1	1	1	1	1	1	1	1	1	1	1	1	1
YPTB0958	1	93	1	93	1	1	1	1	263	93	93	91	1	1	1	93	1	92	1	1	1	1	1	1	1	1	1	1	1	1	1	1	93	93	1
YPTB0959	1	1	1	1	1	1	1	1	1	1	1	1	1	1	1	1	1	1	1	1	1	1	1	1	1	1	1	1	1	1	1	1	1	1	1
YPTB0960	100	100	100	102	1	99	100	100	100	102	102	99	99	99	99	102	99	100	100	100	99	99	99	100	99	99	99	99	99	99	99	99	102	102	100
YPTB0962	1	1	1	1	1	1	1	1	1	1	1	1	1	1	1	1	1	1	1	1	1	1	1	1	1	1	1	1	1	1	1	1	1	1	1
YPTB0963	129	131	129	130	1	1	129	1	131	130	130	1	1	1	1	130	1	130	129	129	1	1	1	129	1	1	1	1	1	1	1	1	130	130	129
YPTB0966	1	103	1	1	1	1	1	1	103	1	1	1	1	1	1	1	1	1	1	1	1	1	1	1	1	1	1	1	1	1	1	1	1	1	1
YPTB0970	1	1	1	1	1	1	1	1	1	1	1	1	1	1	1	1	1	1	1	1	1	1	1	1	1	1	1	1	1	1	1	1	1	1	1
YPTB0979	46	1	46	1	1	1	46	1	1	1	1	1	1	1	1	1	1	46	46	46	1	1	1	46	1	1	1	1	1	1	1	1	1	1	46
YPTB0981	1	1	1	1	1	1	1	1	1	1	1	1	1	1	1	1	1	1	1	1	1	1	1	1	1	1	1	1	1	1	1	1	1	1	1
YPTB0990	1	72	1	72	1	1	1	1	72	72	72	72	1	1	1	72	1	72	1	1	1	1	1	1	1	1	1	1	1	1	1	1	72	72	1
YPTB0993	48	1	48	48	1	1	48	1	1	48	48	48	1	1	1	48	1	48	48	48	1	1	1	48	1	1	1	1	1	1	1	1	48	48	48
YPTB0994	96	96	96	97	1	1	96	96	96	97	97	96	1	1	1	97	1	96	96	96	1	1	1	96	1	1	1	1	1	1	1	1	97	97	96
YPTB1025	1	1	1	1	1	1	1	95	1	1	1	1	1	1	1	1	1	1	1	1	1	1	1	1	1	1	1	1	1	1	1	1	1	1	1
YPTB1027	316	116	1	116	1	1	1	117	116	116	116	1	1	1	1	116	1	1	1	1	1	1	1	1	1	1	1	1	1	1	1	1	116	116	1
YPTB1029	95	98	95	95	1	1	95	101	98	99	95	1	1	1	1	99	1	95	95	95	1	1	1	95	1	1	1	1	1	1	1	1	95	95	95
YPTB1034	1	1	1	71	1	1	1	1	1	71	71	1	1	1	1	71	1	1	1	1	1	1	1	1	1	1	1	1	1	1	1	1	71	71	1
YPTB1091	1	99	1	98	1	1	1	96	99	98	98	96	96	96	1	98	96	1	1	1	1	1	1	1	96	96	1	1	1	1	1	96	98	98	1
YPTB1096	1	1	1	1	1	1	1	94	1	1	1	93	93	93	1	1	93	1	1	1	1	1	1	1	93	93	1	1	1	1	1	93	1	1	1
YPTB1097	126	129	126	131	1	1	126	133	129	131	131	125	1	1	1	131	1	126	126	126	1	1	1	126	1	1	1	1	1	1	1	1	131	131	126
YPTB1098	1	1	1	1	1	1	1	1	1	75	1	1	1	1	1	75	1	1	1	1	1	1	1	1	1	1	1	1	1	1	1	1	1	1	1
YPTB1103	1	126	1	128	1	1	1	130	126	128	128	123	123	123	1	128	123	124	1	1	1	1	1	1	123	123	1	1	1	1	1	123	128	128	1
YPTB1104	1	1		1	1	1	1	1	1	74	1	1	1	1	1	74	1	1	1	1	1	1	1	1	1	1	1	1	1	1	1	1	1	1	1
YPTB1105	1	1	1	1	1	1	1	1	1	1	1	1	1	1	1	1	1	1	1	1	1	1	1	1	1	1	1	1	1	1	1	1	1	1	1
YPTB1106	1	1	1	84	1	1	1	84	1	84	84	1	1	1	1	84	1	84	1	1	1	1	1	1	1	1	1	1	1	1	1	1	84	84	1
YPTB1110	1	1	1	89	1	1	1	89	1	89	89	89	89	89	1	89	89	1	1	1	1	1	1	1	89	89	1	1	1	1	1	89	89	89	1
YPTB1116	92	92	92	1	1	1	92	92	92	1	1	1	1	1	1	1	1	1	92	92	1	1	1	92	1	1	1	1	1	1	1	1	1	1	92
YPTB1119	1	92	1	118	1	1	1	90	92	1	118	1	1	1	1	1	1	90	1	1	1	1	1	1	1	1	1	1	1	1	1	1	118	118	1
YPTB1125	1	1	1	1	1	1	1	1	1	1	1	71	1	1	1	1	1	1	1	1	1	1	1	1	1	1	1	1	1	1	1	1	1	1	1
YPTB1135	1	1	1	1	1	1	1	1	1	1	1	1	1	1	1	1	1	1	1	1	1	1	1	1	1	1	1	1	1	1	1	1	1	1	1
YPTB1141	1	64	1	64	1	1	1	64	64	64	64	1	1	1	1	64	1	64	1	1	1	1	1	1	1	1	1	1	1	1	1	1	64	64	1
YPTB1144	1	1	1	46	1	1	1	46	1	46	46	47	1	1	1	46	1	1	1	1	1	1	1	1	1	1	1	1	1	1	1	1	46	46	1
YPTB1145	1	123	1	122	1	1	1	122	123	122	122	1	1	1	1	122	1	1	1	1	1	1	1	1	1	1	1	1	1	1	1	1	122	122	1
YPTB1146	1	1	1	1	1	1	1	1	1	1	1	1	1	1	1	1	1	1	1	1	1	1	1	1	1	1	1	1	1	1	1	1	1	1	1
YPTB1147	178	139	178	135	1	1	178	135	139	135	135	1	1	1	1	135	1	179	178	178	1	1	1	178	1	1	1	1	1	1	1	1	135	135	178
YPTB1149	108	110	108	107	1	1	108	112	110	107	107	107	1	1	1	107	1	109	108	108	1	1	1	108	1	1	1	1	1	1	1	1	107	107	108
YPTB1155	1	1	1	48	1	1	1	1	1	48	48	1	1	1	1	48	1	1	1	1	1	1	1	1	1	1	1	1	1	1	1	1	48	48	1
YPTB1157	64	65	64	64	1	64	64	64	65	64	64	64	64	64	64	64	64	64	64	64	64	64	64	64	64	64	64	64	64	64	64	64	64	64	64
YPTB1159	1	1	1	1	1	1	1	1	1	1	1	1	120	120	1	1	120	89	1	1	1	1	1	1	120	120	1	1	1	1	1	120	1	1	1
YPTB1166	100	1	100	1	1	1	100	100	1	1	1	100	1	1	1	1	1	1	100	100	1	1	1	100	1	1	1	1	1	1	1	1	1	1	100
YPTB1169	112	113	112	113	1	1	112	112	113	113	113	112	146	1	1	113	1	145	112	112	1	1	1	112	1	1	1	1	1	1	1	1	113	113	112
YPTB1172	104	1	104	107	1	1	104	103	1	107	107	103	1	1	1	107	1	1	104	104	1	1	1	104	1	1	1	1	1	1	1	1	107	107	104
YPTB1192	62	62	62	1	1	1	62	1	62	1	1	1	1	1	1	1	1	62	62	62	1	1	1	62	1	1	1	1	1	1	1	1	1	1	62
YPTB1209	150	110	150	110	1	1	150	113	110	110	152	111	114	114	1	110	114	1	150	150	1	1	1	150	151	151	1	1	1	1	1	151	110	110	150
YPTB1212	109	113	109	114	1	1	109	109	113	114	114	110	114	114	1	114	114	1	109	150	1	1	1	150	114	114	1	1	1	1	1	114	114	114	109
YPTB1253	1	113	1	115	1	1	1	111	113	115	115	112	1	1	1	115	1	1	1	1	1	1	1	1	1	1	1	1	1	1	1	1	115	115	1
YPTB1297	1	1	1	35	1	1	1	1	1	34	35	1	1	1	1	34	1	1	1	1	1	1	1	1	1	1	1	1	1	1	1	1	35	35	1
YPTB1309	1	1	1	1	1	1	1	1	1	1	1	1	1	1	1	1	1	1	1	1	1	1	1	1	1	1	1	1	1	1	1	1	1	1	1
YPTB1316	1	82	1	81	1	1	1	83	82	81	81	1	1	1	1	81	1	97	1	1	1	1	1	1	1	1	1	1	1	1	1	1	81	81	1
YPTB1330	356	119	1	121	1	1	1	114	119	121	121	115	1	1	1	121	1	116	1	1	1	1	1	1	1	1	1	1	1	1	1	1	121	121	1
YPTB1358	1	1	1	1	1	1	1	1	1	1	1	1	1	1	1	1	1	57	1	1	1	1	1	1	1	1	1	1	1	1	1	1	1	1	1
YPTB1361	105	105	105	105	1	1	105	105	105	105	105	1	1	1	1	105	1	106	105	105	1	1	1	105	1	1	1	1	1	1	1	1	105	105	105
YPTB1362	1	118	1	1	1	1	1	1	118	1	1	1	1	1	1	1	1	1	1	1	1	1	382	1	1	1	1	1	1	1	1	1	1	1	1
YPTB1376	94	96	94	1	1	1	94	99	94	1	1	1	1	1	1	1	1	94	94	94	1	1	1	94	1	1	1	1	1	1	1	1	1	1	94
YPTB1377	1	92	1	1	1	1	1	91	92	1	1	1	1	1	1	1	1	1	1	1	1	1	1	1	1	1	1	1	1	1	1	1	1	1	1
YPTB1386	1	134	1	136	1	1	1	138	134	136	136	1	1	1	1	136	1	131	1	1	1	1	1	1	1	1	1	1	1	1	1	1	136	136	1
YPTB1391	132	137	132	132	1	1	132	132	137	132	132	1	1	1	1	132	1	134	132	132	1	1	1	132	1	1	1	1	1	1	1	1	132	132	132
YPTB1394	1	1	1	1	1	1	1	1	1	1	1	1	1	1	1	1	1	1	1	1	1	1	1	1	1	1	1	1	1	1	1	1	1	1	1
YPTB1395	1	1	1	1	1	1	1	1	1	1	1	1	1	1	1	1	1	1	1	1	1	1	1	1	1	1	1	1	1	1	1	1	1	1	1
YPTB1397	127	130	127	131	1	1	127	1	130	131	131	126	1	1	1	131	1	1	127	127	1	1	1	127	1	1	1	1	1	1	1	1	131	131	127
YPTB1403	115	115	115	116	1	1	115	117	115	116	116	1	1	1	1	116	1	115	115	115	1	1	1	115	1	1	1	1	1	1	1	1	116	116	115
YPTB1408	1	1	1	125	167	1	1	126	1	125	125	1	1	1	1	125	1	123	1	1	1	1	1	1	1	1	1	1	1	1	1	1	125	125	1
YPTB1414	1	113	1	113	1	1	1	1	113	113	113	145	115	115	1	113	334	112	1	1	1	1	1	1	115	115	1	1	1	1	1	115	113	113	1
YPTB1416	1	1	1	68	1	1	1	1	1	68	68	1	68	68	1	68	68	1	1	1	1	1	1	1	68	68	1	1	1	1	1	68	68	68	1
YPTB1417	1	80	1	1	1	1	1	1	80	1	1	1	82	82	1	1	82	79	1	1	1	1	1	1	82	82	1	1	1	1	1	82	1	1	1
YPTB1428	1	112	1	1	1	1	1	1	112	1	1	1	1	1	1	1	1	112	1	1	1	1	1	1	1	1	1	1	1	1	1	1	1	1	1
YPTB1436	107	111	107	112	1	1	107	106	111	112	112	1	1	1	1	112	1	108	107	107	1	1	1	107	1	1	1	1	1	1	1	1	112	112	107
YPTB1439	1	104	1	100	1	1	1	100	104	100	100	100	100	100	1	100	100	103	1	1	1	1	1	1	100	100	1	1	1	1	1	100	100	100	1
YPTB1442	1	476	1	130	1	1	1	131	126	130	130	124	124	124	1	130	124	126	1	1	1	1	1	1	124	124	1	1	1	1	1	124	130	130	1
YPTB1445	1	1	1	1	1	1	1	1	1	1	1	1	1	1	1	1	1	1	1	1	1	1	1	1	1	1	1	1	1	1	1	1	1	1	1
YPTB1454	1	1	1	1	1	1	1	66	1	1	1	66	66	66	1	1	66	67	1	1	1	1	1	1	66	66	1	1	1	1	1	66	1	1	1
YPTB1524	101	102	101	108	1	1	101	104	102	101	108	1	1	1	1	101	1	101	126	126	1	1	1	126	1	1	1	1	1	1	1	1	108	108	101
YPTB1531	130	1	130		1	1	130	136	1	134		129	137	137	1	134	137	131	130	130	1	1	1	130	137	137	1	1	1	1	1	137			130
YPTB1535	126	1	126	1	1	1	126	126	1	1	1	1	1	1	1	1	1	127	126	126	1	1	1	126	1	1	1	1	1	1	1	1	1	1	126
YPTB1537	1	80	1	80	1	1	1	1	80	80	80	1	1	1	1	80	1	1	1	1	1	1	1	1	1	1	1	1	1	1	1	1	80	80	1
YPTB1555	80	81	80	80	1	1	80	1	81	80	80	1	1	1	1	80	1	78	80	80	1	1	1	80	1	1	1	1	1	1	1	1	80	80	80
YPTB1557	104	1	104	1	1	1	104	107	1	1	1	1	1	1	1	1	1	104	104	104	1	1	1	104	1	1	1	1	1	1	1	1	1	1	104
YPTB1558	1	1	1	1	1	1	1	1	1	1	1	1	1	1	1	1	1	1	1	1	1	1	1	1	114	1	1	1	1	1	1	1	1	1	1
YPTB1559	121	125	121	126	1	1	121	128	159	126	126	120	1	1	1	126	1	122	121	121	1	1	1	121	1	1	1	1	1	1	1	1	126	126	121
YPTB1615	62	1	62	1	1	1	62	1	1	1	1	1	1	1	1	1	1	1	62	62	1	1	1	62	1	1	1	1	1	1	1	1	1	1	62
YPTB1647	124	1	124	126	1	1	124	1	1	126	126	1	1	1	1	126	1	1	124	124	1	1	1	124	1	1	1	1	1	1	1	1	126	126	124
YPTB1724	123	1	123	124	1	1	123	122	1	126	124	1	1	1	1	126	1	124	123	123	1	1	1	123	1	1	1	1	1	1	1	1	124	124	123
YPTB1795	100	100	100	100	1	1	100	1	100	100	100	1	100	100	1	100	100	100	100	100	1	1	1	100	100	100	1	1	1	1	1	100	100	100	100
YPTB1921	1	1	1	1	1	1	1	1	1	1	1	1	1	1	1	1	1	1	1	1	1	1	1	1	1	1	1	1	1	1	1	1	1	1	1
YPTB1940	1	1	1	1	1	1	1	1	1	1	1	1	1	1	1	1	1	85	1	1	1	1	1	1	1	1	1	1	1	1	1	1	1	1	1
YPTB2001	1	1	1	65	1	1	1	68	1	65	65	1	1	1	1	65	1	66	1	1	1	1	1	1	1	1	1	1	1	1	1	1	65	65	1
YPTB2005	1	113	1	115	1	1	1	114	113	115	115	1	114	114	1	115	114	111	1	1	1	1	1	1	145	145	1	1	1	1	1	145	115	115	1

YPTB2007	1	1	1	1	1	1	1	56	1	1	1	1	1	1	1	1	1	1	1	1	1	1	1	1	1	1	1	1	1	1	1	1	1	1	1
YPTB2008	1	95	1	93	1	1	1	93	110	93	93	92	92	92	1	93	92	93	1	1	1	1	1	1	92	92	1	1	1	1	1	92	93	93	1
YPTB2009	1	100	1	98	1	1	1	101	100	98	98	1	1	1	1	98	1	121	1	1	1	1	1	1	1	1	1	1	1	1	1	1	98	98	1
YPTB2019	1	102	1	129	1	1	1	103	102	1	129	1	1	1	1	1	1	1	1	1	1	1	1	1	1	1	1	1	1	1	1	1	129	129	1
YPTB2023	1	1	1	1	1	1	1	1	1	1	1	1	1	1	1	1	1	1	1	1	1	1	1	1	1	1	1	1	1	1	1	1	1	1	1
YPTB2032	1	108	1	108	1	1	1	110	108	108	108	1	1	1	1	108	1	108	1	1	1	1	1	1	1	1	1	1	1	1	1	1	108	108	1
YPTB2038	1	103	1	105	1	1	1	103	103	105	105	1	1	1	1	105	1	1	1	1	1	1	1	1	1	1	1	1	1	1	1	1	105	105	1
YPTB2042	97	92	97	94	1	1	97	97	92	94	94	1	1	1	1	94	1	94	93	93	1	1	1	93	1	1	1	1	1	1	1	1	94	94	97
YPTB2045	1	1	1	156	1	1	1	124	1	121	156	1	1	1	1	121	1	121	1	1	1	1	1	1	395	1	1	1	1	1	1	1	156	156	1
YPTB2047	121	121	121	121	1	122	121	121	121	121	121	122	122	122	122	121	122	121	121	121	122	122	122	121	122	122	122	122	122	122	122	122	121	121	121
YPTB2053	1	75	1	75	1	1	1	1	75	75	75	1	1	1	1	75	1	1	1	1	1	1	1	1	1	1	1	1	1	1	1	1	75	75	1
YPTB2059	1	1	1	1	1	1	1	1	1	1	1	1	1	1	1	1	1	1	1	1	1	1	1	1	1	1	1	1	1	1	1	1	1	1	1
YPTB2060	1	1	1	1	1	1	1	1	1	1	1	1	1	1	1	1	1	1	1	1	1	1	1	1	1	1	1	1	1	1	1	1	1	1	1
YPTB2064	1	207	1	86	1	1	1	1	86	86	86	1	1	1	1	86	1	86	1	1	1	1	1	1	1	1	1	1	1	1	1	1	86	86	1
YPTB2083	106	88	85	1	1	1	85	1	88	1	1	1	1	1	1	1	1	1	85	85	1	1	1	85	1	1	1	1	1	1	1	1	1	1	85
YPTB2084	76	76	76	1	1	1	76	1	76	77	1	1	1	1	1	77	1	1	76	76	1	1	1	76	1	1	1	1	1	1	1	1	1	1	76
YPTB2089	98	1	98	97	1	1	98	1	1	97	97	97	1	1	1	97	1	99	98	98	1	1	1	98	1	1	1	1	1	1	1	1	97	97	98
YPTB2090	114	116	114	114	1	1	114	117	116	114	114	113	1	1	1	114	1	113	114	114	1	1	1	114	1	1	1	1	1	1	1	1	114	114	114
YPTB2116	1	1	1	1	1	1	1	1	1	1	1	1	1	1	1	1	1	1	1	1	1	1	1	1	1	1	1	1	1	1	1	1	1	1	1
YPTB2125	116	116	116	116	1	1	116	1	116	116	116	115	1	1	1	116	1	1	116	116	1	1	1	116	1	1	1	1	1	1	1	1	116	116	116
YPTB2126	1	118	1	119	1	1	1	1	118	119	119	118	1	1	1	119	1	1	1	1	1	1	1	1	1	1	1	1	1	1	1	1	119	119	1
YPTB2137	112	141	112	112	1	1	112	112	141	112	112	1	1	1	1	112	1	113	112	112	1	1	1	112	1	1	1	1	1	1	1	1	112	112	112
YPTB2139	51	1	51	1	1	1	51	1	1	1	1	1	1	1	1	1	1	1	51	51	1	1	1	51	1	1	1	1	1	1	1	1	1	1	51
YPTB2144	1	1	1	1	1	1	1	1	1	1	1	1	1	1	1	1	1	1	1	1	1	1	1	1	1	1	1	1	1	1	1	1	1	1	1
YPTB2146	1	1	1	1	1	1	1	55	1	1	1	1	1	1	1	1	1	1	1	1	1	1	1	1	1	1	1	1	1	1	1	1	1	1	1
YPTB2147	117	1	117	1	1	1	117	119	352	1	1	1	1	154	1	1	154	117	117	117	1	1	1	117	1	1	1	1	1	1	1	1	1	1	117
YPTB2156	130	134	170	174	129	129	170	138	134	136	174	129	129	129	129	136	129	131	130	130	129	129	129	130	129	129	129	129	129	129	129	129	174	174	130
YPTB2160	1	96	1	97	1	1	1	99	96	97	97	93	1	1	1	97	1	1	1	1	1	1	1	1	1	1	1	1	1	1	1	1	97	97	1
YPTB2167	1	1	1	1	1	1	1	1	1	1	1	1	1	1	1	1	1	1	1	1	1	1	1	1	1	1	1	1	1	1	1	1	1	1	1
YPTB2261	1	1	1	78	1	1	1	1	1	78	78	1	77	77	1	78	77	1	1	1	1	1	1	1	77	77	1	1	1	1	1	77	78	78	1
YPTB2263	1	86	1	85	1	1	1	87	86	85	85	84	84	84	1	85	84	1	1	1	1	1	1	1	84	84	1	1	1	1	1	84	85	85	1
YPTB2266	1	159	1	120	1	1	1	121	159	120	120	115	115	115	1	120	115	117	1	1	1	1	1	1	115	115	1	1	1	1	1	115	120	120	1
YPTB2273	106	109	106	106	1	1	106	106	109	106	106	107	1	1	1	106	1	141	106	106	1	1	1	106	1	1	1	1	1	1	1	1	106	106	106
YPTB2274	116	1	116	116	1	1	116	116	1	116	116	116	1	1	1	116	1	117	116	116	1	1	1	116	1	1	1	1	1	1	1	1	116	116	116
YPTB2275	1	1	1	100	1	1	1	1	1	99	100	98	1	1	1	99	1	1	1	1	1	1	1	1	1	1	1	1	1	1	1	1	100	100	1
YPTB2283	1	113	1	112	1	1	1	116	113	114	112	1	1	1	1	114	1	110	1	1	1	1	1	1	1	1	1	1	1	1	1	1	112	112	1
YPTB2312	104	107	104	1	1	1	104	1	107	1	1	1	1	1	1	1	1	105	104	104	1	1	1	104	1	1	1	1	1	1	1	1	1	1	290
YPTB2322	118	117	118	117	1	1	118	117	117	117	117	1	1	1	1	117	1	118	118	118	1	1	1	118	1	1	1	1	1	1	1	1	117	117	118
YPTB2323	1	1	1	75	1	1	1	1	1	75	75	1	1	1	1	75	1	1	1	1	1	1	1	1	1	1	1	1	1	1	1	1	75	75	1
YPTB2324	71	1	86	1	1	1	86	1	1	1	1	1	1	1	1	1	1	71	71	71	1	1	1	71	1	1	1	1	1	1	1	1	1	1	71
YPTB2336	131	134	131	139	1	1	131	137	134	136	139	1	1	176	1	136	176	177	131	131	1	1	1	131	1	1	1	1	1	1	1	1	139	139	131
YPTB2337	94	95	94	94	1	1	94	94	95	94	94	1	1	1	1	94	1	94	94	94	1	1	1	94	1	1	1	1	1	1	1	1	94	94	94
YPTB2339	1	1	1	1	1	1	1	1	1	1	1	1	1	1	1	1	1	1	1	1	1	1	1	1	1	1	1	1	1	1	1	1	1	1	1
YPTB2340	1	1	1	1	1	1	1	1	1	1	1	1	1	1	1	1	1	1	1	1	1	1	1	1	1	1	1	1	1	1	1	1	1	1	78
YPTB2341	1	1	1	1	1	1	1	1	1	1	1	1	1	1	1	1	1	1	1	1	1	1	1	1	1	1	1	1	1	1	1	1	1	1	1
YPTB2353	1	1	1	1	1	1	1	1	1	1	1	79	1	1	1	1	1	79	1	1	1	1	1	1	1	1	1	1	1	1	1	1	1	1	1
YPTB2354	1	1	1	87	1	1	1	87	1	87	87	87	1	1	1	87	1	87	1	1	1	1	1	1	1	1	1	1	1	1	1	1	87	87	1
YPTB2389	1	1	1	1	1	1	1	1	1	1	1	1	1	1	1	1	1	1	1	1	1	1	1	1	1	1	1	1	1	1	1	1	1	1	1
YPTB2396	1	81	1	98	1	1	1	1	81	1	98	1	1	1	1	1	1	1	1	1	1	1	1	1	1	1	1	1	1	1	1	1	98	98	1
YPTB2397	1	1	1	1	1	1	1	1	1	1	1	1	1	1	1	1	1	1	1	1	1	1	1	1	1	1	1	1	1	1	1	1	1	1	1
YPTB2427	167	119	167	117	1	1	167	168	119	117	117	114	170	1	1	117	1	125	167	167	1	1	1	167	1	1	1	1	1	1	1	170	117	117	167
YPTB2432	1	1	1	118	1	1	1	1	1	118	118	1	1	1	1	118	1	116	1	1	1	1	1	1	1	1	1	1	1	1	1	1	118	118	1
YPTB2442	111	114	111	116	1	111	111	118	141	116	116	110	1	1	111	116	1	111	111	111	111	111	111	111	1	1	111	111	111	111	111	1	116	116	111
YPTB2447	117	116	113	115	1	1	113	114	116	115	115	113	1	1	1	115	1	114	113	113	1	1	1	113	1	1	1	1	1	1	1	1	115	115	113
YPTB2448	85	86	85	85	1	1	85	86	86	85	85	85	1	1	1	85	1	86	85	85	1	1	1	85	1	1	1	1	1	1	1	1	85	85	85
YPTB2467	143	109	1	142	1	1	1	109	109	109	142	106	1	1	1	109	1	109	1	1	1	1	1	1	1	1	1	1	1	1	1	1	142	142	1
YPTB2470	1	1	1	1	1	1	1	1	1	1	1	18	1	1	1	1	1	1	1	1	1	1	1	1	1	1	1	1	1	1	1	1	1	1	1
YPTB2471	63	1	63	1	1	1	63	66	1	1	1	1	1	1	1	1	1	1	63	63	1	1	1	63	1	1	1	1	1	1	1	1	1	1	63
YPTB2474	86	88	86	94	1	1	86	92	88	90	94	1	1	1	1	90	1	87	86	86	1	1	1	86	1	1	1	1	1	1	1	1	94	94	86
YPTB2475	19	19	19	20	1	1	19	1	19	20	20	1	1	1	1	20	1	20	19	19	1	1	1	19	1	1	1	1	1	1	1	1	20	20	19
YPTB2549	1	79	100	77	1	1	1	1	79	77	77	1	82	82	1	77	82	77	1	1	1	1	1	1	82	82	1	1	1	1	1	82	77	77	1
YPTB2557	123	124	123	124	1	382	123	124	124	124	124	122	124	124	382	124	124	124	123	123	382	382	382	123	124	124	382	382	382	382	382	124	124	124	123
YPTB2558	100	102	100	103	1	100	100	105	102	103	103	99	99	99	100	103	99	1	100	100	100	100	100	100	99	99	100	100	100	100	100	99	103	103	100
YPTB2577	137	141	137	143	1	1	137	144	184	143	143	1	1	1	1	143	1	187	137	137	1	1	1	137	1	1	1	1	1	1	1	1	143	143	137
YPTB2578	1	1	1	1	1	1	1	1	1	1	1	1	1	1	1	1	1	1	1	1	1	1	1	1	1	1	1	1	1	1	1	1	1	1	1
YPTB2580	1	1	1	1	1	1	1	1	1	1	1	1	1	1	1	1	1	1	1	1	1	1	1	1	1	1	1	1	1	1	1	1	1	1	1
YPTB2581	97	100	97	101	1	1	97	102	100	101	101	1	1	1	1	101	1	97	97	97	1	1	1	97	1	1	1	1	1	1	1	1	101	101	97
YPTB2585	120	123	120	125	1	1	120	127	123	125	125	119	1	1	1	125	1	120	120	120	1	1	1	120	1	1	1	1	1	1	1	1	125	125	120
YPTB2594	1	63	1	1	1	1	1	63	63	1	1	63	1	1	1	1	1	1	1	1	1	1	1	1	1	1	1	1	1	1	1	1	1	1	1
YPTB2595	1	1	1	1	1	1	1	1	1	1	1	1	1	1	1	1	1	1	1	1	1	1	1	1	1	1	1	1	1	1	1	1	1	1	1
YPTB2597	92	95	92	1	1	1	92	96	95	1	1	93	1	1	1	1	1	1	92	92	1	1	1	92	1	1	1	1	1	1	1	1	1	1	92
YPTB2600	78	79	78	1	1	1	78	1	79	1	1	1	1	1	1	1	1	1	78	78	1	1	1	78	1	1	1	1	1	1	1	1	1	1	78
YPTB2601	1	69	1	70	1	1	1	1	69	70	70	1	1	1	1	70	1	1	1	1	1	1	1	1	1	1	1	1	1	1	1	1	70	70	1
YPTB2602	109	112	109	113	1	1	109	114	112	113	113	1	109	109	1	113	109	110	109	109	1	1	1	109	109	109	1	1	1	1	1	109	113	113	109
YPTB2609	1	87	1	1	1	1	1	86	87	85	1	1	1	1	1	85	1	1	1	1	1	1	1	1	1	1	1	1	1	1	1	1	1	1	1
YPTB2612	101	105	101	107	1	1	101	109	105	107	107	1	140	140	1	107	140	103	101	101	1	1	1	101	140	140	1	1	1	1	1	140	107	107	101
YPTB2613	1	1	1	1	1	1	1	1	1	1	1	1	1	1	1	1	1	1	1	1	1	1	1	1	1	1	1	1	1	1	1	1	1	1	1
YPTB2617	88	1	88	1	1	1	88	1	205	1	1	1	1	1	1	1	1	1	88	88	1	1	1	88	1	1	1	1	1	1	1	1	1	1	88
YPTB2630	109	1	109	1	1	1	109	1	1	1	1	1	1	1	1	1	1	1	109	109	1	1	1	109	1	1	1	1	1	1	1	1	1	1	109
YPTB2632	116	116	116	116	1	1	116	119	116	116	116	116	1	1	1	116	1	117	116	116	1	1	1	116	1	1	1	1	1	1	1	1	116	116	116
YPTB2637	1	1	1	1	1	1	1	1	1	1	1	106	1	1	1	1	1	1	1	1	1	1	1	1	1	1	1	1	1	1	1	1	1	1	1
YPTB2640	1	95	1	95	1	1	1	95	95	95	95	96	1	1	1	95	1	95	1	1	1	1	1	1	1	1	1	1	1	1	1	1	95	95	1
YPTB2714	1	98	1	96	1	1	1	97	98	96	96	1	1	1	1	96	1	97	1	1	1	1	1	1	1	1	1	1	1	1	1	1	96	96	1
YPTB2715	1	1	1	1	1	1	1	1	1	1	1	1	1	1	1	1	1	1	1	1	1	1	1	1	1	1	1	1	1	1	1	1	1	1	1
YPTB2716	107	109	107	108	1	1	107	108	140	108	108	106	1	1	1	108	1	108	107	107	1	1	1	107	1	1	1	1	1	1	1	1	108	108	107
YPTB2761	1	49	1	48	1	1	1	1	49	48	48	48	1	1	1	48	1	1	1	1	1	1	1	1	1	1	1	1	1	1	1	1	48	48	1
YPTB2775	1	112	1	114	1	1	1	112	112	114	114	109	1	1	1	114	1	108	1	1	1	1	1	1	1	1	1	1	1	1	1	1	114	114	1
YPTB2779	1	1	1	1	1	1	1	1	1	1	1	78	1	1	1	1	1	1	1	1	1	1	1	1	1	1	1	1	1	1	1	1	1	1	1
YPTB2781	1	1	1	1	1	1	1	86	1	1	1	1	84	84	1	1	84	1	1	1	1	1	1	1	84	84	1	1	1	1	1	84	1	1	1
YPTB2785	1	74	75	1	1	1	75	1	74	1	1	1	1	1	1	1	1	74	75	75	1	1	1	75	1	1	1	1	1	1	1	1	1	1	75
YPTB2789	1	102	1	102	1	1	1	103	102	102	102	102	102	102	1	102	102	1	1	1	1	1	1	1	102	102	1	1	1	1	1	102	102	102	1
YPTB2794	79	81	79	81	1	1	79	81	81	81	81	79	79	79	1	81	79	1	79	79	1	1	1	79	79	79	1	1	1	1	1	79	81	81	79
YPTB2797	67	68	67	68	1	1	67	69	68	68	68	1	1	1	1	68	1	1	67	67	1	1	1	67	1	1	1	1	1	1	1	1	68	68	67
YPTB2799	96	95	96	1	1	1	96	99	95	1	1	96	1	1	1	1	1	1	96	96	1	1	1	96	1	1	1	1	1	1	1	1	1	1	96
YPTB2819	1	1	1	1	1	125	1	1	1	1	1	1	1	1	125	1	1	1	1	1	125	125	125	1	1	1	125	125	125	125	125	1	1	1	1
YPTB2838	105	1	105	1	1	1	105	1	1	1	1	105	1	1	1	1	1	105	105	145	1	1	1	105	1	1	1	1	1	1	1	1	1	1	105
YPTB2853	1	1	1	1	1	1	1	1	1	1	1	1	1	1	1	1	1	1	1	1	1	1	1	1	1	1	1	1	1	1	1	1	1	1	1
YPTB2857	1	1	1	1	1	1	1	1	1	1	1	1	1	1	1	1	1	1	1	1	1	1	1	1	1	1	1	1	1	1	1	1	1	1	1
YPTB2859	1	107	1	111	1	1	1	1	107	107	111	106	1	1	1	107	1	107	1	1	1	1	1	1	1	1	1	1	1	1	1	1	111	111	1
YPTB2869	1	117	1	118	1	1	1	118	117	118	118	115	1	1	1	118	1	1	1	1	1	1	1	1	1	1	1	1	1	1	1	1	118	118	1
YPTB2872	1	69	1	69	1	1	1	69	69	69	69	1	1	1	1	69	1	1	1	1	1	1	1	1	1	1	1	1	1	1	1	1	69	69	1
YPTB2873	1	130	1	130	1	1	1	133	175	132	130	1	1	1	1	132	1	127	1	1	1	1	1	1	1	1	1	1	1	1	1	1	130	130	1
YPTB2885	1	77	1	79	1	1	1	79	77	79	79	1	1	1	1	79	1	1	1	1	1	1	1	1	1	1	1	1	1	1	1	1	79	79	1
YPTB2886	69	1	69	1	1	1	69	69	1	1	1	69	1	1	1	1	1	1	69	69	1	1	1	69	1	1	1	1	1	1	1	1	1	1	69
YPTB2887	93	91	93	94	1	1	93	92	91	94	94	92	1	1	1	94	1	94	93	93	1	1	1	93	1	1	1	1	1	1	1	1	94	94	93
YPTB2889	100	101	100	100	1	1	100	102	101	100	100	100	1	1	1	100	1	100	100	100	1	1	1	100	1	1	1	1	1	1	1	1	100	100	100
YPTB2890	1	1	59	1	1	1	59	1	1	1	1	1	1	1	1	1	1	1	59	59	1	1	1	59	1	1	1	1	1	1	1	1	1	1	59
YPTB2895	1	1	1	93	1	1	1	94	1	93	93	1	1	1	1	93	1	1	1	1	1	1	1	1	1	1	1	1	1	1	1	1	93	93	1
YPTB2897	1	1	1	1	1	1	1	1	1	1	1	1	1	1	1	1	1	1	1	1	1	1	1	1	1	1	1	1	1	1	1	1	1	1	1
YPTB2915	1	1	1	1	1	1	1	1	1	1	1	1	1	1	1	1	1	1	1	1	1	1	1	1	1	1	1	1	1	1	1	1	1	1	1
YPTB2929	1	70	1	70	1	1	1	1	70	70	70	68	1	1	1	70	1	68	1	1	1	1	1	1	1	1	1	1	1	1	1	1	70	70	1
YPTB2940	1	90	1	1	1	1	1	91	90	1	1	91	1	1	1	1	1	1	1	1	1	1	1	1	1	1	1	1	1	1	1	1	1	1	1
YPTB2942	1	126	1	128	1	436	1	122	126	128	128	122	1	1	436	128	1	123	1	1	436	436	436	1	1	1	436	436	436	436	436	1	128	128	1
YPTB2965	1	83	1	84	1	1	1	1	83	84	84	1	1	1	1	84	1	83	1	1	1	1	1	1	1	1	1	1	1	1	1	1	84	84	1
YPTB2966	1	87	1	1	1	1	1	1	87	1	1	1	1	1	1	1	1	1	1	1	1	1	1	1	1	1	1	1	1	1	1	1	1	1	110
YPTB2967	129	103	101	128	1	1	101	103	103	103	128	1	1	1	1	103	1	130	101	101	1	1	1	101	1	1	1	1	1	1	1	1	128	128	101
YPTB2973	99	101	99	129	1	1	99	101	101	101	129	1	1	1	1	101	1	1	99	99	1	1	1	99	1	1	1	1	1	1	1	1	129	129	99
YPTB2974	83	86	83	87	1	1	83	1	86	87	87	82	1	1	1	87	1	84	83	83	1	1	1	83	1	1	1	1	1	1	1	1	87	87	83
YPTB2975	1	1	1	86	1	1	1	87	1	86	86	86	1	1	1	86	1	87	1	1	1	1	1	1	1	1	1	1	1	1	1	1	86	86	1
YPTB2977	1	95	1	93	1	1	1	1	95	96	93	1	1	1	1	96	1	93	1	1	1	1	1	1	1	1	1	1	1	1	1	1	93	93	1
YPTB2978	1	122	1	122	1	1	1	122	122	122	122	122	1	1	1	122	1	122	123	123	1	1	1	123	1	1	1	1	1	1	1	1	122	122	1
YPTB2987	86	1	86	94	1	1	86	1	1	89	94	85	1	1	1	89	1	87	86	86	1	1	1	86	1	1	1	1	1	1	1	1	94	94	86
YPTB2993	1	1	1	100	1	1	1	1	1	100	100	1	1	1	1	100	1	98	1	1	1	1	1	1	1	1	1	1	1	1	1	1	100	100	1
YPTB2994	1	1	1	1	1	1	1	1	1	1	1	1	1	1	1	1	1	1	1	1	1	1	1	1	1	1	1	1	1	1	1	1	1	1	1
YPTB2997	81	81	81	81	1	1	81	81	81	81	81	82	1	1	1	81	1	81	81	81	1	1	1	81	1	1	1	1	1	1	1	1	81	81	81
YPTB2998	1	1	1	1	1	1	1	1	1	1	1	1	1	1	1	1	1	72	1	1	1	1	1	1	1	1	1	1	1	1	1	1	1	1	1
YPTB3003	55	55	55	1	1	1	55	1	55	1	1	1	1	1	1	1	1	55	55	55	1	1	1	55	1	1	1	1	1	1	1	1	1	1	55
YPTB3012	113	116	113	111	1	1	113	112	111	111	111	112	1	1	1	111	1	113	113	113	1	1	1	113	1	1	1	1	1	1	1	1	111	111	147
YPTB3017	1	1	1	1	1	1	1	1	1	1	1	1	1	1	1	1	1	84	1	1	1	1	1	1	1	1	1	1	1	1	1	1	1	1	1
YPTB3023	1	119	1	158	1	1	1	120	119	119	158	116	157	157	1	119	157	117	1	1	1	1	1	1	157	157	1	1	1	1	1	157	158	158	1
YPTB3033	1	1	1	1	1	1	1	1	1	1	1	1	1	1	1	1	1	98	1	1	1	1	1	1	1	1	1	1	1	1	1	1	1	1	126
YPTB3034	1	1	1	1	1	1	1	1	1	1	1	1	1	1	1	1	1	1	1	1	1	1	1	1	1	1	1	1	1	1	1	1	1	1	1
YPTB3035	1	139	1	141	1	1	1	142	139	141	141	1	1	1	1	141	1	136	1	1	1	1	1	1	1	1	1	1	1	1	1	1	141	141	1
YPTB3036	1	47	1	47	1	1	1	47	47	47	47	1	1	1	1	47	1	47	1	1	1	1	1	1	1	1	1	1	1	1	1	1	47	47	1
YPTB3042	140	141	140	140	1	1	140	142	141	140	140	1	1	1	1	140	1	138	140	140	1	1	1	140	1	1	1	1	1	1	1	1	140	140	140
YPTB3165	165	127	165	130	1	1	165	130	127	129	130	122	133	133	1	129	133	124	165	165	1	1	1	165	133	133	1	1	1	1	1	133	130	130	165
YPTB3167	97	1	97	1	1	1	97	1	1	1	1	97	1	1	1	1	1	1	97	97	1	1	1	97	1	1	1	1	1	1	1	1	1	1	97
YPTB3181	72	73	72	1	1	1	72	1	73	1	1	1	1	1	1	1	1	1	72	72	1	1	1	72	1	1	1	1	1	1	1	1	1	1	72
YPTB3182	124	119	116	121	1	1	116	122	119	121	121	115	115	115	1	121	115	1	116	116	1	1	1	116	115	115	1	1	1	1	1	115	121	121	124
YPTB3196	102	105	102	102	1	1	102	101	105	102	102	101	102	102	1	102	102	1	102	102	1	1	1	102	102	102	1	1	1	1	1	102	102	102	102
YPTB3197	100	100	100	100	1	1	100	100	100	100	100	100	100	100	1	100	100	1	100	100	1	1	1	100	100	100	1	1	1	1	1	100	100	100	100
YPTB3198	132	135	132	146	1	1	132	138	135	183	146	131	141	141	1	183	141	1	132	132	1	1	1	132	141	141	1	1	1	1	1	141	146	146	132
YPTB3205	95	1	95	95	1	1	95	96	1	97	95	1	1	1	1	97	1	95	95	95	1	1	1	95	1	1	1	1	1	1	1	1	95	95	95
YPTB3210	1	105	1	1	1	1	1	106	105	1	1	1	1	1	1	1	1	1	1	1	1	1	1	1	1	1	1	1	1	1	1	1	1	1	1
YPTB3215	1	1	95	77	1	1	1	1	1	1	77	70	1	1	1	1	1	1	1	1	1	1	1	1	1	1	1	1	1	1	1	1	77	77	1
YPTB3217	92	94	92	99	1	1	92	1	94	95	99	91	1	1	1	119	1	1	92	92	1	1	1	92	1	1	1	1	1	1	1	1	99	99	92
YPTB3218	108	111	108	111	1	1	108	110	111	111	111	107	1	1	1	111	1	1	108	108	1	1	1	108	1	1	1	1	1	1	1	1	111	111	108
YPTB3222	1	51	1	51	1	1	1	51	51	51	51	1	1	1	1	51	1	51	1	1	1	1	1	1	1	1	1	1	1	1	1	1	51	51	1
YPTB3225	1	48	1	47	1	1	1	48	48	47	47	1	1	1	1	47	1	47	1	1	1	1	1	1	1	1	1	1	1	1	1	1	47	47	1
YPTB3230	1	1	1	106	1	1	1	1	1	106	106	1	1	1	1	106	1	1	1	1	1	1	1	1	1	1	1	1	1	1	1	1	106	106	1
YPTB3313	74	76	74	74	1	1	74	74	76	74	74	1	1	1	1	74	1	75	74	74	1	1	1	74	1	1	1	1	1	1	1	1	74	74	74
YPTB3319	1	83	1	83	1	1	1	1	83	83	83	1	1	1	1	83	1	83	1	1	1	1	1	1	1	1	1	1	1	1	1	1	83	83	1
YPTB3320	84	84	84	1	1	1	84	86	84	1	1	83	1	1	1	1	1	84	84	84	1	1	1	84	1	1	1	1	1	1	1	1	1	1	84
YPTB3339	1	1	1	1	1	1	1	1	1	73	1	1	1	1	1	73	1	1	1	1	1	1	1	1	1	1	1	1	1	1	1	1	1	1	1
YPTB3340	1	59	1	56	1	1	1	1	59	56	56	1	1	1	1	56	1	56	1	1	1	1	1	1	1	1	1	1	1	1	1	1	56	56	1
YPTB3347	1	105	1	106	1	1	1	105	105	106	106	1	1	1	1	106	1	106	1	1	1	1	1	1	1	1	1	1	1	1	1	1	106	106	1
YPTB3352	1	72	1	72	1	1	1	72	72	72	72	1	1	1	1	72	1	72	1	1	1	1	1	1	1	1	1	1	1	1	1	1	72	72	1
YPTB3354	1	1	1	1	1	1	1	63	1	1	1	1	1	1	1	1	1	1	1	1	1	1	1	1	1	1	1	1	1	1	1	1	1	1	1
YPTB3357	139	142	139	144	1	1	139	201	142	144	144	138	1	1	1	144	1	140	139	139	1	1	1	139	1	1	1	1	1	1	1	1	144	144	139
YPTB3395	170	130	170	132	1	1	170	134	130	132	132	1	1	1	1	132	1	127	170	170	1	1	1	170	1	1	1	1	1	1	1	1	132	132	170
YPTB3396	69	69	69	69	1	1	69	68	69	69	69	1	1	1	1	69	1	69	69	69	1	1	1	69	1	1	1	1	1	1	1	1	69	69	69
YPTB3398	1	1	1	1	1	1	1	1	1	1	1	1	1	1	1	1	1	1	1	1	1	1	1	1	1	1	1	1	1	1	1	1	1	1	1
YPTB3407	1	1	1	1	1	1	1	123	1	1	1	1	1	1	1	1	1	121	1	1	1	1	1	1	1	1	1	1	1	1	1	1	1	1	1
YPTB3413	1	69	1	71	1	1	1	1	69	71	71	1	1	1	1	71	1	1	1	1	1	1	1	1	1	1	1	1	1	1	1	1	71	71	1
YPTB3416	1	1	1	1	1	1	1	1	1	1	1	1	1	1	1	1	1	1	1	1	1	1	1	1	1	1	1	1	1	1	1	1	1	1	1
YPTB3476	126	129	126	131	1	126	126	125	133	131	131	125	125	125	126	131	125	126	126	126	126	126	126	126	125	125	126	126	126	126	126	125	131	131	126

YPTB3478	112	114	112	116	1	1	112	115	160	116	116	1	1	1	1	116	1	112	112	112	1	1	1	112	1	1	1	1	1	1	1	1	116	116	112
YPTB3479	102	104	102	105	1	1	102	100	104	105	105	101	1	1	1	105	1	103	102	102	1	1	1	102	1	1	1	1	1	1	1	1	105	105	102
YPTB3485	1	1	1	1	1	1	1	1	1	1	1	1	1	1	1	1	1	1	1	1	1	1	1	1	1	1	1	1	1	1	1	1	1	1	1
YPTB3488	1	1	1	1	1	1	1	1	1	1	1	1	1	1	1	1	92	1	1	1	1	1	1	1		217	1	1	1	1	1	1	1	1	1
YPTB3491	1	101	1	102	1	1	1	1	124	102	102	99	1	1	1	102	1	100	1	1	1	1	1	1	1	1	1	1	1	1	1	1	102	102	1
YPTB3498	1	84	1	85	1	1	1	83	84	85	85	82	1	1	1	85	1	102	1	1	1	1	1	1	1	1	1	1	1	1	1	1	85	85	1
YPTB3503	129	132	129	134	1	129	129	127	132	134	134	1	1	1	129	134	1	130	129	129	129	129	129	129	1	1	129	129	129	129	129	1	134	134	129
YPTB3506	1	1	1	1	1	1	1	1	1	67	1	1	1	1	1	67	1	1	1	1	1	1	1	1	1	1	1	1	1	1	1	1	1	1	1
YPTB3508	1	1	1	1	1	1	1	1	1	1	1	1	1	1	1	1	1	1	1	1	1	1	1	1	1	1	1	1	1	1	1	1	1	1	1
YPTB3510	1	1	1	1	1	1	1	53	1	1	1	1	1	1	1	1	1	54	1	1	1	1	1	1	1	1	1	1	1	1	1	1	1	1	1
YPTB3518	1	1	1	1	1	1	1	1	1	1	1	1	1	1	1	1	1	1	1	1	1	1	1	1	1	1	1	1	1	1	1	1	1	1	1
YPTB3523	1	72	1	73	1	1	1	72	72	73	73	1	1	1	1	73	1	71	1	1	1	1	1	1	1	1	1	1	1	1	1	1	73	73	1
YPTB3524	1	1	1	77	1	1	1	77	1	77	77	1	1	1	1	77	1	1	1	1	1	1	1	1	1	1	1	1	1	1	1	1	77	77	1
YPTB3525	86	86	86	86	1	1	86	86	86	86	86	1	1	1	1	86	1	86	86	86	1	1	1	86	1	1	1	1	1	1	1	1	86	86	86
YPTB3526	319	114	112	114	1	1	112	116	114	114	114	1	1	1	1	114	1	113	112	112	1	1	1	112	1	1	1	1	1	1	1	1	114	114	112
YPTB3527	1	1	1	1	1	1	1	1	1	1	1	1	1	1	1	1	1	42	1	1	1	1	1	1	1	1	1	1	1	1	1	1	1	1	1
YPTB3528	61	62	61	61	1	1	61	1	62	61	61	1	1	1	1	61	1	61	61	61	1	1	1	61	1	1	1	1	1	1	1	1	61	61	61
YPTB3531	96	94	96	95	1	96	96	101	94	95	95	95	1	1	96	95	1	97	96	96	96	96	96	96	1	1	96	96	96	96	96	1	95	95	96
YPTB3533	1	1	1	64	1	1	1	64	1	64	64	1	1	1	1	64	1	64	1	1	1	1	1	1	1	1	1	1	1	1	1	1	64	64	1
YPTB3536	1	120	1	123	1	1	1	125	120	123	123	1	1	1	1	123	1	120	1	1	1	1	1	1	1	1	1	1	1	1	1	1	123	123	1
YPTB3538	1	72	1	71	1	75	1	1	72	71	71	1	1	1	75	71	1	71	1	1	75	75	75	1	1	1	75	75	75	75	75	1	71	71	1
YPTB3547	1	114	1	116	1	1	1	117	114	116	116	1	1	1	1	116	1	1	1	1	1	1	1	1	1	1	1	1	1	1	1	1	116	116	1
YPTB3548	1	1	1	1	1	1	1	1	1	1	1	1	1	1	1	1	1	1	1	1	1	1	1	1	1	1	1	1	1	1	1	1	1	1	1
YPTB3549	112	115	112	125	1	1	112	119	115	117	125	111	1	1	1	117	1	1	112	112	1	1	1	112	1	1	1	1	1	1	1	1	125	125	
YPTB3558	1	131	1	133	1	1	1	135	131	133	133	127	1	1	1	133	1	128	1	1	1	1	1	1	1	1	1	1	1	1	1	1	133	133	1
YPTB3561	119	122	119	119	1	1	119	123	122	119	119	118	1	1	1	119	1	120	119	119	1	1	1	119	1	1	1	1	1	1	1	1	119	119	119
YPTB3563	90	1	90	1	1	1	90	1	1	1	1	1	1	1	1	1	1	75	90	90	1	1	1	90	1	1	1	1	1	1	1	1	1	1	90
YPTB3565	1	95	1	1	1	1	1	92	95	1	1	1	1	1	1	1	1	89	1	1	1	1	1	1	1	1	1	1	1	1	1	1	1	1	1
YPTB3570	60	61	60	61	1	1	60	62	61	61	61	60	1	1	1	61	1	61	60	60	1	1	1	60	1	1	1	1	1	1	1	1	61	61	60
YPTB3574	129	133	129	1	1	1	129	136	133	1	1	1	1	1	1	1	1	130	129	129	1	1	1	129	1	1	1	1	1	1	1	1	1	1	129
YPTB3649	168	123	121	124	1	1	121	126	123	124	124	121	121	121	1	124	121	121	121	121	1	1	1	121	121	121	1	1	1	1	475	121	124	124	121
YPTB3660	96	1	96	1	1	1	96	1	1	1	1	1	1	1	1	1	1	1	96	96	1	1	1	96	1	1	1	1	1	1	1	1	1	1	96
YPTB3663	1	63	1	1	1	1	1	1	76	1	1	1	1	1	1	1	1	1	1	1	1	1	1	1	1	1	1	1	1	1	1	1	1	1	1
YPTB3665	1	1	1	1	1	1	1	1	1	1	1	1	1	1	1	1	1	1	1	1	1	1	1	1	1	1	1	1	1	1	1	1	1	1	1
YPTB3668	1	1	1	1	1	1	1	1	1	1	1	1	1	1	1	1	1	1	1	1	319	1	1	1	240	1	1	1	1	1	1	1	1	1	366
YPTB3674	1	1	1	1	1	1	1	1	1	1	1	1	1	1	1	1	1	1	1	1	1	1	1	1	1	1	1	1	1	1	1	1	1	1	1
YPTB3675	1	1	1	1	1	1	1	1	1	1	1	1	1	1	1	1	1	1	1	1	1	1	1	1	1	1	1	1	1	1	1	1	1	1	1
YPTB3676	1	1	1	1	1	1	1	1	1	1	1	1	1	1	1	1	1	1	1	1	1	1	1	1	1	1	1	1	1	1	1	1	1	1	1
YPTB3677	1	1	1	1	1	1	1	1	1	1	1	1	1	1	1	1	1	1	1	1	1	1	1	1	1	1	1	1	1	1	1	1	1	1	1
YPTB3678	1	60	1	1	1	1	1	1	63	1	1	1	1	1	1	1	1	1	1	1	1	1	1	1	1	1	1	1	1	1	1	1	1	1	1
YPTB3679	1	28	1	1	1	1	1	1	1	1	1	1	1	1	1	1	1	1	1	1	1	1	1	1	1	1	1	1	1	1	1	1	1	1	1
YPTB3680	1	1	1	1	1	1	1	1	1	1	1	1	1	1	1	1	1	1	1	1	1	1	1	1	1	1	1	1	1	1	1	1	1	1	1
YPTB3681	32	33	32	1	1	1	32	32	33	1	1	1	1	1	1	1	1	32	32	32	1	1	1	32	1	1	1	1	1	1	1	1	1	1	32
YPTB3682	1	1	1	1	1	1	1	1	1	1	1	1	1	1	1	1	1	1	1	1	1	1	1	1	1	1	1	1	1	1	1	1	1	1	1
YPTB3683	121	1	1	1	1	1	1	1	1	1	1	1	1	1	1	1	1	1	1	1	1	1	1	1	1	1	1	1	1	1	1	1	1	1	1
YPTB3684	1	1	1	1	1	1	1	1	1	1	1	1	1	1	1	1	1	1	1	1	1	1	1	1	1	1	1	1	1	1	1	1	1	1	1
YPTB3685	1	1	1	1	1	1	1	1	1	1	1	1	1	1	1	1	1	1	1	1	1	1	1	1	1	1	1	1	1	1	1	1	1	1	1
YPTB3686	1	1	1	1	1	1	1	1	1	1	1	1	1	1	1	1	1	1	1	1	1	1	1	1	1	1	1	1	1	1	1	1	1	1	1
YPTB3687	1	1	1	1	1	1	1	1	1	1	1	1	1	1	1	1	1	1	1	1	1	1	1	1	1	1	1	1	1	1	1	1	1	1	1
YPTB3688	1	1	1	1	1	1	1	1	1	1	1	1	1	1	1	1	1	1	1	1	1	1	1	1	1	1	1	1	1	1	1	1	1	1	1
YPTB3690	1	1	1	1	1	1	1	1	1	1	1	1	1	1	1	1	1	1	1	1	1	1	1	1	1	1	1	1	1	1	1	1	1	1	1
YPTB3691	1	1	1	1	1	1	1	1	1	1	1	1	1	1	1	1	1	29	1	1	1	1	1	1	1	1	1	1	1	1	1	1	1	1	1
YPTB3692	33	1	33	1	1	1	33	1	1	1	1	1	1	1	1	1	1	1	33	33	1	1	1	33	1	1	1	1	1	1	1	1	1	1	33
YPTB3693	1	1	1	1	1	1	1	1	1	1	1	1	1	1	1	1	1	1	1	1	1	1	1	1	1	1	1	1	1	1	1	1	1	1	1
YPTB3694	1	1	1	1	1	1	1	1	1	1	1	1	1	1	1	1	1	1	1	1	1	1	1	1	1	1	1	1	1	1	1	1	1	1	1
YPTB3695	42	1	42	1	1	1	42	1	1	1	1	1	1	1	1	1	1	1	42	42	1	1	1	42	1	1	1	1	1	1	1	1	1	1	42
YPTB3696	1	1	1	1	1	1	1	1	1	1	1	1	1	1	1	1	1	1	1	1	1	1	1	1	1	1	1	1	1	1	1	1	1	1	1
YPTB3697	1	1	1	47	1	1	1	1	1	47	47	1	1	1	1	47	1	1	1	1	1	1	1	1	1	1	1	1	1	1	1	1	47	47	1
YPTB3698	1	47	1	46	1	1	1	1	47	46	46	1	1	1	1	46	1	1	1	1	1	1	1	1	1	1	1	1	1	1	1	1	46	46	1
YPTB3699	1	1	1	1	1	1	1	1	1	1	1	1	1	1	1	1	1	1	1	1	1	1	1	1	1	1	1	1	1	1	1	1	1	1	1
YPTB3704	1	1	1	37	1	1	1	37	1	37	37	1	1	1	1	37	1	37	1	1	1	1	1	1	1	1	1	1	1	1	1	1	37	37	1
YPTB3705	1	1	1	1	1	1	1	1	1	1	1	1	1	1	1	1	1	1	1	1	1	1	1	1	1	1	1	1	1	1	1	1	1	1	1
YPTB3706	63	1	63	1	1	1	63	1	1	1	1	1	1	1	1	1	1	1	63	63	1	1	1	63	1	1	1	1	1	1	1	1	1	1	63
YPTB3709	82	1	82	102	1	1	82	1	1	84	102	1	1	1	1	84	1	1	82	82	1	1	1	82	1	1	1	1	1	1	1	1	102	102	82
YPTB3711	1	1	1	1	1	1	1	1	1	1	1	1	1	1	1	1	1	1	1	1	1	1	1	1	1	1	1	1	1	1	1	1	1	1	1
YPTB3714	176	124	176	126	1	1	176	119	124	126	126	119	122	122	1	126	122	176	176	176	1	1	1	176	122	122	1	1	1	1	1	122	126	126	176
YPTB3725	64	65	64	1	1	64	64	1	65	1	1	63	64	64	64	1	64	1	64	64	64	64	64	64	64	64	64	64	64	64	64	64	1	1	64
YPTB3729	1	53	1	1	1	53	1	53	53	1	1	53	53	53	53	1	53	1	1	1	53	53	53	1	53	53	53	53	53	53	53	53	1	1	1
YPTB3732	93	95	93	94	1	1	93	1	95	95	94	92	1	1	1	95	1	94	93	93	1	1	1	93	1	1	1	1	1	1	1	1	94	94	93
YPTB3734	80	81	80	1	1	1	80	1	81	1	1	1	1	1	1	1	1	1	80	80	1	1	1	80	1	1	1	1	1	1	1	1	1	1	80
YPTB3743	1	124	1	125	1	1	1	126	163	125	125	122	1	1	1	125	1	1	1	1	1	1	1	1	1	1	1	1	1	1	1	1	125	125	1
YPTB3746	110	1	110	109	1	110	110	109	1	109	109	110	110	110	110	109	110	1	110	110	110	110	110	110	110	110	110	110	110	110	110	110	109	109	110
YPTB3750	1	47	1	1	1	1	1	1	47	1	1	1	1	1	1	1	1	47	1	1	1	1	1	1	1	1	1	1	1	1	1	1	1	1	1
YPTB3760	1	1	1	1	1	1	1	1	1	1	1	72	1	1	1	1	1	1	1	1	1	1	1	1	1	1	1	1	1	1	1	1	1	1	1
YPTB3764	1	1	1	74	1	1	1	1	1	74	74	1	1	1	1	74	1	1	1	1	1	1	1	1	1	1	1	1	1	1	1	1	74	74	1
YPTB3786	1	141	1	1	1	1	1	137	141	143	1	137	145	145	1	143	145	138	1	1	1	1	1	1	145	145	1	1	1	1	1	145	1	1	177
YPTB3790	1	1	1	92	1	1	1	1	1	92	92	1	1	1	1	92	1	92	1	1	1	1	1	1	1	1	1	1	1	1	1	1	92	92	1
YPTB3791	94	96	94	97	1	94	94	99	96	97	97	94	94	94	94	97	94	1	94	94	94	94	94	94	94	94	94	94	94	94	94	94	97	97	94
YPTB3840	1	117	1	117	1	116	1	117	117	117	117	1	116	116	116	117	116	115	1	1	116	116	116	1	116	116	116	116	116	116	116	116	117	117	1
YPTB3847	1	1	1	93	1	1	1	1	1	93	93	92	1	1	1	93	1	1	1	1	1	1	1	1	1	1	1	1	1	1	1	1	93	93	1
YPTB3914	91	94	91	95	1	1	91	96	94	95	95	90	1	1	1	95	1	1	91	91	1	1	1	91	1	1	1	1	1	1	1	1	95	95	91
YPTB3915	99	99	99	99	99	99	99	99	99	99	99	99	99	99	99	99	99	99	99	99	99	99	99	99	99	99	99	99	99	99	99	99	99	99	99
YPTB3916	137	141	137	140	1	1	137	192	141	143	140	136	1	1	1	143	1	138	137	137	1	1	1	137	1	1	1	1	1	1	1	1	140	140	137
YPTB3920	1	81	1	83	1	1	1	1	81	83	83	80	1	1	1	83	1	81	1	1	1	1	1	1	1	1	1	1	1	1	1	1	83	83	1
YPTB3921	1	61	1	1	1	1	1	1	61	1	1	1	1	1	1	1	1	1	1	1	1	1	1	1	1	1	1	1	1	1	1	1	1	1	1
YPTB3940	129	133	129	134	1	1	129	136	133	134	134	128	1	1	1	134	1	130	129	129	1	1	1	129	1	1	1	1	1	1	1	1	134	134	129
YPTB3942	95	98	95	98	1	1	95	98	98	98	98	1	1	1	1	98	1	96	95	95	1	1	1	95	1	1	1	1	1	1	1	1	98	98	95
YPTB3945	1	1	1	1	1	1	1	1	1	1	1	1	1	1	1	1	1	1	1	1	1	1	1	1	1	1	1	1	1	1	1	1	1	1	1
YPTB3946	1	1	1	1	1	1	1	1	1	1	1	1	1	1	1	1	1	39	1	1	1	1	1	1	1	1	1	1	1	1	1	1	1	1	1
YPTB3947	1	29	1	1	1	1	1	1	29	1	1	1	1	1	1	1	1	28	1	1	1	1	1	1	1	1	1	1	1	1	1	1	1	1	1
YPTB3950	1	1	1	104	1	1	1	99	1	103	104	1	1	133	1	103	133	100	1	1	1	1	1	1	1	1	1	1	1	1	1	1	104	104	1
YPTB3960	1	1	1	1	1	1	1	1	1	1	1	1	1	1	1	1	1	91	1	1	1	1	1	1	1	1	1	1	1	1	1	1	1	1	1
YPTB3961	1	97	1	1	1	1	1	1	97	1	1	1	1	1	1	1	1	97	1	125	1	1	1	125	1	1	1	1	1	1	1	1	1	1	1
YPTB3965	1	118	1	122	1	1	1	123	118	122	122	119	1	1	1	122	1	120	1	1	1	1	1	1	1	1	1	1	1	1	1	1	122	122	1
YPTB3966	1	1	1	53	1	1	1	1	1	53	53	1	1	1	1	53	1	54	1	1	1	1	1	1	1	1	1	1	1	1	1	1	53	53	1
YPTB3969	101	101	101	130	1	1	101	101	101	103	130	1	1	1	1	103	1	131	101	101	1	1	1	101	1	1	1	1	1	1	1	1	130	130	101
YPTB3970	1	1	1	1	1	1	1	1	1	1	1	1	1	1	1	1	1	46	1	1	1	1	1	1	1	1	1	1	1	1	1	1	1	1	1
YPTB3971	1	1	1	1	1	1	1	1	1	1	1	1	1	1	1	1	1	1	1	1	1	1	1	1	1	1	1	1	1	1	1	1	1	1	1
YPTB3973	1	1	1	1	1	1	1	1	1	1	1	1	1	1	1	1	1	1	1	1	1	1	1	1	1	1	1	1	1	1	1	1	1	1	1

Table S3: Pairwise distance matrix cgMLST-based and SNP-based obtained comparing the 39 *Y. pseudotuberculosis* isolates belonging to the lineage 16.

cgMLST distance	Isolation Date	IP32953	IP32997	IP32998	IP33018	IP33060	IP33094	IP33357	IP33362	IP33363	IP33443	IP33444	IP33467	IP33966	IP34864	IP35660	IP37713	IP37784	IP39187	IP40258	IP41727	IP32400	IP32759	IP32780	IP31871	IP32627	IP32641	IP32006	IP43278	IP43304	IP31922	IP43492	IP43951	IP43952	IP43989	IP44094	IP44110	IP44154	IP44180	IP44214
IP32953	1990	0																																						
IP32997	1993	105	0																																					
IP32998	1993	29	102	0																																				
IP33018	1994	105	13	102	0																																			
IP33060	1997	31	103	8	103	0																																		
IP33094	2000	112	23	109	18	110	0																																	
IP33357	2004	114	71	110	71	111	80	0																																
IP33362	2005	31	102	8	102	10	109	110	0																															
IP33363	2005	29	101	6	101	8	108	109	5	0																														
IP33443	2007	114	24	111	19	112	3	81	110	110	0																													
IP33444	2007	109	18	106	17	107	27	75	106	105	28	0																												
IP33467	2009	113	70	109	70	110	79	6	108	108	80	74	0																											
IP33966	2010	31	103	10	103	12	110	111	11	9	112	107	110	0																										
IP34864	2012	29	102	8	102	10	109	110	9	8	111	106	109	6	0																									
IP35660	2014	112	21	109	20	110	30	76	109	108	31	9	75	110	109	0																								
IP37713	2016	115	30	112	29	113	38	80	112	111	39	18	79	113	112	13	0																							
IP37784	2016	35	117	41	117	43	124	125	43	41	126	120	124	43	41	123	123	0																						
IP39187	2017	106	16	104	15	105	25	74	104	103	26	18	73	105	103	21	29	117	0																					
IP40258	2018	113	21	110	20	111	30	78	110	109	31	9	77	111	110	4	13	124	21	0																				
IP41727	2019	105	13	102	12	102	22	70	102	101	23	17	68	103	102	20	28	117	15	20	0																			
IP32400	1976	28	110	34	110	36	117	118	36	34	119	114	117	36	34	117	118	25	112	118	110	0																		
IP32759	1985	33	106	10	106	12	113	113	11	9	114	110	113	14	12	111	116	45	108	114	106	38	0																	
IP32780	1986	107	15	104	14	105	24	73	104	103	25	17	72	105	104	20	29	118	5	20	14	112	108	0																
IP31871	1969	114	13	111	22	112	32	80	110	110	32	26	79	112	111	29	39	126	25	30	22	119	115	24	0															
IP32627	1982	116	24	112	23	113	32	82	111	111	33	12	81	113	112	13	22	127	24	13	23	121	116	23	32	0														
IP32641	1983	105	26	102	26	103	35	71	101	101	34	31	70	103	102	32	43	116	29	34	26	110	106	28	34	35	0													
IP32006	1970	29	102	6	102	4	109	110	8	6	111	106	109	10	8	109	112	41	104	110	101	34	10	104	111	112	102	0												
IP43278	2020	28	100	7	100	9	107	108	8	6	109	104	107	8	7	107	109	40	101	108	100	33	11	102	109	110	100	7	0											
IP43304	2020	145	96	142	96	143	104	109	141	140	104	100	108	143	142	104	111	155	99	104	95	149	146	98	106	106	97	142	140	0										
IP31922	1970	108	17	105	16	106	25	73	105	104	27	21	72	106	105	24	32	120	19	24	16	113	109	18	26	27	30	105	103	99	0									
IP43492	2020	147	98	144	98	145	106	111	142	142	105	101	110	145	144	106	113	157	101	106	97	151	148	100	106	107	98	144	142	2	101	0								
IP43951	2020	146	98	143	98	144	106	110	142	141	106	102	109	143	143	105	113	155	101	106	97	150	147	100	107	108	98	143	141	2	101	4	0							
IP43952	2020	145	96	142	96	143	104	109	141	140	104	100	108	142	142	104	111	154	99	104	95	149	146	98	106	106	97	142	140	0	99	2	2	0						
IP43989	2020	146	97	143	97	144	105	110	142	141	105	101	109	143	143	105	112	154	100	105	96	150	147	99	107	107	98	143	141	1	100	3	3	1	0					
IP44094	2020	145	97	142	97	143	105	109	141	140	105	101	108	142	142	104	112	154	100	105	96	149	146	99	106	107	97	142	140	1	100	3	2	1	2	0				
IP44110	2020	146	97	143	97	144	105	110	142	139	105	101	109	143	143	105	112	155	100	105	96	150	146	99	107	107	98	143	141	1	100	3	3	1	2	2	0			
IP44154	2020	145	96	142	96	143	104	109	141	140	104	100	108	142	142	104	111	154	99	104	95	149	146	98	106	106	97	142	140	0	99	2	2	0	1	1	1	0		
IP44180	2020	146	97	143	97	144	105	110	142	140	105	101	109	143	143	105	112	155	100	105	96	150	146	99	107	107	98	143	141	1	100	3	3	1	2	2	1	1	0	
IP44214	2020	148	99	145	99	146	107	112	143	143	106	103	111	145	145	107	114	157	102	107	98	152	149	101	108	108	99	145	143	3	102	4	5	3	4	4	4	3	4	0

SNP distance	Isolation Date	IP32953	IP32997	IP32998	IP33018	IP33060	IP33094	IP33357	IP33362	IP33363	IP33443	IP33444	IP33467	IP33966	IP34864	IP35660	IP37713	IP37784	IP39187	IP40258	IP41727	IP32400	IP32759	IP32780	IP31871	IP32627	IP32641	IP32006	IP43278	IP43304	IP31922	IP43492	IP43951	IP43952	IP43989	IP44094	IP44110	IP44154	IP44180	IP44214
IP32953	1990	0																																						
IP32997	1993	243	0																																					
IP32998	1993	65	222	0																																				
IP33018	1994	245	46	224	0																																			
IP33060	1997	69	226	18	228	0																																		
IP33094	2000	244	47	223	43	227	0																																	
IP33357	2004	267	162	246	164	250	163	0																																
IP33362	2005	69	226	18	228	22	227	250	0																															
IP33363	2005	69	226	18	228	22	227	250	0	0																														
IP33443	2007	244	47	223	43	227	4	163	227	227	0																													
IP33444	2007	243	46	222	48	226	47	162	226	226	47	0																												
IP33467	2009	268	163	247	165	251	164	9	251	251	164	163	0																											
IP33966	2010	71	228	20	230	24	229	252	24	24	229	228	253	0																										
IP34864	2012	71	228	20	230	24	229	252	24	24	229	228	253	12	0																									
IP35660	2014	243	46	222	48	226	47	162	226	226	47	22	163	228	228	0																								
IP37713	2016	242	45	221	47	225	46	161	225	225	46	21	162	227	227	5	0																							
IP37784	2016	85	266	88	268	92	267	290	92	92	267	266	291	94	94	266	265	0																						
IP39187	2017	233	36	212	38	216	37	152	216	216	37	32	153	218	218	32	31	256	0																					
IP40258	2018	245	48	224	50	228	49	164	228	228	49	24	165	230	230	8	7	268	34	0																				
IP41727	2019	244	43	223	49	227	48	163	227	227	48	47	164	229	229	47	46	267	37	49	0																			
IP32400	1976	83	264	86	266	90	265	288	90	90	265	264	289	92	92	264	263	72	254	266	265	0																		
IP32759	1985	71	228	20	230	24	229	252	24	24	229	228	253	26	26	228	227	94	218	230	229	92	0																	
IP32780	1986	235	38	214	40	218	39	154	218	218	39	34	155	220	220	34	33	258	4	36	39	256	220	0																
IP31871	1969	254	33	233	57	237	58	173	237	237	58	57	174	239	239	57	56	277	47	59	54	275	239	49	0															
IP32627	1982	248	51	227	53	231	52	167	231	231	52	27	168	233	233	21	20	271	37	23	52	269	233	39	62	0														
IP32641	1983	425	250	404	252	408	251	344	408	408	251	250	345	410	410	250	249	448	240	252	251	446	410	242	261	255	0													
IP32006	1970	65	222	14	224	6	223	246	18	18	223	222	247	20	20	222	221	88	212	224	223	86	20	214	233	227	404	0												
IP43278	2020	69	226	18	228	22	227	250	22	22	227	226	251	22	22	226	225	92	216	228	227	90	24	218	237	231	408	18	0											
IP43304	2020	723	620	702	622	706	621	644	706	706	621	620	645	706	708	620	619	746	610	622	621	744	708	612	631	625	800	702	706	0										
IP31922	1970	248	47	227	53	231	52	167	231	231	52	51	168	233	233	51	50	271	41	53	46	269	233	43	58	56	255	227	231	625	0									
IP43492	2020	724	621	703	623	707	622	645	707	707	622	621	646	707	709	621	620	747	611	623	622	745	709	613	632	626	801	703	707	1	626	0								
IP43951	2020	723	620	702	622	706	621	644	706	706	621	620	645	706	708	620	619	746	610	622	621	744	708	612	631	625	800	702	706	0	625	1	0							
IP43952	2020	723	620	702	622	706	621	644	706	706	621	620	645	706	708	620	619	746	610	622	621	744	708	612	631	625	800	702	706	0	625	1	0	0						
IP43989	2020	723	620	702	622	706	621	644	706	706	621	620	645	706	708	620	619	746	610	622	621	744	708	612	631	625	800	702	706	0	625	1	0	0	0					
IP44094	2020	723	620	702	622	706	621	644	706	706	621	620	645	706	708	620	619	746	610	622	621	744	708	612	631	625	800	702	706	0	625	1	0	0	0	0				
IP44110	2020	723	620	702	622	706	621	644	706	706	621	620	645	706	708	620	619	746	610	622	621	744	708	612	631	625	800	702	706	0	625	1	0	0	0	0	0			
IP44154	2020	723	620	702	622	706	621	644	706	706	621	620	645	706	708	620	619	746	610	622	621	744	708	612	631	625	800	702	706	0	625	1	0	0	0	0	0	0		
IP44180	2020	723	620	702	622	706	621	644	706	706	621	620	645	706	708	620	619	746	610	622	621	744	708	612	631	625	800	702	706	0	625	1	0	0	0	0	0	0	0	
IP44214	2020	725	622	704	624	708	623	646	708	708	623	622	647	708	710	622	621	748	612	624	623	746	710	614	633	627	802	704	708	2	627	3	2	2	2	2	2	2	2	0

Figure S1: Repartition of the different *Y. pseudotuberculosis* lineages in France according to the year of isolation.

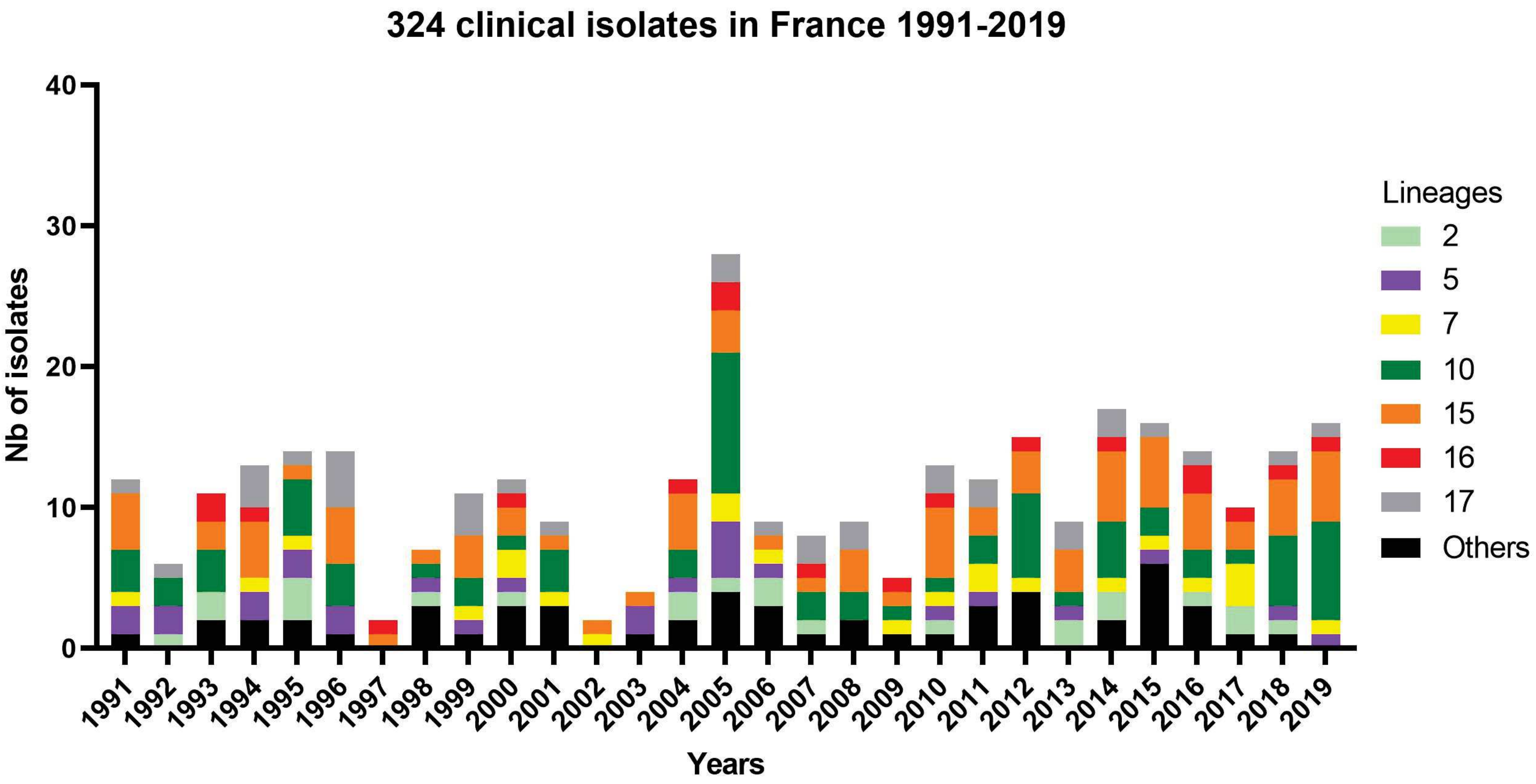

Figure S2: Timeline of the lineage 16 isolates during summer 2020. Number between brackets correspond to the isolation month.

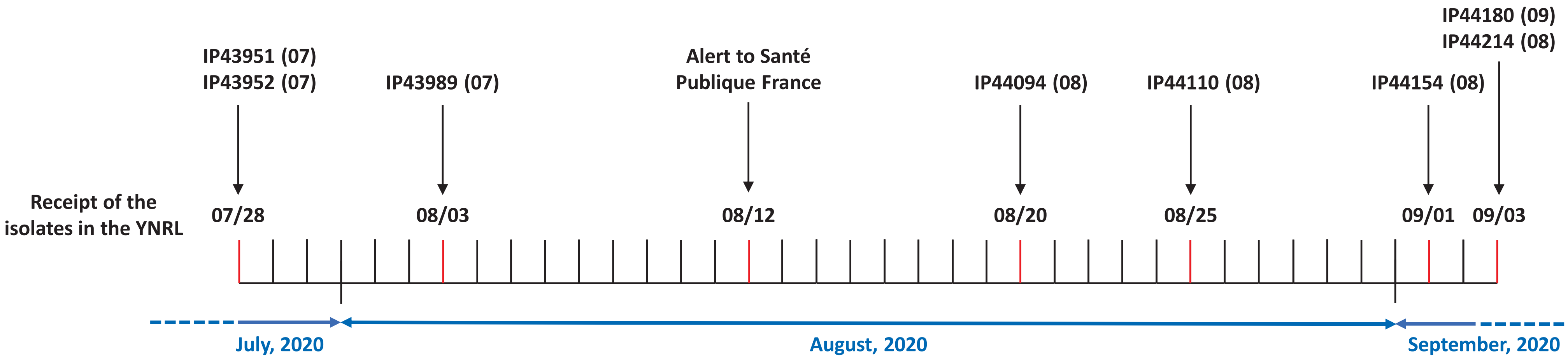

Supplement: Supplemental file 1 — Supplemental material. Download spectrum.01145-22-s0001.pdf, PDF file, 7.2 MB [file spectrum.01145-22-s0001.pdf]
